# The molecular steps of citrinin biosynthesis in fungi†
†Electronic supplementary information (ESI) available: All experimental, chromatographic, bioinformatic and spectroscopic details. See DOI: 10.1039/c5sc04027b


**DOI:** 10.1039/c5sc04027b

**Published:** 2015-12-17

**Authors:** Yi He, Russell J. Cox

**Affiliations:** a College of Food Science and Technology , Huazhong Agricultural University , Wuhan 430070 , Hubei Province , P. R. China; b Institut für Organische Chemie , Leibniz Universität Hannover , Schneiderberg 1B , 30167 Hannover , Germany . Email: russell.cox@oci.uni-hannover.de; c School of Chemistry , University of Bristol , Cantock's Close , Bristol , UK BS8 1TS, UK

## Abstract

The individual steps of citrinin **1** biosynthesis in *Monascus ruber* M7 were determined by a combination of targeted gene knockout and heterologous gene expression in *Aspergillus oryzae*. The pathway involves the synthesis of an unreduced trimethylated pentaketide **10** by a non-reducing polyketide synthase (nrPKS) known as CitS. Reductive release yields the keto-aldehyde **2** as the first enzyme-free intermediate. The nrPKS appears to be assisted by an as-yet cryptic hydrolysis step catalysed by CitA which was previously wrongly annotated as an oxidase. CitB is a non-heme iron oxidase which oxidises the 12-methyl of **2** to an alcohol. Subsequent steps are catalysed by CitC which oxidises the 12-alcohol to an aldehyde and CitD which converts the 12-aldehyde to a carboxylic acid. Final reduction of C-3 by CitE yields citrinin. The pathway rules out alternatives involving intramolecular rearrangements, and fully defines the molecular steps for the first time and corrects previous errors in the literature. The activity of CitB links the pathway to fungal tropolone biosynthesis and the observation of aminated shunt products links the pathway to azaphilone biosynthesis. Production of citrinin by coordinated production of CitS + CitA–CitE in the heterologous host *A. oryzae*, in which each gene was driven by a constitutive promoter, was achieved in high yield.

## Abstract 2

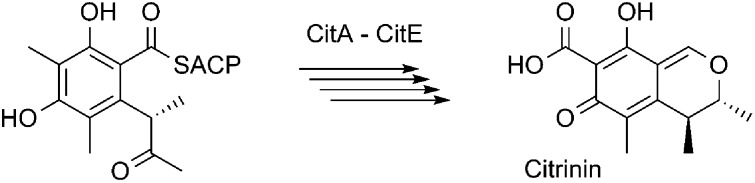
Heterologous expression of the citrinin polyketide synthase, CitS, plus the tailoring enzymes CitA–CitE from *Monascus ruber* has fully elucidated the biosynthetic pathway to citrinin for the first time, showing relationships to tropolone, azaphilone and sorbicillinoid biosynthetic pathways in fungi.

## Introduction

Like many mycotoxins, citrinin **1** was discovered by Harold Raistrick and coworkers in the 1930s.^1^ Its structure was solved by Whalley and coworkers in 1948.^2^**1** was isolated first from *Penicillium citrinum*, but is also known to be produced by other *Penicillium* species^3^ as well as species of *Monascus* including *M. ruber* and *M. purpureus*.^4^ It is a potent mycotoxin with nephrotoxic activities due to inhibition of respiration complex III.^5^ Citrinin was among the first compounds to be identified as a polyketide and its biosynthesis was investigated by Birch, Whalley and others during the 1950s using radioisotopes,^6^ and more latterly by Staunton,^7^ Sankawa^8^ and others^9^ using stable isotopes and NMR during the 1980s and 1990s. The gene cluster for citrinin, centered on the 7.9 kb gene *pksCT* which encodes an iterative type I non-reducing polyketide synthase (nr-PKS), was discovered in *M. purpureus* during the mid 2000s by Nihira and coworkers.^10^,^11^ Near-identical clusters have been discovered in *M. ruber*, *M. aurantiacus* and *Penicillium expansum*.

Remarkably little is known, however, of the individual chemical assembly steps of citrinin biosynthesis, and many assumptions and misleading interpretations have appeared in the literature (see ESI†).

Citrinin is widely regarded as a model compound, especially in the arena of fungal polyketide biosynthesis, where it is related to many bioactive metabolites such as the azaphilones,^12^ sorbicillinoids^13^ and tropolones.^14^ We thus sought to definitively clarify its biosynthesis. For this reason we decided to perform a thorough and systematic investigation of the citrinin cluster from *Monascus ruber* M7 using gene knockout and heterologous expression strategies.

Key questions focus on the identity of the product of the PKS, the order of the post-PKS tailoring steps, the precise limits of the citrinin gene cluster, and the relationship between the citrinin biosynthetic pathway and those of other fungal metabolites. Furthermore, a significant number of errors have appeared in the literature regarding citrinin biosynthesis and we wished to correct these. For example the frequently repeated assignment of *ctnB* as an oxidoreductase appeared unlikely;^7^,^15^ bizarre interpretations of labelling experiments in *M. ruber* appeared to justify the proposal of unprecedented intramolecular rearrangements during the formation of **1** (see ESI†);^16^ and errors in diagrams propagate incorrect conclusions about the biosynthetic steps (see ESI†).^3^

## Results

**Fig. 1:**
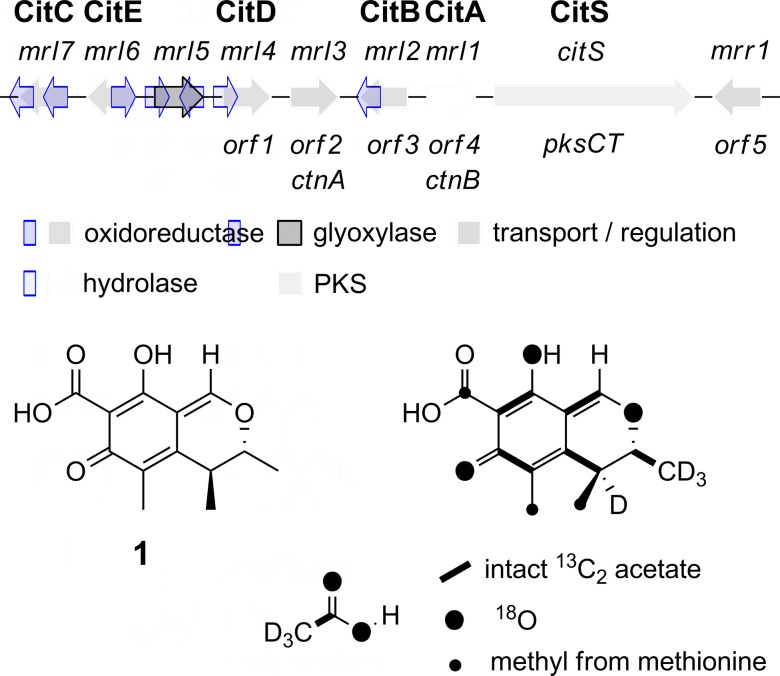
The citrinin gene cluster from *Monascus* species and the labelling pattern of citrinin deduced from isotopic feeding experiments.

We began by analysing the known gene clusters for citrinin biosynthesis from *Penicillium* and *Monascus* species (Fig. 1). Comparisons show that a minimal set of conserved genes likely to be involved in biosynthesis include: *citS*, encoding an nr-PKS^17^ (citrinin synthase) with SAT, KS, AT, PT, ACP, C-MeT and R domains; *mrl1*,^18^ encoding a serine hydrolase of no known function; *mrl2*, encoding a non-heme Fe(ii) dependent oxygenase similar to TropC from stipitatic acid biosynthesis known to be involved in oxidative ring expansion;^14^,^19^ *mrl4*, encoding an NAD(P)^+^ dependent aldehyde dehydrogenase; *mrl6*, encoding a short-chain dehydrogenase; and another NAD(P)^+^-dependent oxidoreductase encoded by *mrl7*. Other genes nearby such as *mrr1* (encoding a likely transporter), *mrl3* (encoding a transcription factor) and *mrl5* (glyoxylase-like domain) and genes further downstream of *mrr*1 or upstream of *mrl7* appeared to have no likely role in catalysis and were not considered further.

**Fig. 2:**
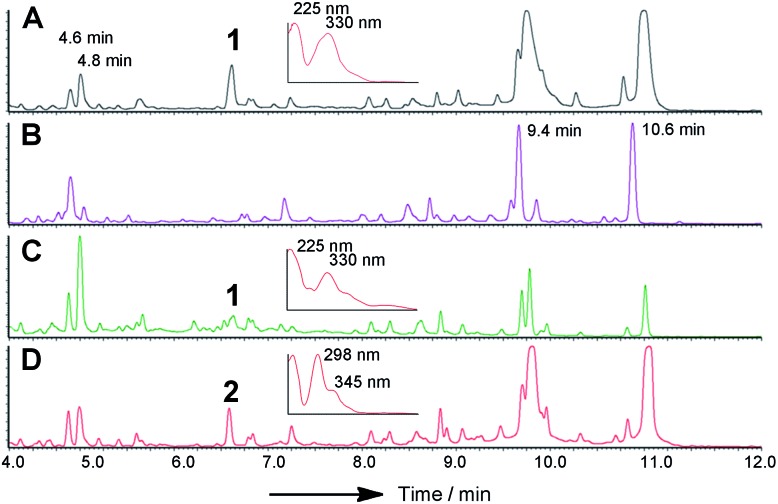
LCMS chromatograms (DAD trace) of organic extracts from targeted KO experiments in *M. ruber* M7 Δ*ku80*: (A) WT, inset shows DAD spectrum of citrinin 6.4 min; (B) Δ*citS*; (C) Δ*mrl1*, inset shows DAD spectrum of citrinin 6.4 min; (D) Δ*mrl2*, inset shows DAD spectrum of compound **2**, 6.4 min. Peaks at 4.6, 4.8, 9.4 and 10.6 min are due to other *M. ruber* polyketides unrelated to citrinin.

Knockout (KO) of *citS* encoding the citrinin PKS in *Monascus purpureus* has already been reported to abolish citrinin biosynthesis.^20^ We repeated this in *M. ruber* M7 using a KO strategy based on insertion of a neomycin antibiotic resistance cassette in the target gene *via* agrobacterium mediated transformation and selection on PDA plates containing the antibiotic G418 (15 μg mL^–1^). This was facilitated by use of a Δ*ku80* strain as previously reported.^21^ As expected citrinin biosynthesis was totally abolished (Fig. 2).

**Fig. 3:**
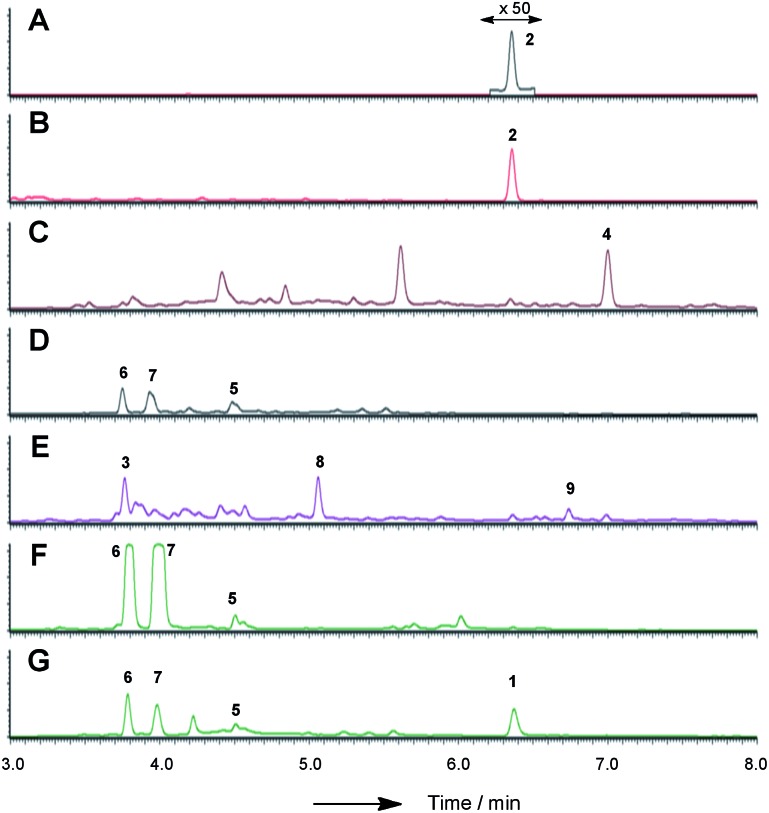
LCMS chromatograms (UV, 350 nm) of organic extracts from heterologous expression experiments in *A. oryzae* NSAR1: (A) *A. oryzae* + *citS*; (B) *A. oryzae* + *citS* + *mrl1*; (C) *A. oryzae* + *citS* + *mrl1* + *mrl2*; (D) *A. oryzae* + *citS* + *mrl1* + *mrl2* + *mrl4*; (E) *A. oryzae* + *citS* + *mrl1* + *mrl2* + *mrl7*; (F) *A. oryzae* + *citS* + *mrl1* + *mrl2* + *mrl7* + *mrl4*; (G) *A. oryzae* + *citS* + *mrl1* + *mrl2* + *mrl7* + *mrl4* + *mrl6*. All chromatograms are presented at the same scale. See ESI† for further detail.

Repeating the procedure with *mrl1* led to a significant reduction (>95%), but not abolition, of citrinin biosynthesis, and no new compounds appeared in the LCMS chromatograms of these mutants. KO of *mrl2*, however, led to complete absence of citrinin and the production of a new compound at the same retention time which was distinguishable by its UV and mass spectra. This new compound was purified and identified by HRMS, 1D and 2D NMR as the ketoaldehyde **2** (see ESI†). Further KO experiments were performed using the same methodology targeted at *mrl4*, *mrl6* and *mrl7*. In each case abolition of citrinin biosynthesis was observed, but no significant new peaks were detected in the LCMS chromatograms despite genetic analysis showing that the deletions had been created as expected.

In order to gain more detail on the function of the PKS and the tailoring enzymes, the cluster was reconstituted in the heterologous host *Aspergillus oryzae* using the modular expression system described by Lazarus and coworkers.^22^ Initial work focussed on the expression of the PKS itself in order to determine the first enzyme-free intermediate. Previous analysis of the PKS gene suggested that it contained a 56 bp intron located at 640 bp of *citS*. This was removed during yeast-recombination-mediated construction of an expression system (see ESI†). Thus the vector pTYGS arg *citS* was created in which the inducible promoter *P*_*amyB*_ was used to drive expression of *citS*. Transformation of this system into *A. oryzae* NSAR1 (ref. 23) led to the production of low levels of the ketoaldehyde **2** (0.8 mg L^–1^).

In previous heterologous experiments using *A. oryzae* as a host we have usually observed high yields of secondary metabolites.^24^ However we are aware that *A. oryzae* can sometimes have problems in splicing some introns from heterologous genes.^25^ Although we had removed one intron during cloning we could not be sure that all introns in *citS* had been located. We therefore sequenced cDNA prepared from the native host *M. ruber* and found clear evidence for a second intron located between the ACP- and R-domain-encoding regions of the gene. Careful and repeated sequencing indicated this to be 62 base-pairs in length meaning that when spliced the remaining R-domain located downstream would be out-of frame with respect to the rest of the protein and thus unable to form a functional reductive domain (see ESI†). The sequencing results, however, showed a mixed population of transcripts – spliced transcript formed the majority, but unspliced (and thus potentially catalytically active) transcript was clearly present as a minority population. Exhaustive control experiments indicated that this was not caused by contamination with gDNA. We repeated this analysis in the *A. oryzae citS* expression system and found that *A. oryzae* treats the intron in the same way, again giving a mixed population of transcripts. Construction of an expression clone in which the 62 bp intron sequence had been removed resulted in no production of **2** or any other new compounds. However, removal of 60 bp (*i.e*. in-frame) resulted in an active synthase capable of producing **2** at the same titre as the WT system (see ESI†).

We next co-expressed *citS* with *mrl1* (exp. 2, Table 1) using the same promoter system for *citS* and *P*_*adh*_ from *A. oryzae*^26^ for *mrl1*. These clones proved to be very good producers of the ketoaldehyde **2** producing approximately 15 mg L^–1^.

**Table 1:** Summary of heterologous expression experiments. See ESI for details of construction of expression vectors and detailed chromatograms of each experiment. *Minor component

Exp.	*citS*	*mrl1*/CitA	*mrl2*/CitB	*mrl4*/CitD	*mrl6*/CitE	*mrl7*/CitC	Products
1	X	—	—	—	—	—	**2**
2	X	X	—	—	—	—	**2**, 3*
3	X	X	X	—	—	—	2*, 3*, **4**
4	X	X	X	X	—	—	5*, 6*, 7*
5	X	X	X	—	—	X	2*, **3**, 4*, **8**, **9**
6	X	X	X	—	X	—	**2**
7	X	X	X	X	—	X	5*, **6**, **7**
8	X	X	X	—	X	X	2*, **3**, 4*, **8**
9	X	X	X	X	X	—	1*, 5*, **6**, **7**
10	X	X	X	X	X	X	**1**, 2*, 4*, 5*, **6**, **7**

In addition shunt compounds were observed such as **3** which was isolated and purified (see ESI†) and identified as the product of aldehyde reduction by full NMR and HRMS analysis.

Bioinformatic analysis of *mrl1* indicated two possible start codons differing by 52 codons. We thus also cloned the shorter possibility and expressed this with *citS*. Since the shorter clone proved to be just as active as the longer clone, and bioinformatic analysis suggested that the initial 52 residues of the longer peptide were not highly homologous to other known proteins we concluded that the shorter ORF is likely to be the true coding region and used this for further experiments.

Introduction of *mlr2*, encoding the non-heme iron oxygenase, driven by the *P*_*adh*_ promoter, then led to the production of the alcohol **4**, again in high titre (16 mg L^–1^, exp. 3), which was purified and fully characterised by HRMS, 1D and 2D NMR (see ESI†). Another compound eluting at 5.6 min showed a similar LCMS profile to **4** but could not be identified due to instability during purification.

We next expressed the *citS* + *mrl1* + *mrl2* core genes plus all 7 possible combinations of the remaining genes *mrl4*, *mrl6* and *mrl7* (Table 1). Addition of *mrl4* alone (exp. 4) to the core genes led to the weak production of compounds **5** (2.5 mg L^–1^), **6** (3.6 mg L^–1^) and **7** (4.6 mg L^–1^). Compound **6** was isolated as a mixture of interconverting diastereomers shown to be the enamine aminal **6** by HRMS and full NMR structural determination (see ESI†). At this stage compound **7** proved highly unstable and could not be identified, but was isolated later (exp. 7, *vide infra*). Addition of *mrl6* to the core genes (exp. 6) produced no additional new compounds that were detected.

Addition of *mrl*7 alone (exp. 5) gave chromatograms which were substantially similar to WT *A. oryzae* NSAR, with either very low or zero concentrations of compounds **2** and **4**. This showed that **4** was probably converted to a reactive intermediate which was either degraded or converted to numerous shunt products. Close examination of the mass chromatogram revealed a new peak at 5.1 minutes with the same mass as compound **4** (*m*/*z* 252, 7.0 min). Purification revealed this to be the hemiacetal **8** (18 mg L^–1^) which appeared to equilibrate to a mixture of diastereomers. In the presence of methanol (for example during workup) the methyl acetal **9** (as two diastereomers) was also detected by HRMS analysis, but rapid degradation prevented its full characterisation.

Coexpression of *mrl7* and *mrl4* (exp. 7) with the core genes provided **6** (23 mg L^–1^) and **7** (30 mg L^–1^) as the major products in high titre. Although **7**, the *N*-(2-hydroxyethyl) congener, was produced as a major metabolite it proved highly unstable upon purification and NMR solutions always contained diastereomers of **6**. Two diastereomers of **7** were evident in the NMR spectra, but only the major diastereomer could be fully characterised. Facile fragmentation of **7** presumably occurs by acid catalysed loss of ethylene oxide, leading to **6** as observed.

**Scheme 1:**
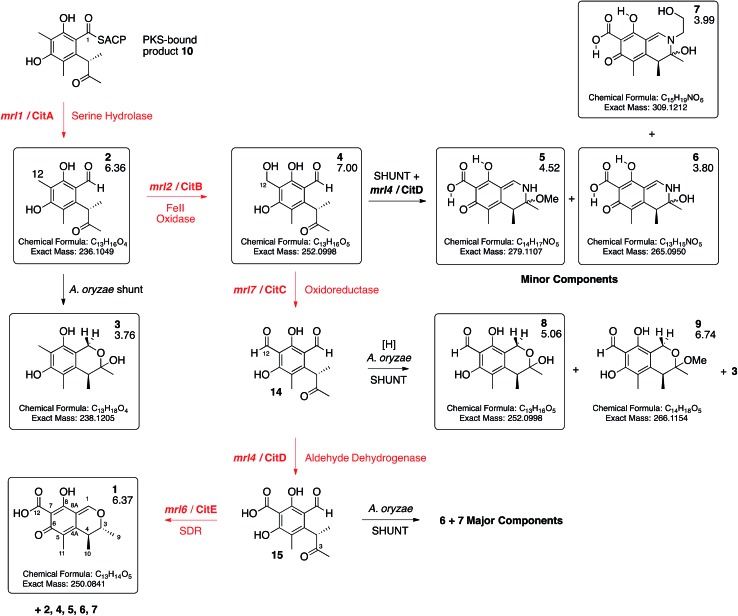
Deduced biosynthetic pathway to citrinin **1** (red steps). Boxed compounds purified and characterised. Black steps represent shunts branching from the main pathway. Figures below compound numbers are LCMS *R*_T_ values (see ESI† and Fig. 3). Unboxed compounds structures inferred.

Expression of *mrl7* and *mrl6* together (exp. 8) gave minor amounts of **2** and **4** as well as **3** and **8** (15.6 mg L^–1^). Coexpression of *mrl4* and *mrl6* (exp. 9) also generated no new compounds. Finally, coexpression of the full complement of *mrl4*, *mrl6* and *mrl7* (exp. 10) led to the production of citrinin **1** in high titre (19.1 mg L^–1^), accompanied by many of the previously identified intermediates and shunt compounds (Scheme 1).

The *mrl1* gene encodes a protein most likely to be a hydrolase or an acyl transferase. Similar hydrolases are involved in product release during brefeldin biosynthesis, for example. However **2** is an aldehyde, most likely released by reduction of the ACP-bound thiolester **10** (see Scheme 3). We previously observed such a reductive release during biosynthesis of 3-methylorcinaldehyde, the precursor of fungal tropolones, in *Acremonium strictum* encoded by the *aspks1* gene, but in this case no hydrolase is required in *A. oryzae*.^14^,^27^ The role of a hydrolytic protein during citrinin biosynthesis is thus obscure. In order to probe this question further we produced mutants of *mrl1*. The encoded protein contains a typical catalytic triad of S122, D207 and H235 and each of these residues was individually mutated to Ala. In each case coexpression of *citS* with the mutated *mrl1* genes led to a chemotype identical to that of expression of *citS* alone showing that the hydrolytic properties of the *mrl1* protein are required for the effective formation of **2** (see ESI†). In the cases of the WT protein and each mutant we also searched the LCMS chromatograms for possible product-related compounds released by hydrolysis – however no evidence supporting the existence of such compounds could be found.

Bioinformatic analysis of the CitS R-domain and R-domains of related nrPKS indicate a highly conserved cysteine (C2551, see ESI†). A homology model of the CitS R-domain constructed using the X-ray structure of the recently reported myxalamid R-domain as a template (see ESI†) showed that C2551 is located in the active site close to the catalytically important Y2392 and K2396 residues, in the vicinity of the NADPH binding pocket. In order to probe the role of this cysteine we mutated it to Ala. However we observed no differences in the function of CitS-C2551A *vs.* the WT protein when expressed either alone, or with either of the mrrl1 long or short proteins (see ESI†).

## Discussion

**Scheme 2:**
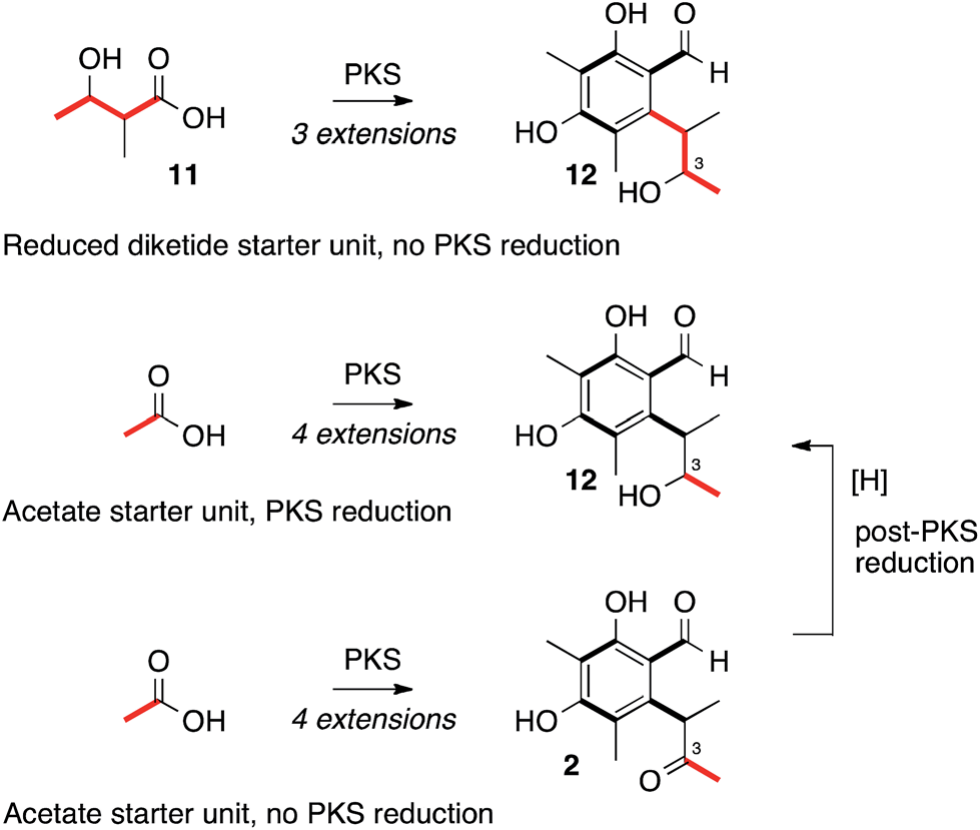
Plausible routes to alcohol oxidation level at C-3.

Heterologous expression of *citS*, the citrinin PKS, yields the trimethylated pentaketide aldehyde **2**. Experiments by Staunton and coworkers sought to determine if 2-methyl-3-hydroxy butyrate **11** could be the starter unit for biosynthesis, giving **12** as an intermediate, or whether the PKS itself reduces the C-3 carbonyl giving **12** directly (Scheme 2).^28^ Other fungal systems, such as the resorcylic acid lactones, are well known in which a reduced polyketide acts as a starter for a non-reducing PKS.^29^ Our results clearly show that the citrinin PKS produces the ketone **2** as the first enzyme free intermediate meaning that reduction of C-3 must be performed by a tailoring enzyme. This is in accord with the production of **2** by an iterative PKS which is now known to belong to the non-reducing class.^17^ Release of the aldehyde **2** is consistent with the presence of the C-terminal reductive release domain. The titre of **2** afforded in the heterologous expression experiment was surprisingly low (<1 mg L^–1^) – in our previous investigation into the related fungal tetraketide PKS methylorcinaldehyde synthase (MOS) we observed high titres from similar expression experiments (*ca.* 40 mg L^–1^).^28^

Immediately adjacent to the *citS* gene is *mrl1* which encodes a hydrolase. This gene, also known as *ctnB*, is frequently and incorrectly referred to as an oxidoreductase.^10^ The protein encoded by *mrl1* contains no recognisable protein motifs for redox cofactor binding sites and is clearly closely related to the esterase/lipase family (EC 3.1.1) of serine hydrolases.^30^

We have shown that the putative active site Ser-122, Asp-207 and His-235 residues are involved in a catalytic step during biosynthesis by systematic mutation of each to Ala. Coexpression of c*itS* and *mrl1* leads to dramatic increase in titre of **2**. Likewise KO of *mrl1* in WT *M. ruber* leads to drastic reduction, but not abolition, of citrinin **1** titres.

**Scheme 3:**
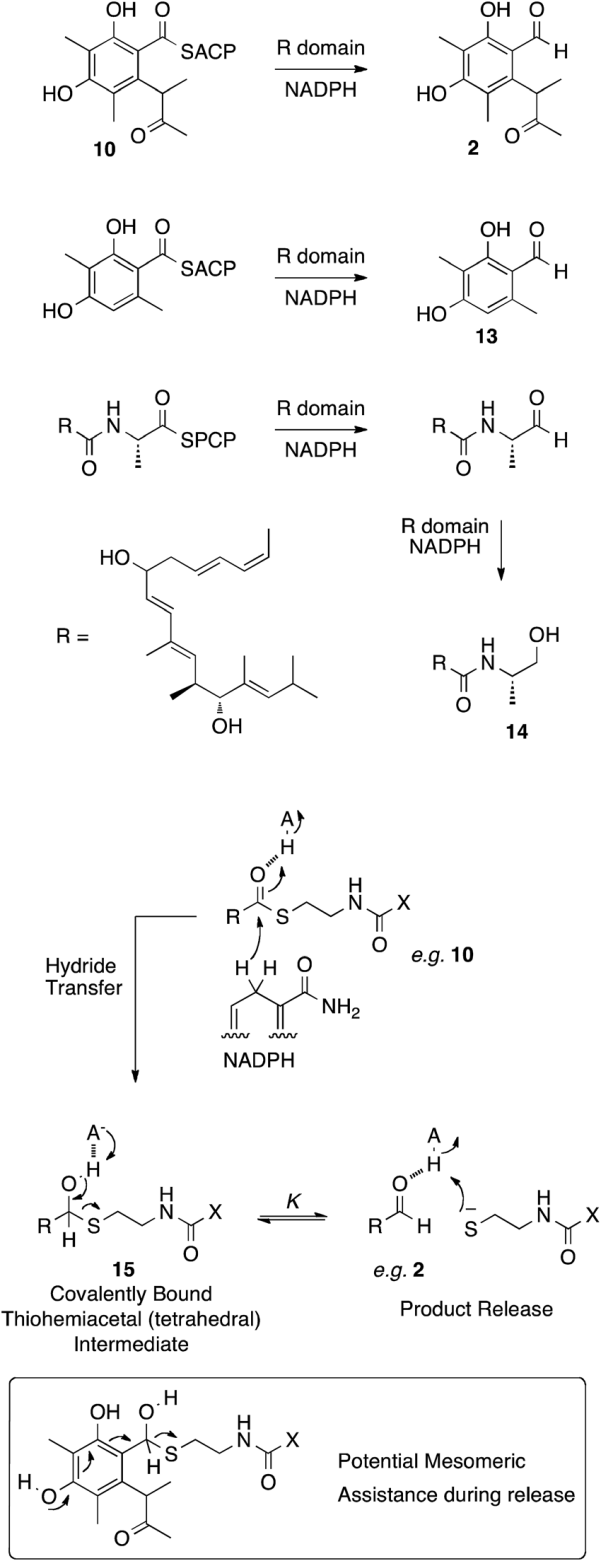
Reductive release mechanisms and a plausible mechanism. X = pantetheine-ACP.

In other PKS systems hydrolases are often involved in product release. For example an α/β hydrolase is involved in release of brefeldin intermediates,^31^ and LovG releases the completed nonaketide dihydromonacolin from the lovastatin nonaketide synthase (LNKS).^31^ However the release of the final polyketide acyl group **10**, bound to the thiol of ACP is reductive, in analogy to the reductions of many other enzyme-bound thiolesters, for example during the biosynthesis of 3-methylorcinaldehyde **13** by the PKS methylorcinaldehyde synthase, and myxalamid **14** by the mixed PKS-NRPS MxaA (Scheme 3).^32^

These reactions involve the transfer of hydride from NADPH to form a thiohemiacetal intermediate **15** which is still covalently bound to the enzyme (Scheme 3). Breakdown of the thiohemiacetal **15** then releases the aldehyde (*e.g.***2**) from the active site. In the case of **14** biosynthesis the released aldehyde is reduced a second time.

Previous studies on the thermodynamics of thiohemiacetal formation have shown that these compounds are in equilibrium with the free thiol and aldehyde. The equilibrium constant *K* for this process does not change significantly with thiol p*K*_a_, but does change with the identity of the acyl group. In general, electron-withdrawing groups stabilise the electron-rich thiohemiacetal reducing the value of *K* (so that *K* < 1 [Scheme 3], although not usually less than 0.1), favouring thiohemiacetal formation.^33^ In contrast, electron donating groups favour the separate thiol and aldehyde so that *K* > 1 (*i.e.* product release here).^34^ In the case of CitS the substrate is highly electron rich – the conjugated aromatic would be expected to contribute strongly to collapse of the tetrahedral thiohemiacetal and release of the product (Scheme 3). Thus it seems unlikley that the *mrl1* protein could be involved in product release.

**Scheme 4:**
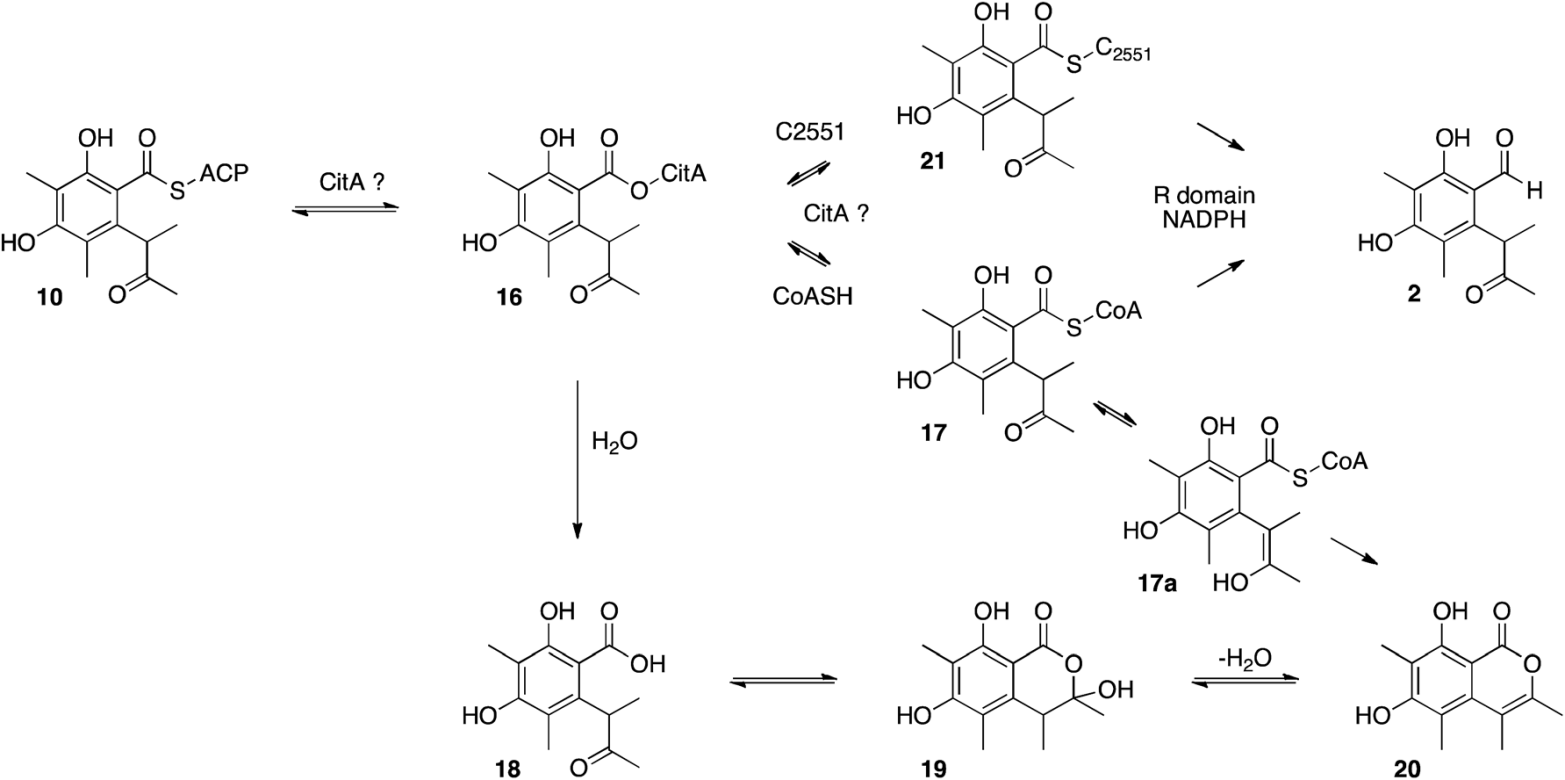
Possible roles of CitA during citrinin biosynthesis.

Other roles for the *mrl1* protein could be as an acyl transferase, possibly responsible for loading starter unit or malonyl extender unit on to the PKS. These roles are normally performed by the PKS SAT and AT domains respectively. Sequence analysis of CitS suggests that these domains are intact, with the expected conserved catalytic residues (see ESI†). Other possibilities include transfer of enzyme-bound thiolester **10** to the mrl1 protein (**16**) and then on to CoA, forming **17**, prior to reduction. Hydrolysis of **16** or **17** could lead to compounds such as **18–20**, however LCMS analysis of extracts failed to provide evidence for the presence of these species (Scheme 4).

Another possibility could be transfer of the polyketide to a conserved cysteine thiol in the R-domain active site (*e.g.***21**). Some acyl group reductases, for example aspartate semialdehyde dehydrogenase,^35^ use an active site thiol rather than a pantetheine thiol to hold the acyl group. One such conserved cysteine (C2551) was identified in the active site of the CitS R-domain, but mutational analysis showed it is not required for reductive release.

Thus a hydrolytic/transferase enzyme activity such as that seemingly catalysed by the *mrl1* hydrolase is clearly not expected in this case, or required by closely related polyketide synthases such as those which produce 3-methylorcinaldehyde **13** at high titre without hydrolytic assistance. The exact catalytic role of the *mrl1* protein therefore remains mysterious, although it is clear that it increases the productivity of the PKS. We thus rename the *mrl1* hydrolase protein as CitA as it is the earliest non-PKS step during citrinin biosynthesis. It should be noted that during the preparation of this manuscript Kwon and coworkers^36^ reported a similar phenomenon during the biosynthesis of azaphilones also in *Monascus* species, and the involvement of hydrolytic processes during aromatic polyketide biosynthesis may therefore be a more general phenomenon. We plan further *in vitro* experiments to probe the interaction of CitA with CitS in future.

**Scheme 5:**
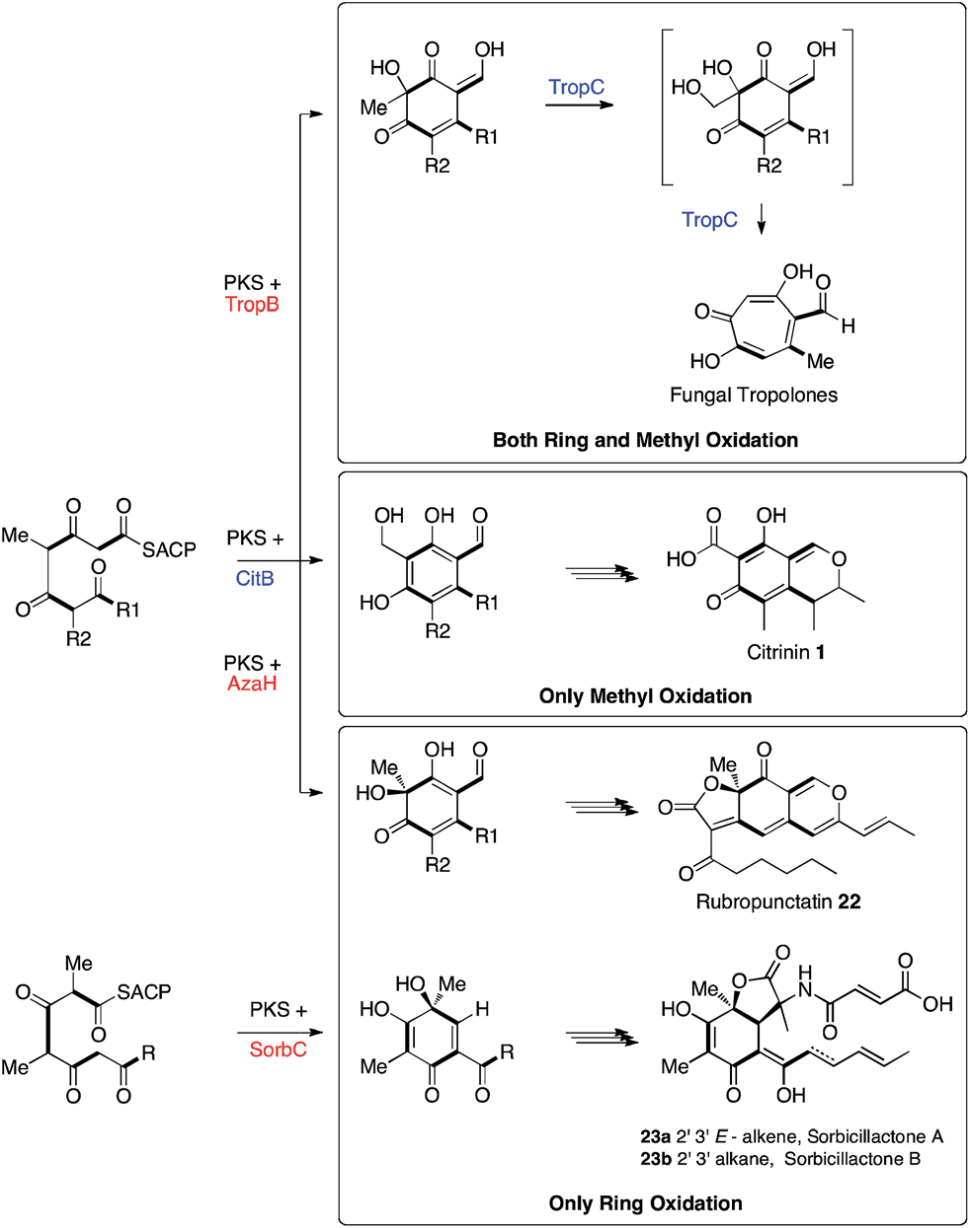
Biosynthetic relationships between the fungal tropolone, citrinin, azaphilone and sorbicillinoid pathways.

Adjacent to the *mrl1* gene in *M. ruber* is *mrl2* which encodes a non-heme iron oxidase related to TropC. TropC oxidises a methyl group during tropolone biosynthesis and also catalyses rearrangement of the dearomatised ring (Scheme 5). Here we have shown by KO and by heterologous expression that the *mrl2* encoded protein catalyses the oxidation of the C-12 methyl of **2** to an alcohol **4**, but in contrast to TropC it does this while the ring is still aromatic, and there is thus no oxidative rearrangement. This is the second step in the pathway and we thus name this protein CitB.

Tropolone biosynthesis requires oxidation of *both* the ring itself and the ring-methyl group of a polyketide aldehyde. The biosynthesis of citrinin, in which only the ring-methyl is oxidised, contrasts with the biosynthesis of azaphilones such as rubropunctatin **22**^37^ and the sorbicillinoids such as sorbicillactones **23ab**^13^ where only ring oxidation is required. Thus the three pathways are clearly differentiated by the presence or absence of these two different oxidative steps. This knowledge will be important for more accurately predicting the products of related fungal biosynthetic gene clusters.

Conversion of **4** to **1** requires two further oxidations of C-12 and a reduction of C-3. The remaining 3 genes in the cluster all encode oxidoreductases and heterologous expression of *citS*, *mrl1* and *mrl2* with *mrl4*, *mrl6* and *mrl7* leads to good production of **1** in *A. oryzae*. The data obtained here are consistent with a synthetic scheme in which **4** is first oxidised by the *mlr7* oxidoreductase (now renamed CitC) to produce the bisaldehyde **14**. In our expression experiments this compound is highly unstable and thus not observed directly, but we do observe the shunt compounds **8** and **9** which result from the expected oxidation accompanied by an adventitious *A. oryzae* shunt reduction of C-1. In cases where MeOH was used during purification the methyl acetal **9** was formed. The course of oxidation mirrors that observed during stipitatic acid biosynthesis where a ring-methyl is converted to a carboxylic acid.^19^

The fourth catalytic step is catalysed by the *mrl4* enzyme (now renamed CitD) which encodes an aldehyde dehydrogenase. This is expected to give **15**, but again we only observe the shunt metabolites **6** and **7** which have incorporated ammonia or ethanolamine respectively – again presumably because **15** is highly reactive. Once again, if MeOH is present during purification then the methylaminal **5** is observed. In this experiment **6** and **7** are produced as the major metabolites. This contrasts with experiments expressing these genes but lacking *mrl7* where the same compounds were observed in overall low titre, presumably due to low activity of an adventitious *A. oryzae* 12-oxidase.

The final transformation is reduction of C-3 by the *mrl6* enzyme (now renamed CitE). This protein appears highly selective for its substrate as its presence in any context other than a full complement of CitS + CitA–D does not result in observable new compounds. Reduction by CitE provides the chemically stable citrinin nucleus. Although this experiment afforded **1** in good titre, the conversion is not clean and several of the previously observed intermediates and shunts are also observed. This may occur because of an unnatural stoichiometry of gene expression means that the activities of the pathway enzymes are not properly balanced.

## Conclusion

Heterologous expression of the citrinin biosynthetic genes in *A. oryzae* using strong promoters has elucidated the precise steps of citrinin biosynthesis in fungi and once again demonstrated that full pathways can be reconstituted in fungi to yield metabolites in good titre. The titres obtained here (*ca.* 20–50 mg L^–1^) compare favourably with low titres reported (up to 1.5 mg L^–1^) when a simple transcript containing the *citS* + *citA*–*D* genes was transferred to *A. oryzae* from *M. purpureus*, relying on recognition of *Monascus* promoters by the *Aspergillus* host.^11^

Our results show that an unprecedented hydrolytic activity appears to be involved during polyketide construction or release by the citrinin PKS. They also show that reduction of C-3 occurs as the final step of the pathway, proving that the PKS itself does not reduce this position and that 2-methyl-3-hydroxybutyrate **11** is not used as a starter unit for subsequent 3-fold extension by the PKS as had been previously hypothesised. The results also show that *ctnB*/*citA* does not encode an oxidoreductase as widely reported, but a hydrolase which appears to assist the PKS in generating product efficiently. The next steps are oxidative-hydroxylation of the 12-methyl group of **2** to a carboxylic acid **15***via* an initial alcohol **4** and an aldehyde **14**. The presence or absence of these oxidative genes in other pathways defines tropolone, sorbicillinoid and azaphilone biosynthesis. The intermediates in this oxidative process appear to be highly reactive and, in the absence of the final reduction, are shunted by native *A. oryzae* enzymes to numerous biproducts, including azaphilones and their aminated products. However, the final ketone reduction catalysed by CitE sets up formation of the stable quinomethide structure of citrinin **1**. Thus the heterologous expression strategy also reveals the precise nature and order of the reactions which were not revealed by targeted KO in the native organism, and furthermore rules out the unprecedented rearrangements suggested previously.^16^ Overall our results have completely defined the citrinin pathway in fungi and as the biosynthesis of this compound serves as a model for that of many other fungal metabolites this knowledge will underpin future advances in fungal biosynthesis and engineering.

## Supplementary Material

Supplementary information
