# Supplementary material for: The molecular steps of citrinin biosynthesis in fungi
Source: Chem Sci. 2015 Dec 17;7(3):2119–27. doi: 10.1039/c5sc04027b (PMC5968754; doi:10.1039/c5sc04027b)
Supplement: Supplementary file 1 [file SC-007-C5SC04027B-s001.pdf]

Electronic Supplementary Material (ESI) for Chemical Science.
This journal is © The Royal Society of Chemistry 2015

The Molecular Steps of Citrinin Biosynthesis in Fungi

Yi He[1, 2] and Russell J. Cox[2, 3*]

1. College of Food Science and Technology, Huazhong Agricultural University, Wuhan 430070, Hubei Province, P. R. China.
2. Institut für Organische Chemie, Leibniz Universität Hannover, Schneiderberg 1B, 30167 Hannover, Germany.
3. School of Chemistry, University of Bristol, Cantock's Close, Bristol, BS8 1TS, UK.

* Corresponding author: russell.cox@oci.uni-hannover.de

ELECTRONIC SUPPLEMENTARY INFORMATION

1. **Annotated Genome Map of Citrinin Biosynthetic Gene Cluster in *M. ruber*** 2
2. **Comparision of citrinin biosynthesis gene clusters from *M. ruber*, *M. aurantiacus*, *M. purpureus* and *P. expansum*** 4
3. **Investigation of *citS* intron 2** 5
4. **Domain analysis of citrinin polyketide synthase (CitS)** 7
5. **LCMS chromatograms** 8
6. **LC-HRMS data for compounds 1 - 9** 35
7. **NMR data for compounds 1 - 9** 46
8. **Experimental Details** 109
9. **Previous Biosynthetic Proposals for Citrinin from the Literature** 126
10. **3D Model of CitS Reductive Release Domain** 129
11. **Presumed Mechanism of CitE / mrl6** 131
12. **References** 132

1. Gene map and annotation of the 44 kb citrinin gene cluster from *Monascus ruber* M7

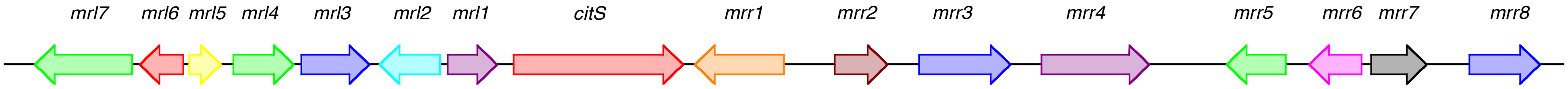

Gene	Size (bp/aa)	Plausible function	Protein with high similarity	Similarity at amino acid level (%)	Gene loci in the *M. ruber* genome
citS	7782/2593	Citrinin polyketide synthase	*M. purpureus* citrinin PKS (BAD44749.1)	99	GME2757
mrl1	786/261	Serine hydrolase, FSH1	*Botryotinia fuckeliana* putative citrinin biosynthesis oxydoreductase protein (EMR80654.1)	63	GME2756
mrl2	990/329	2-oxoglutarate (2OG) and Fe(II)-dependent oxygenase	*Botryotinia fuckeliana* putative 2og-fe oxygenase protein (XP_001559291.1)	69	GME2755
mrl3	1530/509	Transcriptional regulator (GAL4-like Zn(II)2Cys6)	*Metarhizium acridum* citrinin biosynthesis transcriptional activator CtnR (EFY89368.1)	50	GME2754
mrl4	1506/501	NAD(P)+ dependent aldehyde dehydrogenase	*Metarhizium anisopliae* dehydrogenase (EFY95582.1)	58	GME2753
mrl5	390/129	Glyoxalase-like domain	*Aspergillus terreus* predicted protein (XP_001212470.1)	81	GME2752
mrl6	879/292	Short-chain dehydrogenase	*Botryotinia fuckeliana* similar to short-chain dehydrogenase/reductase SDR (CCD44944.1)	69	GME2751
mrl7	1869/622	Glucose-methanol-choline oxidoreductase	*Botryotinia fuckeliana* GMC oxidoreductase (CCD44943.1)	61	GME2750

mrr1	1500/499	Major Facilitator Superfamily (MFS) protein	*Aspergillus oryzae* MFS multidrug transporter (XP_001822369.1)	64	GME2758
mrr2	828/275	Histidine Phosphatase	*Aspergillus niger* phosphoglycerate mutase family protein (XP_001394319.1)	74	GME2759
mrr3	1500/499	No putative conserved domains have been detected	*Aspergillus oryzae* hypothetical protein AOR_1_400154 (XP_001819450.1)	55	GME2760
mrr4	2118/705	WD40 protein	*Aspergillus flavus* WD repeat protein (XP_002372745.1)	59	GME2761
mrr5	666/221	Carbonic anhydrases	*Aspergillus oryzae* putative carbonic anhydrase involved in protection against oxidative damage (EIT80876.1)	73	GME2762
mrr6	570/189	No putative conserved domains have been detected	*Aspergillus terreus* conserved hypothetical protein (XP_001216999.1)	62	GME2763
mrr7	999/332	Enoyl-(Acyl carrier protein) reductase	*Neosartorya fischeri* oxidoreductase, short-chain dehydrogenase/reductase family (XP_001259935.1)	75	GME2764
mrr8	1974/657	AMP-binding enzyme	*Neosartorya fischeri* long-chain fatty acid transporter, putative (XP_001257846.1)	70	GME2765

2. Comparision of citrinin biosynthesis gene clusters from *M. ruber*, *M. aurantiacus*, *M. purpureus* and *Penicillium expansum*

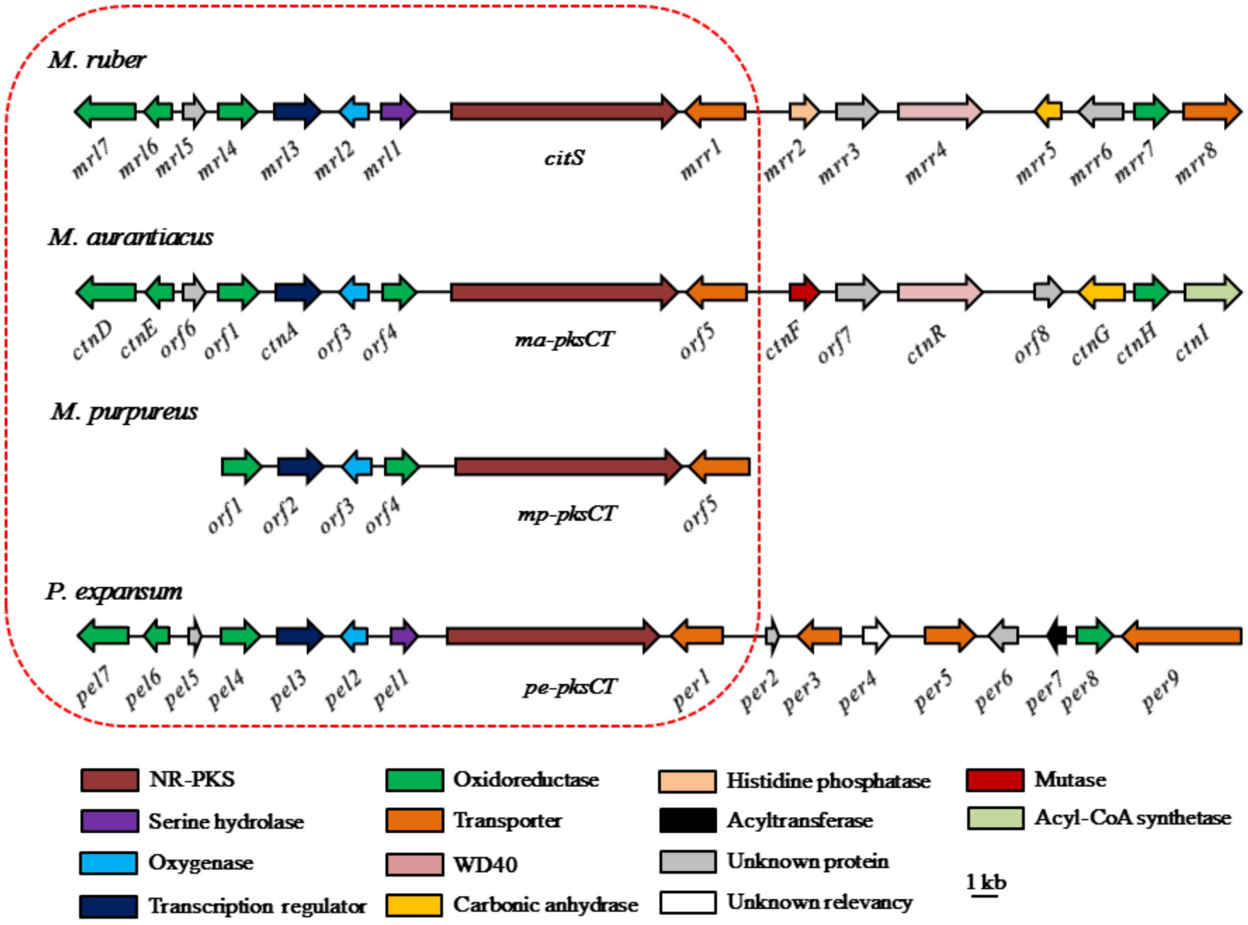

The genes inclosed by the red dotted line between *Monascus* spp. and *P. expansum* are highly homologous. The nucleotide identity of these marked genes reaches 99% among these three different *Monascus* strains, and their nucleotide identity with *P. expansum* reaches 81%. Note that the *orf4* gene in *M. aurantiacus* and *M. purpureus* was annotated as oxidoreductase according to the published papers, actually it is a homolog of *mrl1*.

Table S1 Description of homologous genes in citrinin gene cluster among *M. ruber*, *M. aurantiacus*, *M. purpureus* and *P. expansum*

**Gene Name in *M. ruber***	**Putative Function**	**Homologous Gene in *P. expansum***	**Protein Identity between *Monascus* spp. and *P. expansum* (%)**	**Homologous Gene in *M. aurantiacus***	**Homologous Gene in *M. purpureus***
mrl7	Oxydoreductase	*pel7*	83	*ctnD*	-
mrl6	Dehydrogenase	*pel6*	89	*ctnE*	-
mrl5	Glyoxylase-like domain	*pel5*	87	*orf6*	-
mrl4	Dehydrogenase	*pel4*	90	*orf1*	*orf1*
mrl3	Zn_2Cys_6 regulator	*pel3*	84	*ctnA*	*orf2*
mrl2	Fe(II)-dependent oxygenase	*pel2*	89	*orf3*	*orf3*
mrl1	Serine hydrolase	*pel1*	88	*orf4*	*orf4*
citS	Non-reducing PKS	*pe-pksCT*	85	*ma-pksCT*	*mp-pksCT*
mrr1	Transporter	*per1*	88	*orf5*	*orf5*
mrr2	Histidine phosphatase	-	-	*ctnF*	-
mrr3	Unknown protein	-	-	*orf7*	-
mrr4	WD40 protein	-	-	*ctnR*	-
mrr5	Carbonic anhydrase	-	-	*orf8*	-
mrr6	Unknown protein	-	-	*ctnG*	-
mrr7	Reductase	-	-	*ctnH*	-
mrr8	Transporter	-	-	*ctnI*	-

3. Investigation of *citS* intron 2

The spores of *M. ruber* M7 were inoculated into 100 mL PDB medium in 500 mL flask, 28 °C, 160 rpm, cultured for 3 days. The mycelium were collected and then washed with distilled water and used for genomic DNA and RNA preparation. Genomic DNA was isolated with GenElute Plant Genomic DNA Miniprep Kit (Sigma) and then used as template to amplify partial *citS* gene (cover intron 2) with primers pks-4-F/ pks-4-R. PCR products were purified and then sequenced. RNA were isolated with ZR Fungal/Bacterial RNA MiniPrep (Zymo Research) and then digested with Dnase I to remove the residual genomic DNA. The obtained RNA was used as template to do PCR with primers pks-4-F/ pks-4-R to demonstrate no genomic DNA contamination (no PCR band obtained), then was transcribed into cDNA with a RevertAid Premiun Reverse Transcriptase Kit (Thermo Scientific). The transcribed cDNA was used as template to amplify partial *citS* gene (cover intron 2) with primers pks-4-F/ pks-4-R. PCR products were purified and then sequenced.

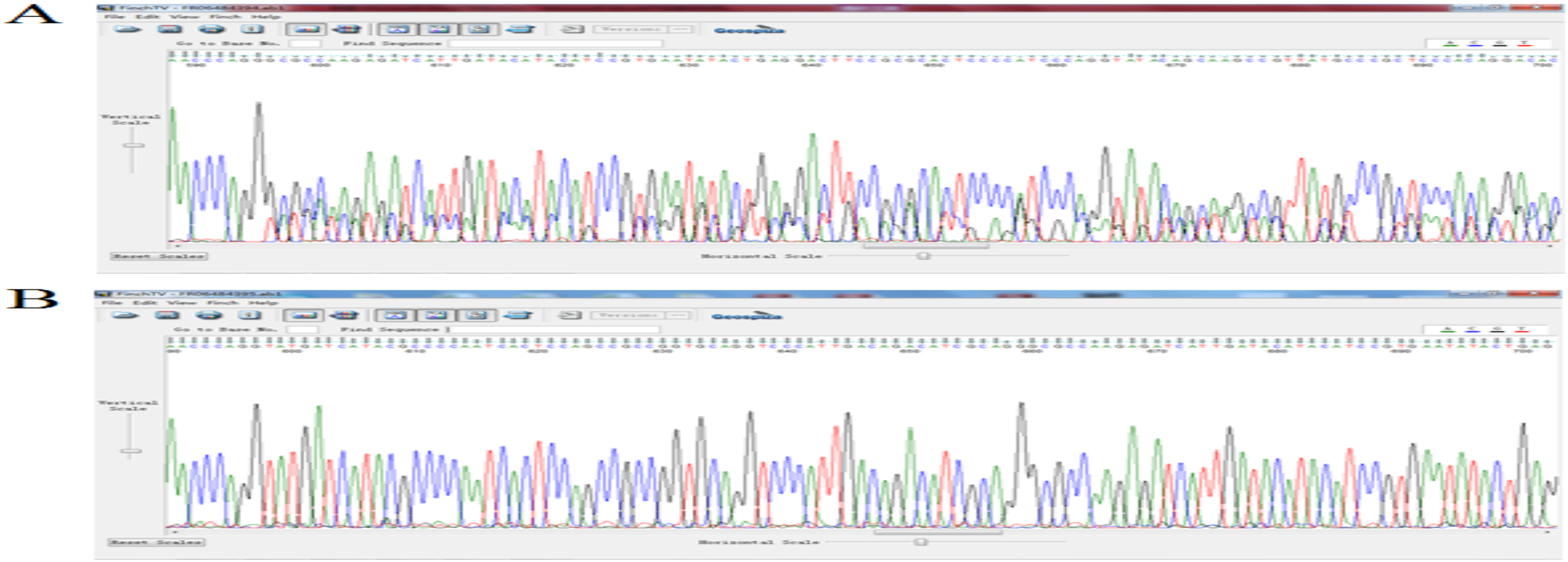

Figure S3.1 A: Partial sequencing results of mRNA of *citS* from *M. ruber M7* (start from the 6541th base of *citS*; a obvious mixture of two different sequences appears form the 6549th of the *citS* which is also the first nucleotide of intron 2); B: Partial sequencing results of genomic DNA of *citS* from *M. ruber M7* (start from the 6541th base of *citS*)

4. Domain analysis of citrinin polyketide synthase (CitS).

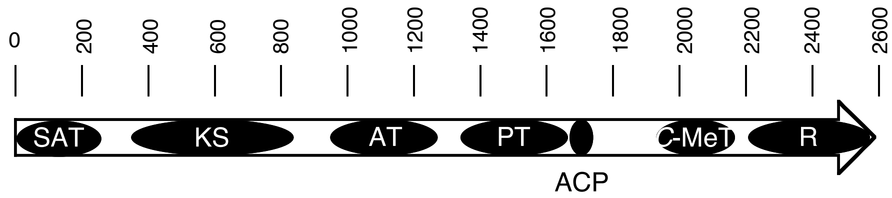

CitS 2593 Amino Acids

Domain	Predicted Boundaries	Conserved Active Site Motif
SAT	$A_{14} – V_{266}$	$G_{137}X\underline{C}XG_{141}$ – active site cysteine
KS	$D_{391} – Q_{806}$	$T_{553}A\underline{C}SSS_{558}$ – active site cysteine
AT	$F_{915} – T_{1226}$	$G_{998}H\underline{S}XG_{1002}$ – active site serine
PT	$V_{1308} – V_{1649}$	$H_{1326}X_9(P/T)_{1336}$ / $\underline{D}_{1508}X_3(Q/H)X_6N_{1519}$ – active site residues
ACP	$M_{1670} – L_{1730}$	$G_{1695}XD\underline{S}L_{1699}$ – phosphopantetheine attachment site
C-MeT	$F_{1927} – G_{2138}$	$G_{1992}XGXG_{1996}$ – S-adenosylmethionine binding
R	$T_{2198} – V_{2523}$	$G_{2226}XXGXXG_{2232}$ Rossmann fold [NAD(P) binding]<br>$Y_{2392}XXXK_{2396}$ – active site residues

5. LCMS chromatograms

exp. 0 Organic extract of *A. oryzae* NSAR 1+ pTYGS·arg

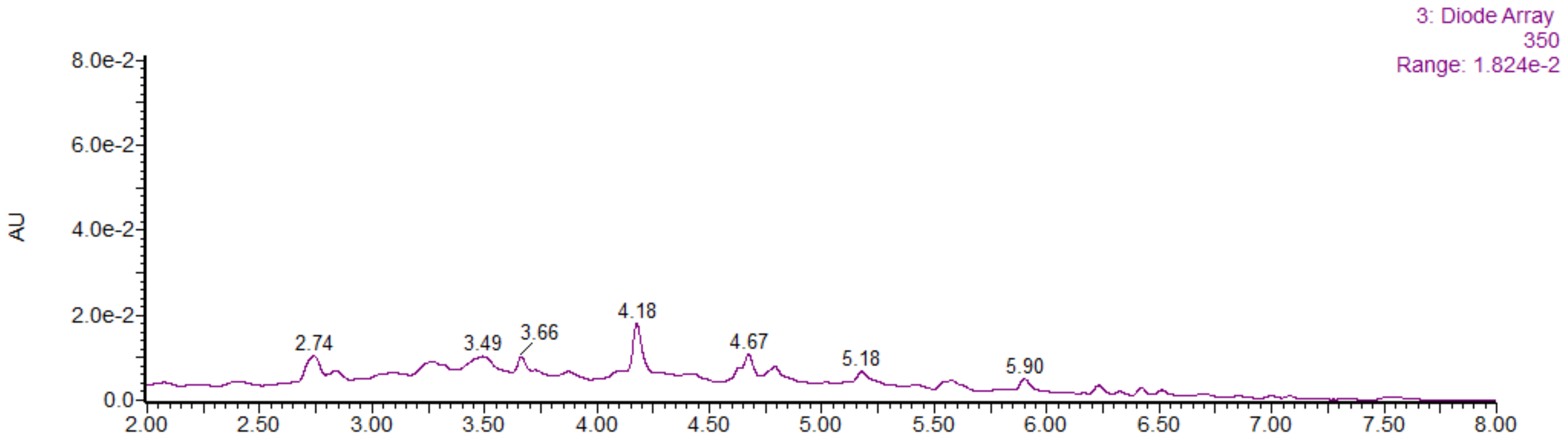

exp. 1 Organic extract of *A. oryzae* NSAR 1+ *citS*

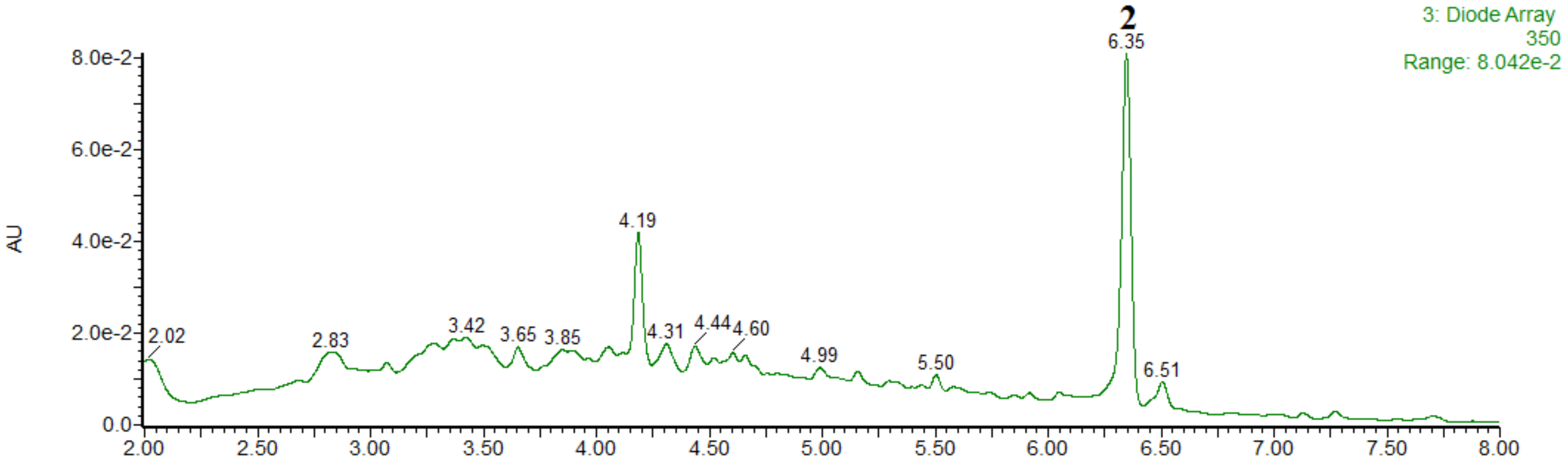

exp. 2 Organic extract of *A. oryzae* NSAR 1+ *citS* + *mrl1*·long

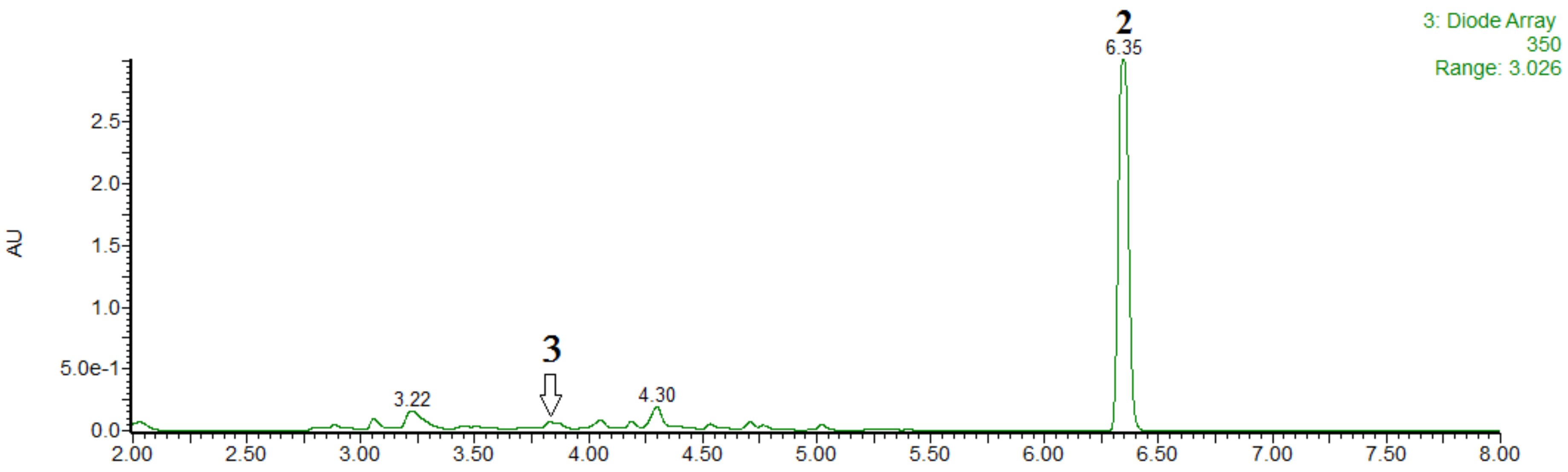

exp. 2´ Organic extract of *A. oryzae* NSAR 1+ *citS* + *mrl1*

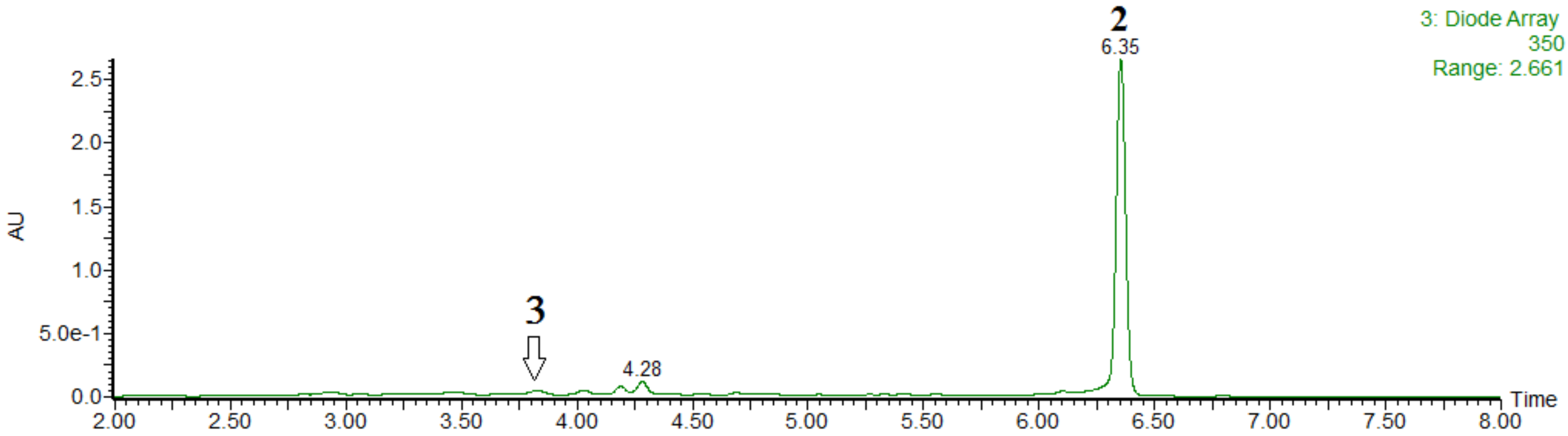

exp. 3   Organic extract of *A. oryzae* NSAR 1+ *citS* + *mrl1* + *mrl2*

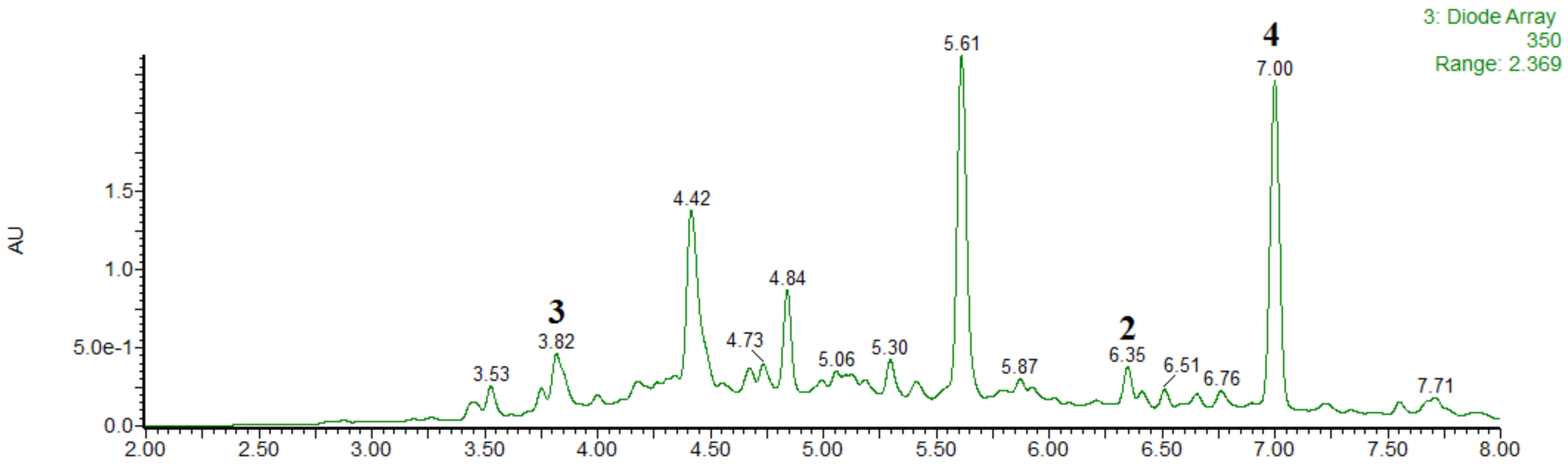

exp. 4 Organic extract of *A. oryzae* NSAR 1+ *citS* + *mrl1* + *mrl2* + *mrl4*

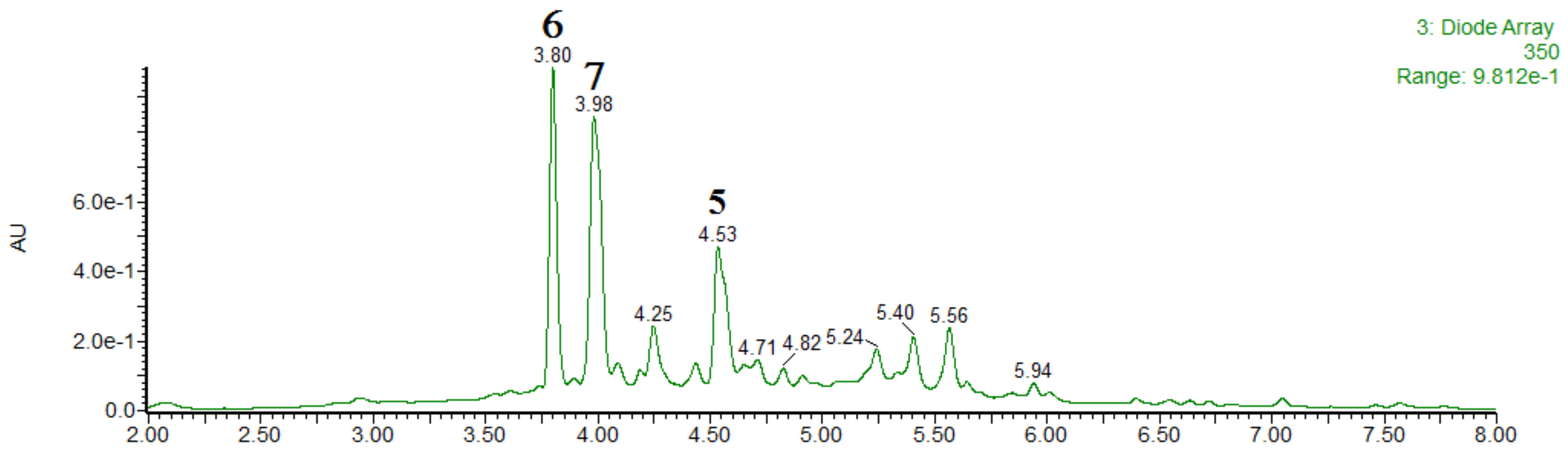

exp. 5 Organic extract of *A. oryzae* NSAR 1+ *citS* + *mrl1* + *mrl2* + *mrl7*

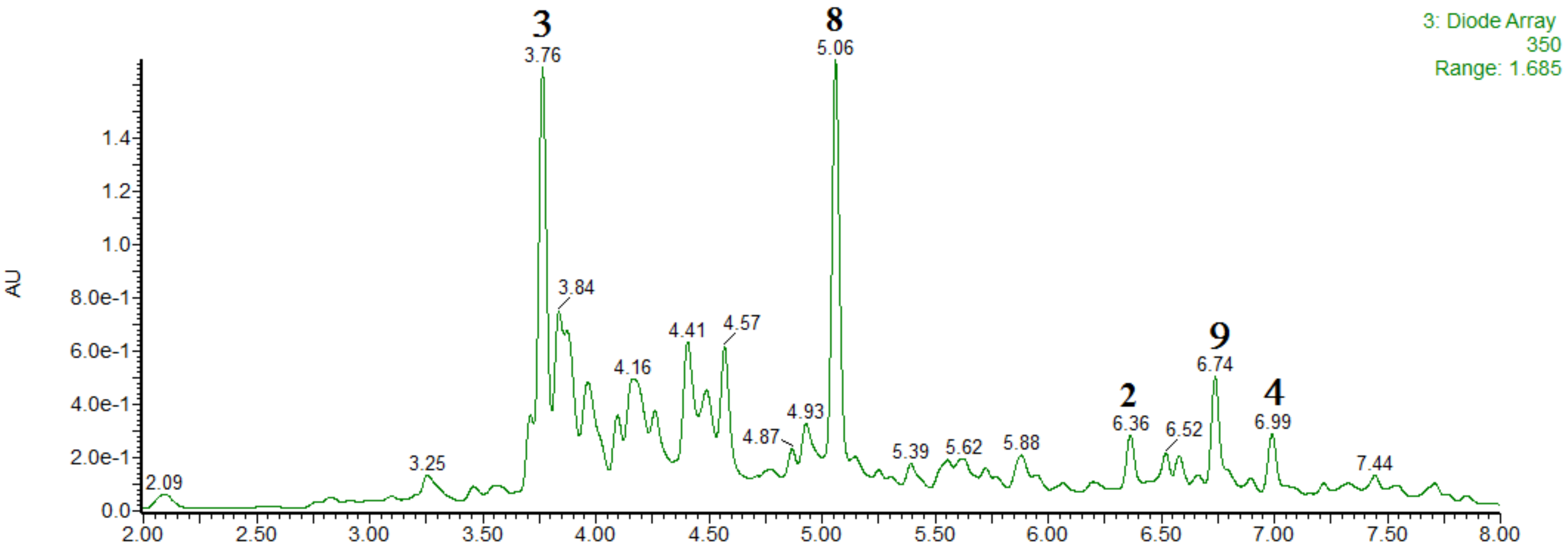

exp. 6 Organic extract of *A. oryzae* NSAR 1+ *citS* + *mrl1* + *mrl2* + *mrl6*

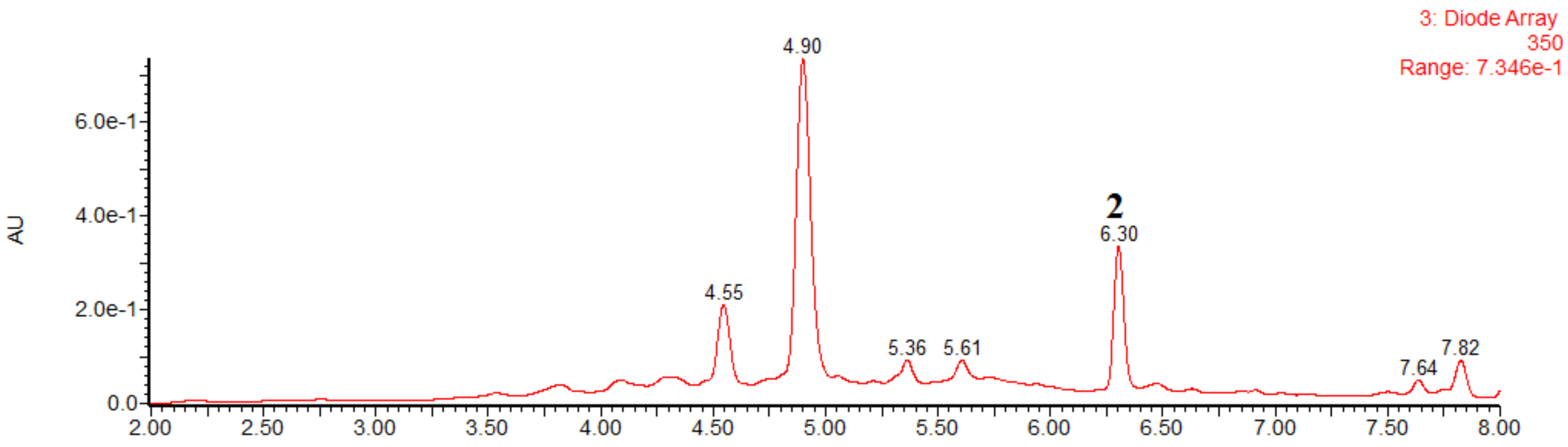

exp. 7 Organic extract of *A. oryzae* NSAR 1+ *citS* + *mrl1* + *mrl2* + *mrl4* + *mrl7*

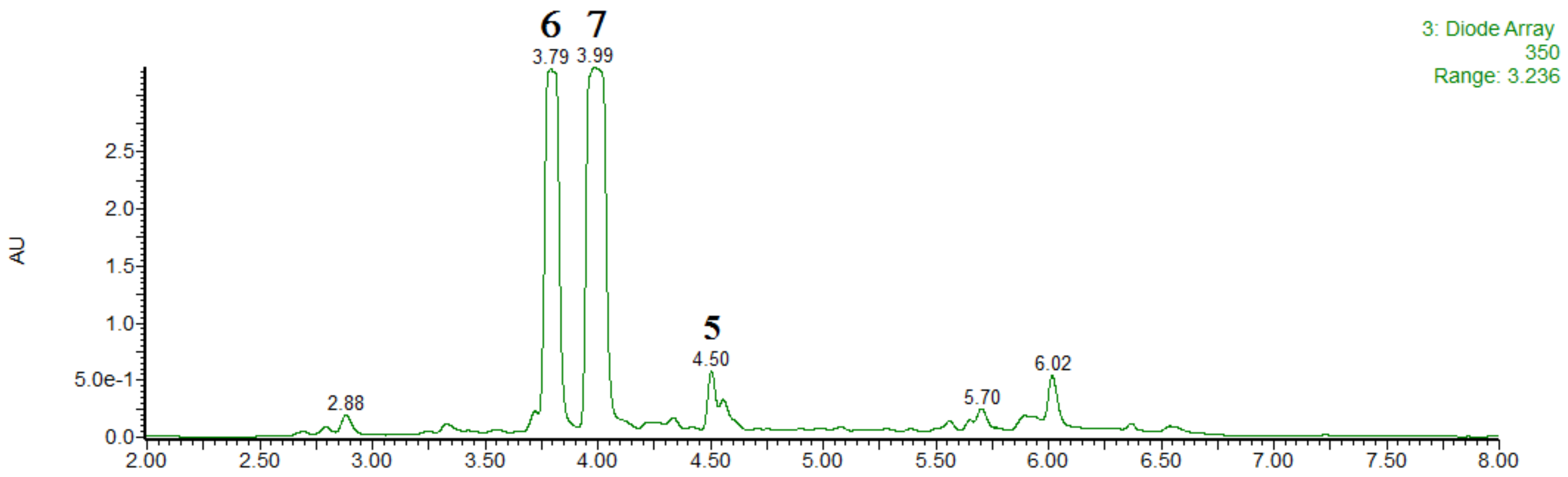

exp. 8 Organic extract of *A. oryzae* NSAR 1+ *citS* + *mrl1* + *mrl2* + *mrl6* + *mrl7*

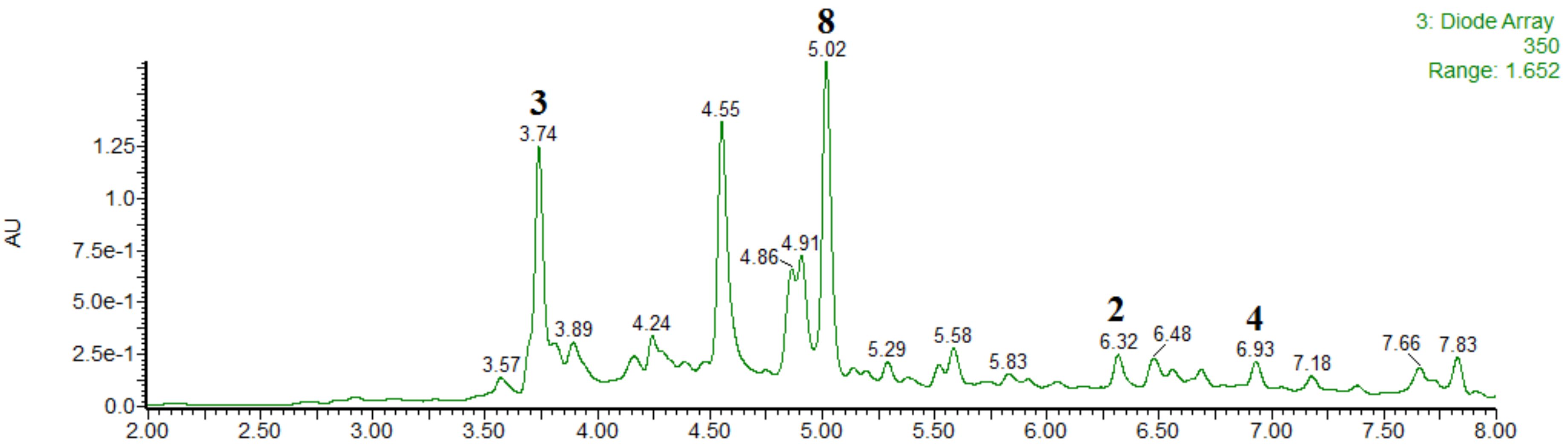

exp. 9 Organic extract of *A. oryzae* NSAR 1+ *citS* + *mrl1* + *mrl2* + *mrl4* + *mrl6*

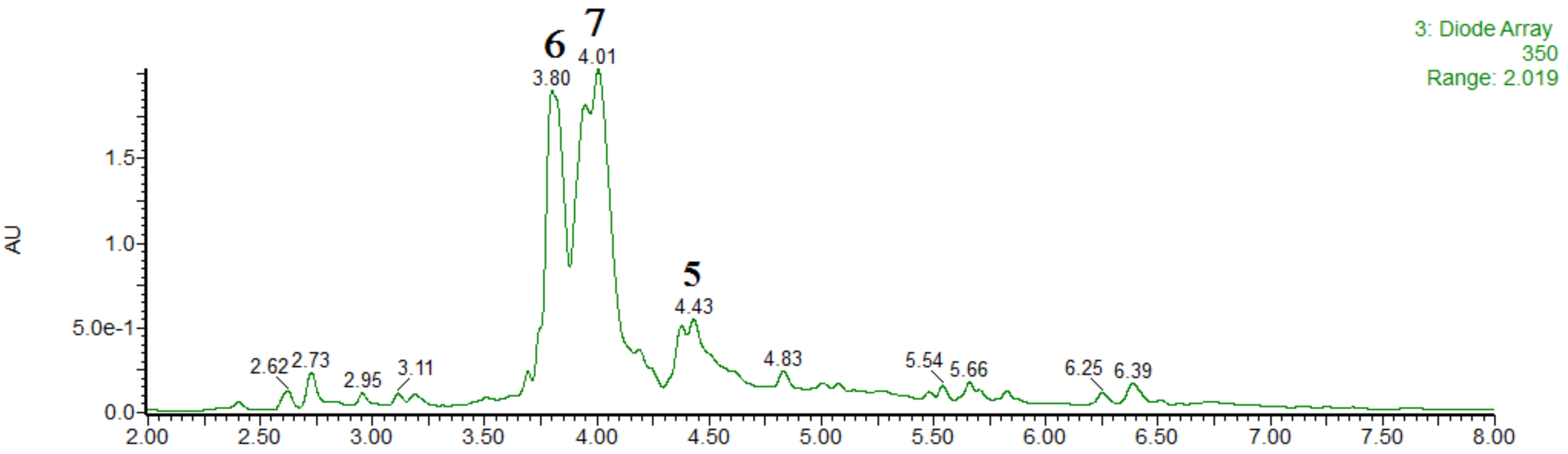

exp. 10 Organic extract of *A. oryzae* NSAR 1+ *citS* + *mrl1* + *mrl2* + *mrl4* + *mrl6* + *mrl7*

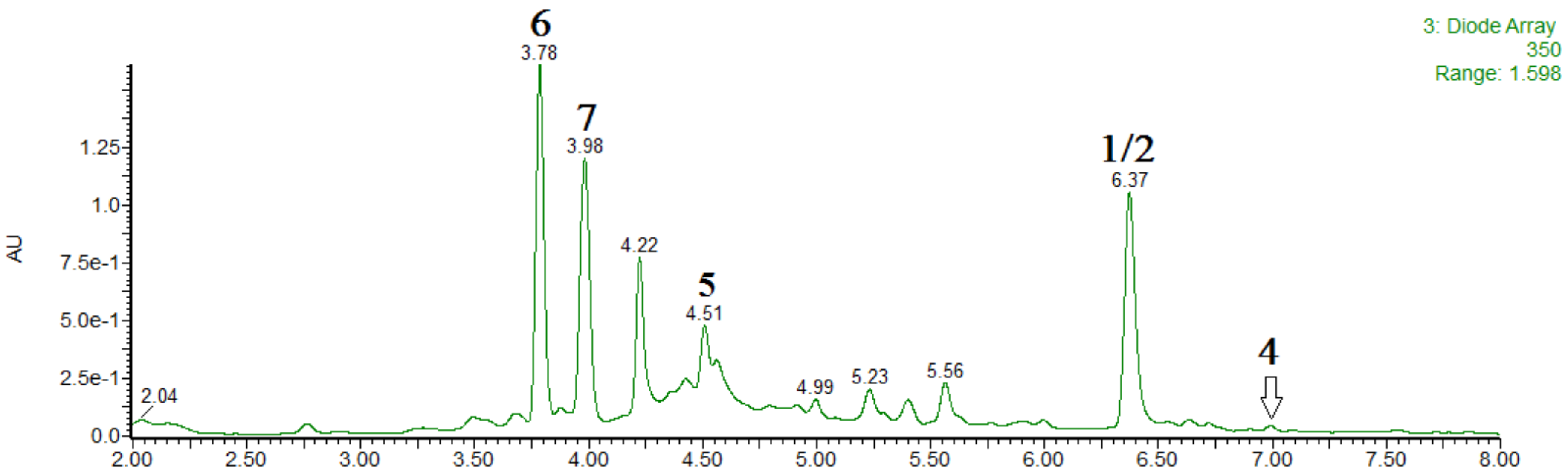

exp. 11 Organic extract of *M. ruber ΔcitS*

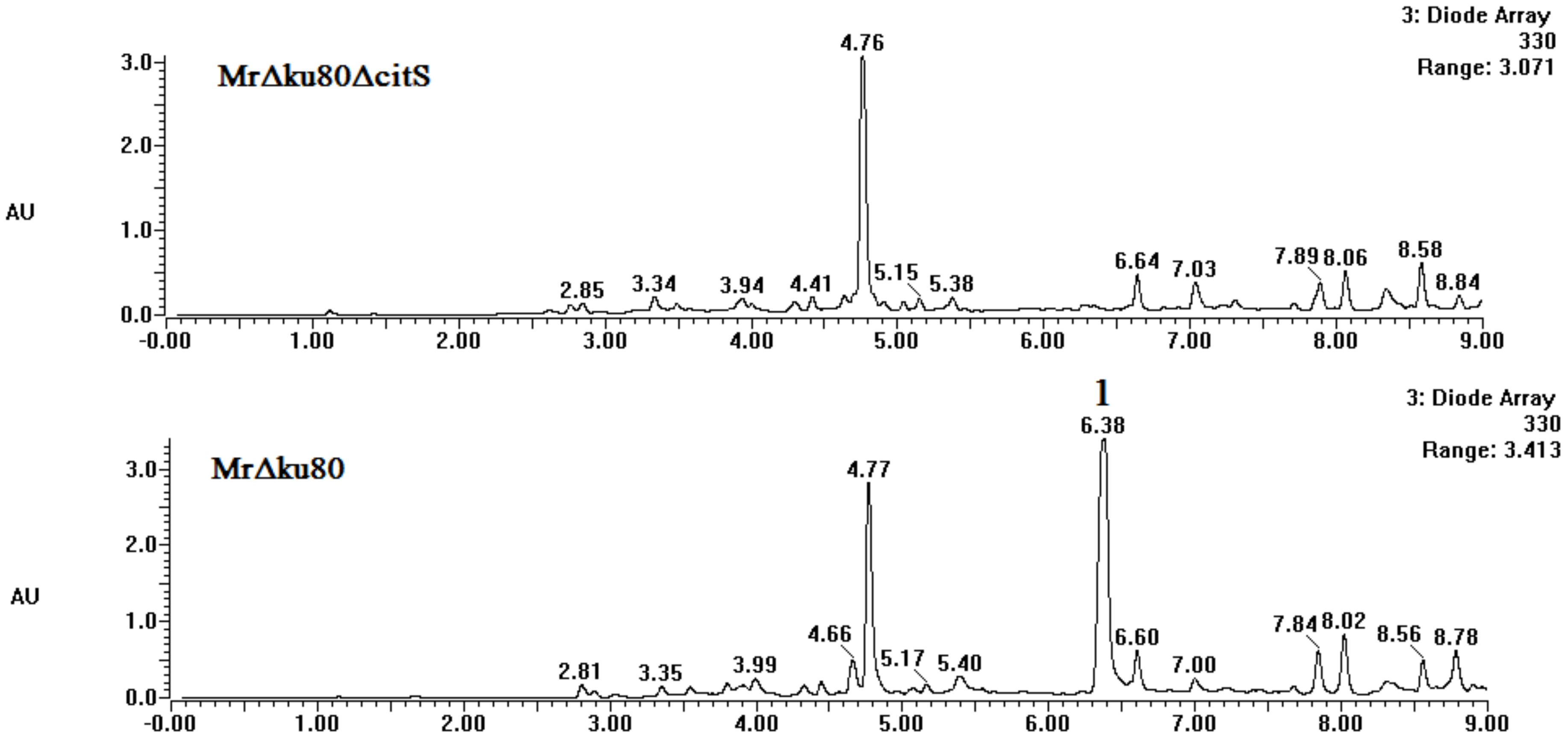

exp. 12 Organic extract of *M. ruber Δmrl1*

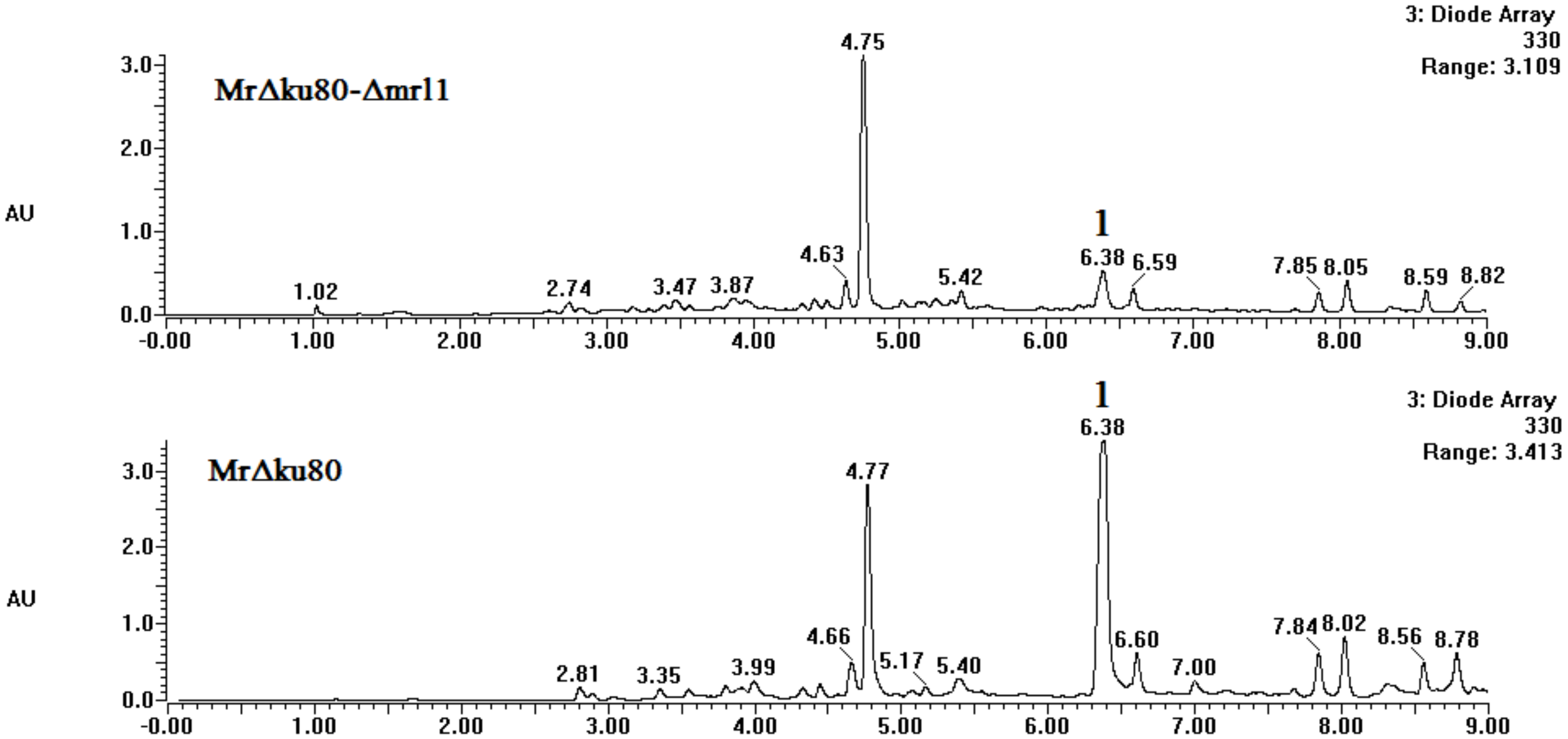

exp. 13 Organic extract of *M. ruber Δmrl2*

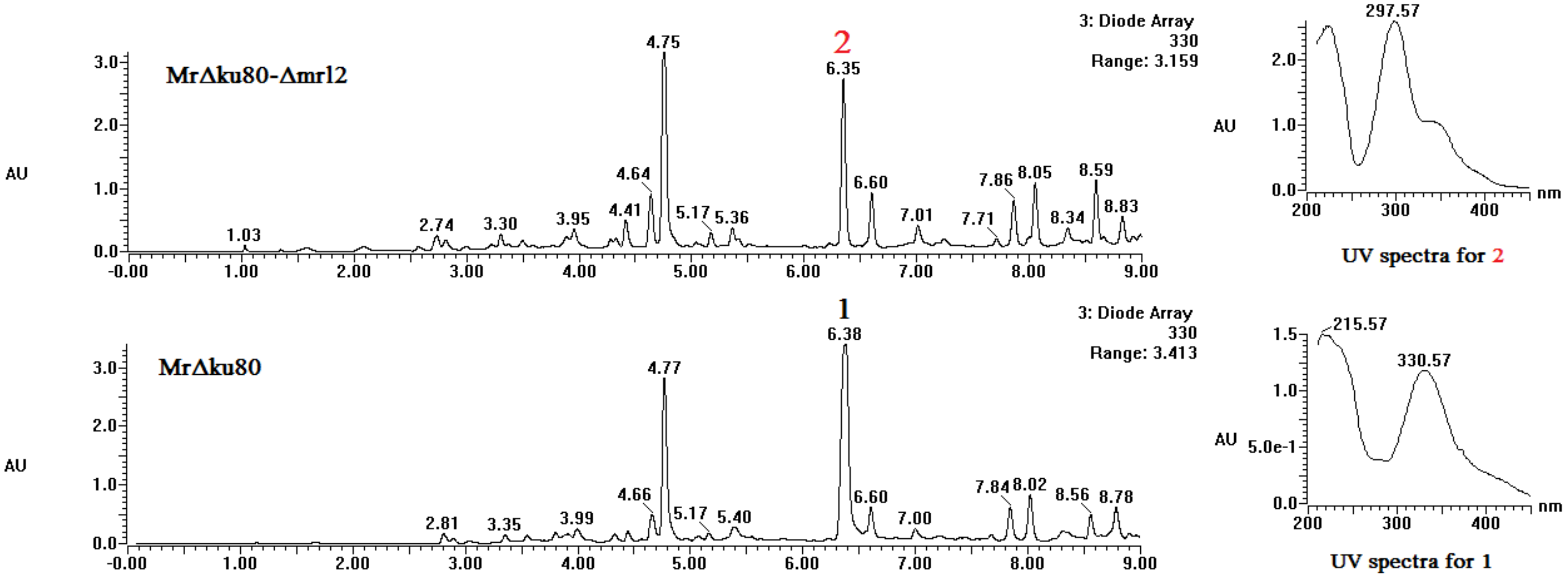

exp. 14 Organic extract of *M. ruber Δmrl4*

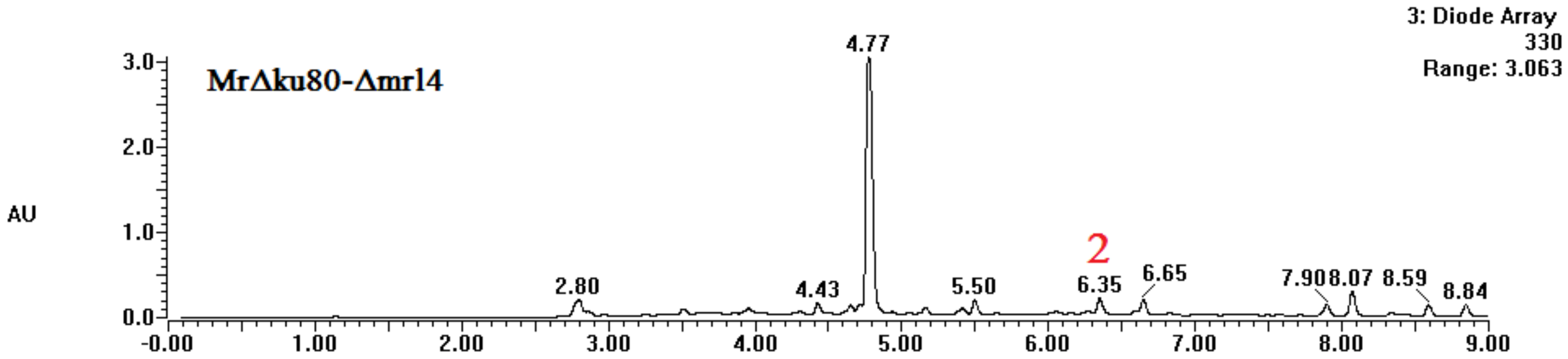

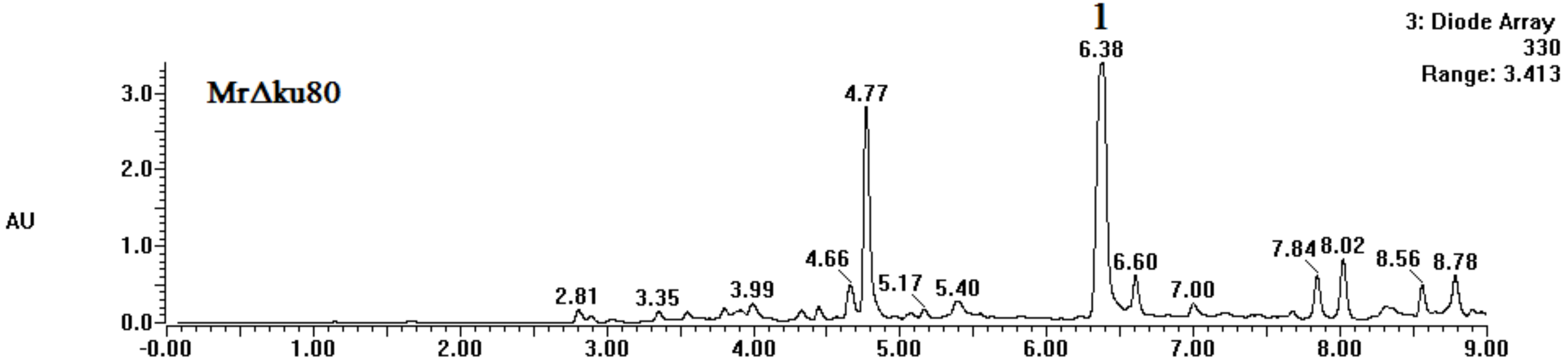

exp. 15 Organic extract of *M. ruber Δmrl6*

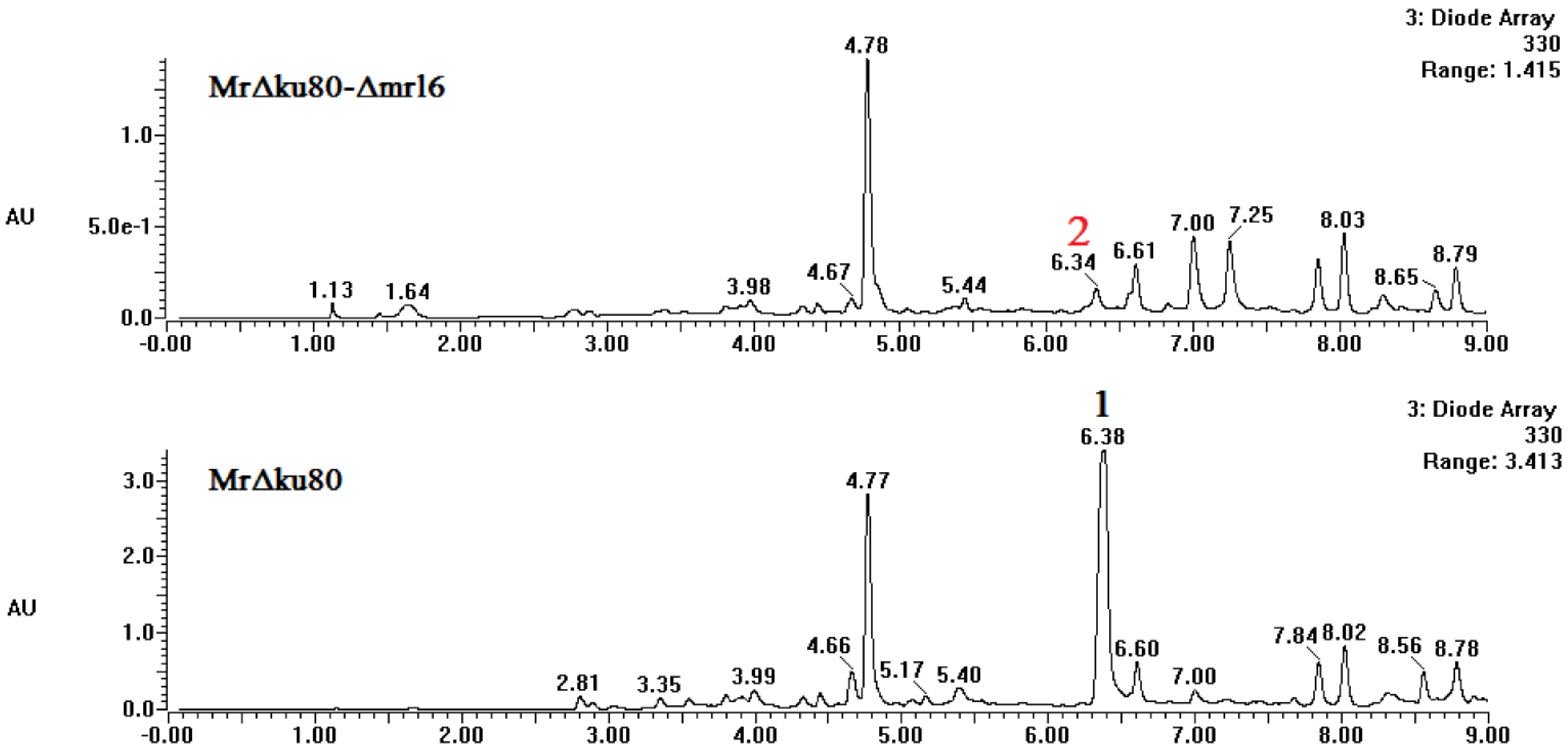

exp. 16 Organic extract of *M. ruber Δmrl7*

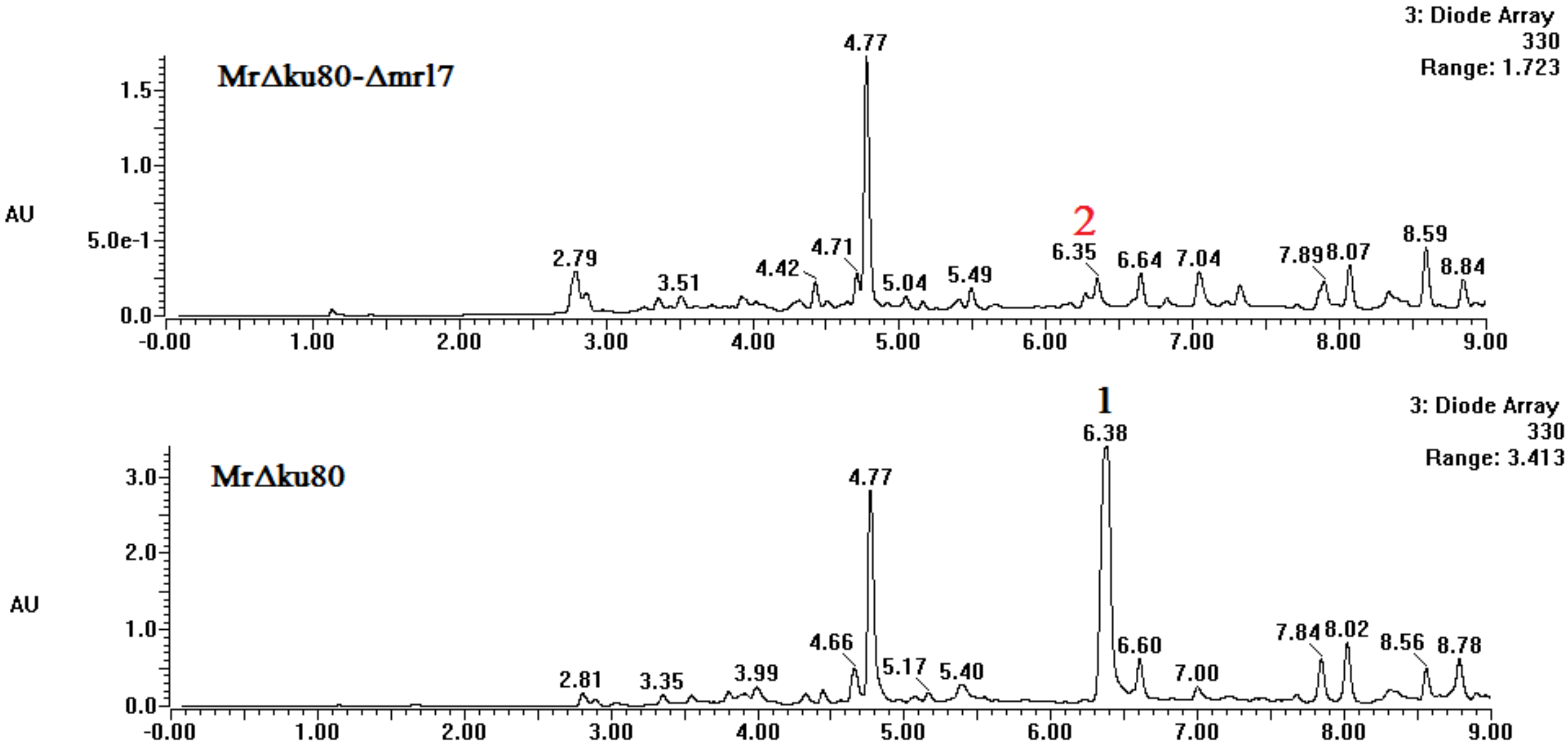

exp. 17 Organic extract of *A. oryzae* M-2-3 + pTYGS·arg·*citS*·Δ62bp

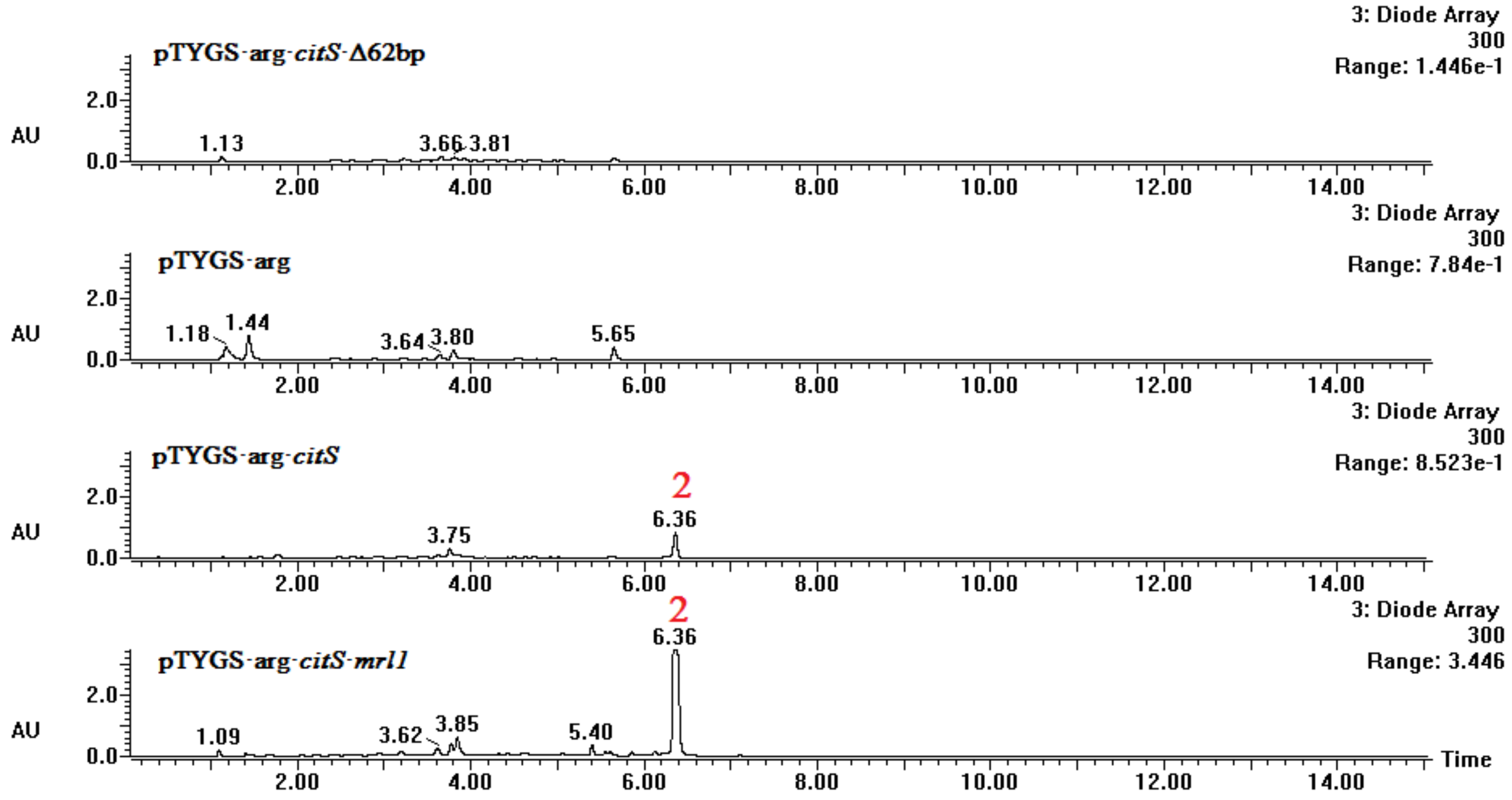

exp. 18 Organic extract of *A. oryzae* M-2-3 + pTYGS·arg·*citS*·Δ62bp·*mrl1*

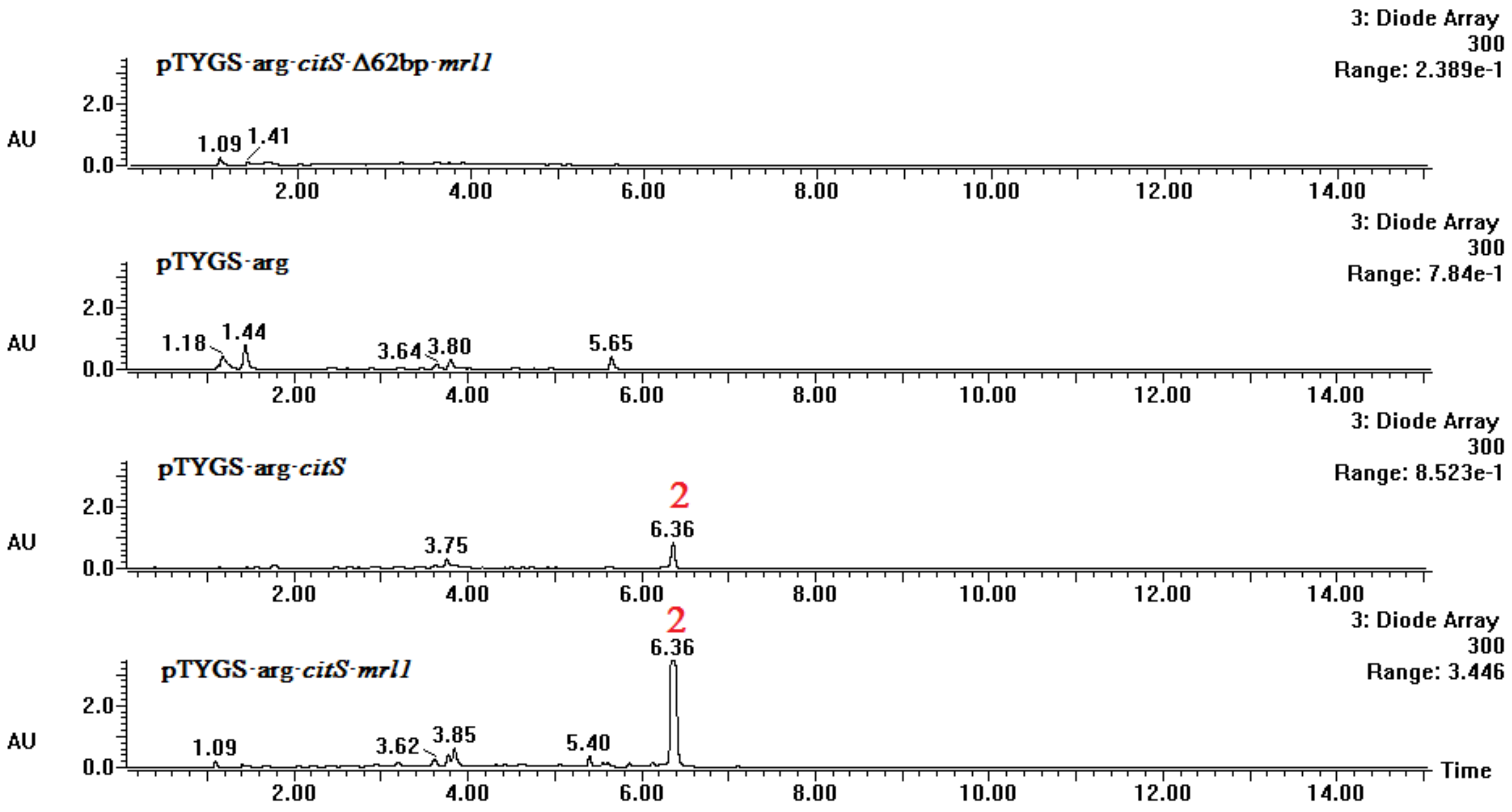

exp.19 Organic extract of *A. oryzae* M-2-3 + pTYGS·arg·*citS*·Δ60bp

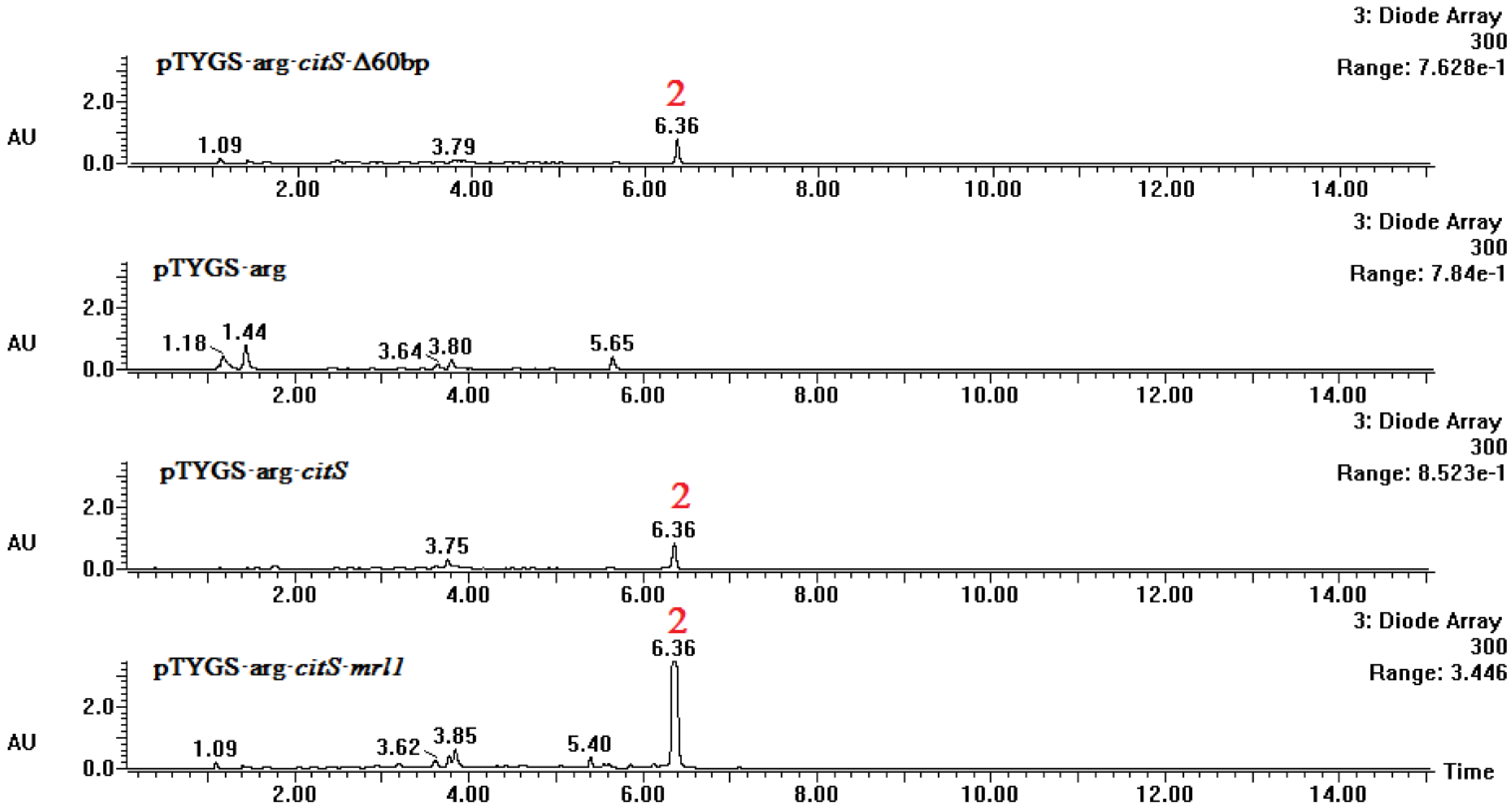

exp. 20 Organic extract of *A. oryzae* M-2-3 + pTYGS·arg·*citS*·Δ60bp·*mrl1*

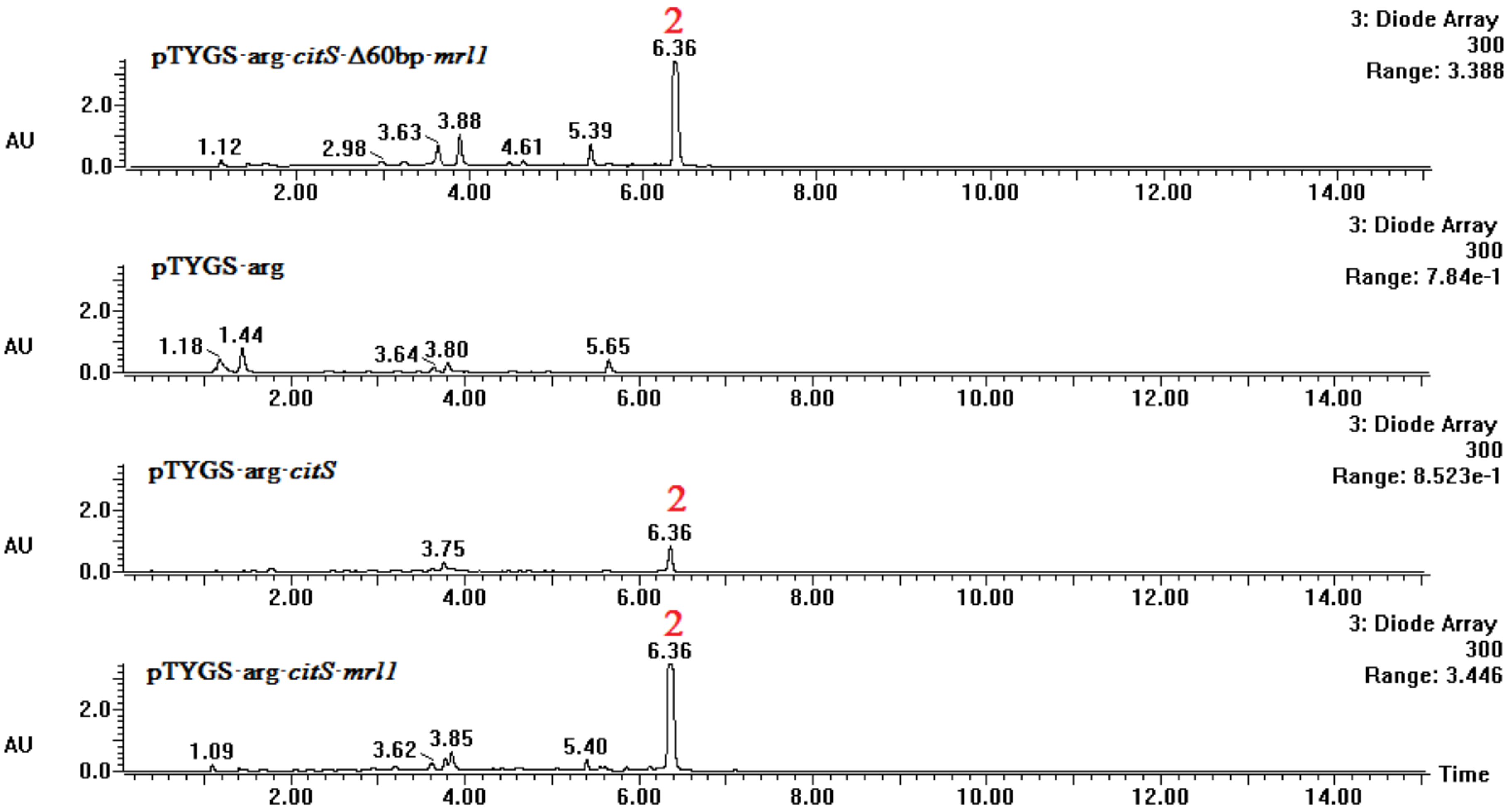

exp. 21 Organic extract of *A. oryzae* M-2-3 + pTYGS·arg·*citS*·C_{2551}A

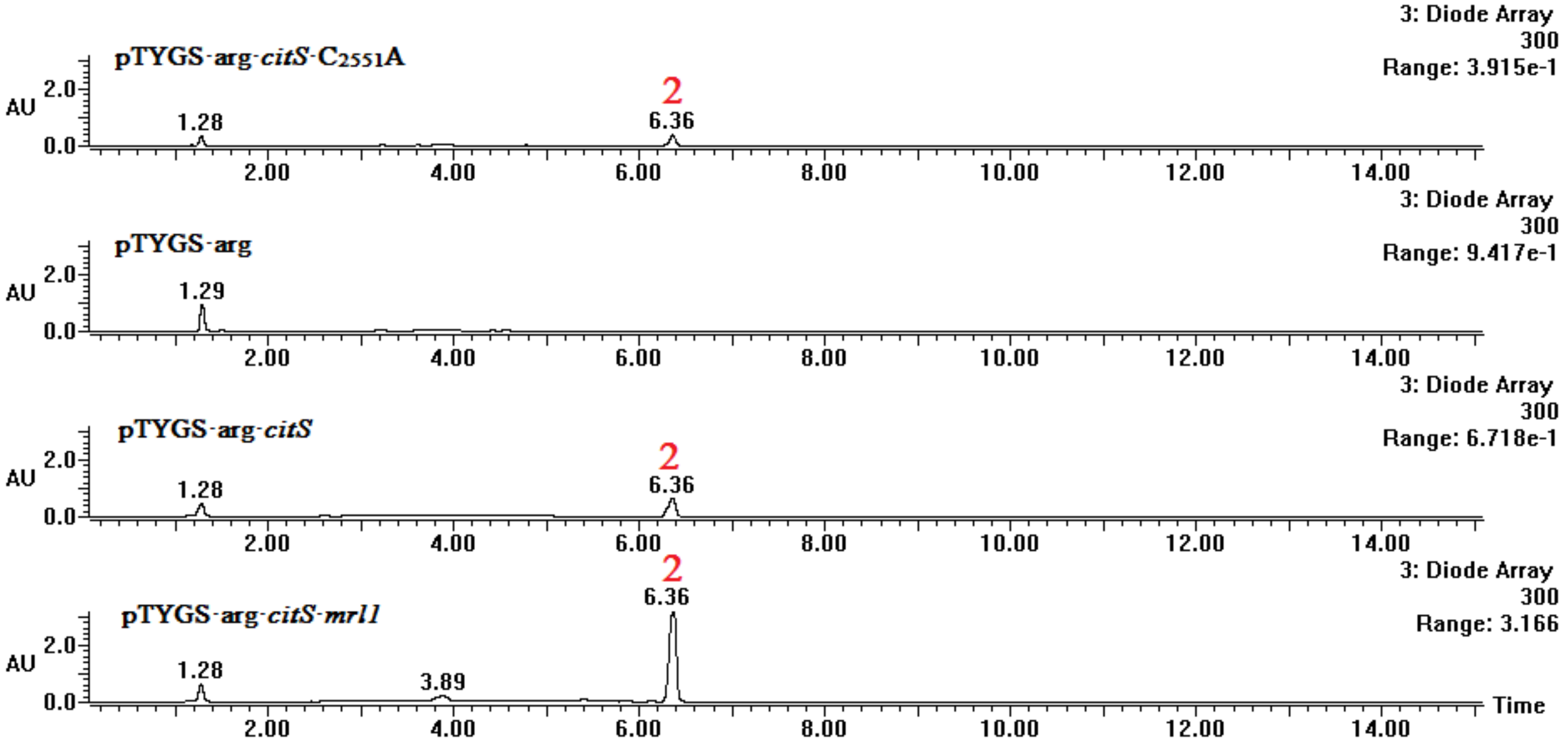

exp. 22 Organic extract of *A. oryzae* M-2-3 + pTYGS·arg·*citS*·C_{2551}A·*mrl1*

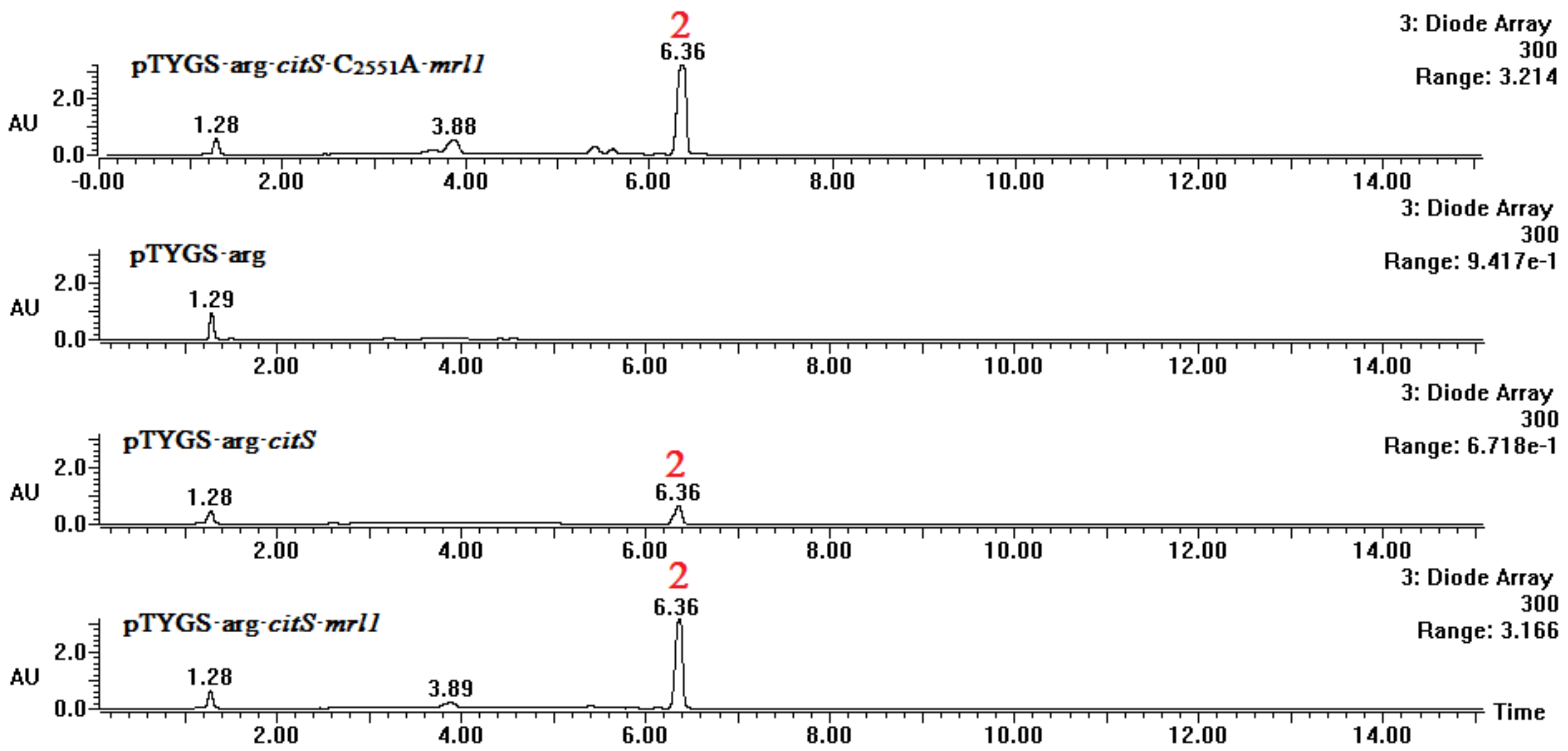

exp. 23 Organic extract of *A. oryzae* M-2-3 + pTYGS·arg·*citS*·*mrl1*·S_{122}A

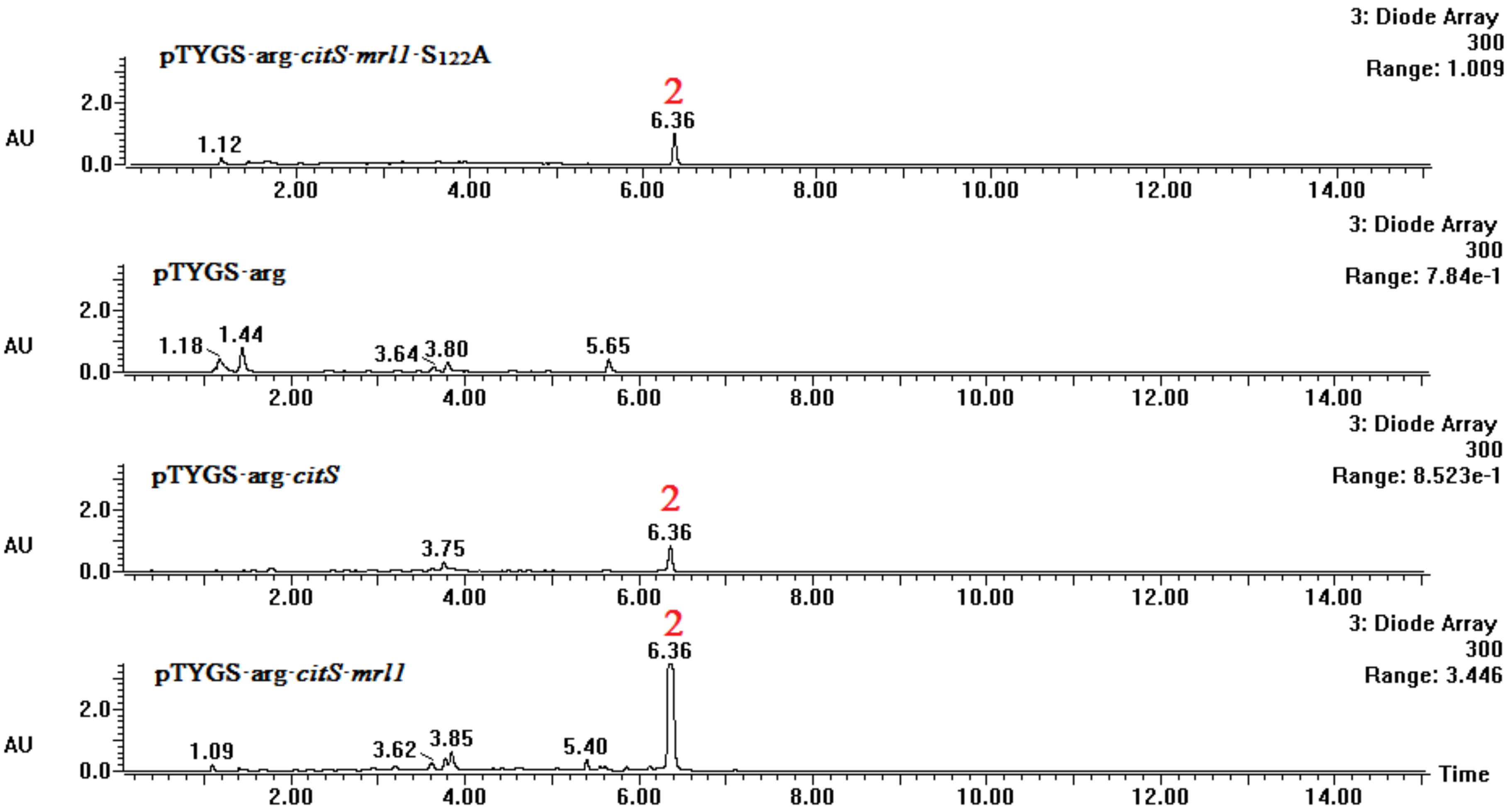

exp. 24 Organic extract of *A. oryzae* M-2-3 + pTYGS·arg·*citS*·*mrl1*·D$_{207}$A

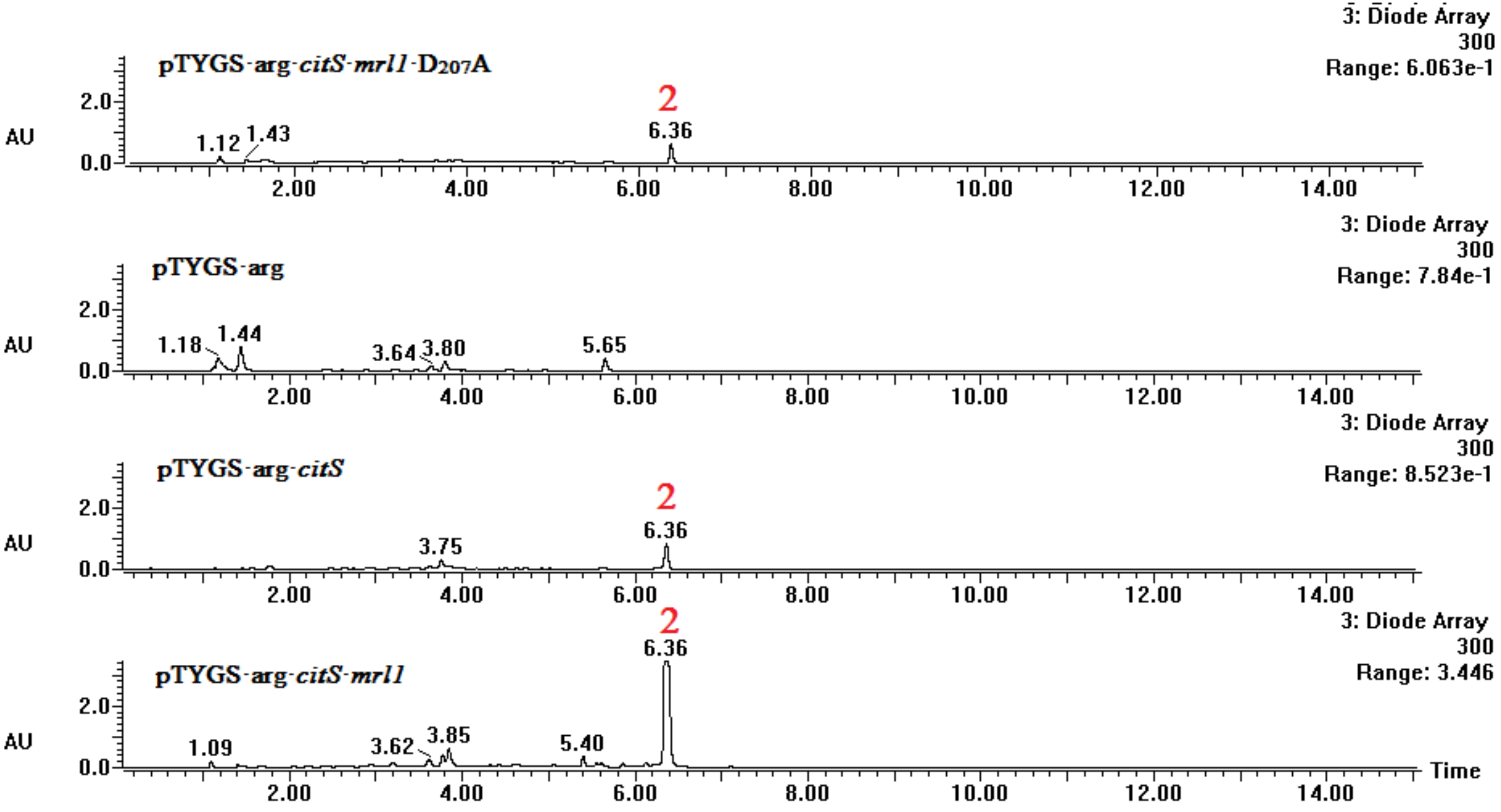

exp. 25 Organic extract of *A. oryzae* M-2-3 + pTYGS·arg·*citS*·*mrl1*·H_{235}A

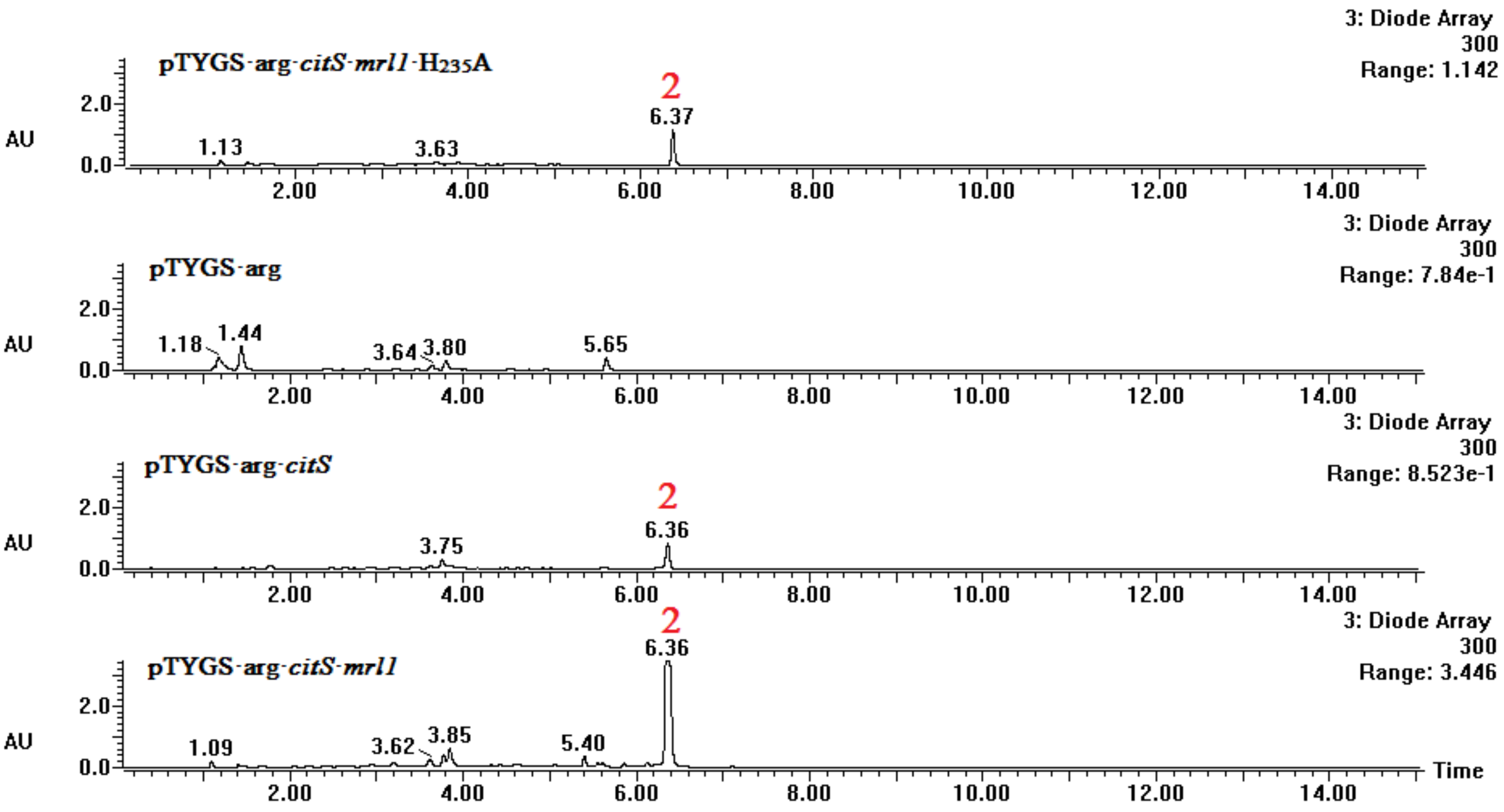

6. LC-HRMS data for compounds 1- 9

UV spectra of compounds 1- 9

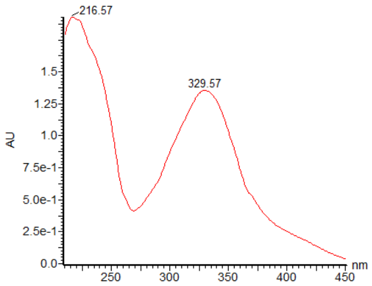

1

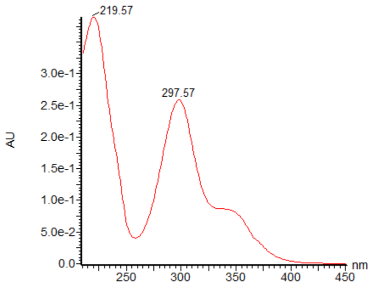

2

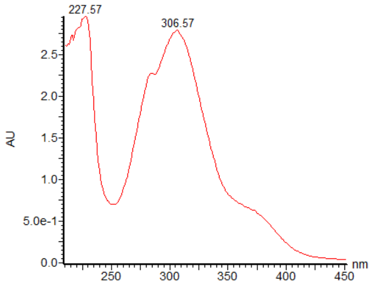

3

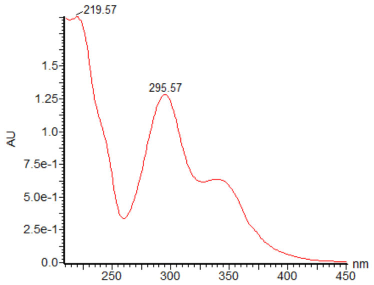

4

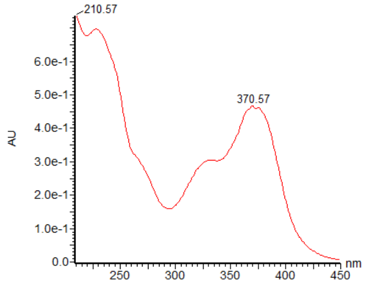

5

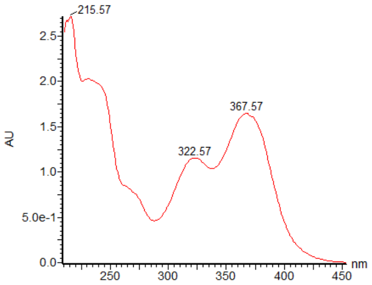

6

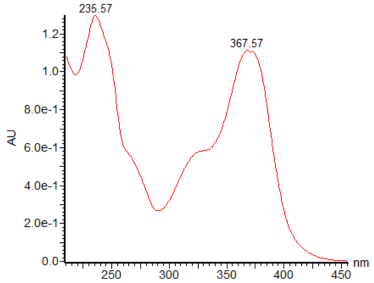

7

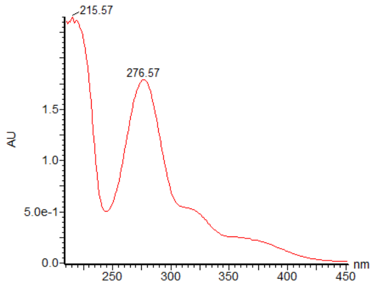

8

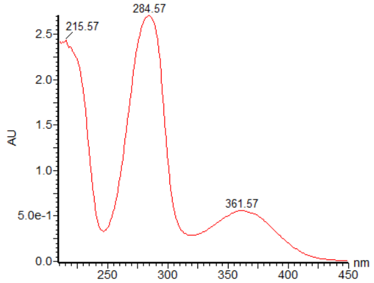

9

Mass spectra of compounds 1- 9 (ES$^-$)

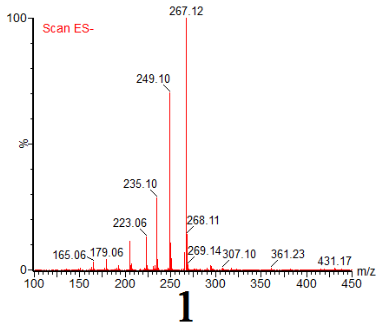

1

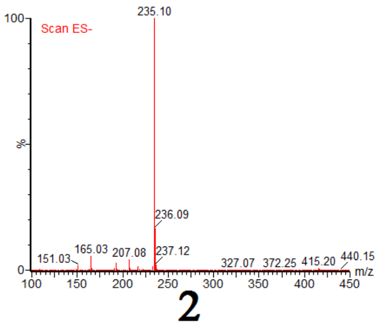

2

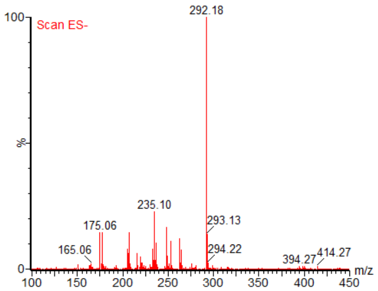

3

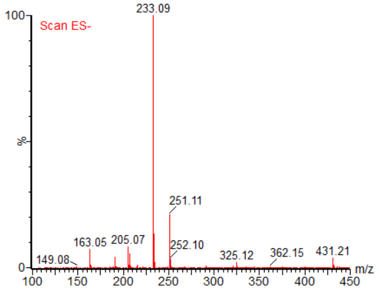

4

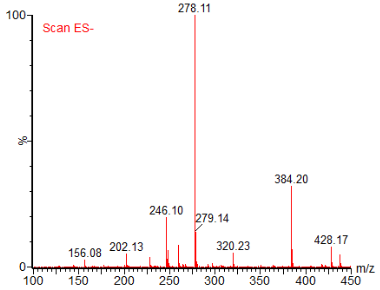

5

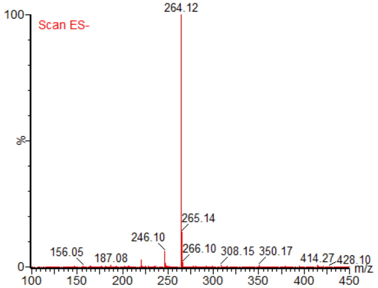

6

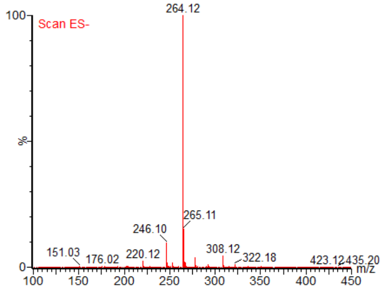

7

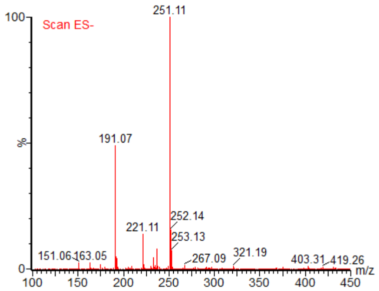

8

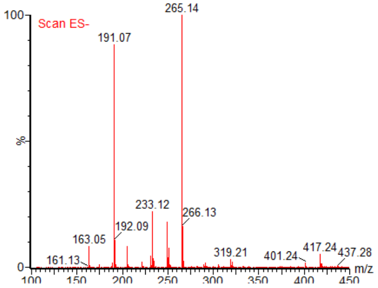

9

HR-MS of 1

Elemental Composition Report

Single Mass Analysis
Tolerance = 15.0 PPM / DBE: min = -1.5, max = 50.0
Element prediction: Off
Number of isotope peaks used for i-FIT = 3

Monoisotopic Mass, Odd and Even Electron Ions
58 formula(e) evaluated with 3 results within limits (up to 50 closest results for each mass)
Elements Used:
C: 0-30 H: 0-60 O: 0-8 Na: 0-1

He Q-Tof Premier UPLC-MS 31-Aug-2015 14:41:58

Yi He Comp-1 297 (3.046) AM (Cen,5, 75.00, Ar,10000.0,556.28,0.70,LS 5) 1: TOF MS ES+ 5.23e+003

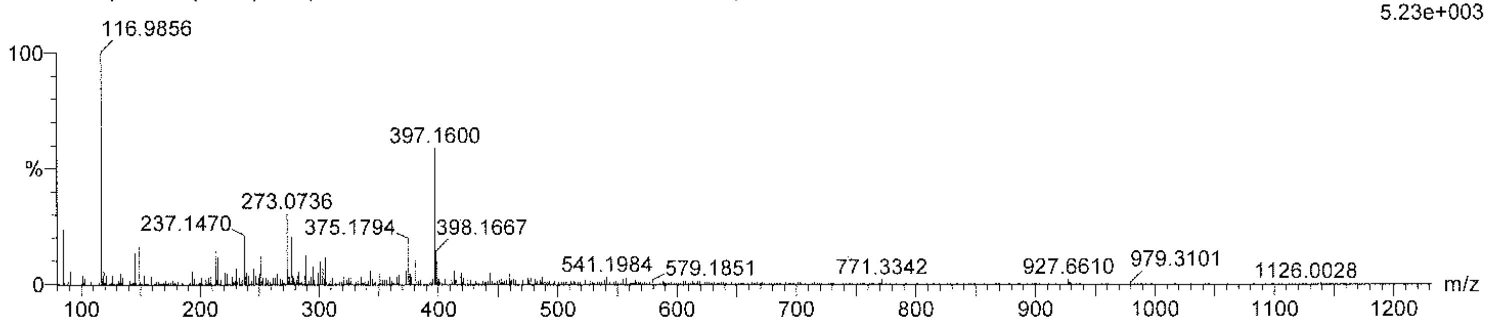

Minimum:				-1.5		
Maximum:		100.0	15.0	50.0		
Mass	Calc. Mass	mDa	PPM	DBE	i-FIT	Formula
273.0736	273.0739	-0.3	-1.1	6.5	57.3	C13 H14 O5 Na
	273.0763	-2.7	-9.9	9.5	52.2	C15 H13 O5
	273.0704	3.2	11.7	18.5	74.0	C22 H9

HR-MS of 2

Elemental Composition Report

Page 1

Single Mass Analysis
Tolerance = 15.0 PPM / DBE: min = -1.5, max = 51.0
Element prediction: Off
Number of isotope peaks used for i-FIT = 3

Monoisotopic Mass, Even Electron Ions
273 formula(e) evaluated with 4 results within limits (all results (up to 1000) for each mass)
Elements Used:
C: 0-100 H: 0-100 N: 0-4 O: 0-10 Na: 0-1

He Q-Tof Premier UPLC-MS 13-May-2014 12:19:33

YH III-3-236prep 307 (3.145) AM (Cen,5, 90.00, Ar,0.0,556.28,0.70,LS 5) 1: TOF MS ES+ 1.36e+003

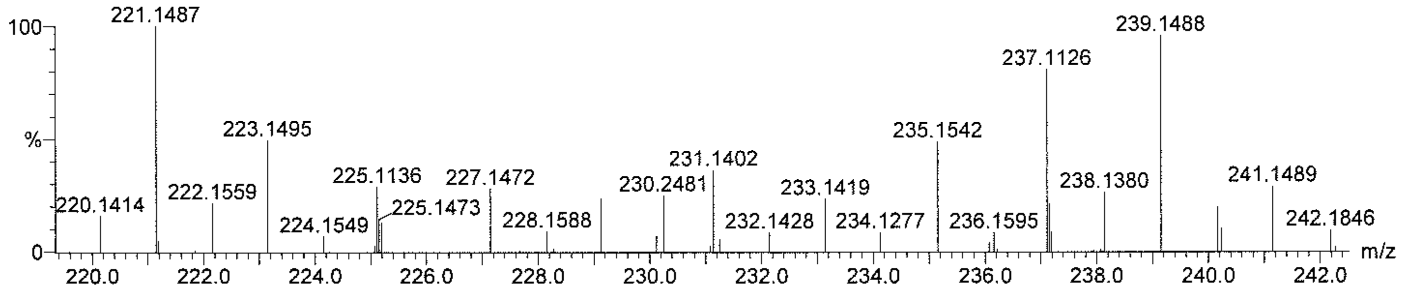

Minimum:				-1.5		
Maximum:		6.0	15.0	51.0		
Mass	Calc. Mass	mDa	PPM	DBE	i-FIT	Formula
237.1126	237.1127	-0.1	-0.4	5.5	738.3	C13 H17 O4
	237.1116	1.0	4.2	7.5	752.9	C12 H14 N4 Na
	237.1140	-1.4	-5.9	10.5	725.2	C14 H13 N4
	237.1103	2.3	9.7	2.5	768.2	C11 H18 O4 Na

HR-MS of 3

Elemental Composition Report

Single Mass Analysis

Tolerance = 20.0 PPM / DBE: min = -1.5, max = 50.0
Element prediction: Off
Number of isotope peaks used for i-FIT = 3

Monoisotopic Mass, Even Electron Ions
459 formula(e) evaluated with 6 results within limits (up to 50 closest results for each mass)
Elements Used:
C: 0-40 H: 0-70 N: 0-10 O: 0-8 S: 0-1

He Q-Tof Premier UPLC-MS 28-Jul-2015 08:56:22

YH III-66-Comp11 neg 291 (2.760) AM (Cen,5, 70.00, Ar,10000.0,554.26,0.70,LS 5); Cm (280:300) 1: TOF MS ES- 2.16e+004

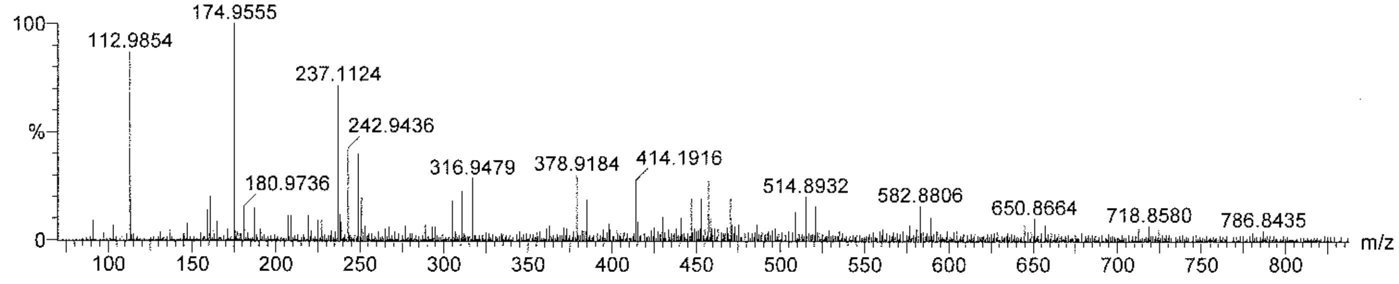

Mass	Calc. Mass	mDa	PPM	DBE	i-FIT	Formula
Minimum:				-1.5		
Maximum:		100.0	20.0	50.0		
237.1124	237.1127	-0.3	-1.3	5.5	704.9	C13 H17 O4
	237.1134	-1.0	-4.2	1.5	462.1	C6 H17 N6 O2 S
	237.1140	-1.6	-6.7	10.5	766.6	C14 H13 N4
	237.1100	2.4	10.1	6.5	848.8	C9 H13 N6 O2
	237.1161	-3.7	-15.6	0.5	322.0	C10 H21 O4 S
	237.1087	3.7	15.6	1.5	884.0	C8 H17 N2 O6

HR-MS of 4

Elemental Composition Report

Page 1

Single Mass Analysis

Tolerance = 10.0 mDa / DBE: min = -1.5, max = 50.0
Element prediction: Off
Number of isotope peaks used for i-FIT = 3

Monoisotopic Mass, Even Electron Ions
61 formula(e) evaluated with 4 results within limits (up to 50 closest results for each mass)
Elements Used:
C: 0-25 H: 0-50 O: 0-10 Na: 0-1

He Q-Tof Premier UPLC-MS 22-Jan-2015
11:16:21

Yi He III-24-E-252 490 (5.018) AM (Cen,2, 80.00, Ar,10000.0,556.28,0.70,LS 5) 1: TOF MS ES+
1.59e+003

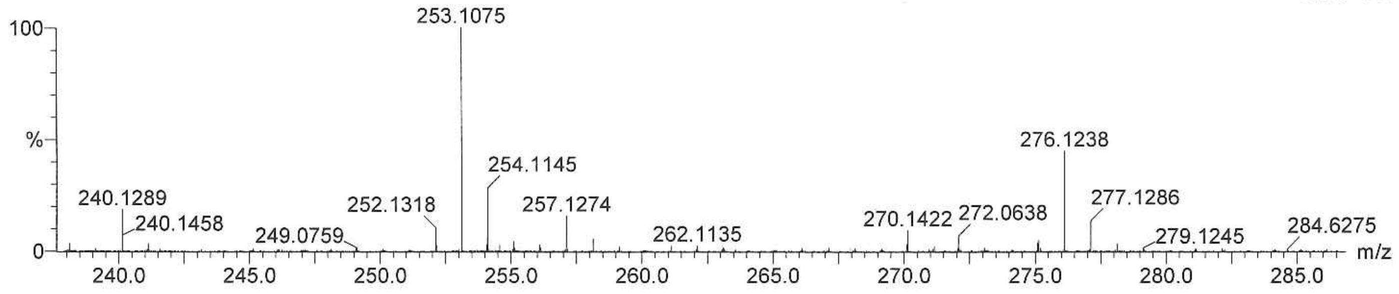

Minimum:				-1.5		
Maximum:		10.0	10.0	50.0		
Mass	Calc. Mass	mDa	PPM	DBE	i-FIT	Formula
253.1075	253.1076	-0.1	-0.4	5.5	53.3	C13 H17 O5
	253.1052	2.3	9.1	2.5	74.5	C11 H18 O5 Na
	253.1017	5.8	22.9	14.5	16.8	C20 H13
	253.0993	8.2	32.4	11.5	31.0	C18 H14 Na

HR-MS of 5

Elemental Composition Report **Page 1**

Single Mass Analysis
Tolerance = 10.0 mDa / DBE: min = -1.5, max = 50.0
Element prediction: Off
Number of isotope peaks used for i-FIT = 3

Monoisotopic Mass, Odd and Even Electron Ions
124 formula(e) evaluated with 9 results within limits (up to 50 closest results for each mass)
Elements Used:
C: 0-50 H: 0-60 N: 0-3 O: 0-8

He Q-Tof Premier UPLC-MS 15-Jul-2015
13:44:27

YiHe III-48-prepC 265 (2.719) AM (Cen,5, 55.00, Ar,10000.0,556.28,0.70,LS 5) 1: TOF MS ES+
1.92e+003

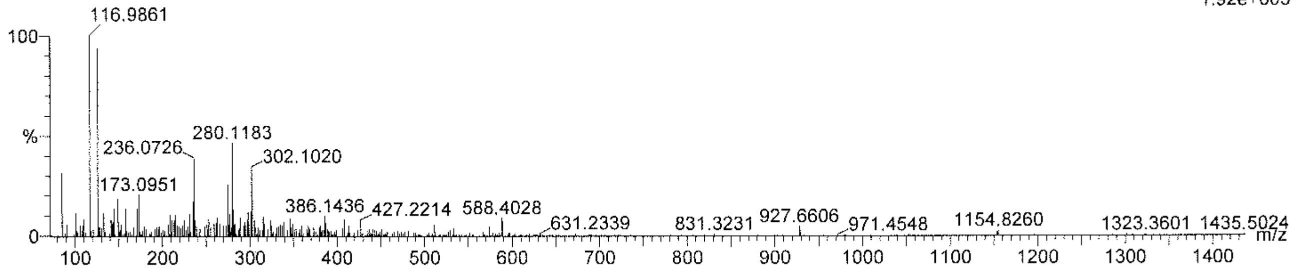

Minimum:				-1.5		
Maximum:		10.0	50.0	50.0		
Mass	Calc. Mass	mDa	PPM	DBE	i-FIT	Formula
280.1183	280.1185	-0.2	-0.7	6.5	57.7	C14 H18 N O5
	280.1158	2.5	8.9	2.0	71.3	C11 H20 O8
	280.1212	-2.9	-10.4	11.0	48.3	C17 H16 N2 O2
	280.1145	3.8	13.6	2.5	79.9	C9 H18 N3 O7
	280.1126	5.7	20.3	15.5	37.6	C21 H14 N
	280.1252	-6.9	-24.6	15.0	39.7	C22 H16
	280.1099	8.4	30.0	11.0	45.1	C18 H16 O3
	280.1271	-8.8	-31.4	2.0	81.0	C10 H20 N2 O7
	280.1086	9.7	34.6	11.5	51.8	C16 H14 N3 O2

HR-MS of 6

Elemental Composition Report

Single Mass Analysis

Tolerance = 10.0 mDa / DBE: min = -1.5, max = 50.0
Element prediction: Off
Number of isotope peaks used for i-FIT = 3

Monoisotopic Mass, Odd and Even Electron Ions
118 formula(e) evaluated with 8 results within limits (up to 50 closest results for each mass)
Elements Used:
C: 0-50 H: 0-60 N: 0-3 O: 0-8

He | Q-Tof Premier UPLC-MS | 15-Jul-2015 13:53:01

YiHe III-60-265b 243 (2.491) AM (Cen,5, 73.00, Ar,10000.0,556.28,0.70,LS 5) | 1: TOF MS ES+ 3.08e+003

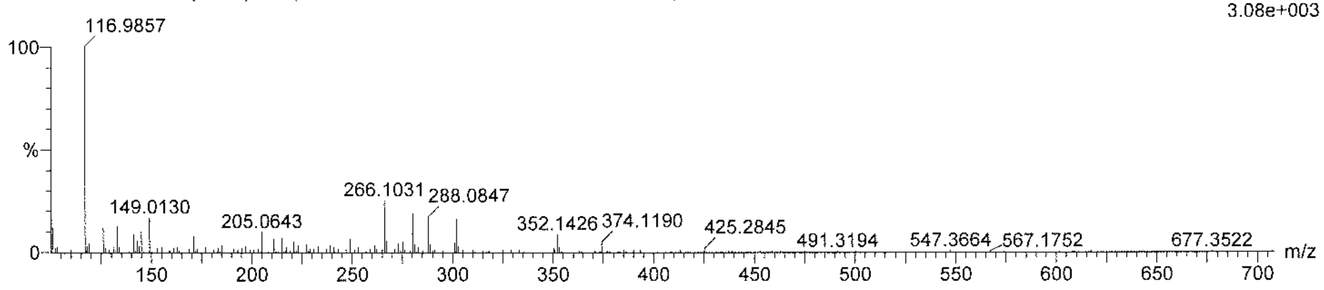

Mass	Calc. Mass	mDa	PPM	DBE	i-FIT	Formula
Minimum:				-1.5		
Maximum:		10.0	50.0	50.0		
266.1031	266.1028	0.3	1.1	6.5	n/a	C13 H16 N O5
	266.1055	-2.4	-9.0	11.0	n/a	C16 H14 N2 O2
	266.1002	2.9	10.9	2.0	n/a	C10 H18 O8
	266.0988	4.3	16.2	2.5	n/a	C8 H16 N3 O7
	266.0970	6.1	22.9	15.5	n/a	C20 H12 N
	266.1096	-6.5	-24.4	15.0	n/a	C21 H14
	266.1114	-8.3	-31.2	2.0	n/a	C9 H18 N2 O7
	266.0943	8.8	33.1	11.0	n/a	C17 H14 O3

HR-MS of 7

Elemental Composition Report

Single Mass Analysis

Tolerance = 20.0 PPM / DBE: min = -1.5, max = 50.0
Element prediction: Off
Number of isotope peaks used for i-FIT = 3

Monoisotopic Mass, Even Electron Ions
795 formula(e) evaluated with 16 results within limits (up to 50 closest results for each mass)
Elements Used:
C: 0-60 H: 0-90 N: 0-11 O: 0-10 Na: 0-1

He Q-Tof Premier UPLC-MS 30-Jul-2015 09:35:07

YH III-65-Comp10 255 (2.608) AM (Cen,5, 70.00, Ar,10000.0,556.28,0.70,LS 5) 1: TOF MS ES+ 1.26e+003

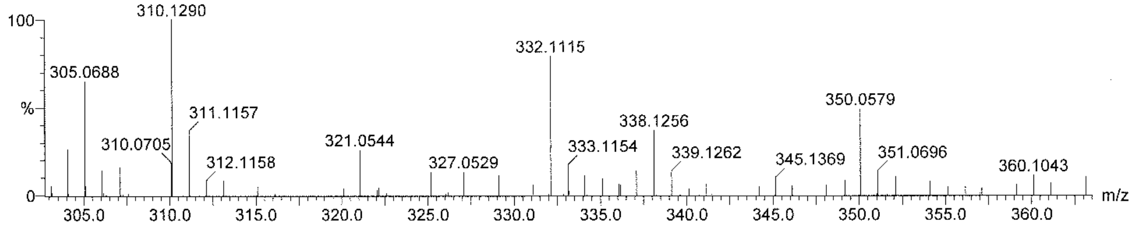

Minimum:				-1.5		
Maximum:		100.0	20.0	50.0		

Mass	Calc. Mass	mDa	PPM	DBE	i-FIT	Formula
310.1290	310.1291	-0.1	-0.3	6.5	85.0	C15 H20 N O6
	310.1280	1.0	3.2	8.5	90.6	C14 H17 N5 O2 Na
	310.1277	1.3	4.2	12.5	95.7	C12 H12 N11
	310.1304	-1.4	-4.5	11.5	74.5	C16 H16 N5 O2
	310.1312	-2.2	-7.1	0.5	182.3	C3 H17 N11 O5 Na
	310.1267	2.3	7.4	3.5	102.8	C13 H21 N O6 Na
	310.1264	2.6	8.4	7.5	107.7	C11 H16 N7 O4
	310.1320	-3.0	-9.7	12.5	61.9	C19 H17 N3 Na
	310.1323	-3.3	-10.6	-1.5	177.1	C4 H20 N7 O9
	310.1253	3.7	11.9	9.5	114.6	C10 H13 N11 Na
	310.1250	4.0	12.9	2.5	122.1	C10 H20 N3 O8
	310.1336	-4.6	-14.8	3.5	160.4	C5 H16 N11 O5
	310.1339	-4.9	-15.8	-0.5	155.5	C7 H21 N5 O7 Na
	310.1240	5.0	16.1	4.5	128.3	C9 H17 N7 O4 Na
	310.1344	-5.4	-17.4	15.5	50.6	C21 H16 N3
	310.1232	5.8	18.7	15.5	40.8	C22 H16 N O

HR-MS of 8

Elemental Composition Report **Page 1**

Single Mass Analysis
Tolerance = 10.0 mDa / DBE: min = -1.5, max = 50.0
Element prediction: Off
Number of isotope peaks used for i-FIT = 3

Monoisotopic Mass, Even Electron Ions
71 formula(e) evaluated with 5 results within limits (up to 50 closest results for each mass)
Elements Used:
C: 0-50 H: 0-60 O: 0-10 Na: 0-1

He Q-Tof Premier UPLC-MS 16-Jul-2015 08:10:42

YiHe III-32 265 (2.719) AM (Cen,5, 30.00, Ar,10000.0,556.28,0.70,LS 5) 1: TOF MS ES+ 1.30e+003

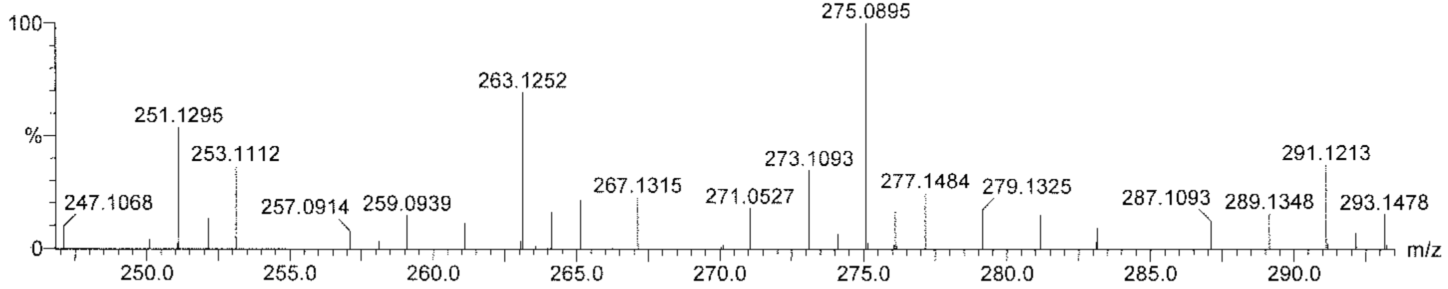

Minimum:				-1.5		
Maximum:		10.0	50.0	50.0		
Mass	Calc. Mass	mDa	PPM	DBE	i-FIT	Formula
275.0895	275.0895	0.0	0.0	5.5	168.4	C13 H16 O5 Na
	275.0919	-2.4	-8.7	8.5	160.6	C15 H15 O5
	275.0861	3.4	12.4	17.5	181.7	C22 H11
	275.0837	5.8	21.1	14.5	182.9	C20 H12 Na
	275.0978	-8.3	-30.2	-0.5	175.5	C8 H19 O10

HR-MS of 9

Elemental Composition Report

Single Mass Analysis

Tolerance = 10.0 mDa / DBE: min = -1.5, max = 50.0
Element prediction: Off
Number of isotope peaks used for i-FIT = 3

Monoisotopic Mass, Odd and Even Electron Ions
35 formula(e) evaluated with 2 results within limits (up to 50 closest results for each mass)
Elements Used:
C: 0-30 H: 0-60 O: 0-10

He Q-Tof Premier UPLC-MS 17-Jul-2015 09:23:53

YiHe III-32-266 329 (3.117) AM (Cen,5, 80.00, Ar,10000.0,554.26,0.70,LS 5) 1: TOF MS ES- 5.75e+003

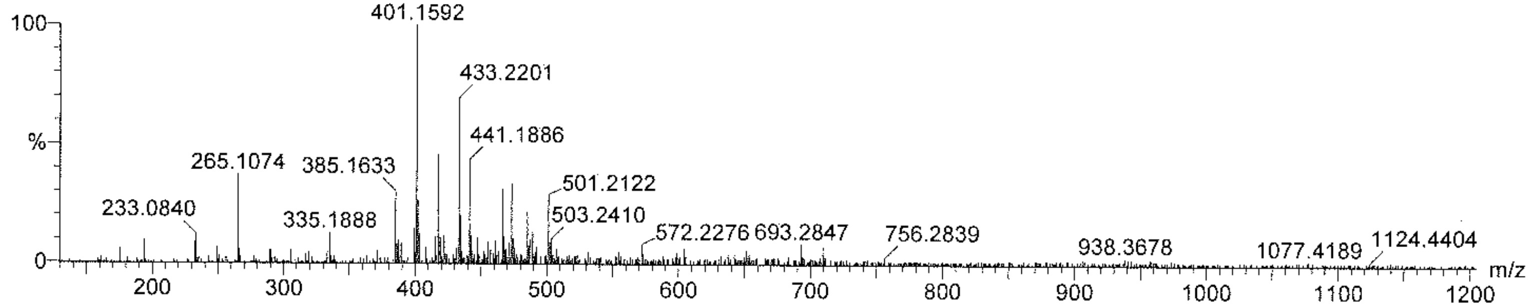

Minimum:				-1.5		
Maximum:		10.0	50.0	50.0		
Mass	Calc. Mass	mDa	PPM	DBE	i-FIT	Formula
265.1074	265.1076	-0.2	-0.8	6.5	38.4	C14 H17 O5
	265.1017	5.7	21.5	15.5	70.4	C21 H13

7. NMR data for compounds 1 - 9

NMR data for compound **1**

	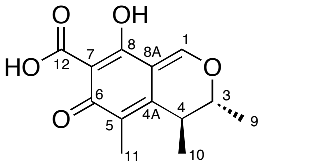 Chemical Formula: $C_{13}H_{14}O_5$ Exact Mass: 250.0841	
Pos.	d_H ($CDCl_3$) 400 MHz / ppm	d_C ($CDCl_3$) 100 MHz / ppm
1	8.23, s, 1H	163.0
3	4.77, q, J = 6.7 Hz, 1H	81.9
4	2.97, q, J = 7.2 Hz, 1H	34.8
4A	-	139.2
5	-	123.3
6	-	184.1
7	-	100.6
8	-	177.5
8A	-	107.7
9	1.33, d, J = 6.7 Hz, 3H	18.6
10	1.21, d, J = 7.2 Hz, 3H	18.4
11	2.00, s, 3H	9.6
12	-	174.8

NMR data for compound **2**

	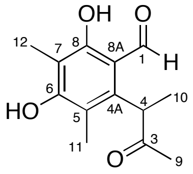 Chemical Formula: $C_{13}H_{16}O_4$ Exact Mass: 236.1049	
Pos.	d_H ($CDCl_3$) 500 MHz / ppm	d_C ($CDCl_3$) 125 MHz / ppm
1	9.91, brs , 1H	192.9
3	-	208.3
4	4.11, q, *J* = 6.9 Hz, 1H	48.8
4A	-	141.8
5	-	115.1
6	-	160.0
7	-	108.9
8	-	162.9
8A	-	112.3
8-OH	13.01, s, 1H	-
9	2.04, s, 3H	28.4
10	1.48, d, *J* = 6.9 Hz, 3H	17.0
11	2.16, brs, 3H	11.5
12	2.14, s, 3H	7.2

NMR data for compound **3**

	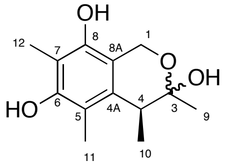 **3** Chemical Formula: $C_{13}H_{18}O_4$ Exact Mass: 238.1205 3.7 min

Pos.	d_H (DMSO-d6) 400 MHz / ppm	d_C (DMSO-d6) 100 MHz / ppm
1	4.56, 1H, d, *J* = 15.8 Hz<br>4.51, 1H, d, *J* = 15.8 Hz	40.1
3	-	96.2
4	2.61, 1H, q, *J* = 6.9 Hz	38.0
4A	-	135.3
5	-	113.7
6	-	152.1
7	-	109.9
8	-	148.2
8A	-	112.7
9	1.36, 3H, s	27.1
10	0.97, 3H, d, *J* = 6.9 Hz	18.8
11	1.98, 3H, s	9.9
12	2.01, 3H, s	10.9

NMR data for compound **4**

	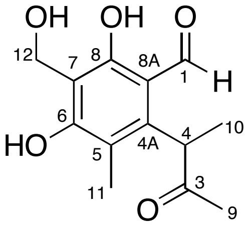 Chemical Formula: $C_{13}H_{16}O_5$ Exact Mass: 252.0998

Pos.	d_H (CD_3OD) 400 MHz / ppm	d_C (CD_3OD) 100 MHz / ppm
1	9.94, s, 1H	194.5
3	-	211.0
4	4.39, q, *J* = 6.93 Hz, 1H	50.4
4A	-	145.8
5	-	118.6
6	-	165.1
7	-	109.8
8	-	163.9
8A	-	112.8
9	2.03, s, 3H	29.2
10	1.45, d, *J* = 6.93 Hz, 3H	16.9
11	2.11, s, 3H	11.6
12	4.72, s, 2H	66.0

NMR data for compound **5**

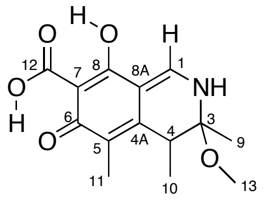

Chemical Formula: $C_{14}H_{17}NO_5$
Exact Mass: 279.1107

Pos.	d_H (DMSO-d6) 400 MHz / ppm	d_C (DMSO-d6) 100 MHz / ppm
1	8.61, s, 1H.	155.2
3	-	87.2
4	3.26, q, *J* = 7.2, 1H.	38.8
4A	-	146.5
5	-	116.0
6	-	175.6
7	-	100.6
8	(O) 16.61, s, 1H.	175.4
8A	-	102.7
9	1.55, 3H.	18.7
10	0.96, d, *J* = 7.2, 3H.	16.2
11	1.98, s, 3H.	9.5
12	17.02, s, 1H.	170.6
13	3.11, s, 3H.	50.1

NMR data for compound **6**

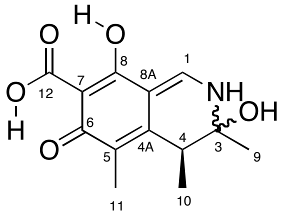

Chemical Formula: $C_{13}H_{15}NO_5$
Exact Mass: 265.0950

	**A** hemiaminal diastereomer 1		**B** hemiaminal diastereomer 2	
Pos.	d_H (DMSO-d6) 400 MHz/ ppm	d_C (DMSO-d6) 100 MHz / ppm	d_H (DMSO-d6) 400 MHz / ppm	d_C (DMSO-d6) 100MHz / ppm
1	8.38, s, 1H.	154.2	8.54, s, 1H.	154.7
3	-	85.8	-	83.1
4	3.12, q, *J* = 7.1, 1H.	40.4	3.19, q, *J* = 7.2, 1H.	39.9
4A	-	146.1	-	146.6
5	-	115.4	-	115.7
6	-	173.8	-	174.5
7	-	100.3	-	100.9
8	(OH) 16.54, s, 1H.	175.4	(OH) 16.67, s, 1H.	175.5
8A	-	101.2	-	102.3
9	1.39, s, 3H.	26.0	1.57, 3H.	24.7
10	1.00, d, *J* = 7.1, 3H.	14.0	0.92, d, *J* = 7.2, 3H.	16.5
11	2.001, s, 3H.	9.5	1.995, s, 3H.	9.5
12	16.92, s, 1H.	169.0	16.99, s, 1H.	169.6

NMR data for compound **7**

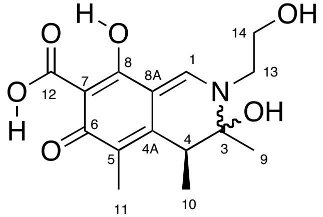

Chemical Formula: $C_{15}H_{19}NO_6$
Exact Mass: 309.1212

Pos.	d_H (DMSO-d6) 400 MHz / ppm	d_C (DMSO-d6) 100 MHz /ppm
1	8.45, 1H, s	143.5
3	-	90.6
4	3.18, 1H, m	41.7
4A	-	145.9
5	-	115.5
6	-	174.0
7	-	110.1
8	(OH) 14.55, 1H, s	158.7
8A	-	101.1
9	1.35, 3H, s	24.2
10	1.02, 1H, d, *J* = 7.0 Hz	14.0
11	2.01, 3H, s	10.7
12	(OH) 16.90, 1H, s	173.4
13	3.75, 1H, m, 3.91, 1H, m	53.2
14	3.77, 1H, m, 3.64, 1H, m	59.4

NMR data for compound **8**

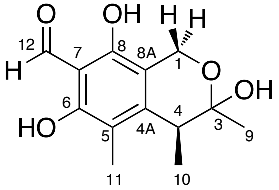

Chemical Formula: $C_{13}H_{16}O_5$
Exact Mass: 252.0998

Pos.	d_H (DMSO-d6) 400 MHz / ppm	d_C (DMSO-d6) 100 MHz / ppm
1	4.59, d, *J* = 9.4 Hz, 1H<br>4.53, d, *J* = 9.4 Hz, 1H	58.5
3	-	96.1
4	2.71, q, *J* = 6.95 Hz, 1H	39.1
4A	-	150.1
5	-	114.0
6	-	157.7
7	-	109.5
8	-	155.0
8A	-	112.4
9	1.38, s, 3H	26.8
10	1.01, d, *J* = 6.95 Hz, 3H	18.6
11	1.98, s, 3H	9.8
12	10.22, s, 1H	194.6

7.1 NMR Spectra Compound 1

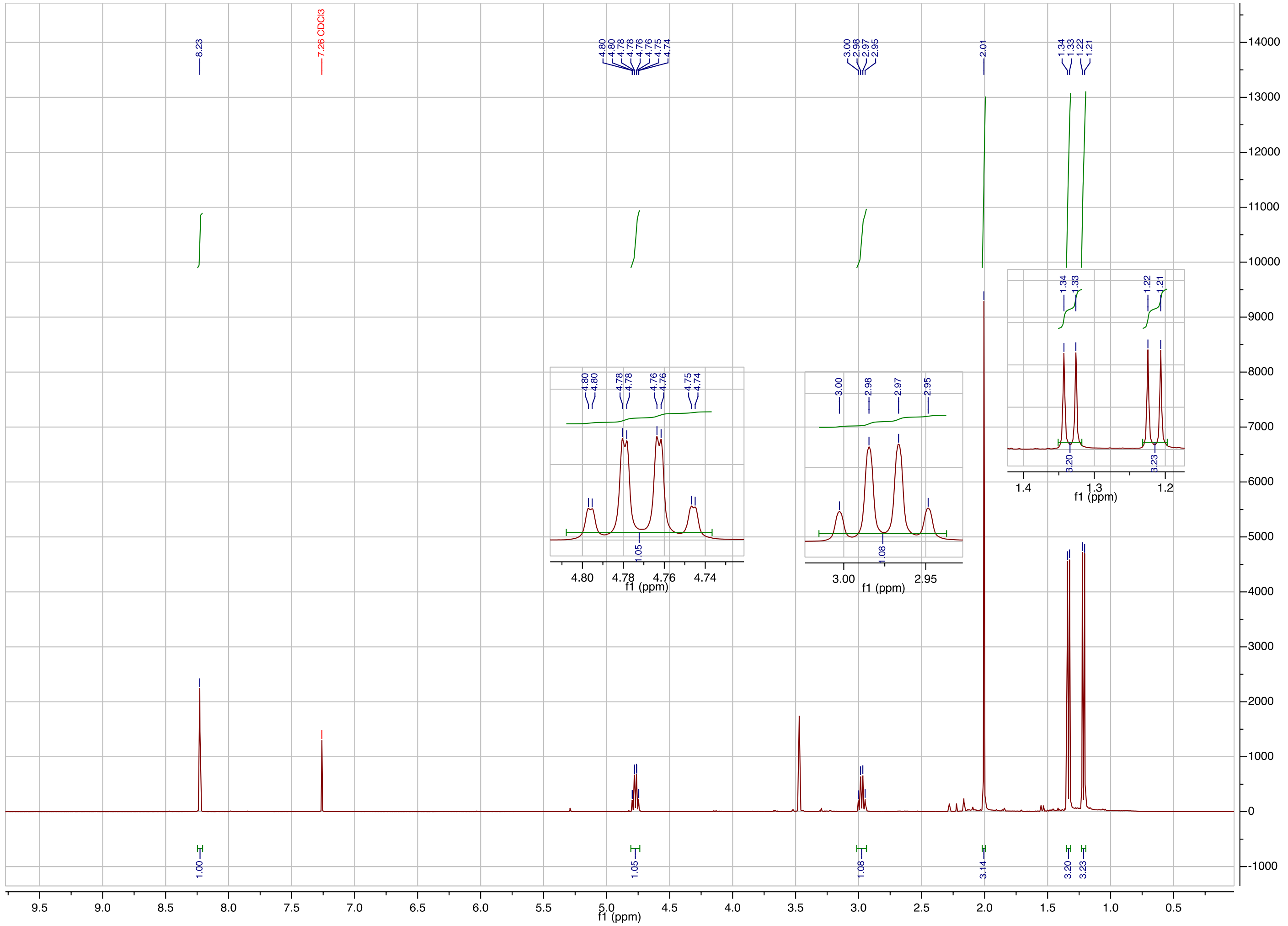

Compound 1 ^{1}H NMR $CDCl_3$, 400 MHz.

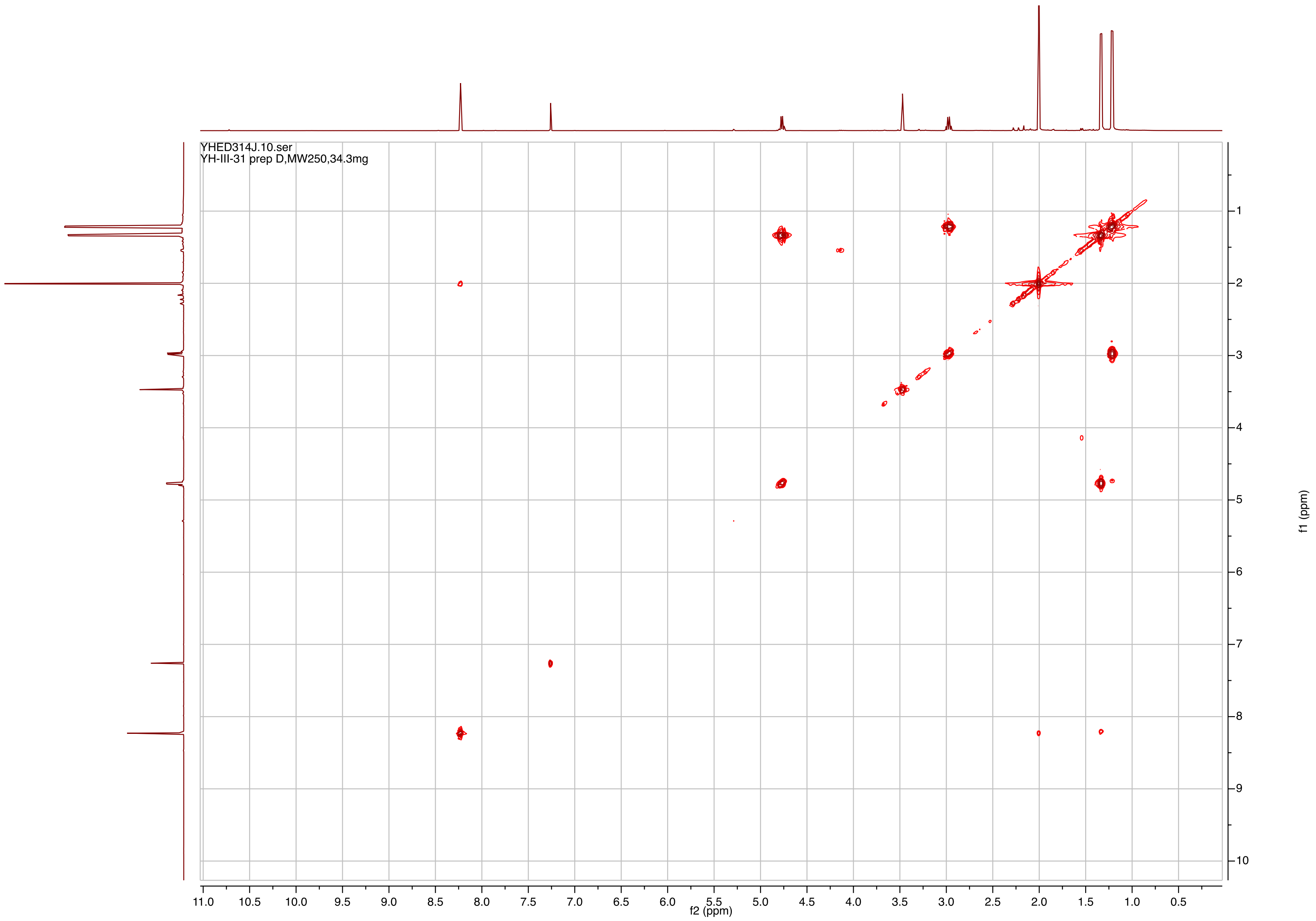

Compound 1 COSY

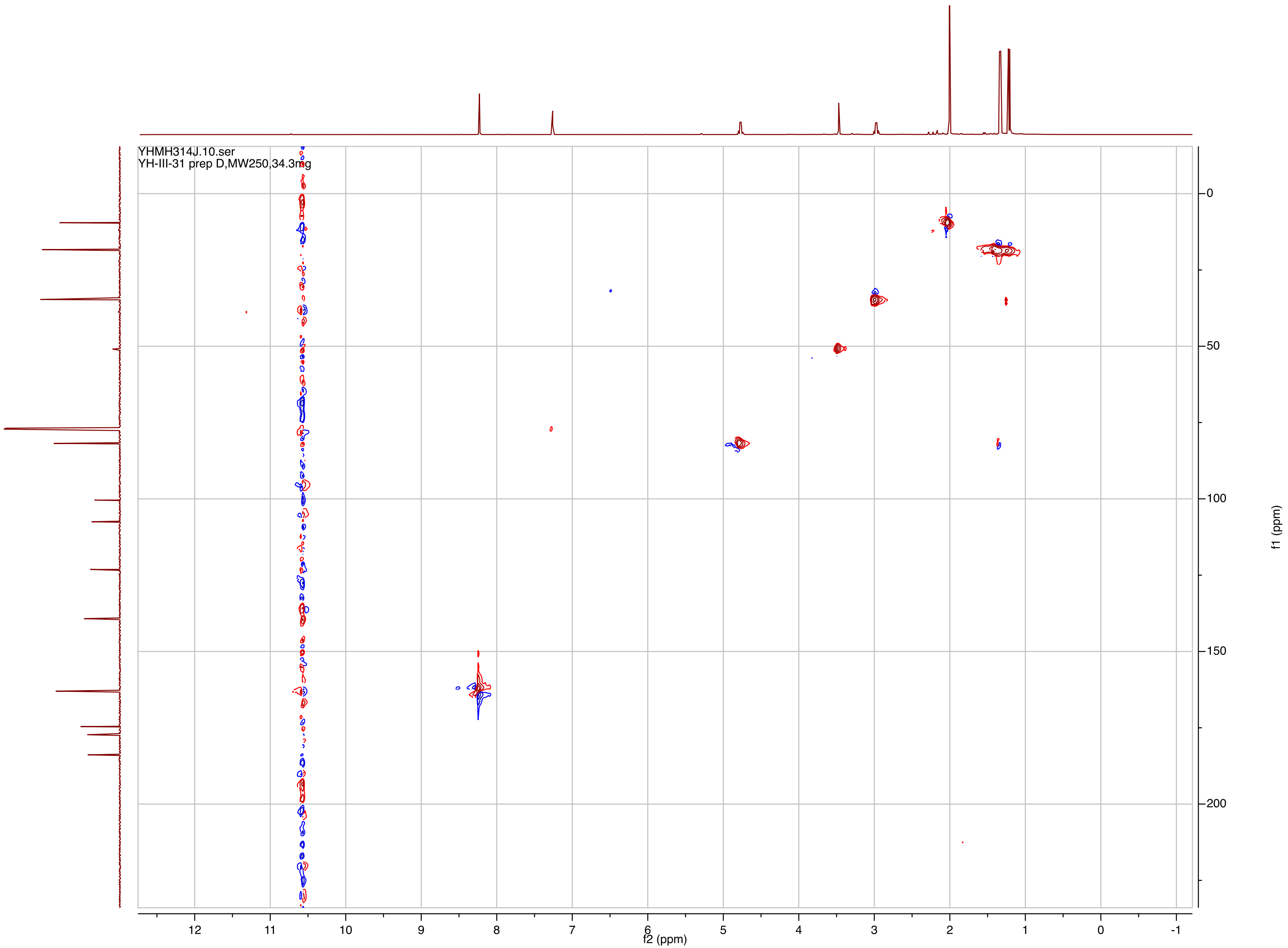

Compound 1 HSQC

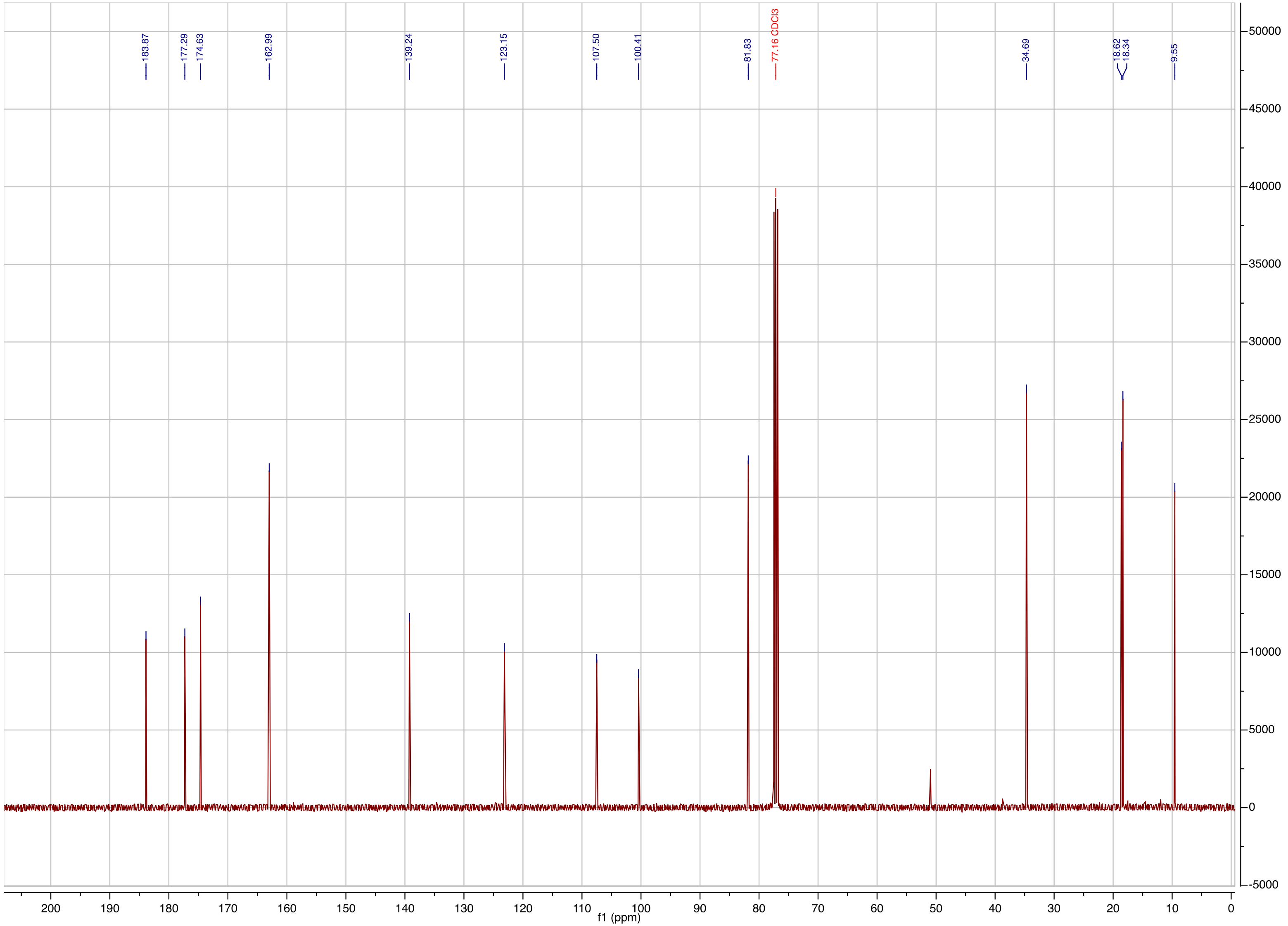

Compound 1 ^{13}C NMR $CDCl_3$ 100 MHz

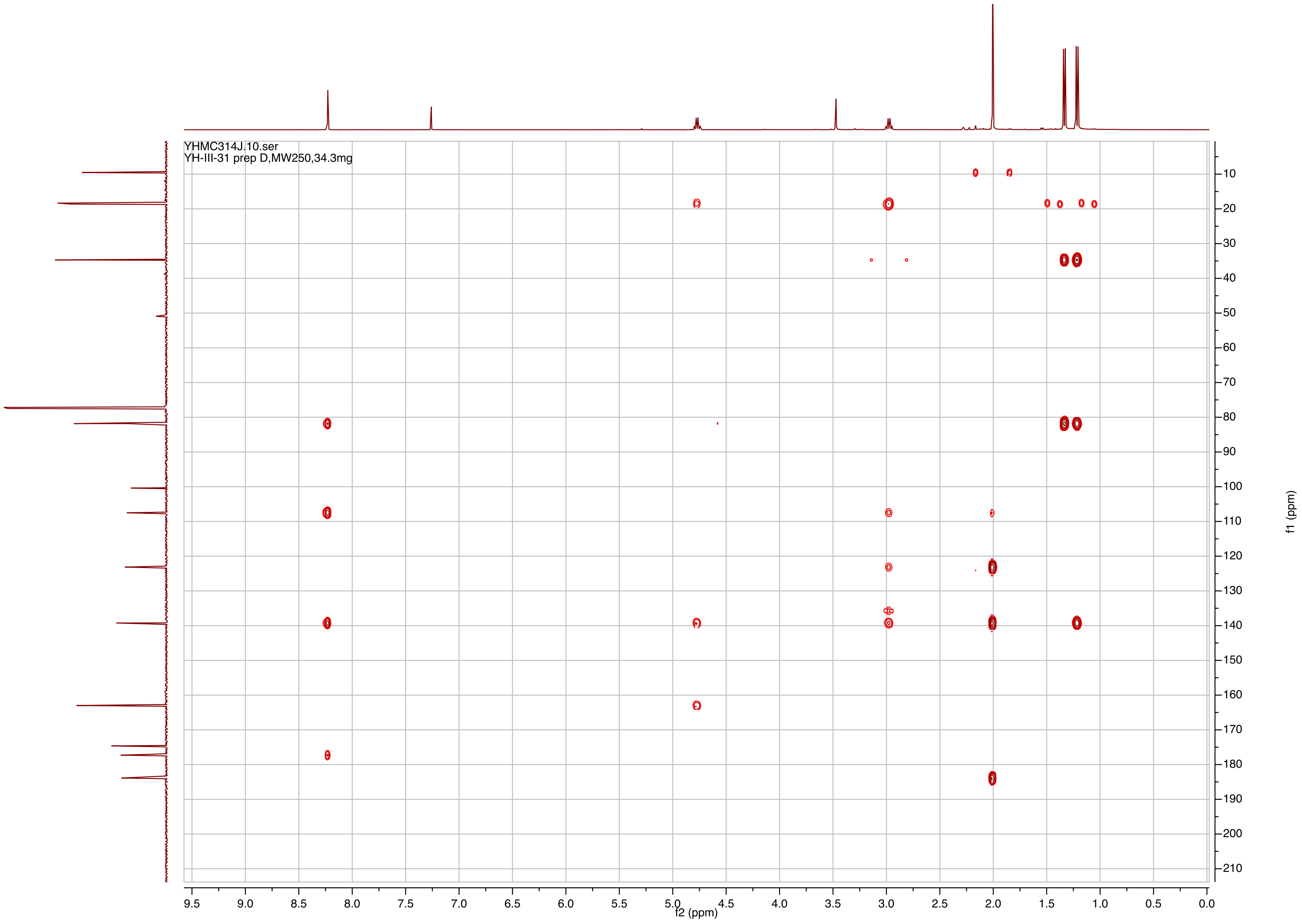

Compound 1 HMBC

7.2 NMR Spectra Compound 2

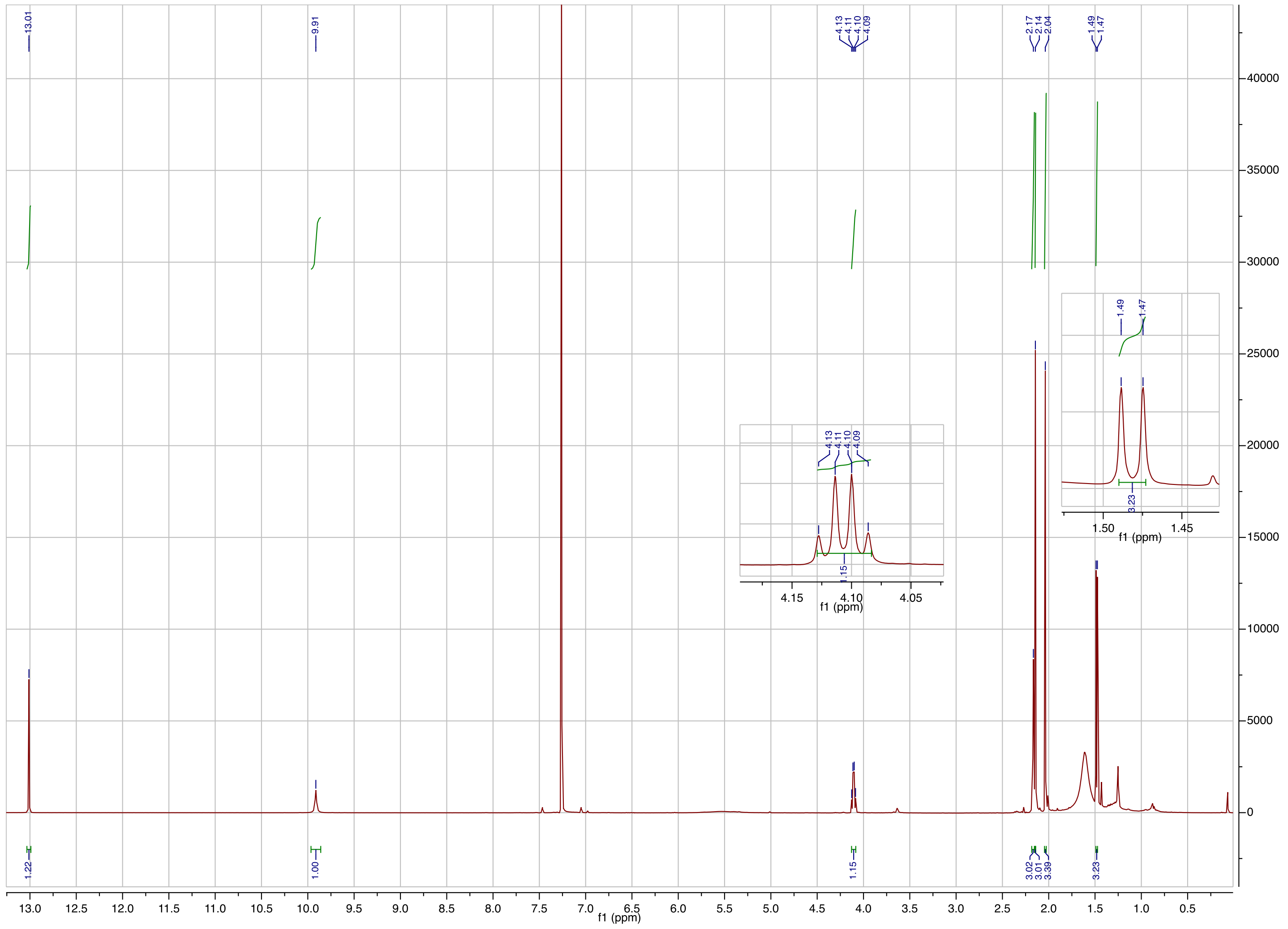

Compound 2 ^{1}H NMR $CDCl_3$ 500 MHz

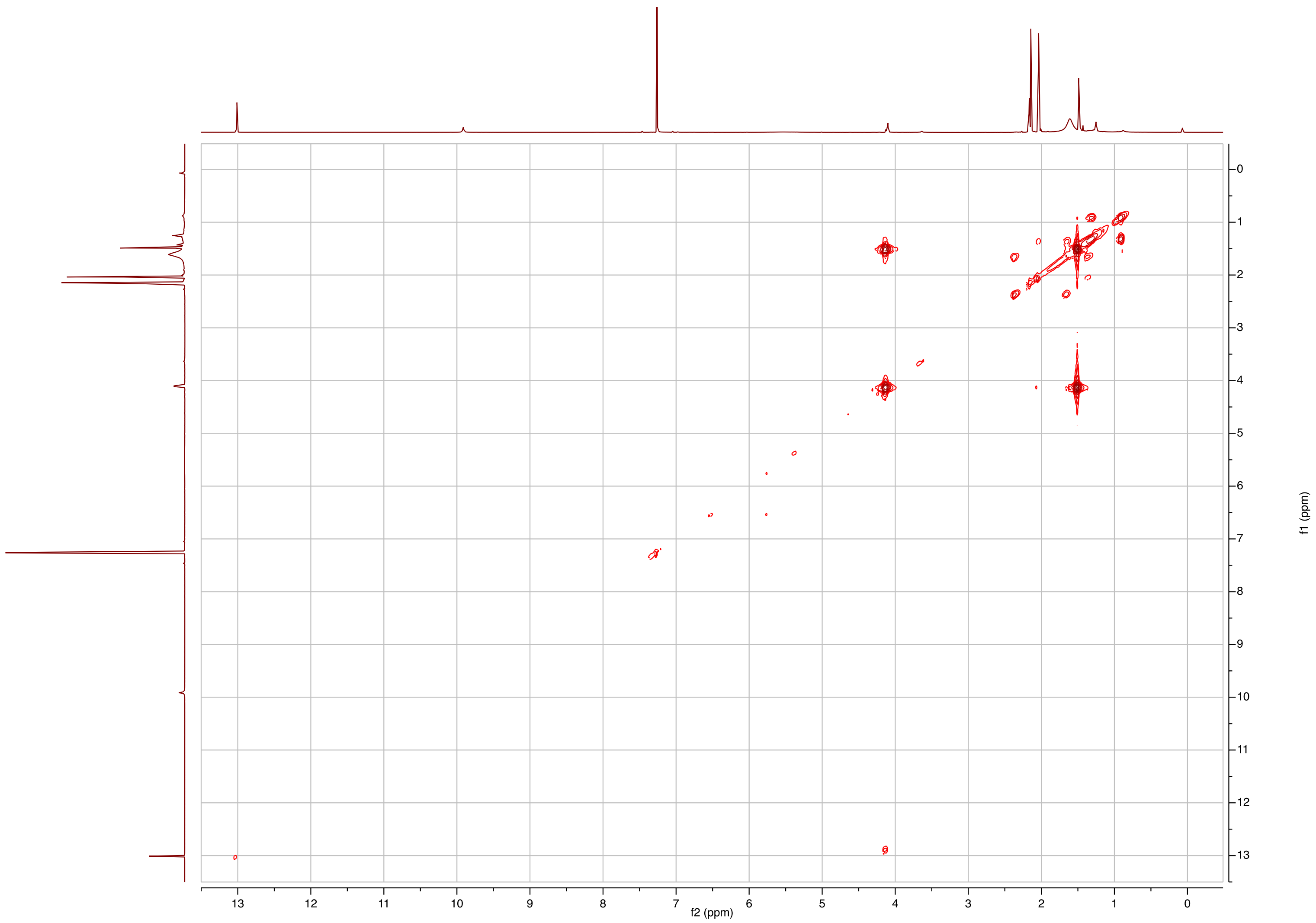

Compound 2 COSY

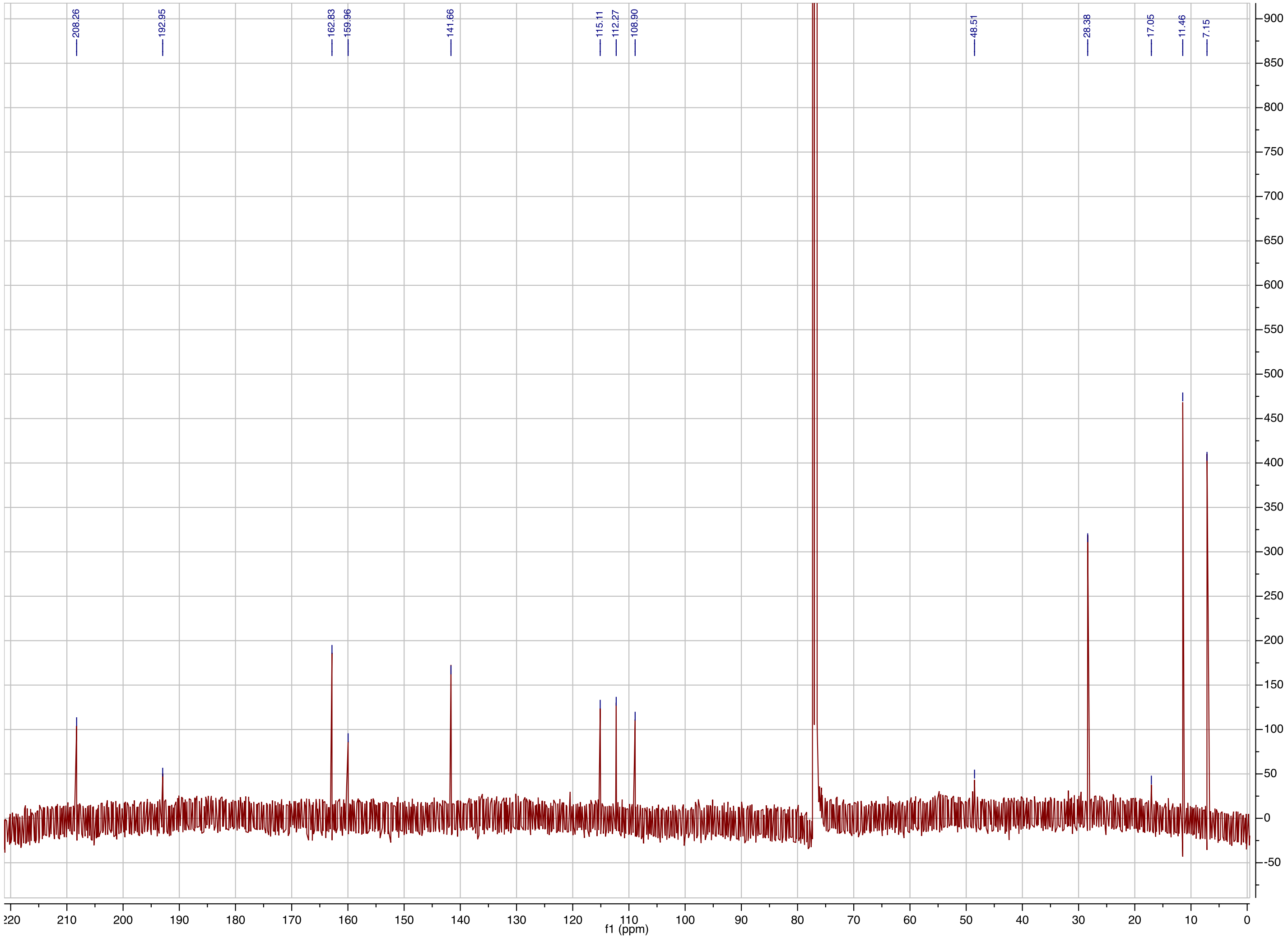

Compound 2 ^{13}C NMR $CDCl_3$ 125 MHz

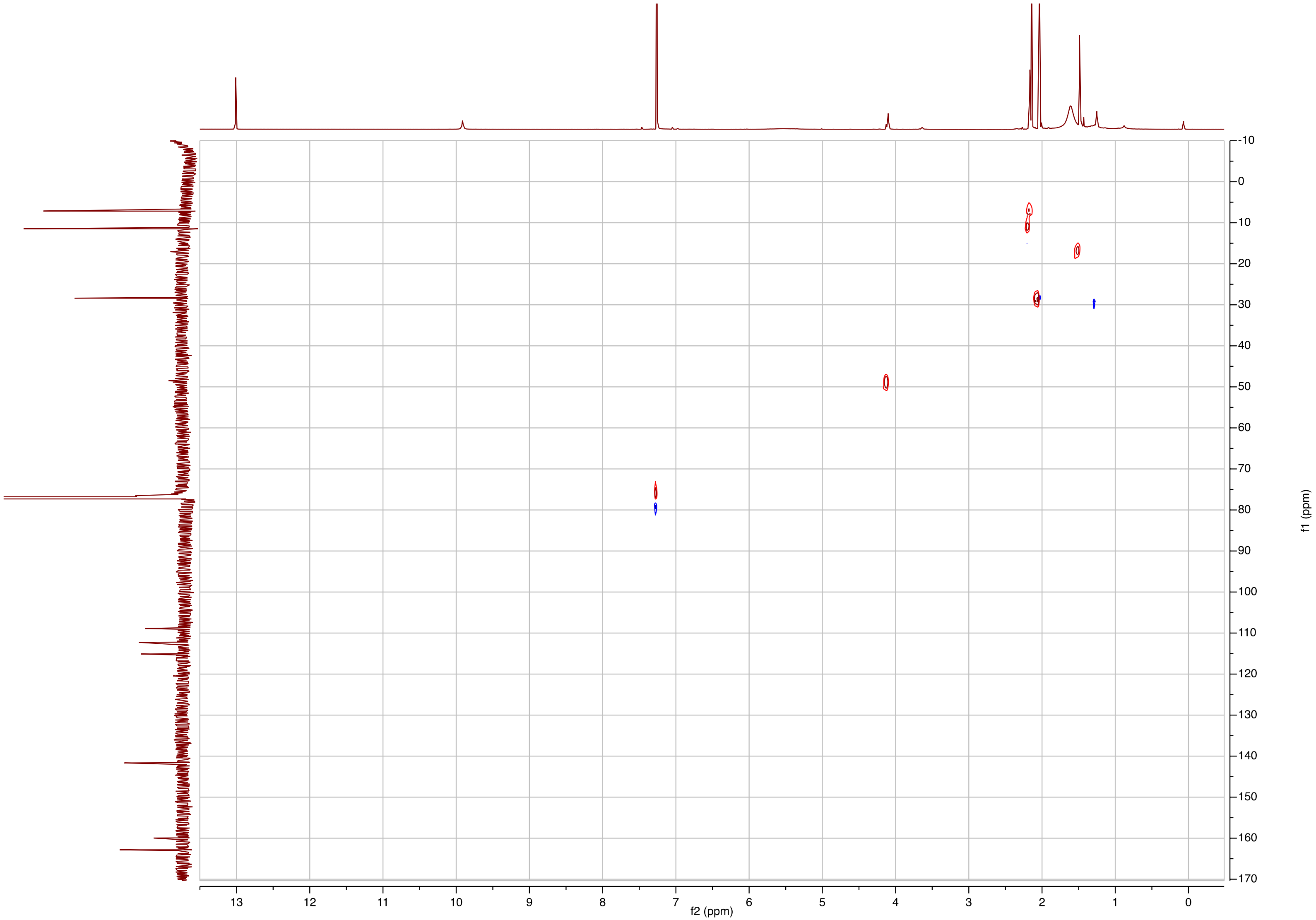

Compound 2 HSQC

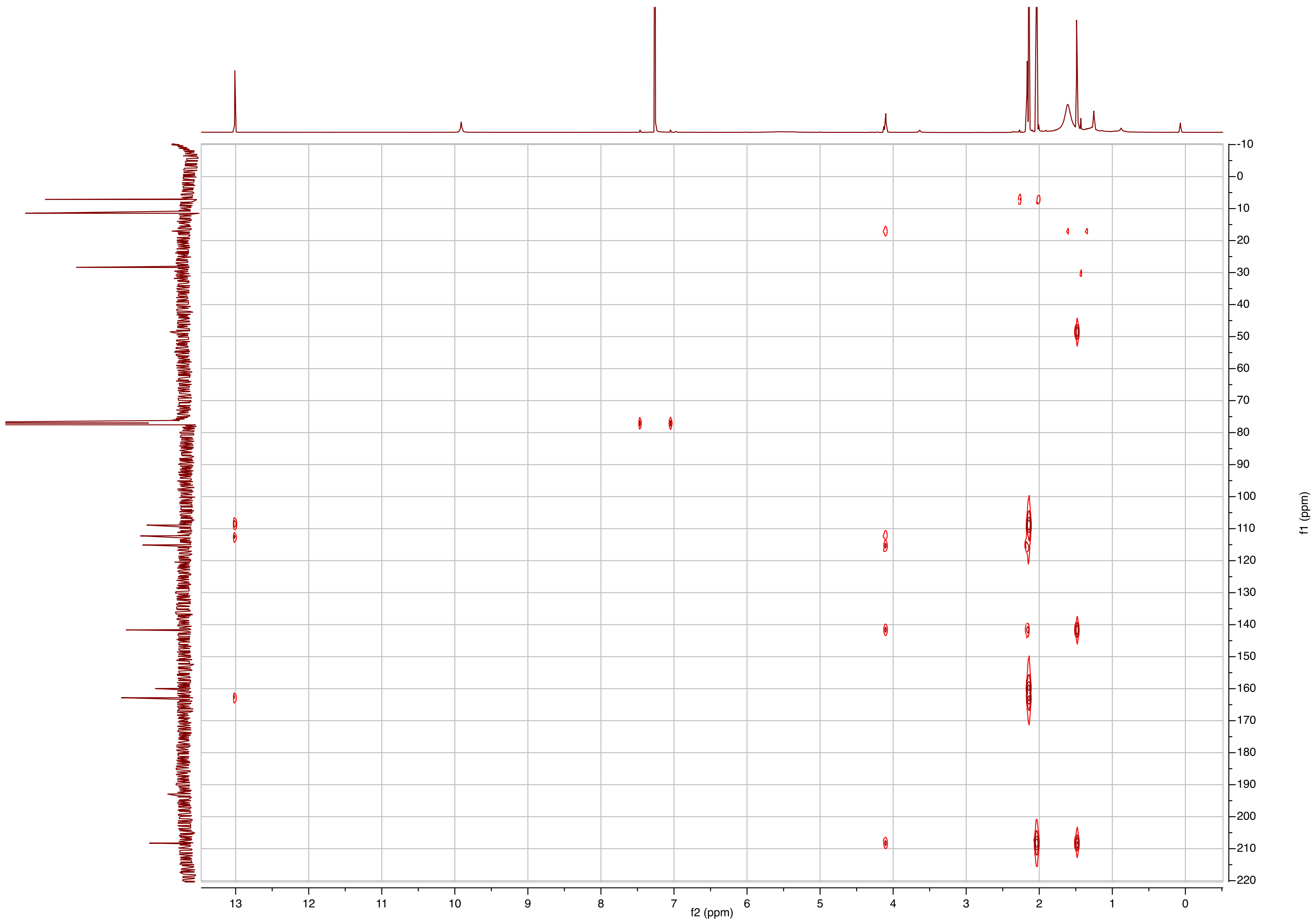

Compound 2 HMBC

7.3 NMR Spectra Compound 3

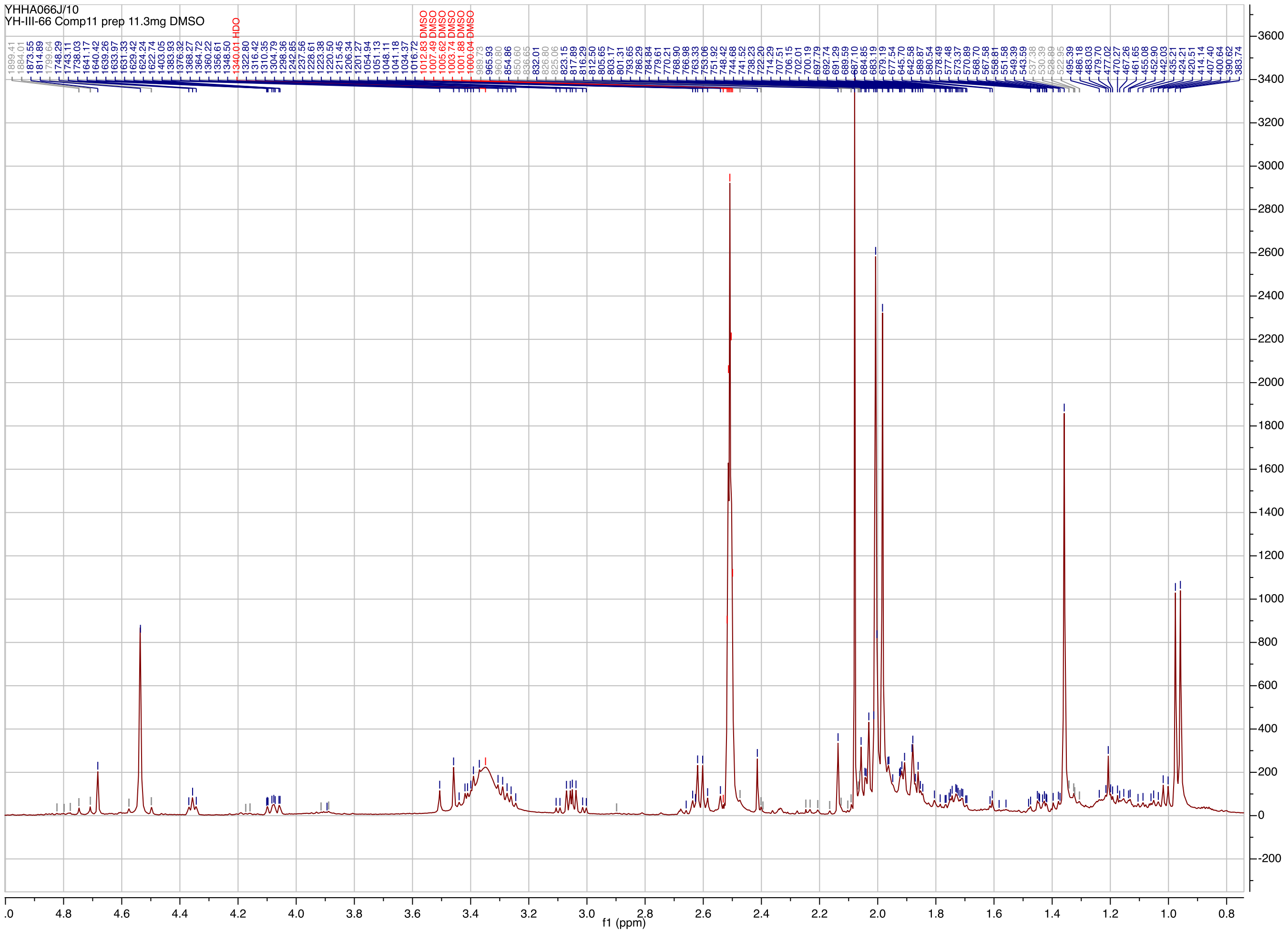

Compound 3 ^{1}H NMR DMSO d_6 400 MHz

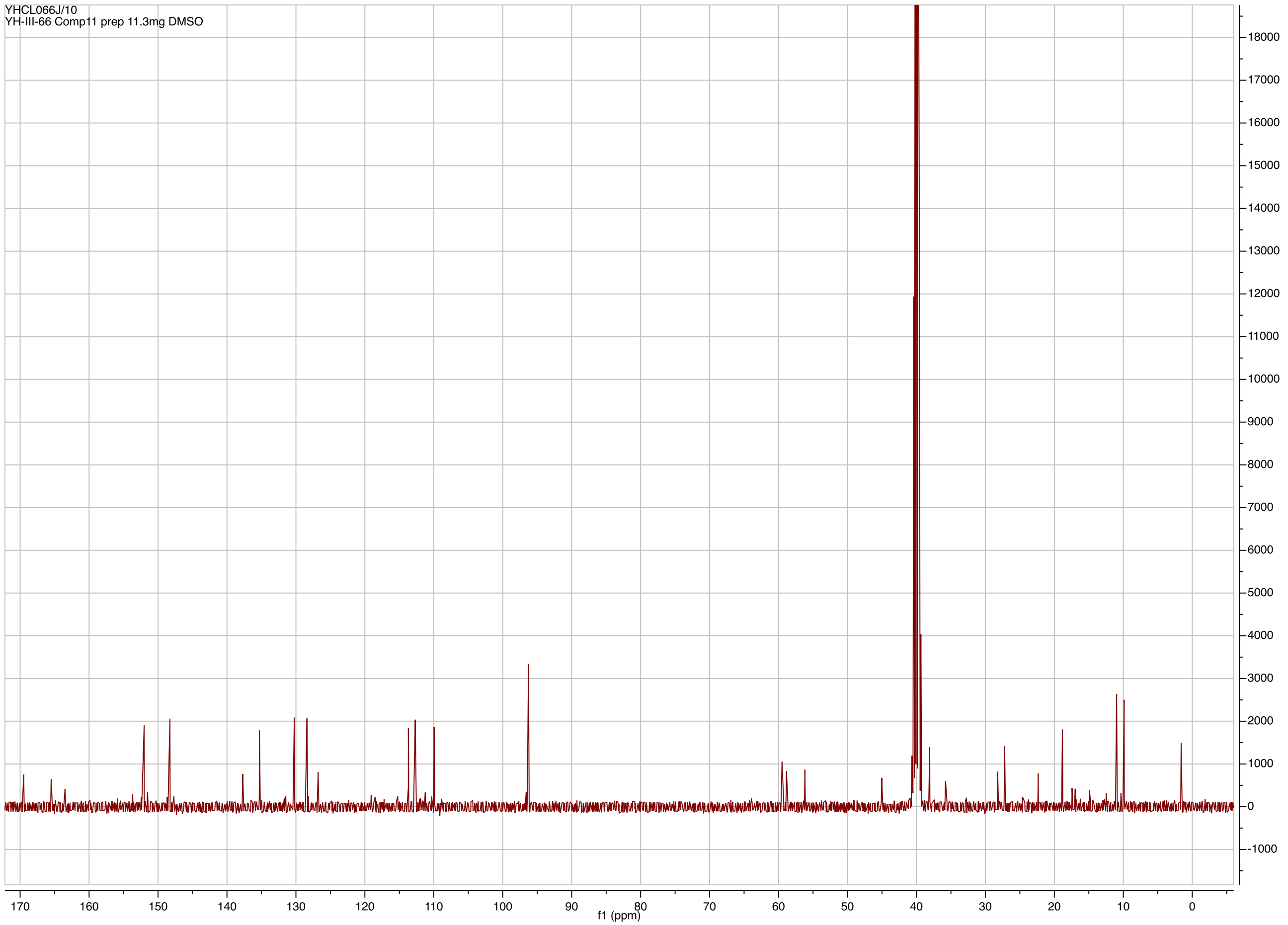

Compound 3 ^{1}H NMR DMSO d_6 100 MHz

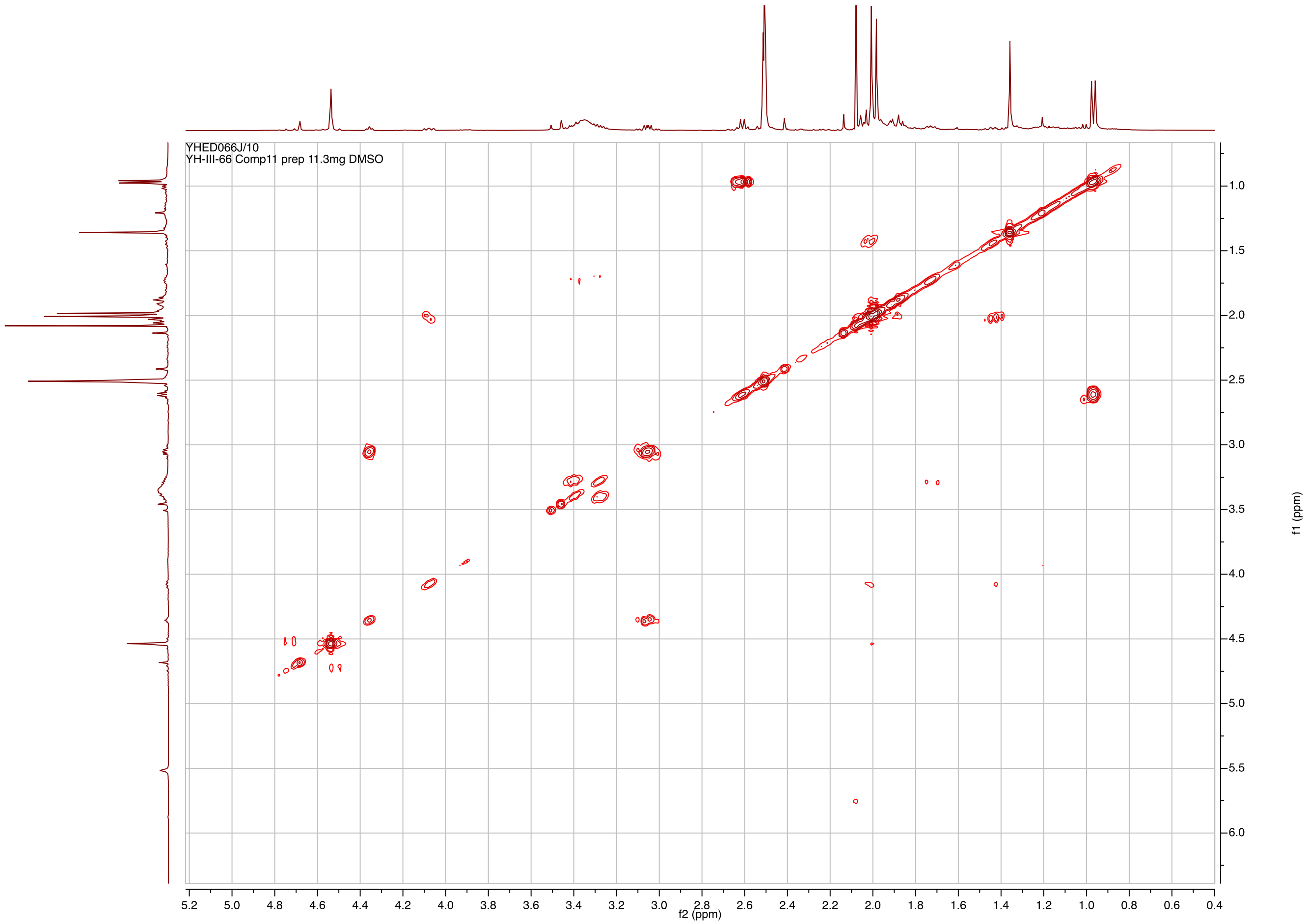

Compound 3 COSY

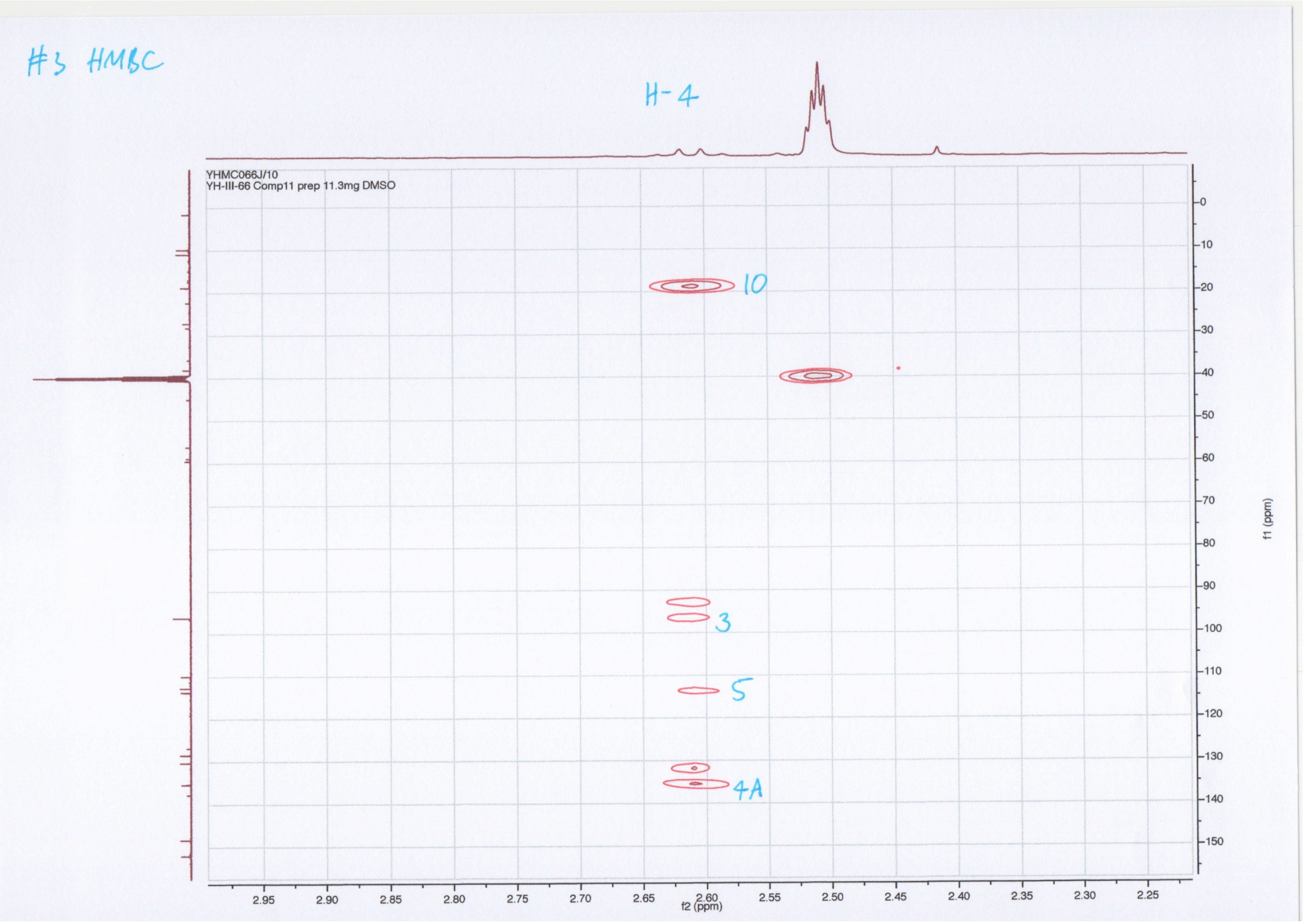

Compound 3 HMBC Expansion

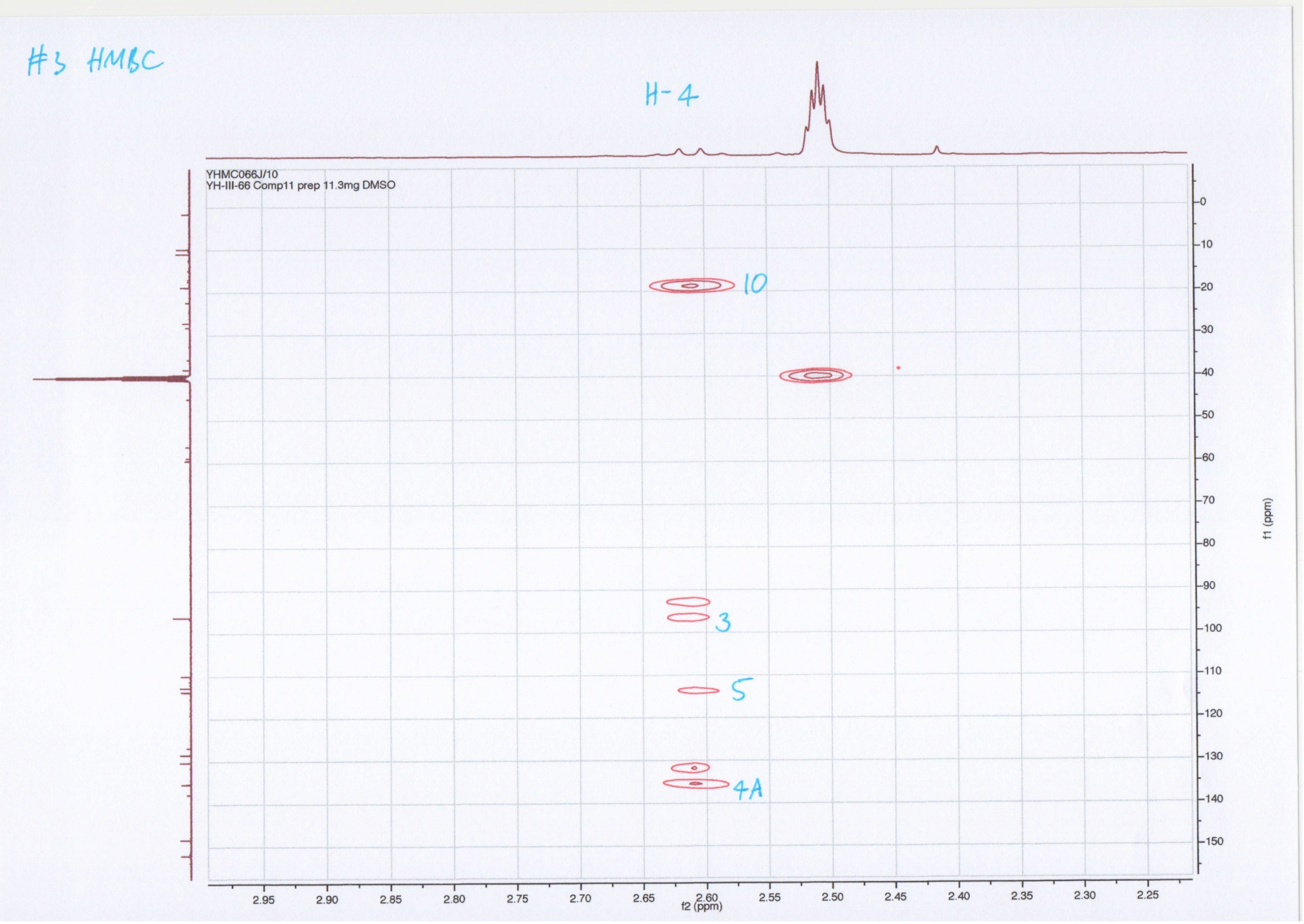

Compound 3 HMBC Expansion

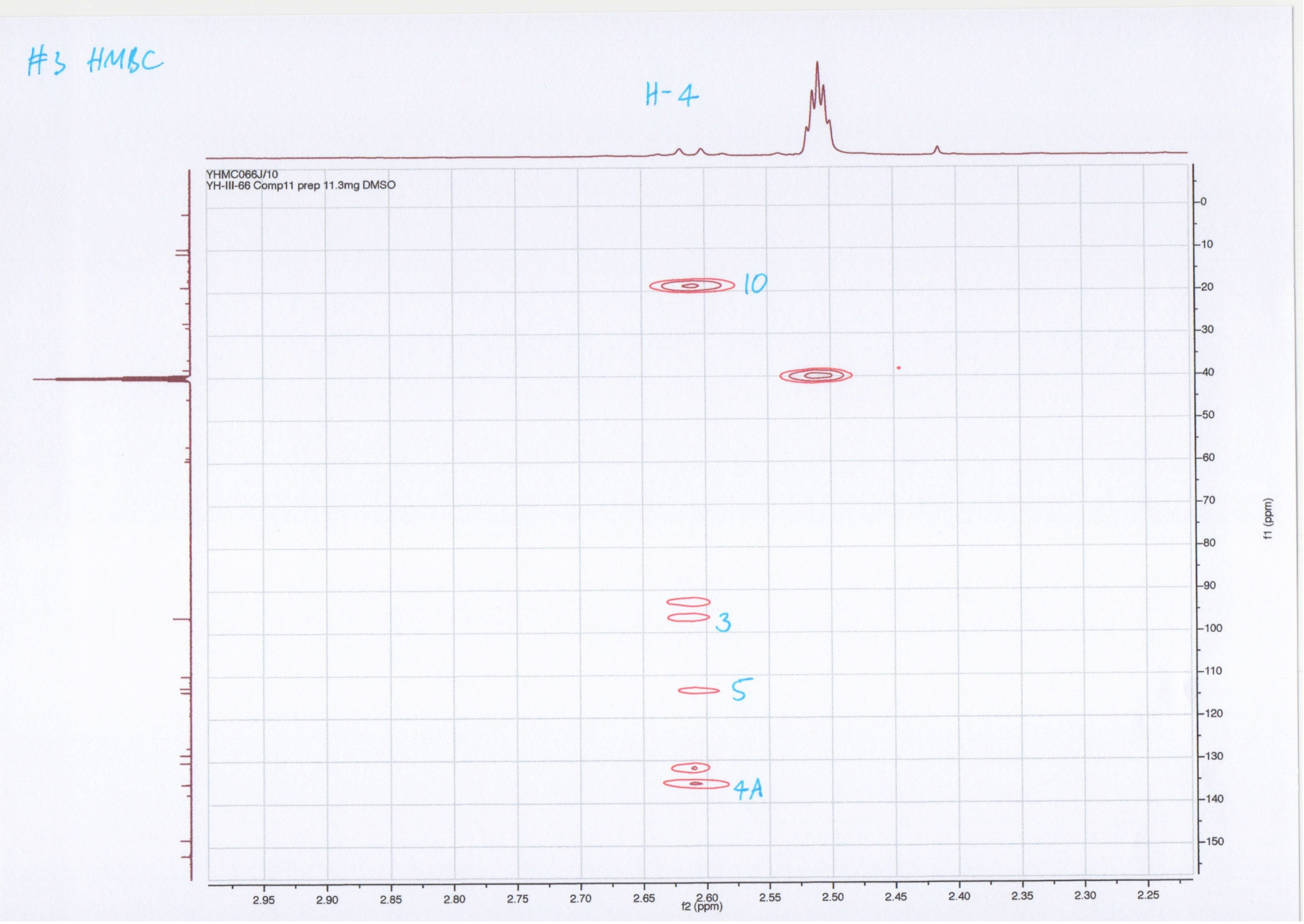

Compound 3 HMBC Expansion

7.4 NMR Spectra Compound 4

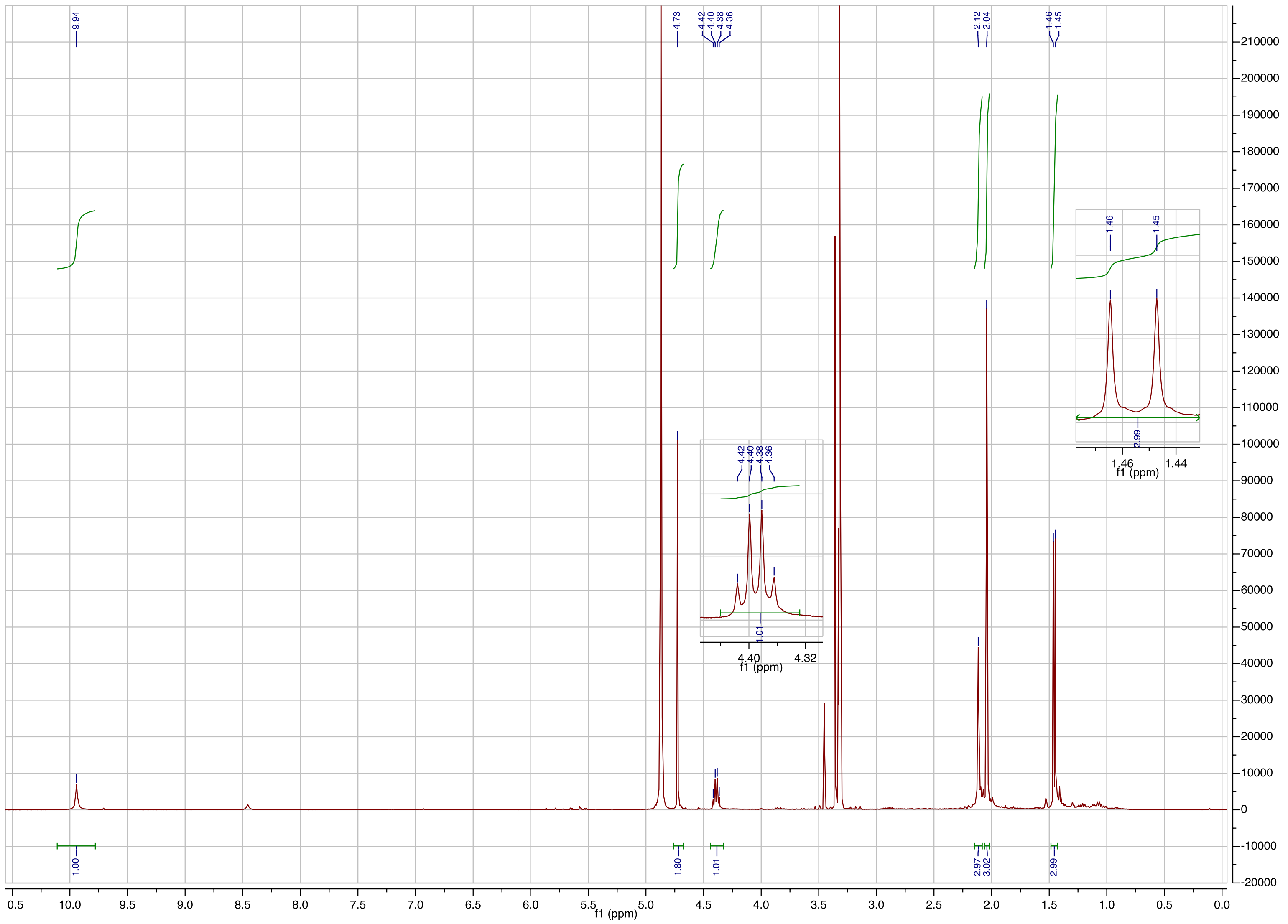

Compound 4 ^{1}H NMR CD_3OD 400 MHz

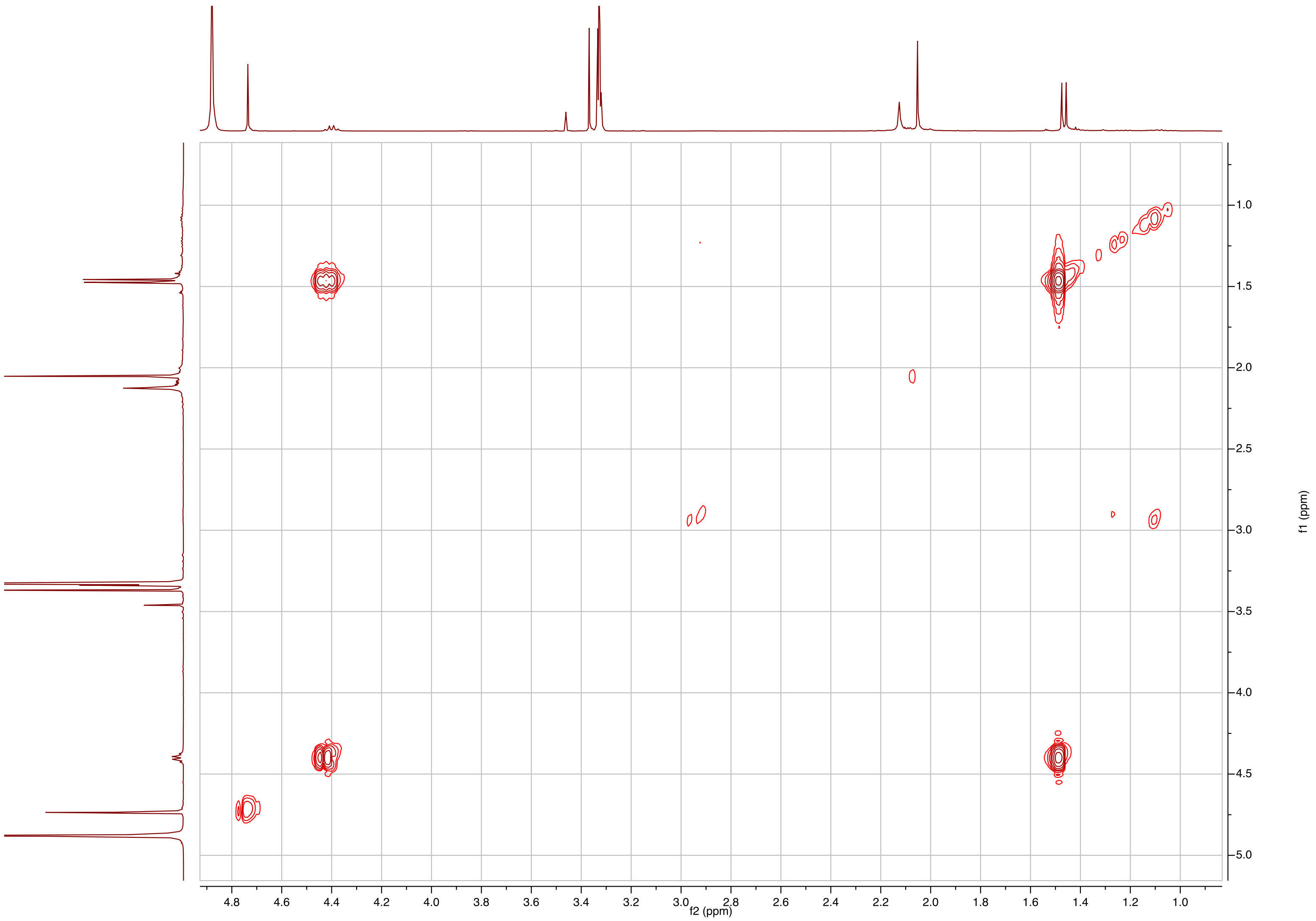

Compound 4 COSY

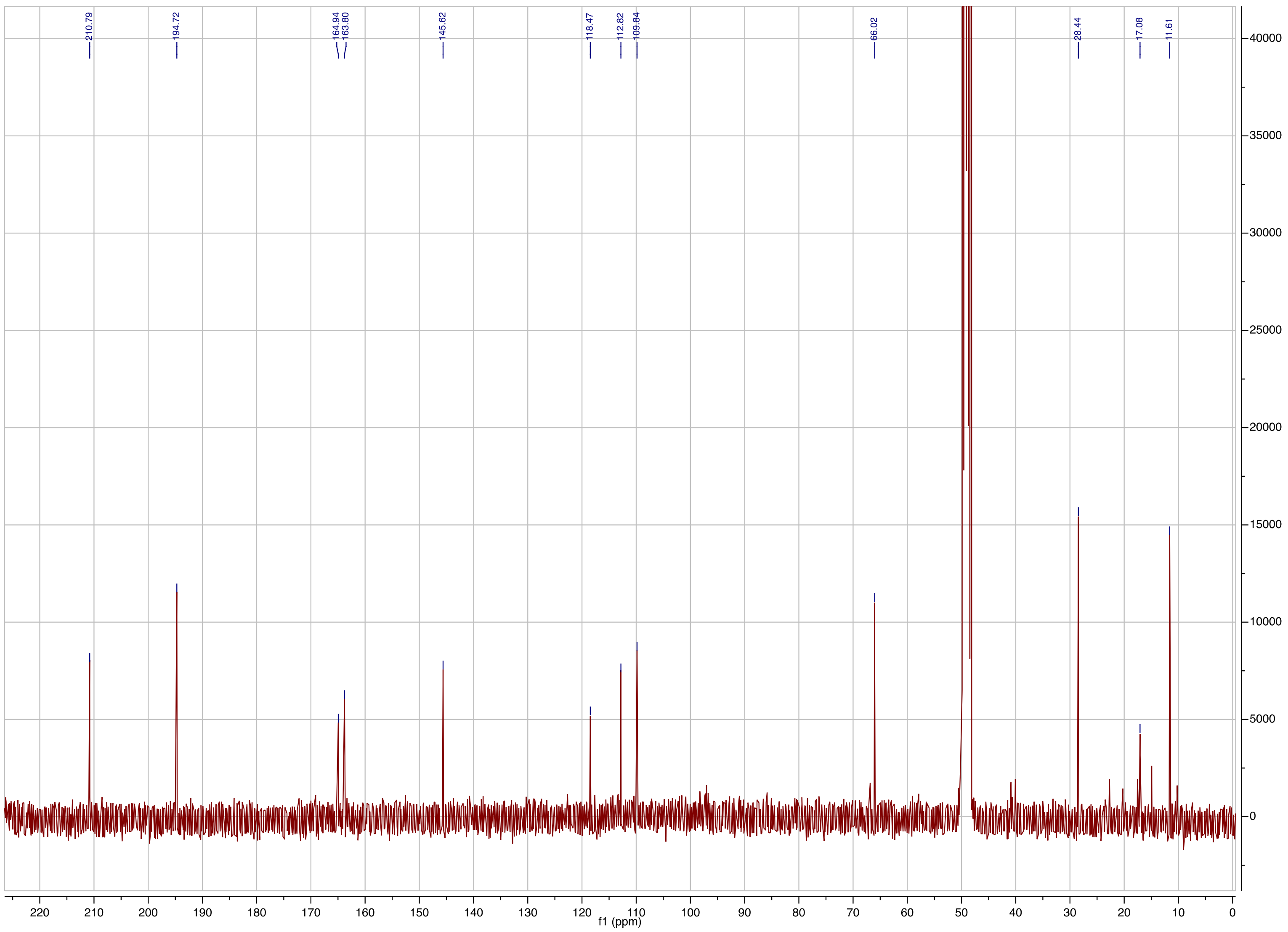

Compound 4 ^{13}C NMR CD_3OD 100 MHz

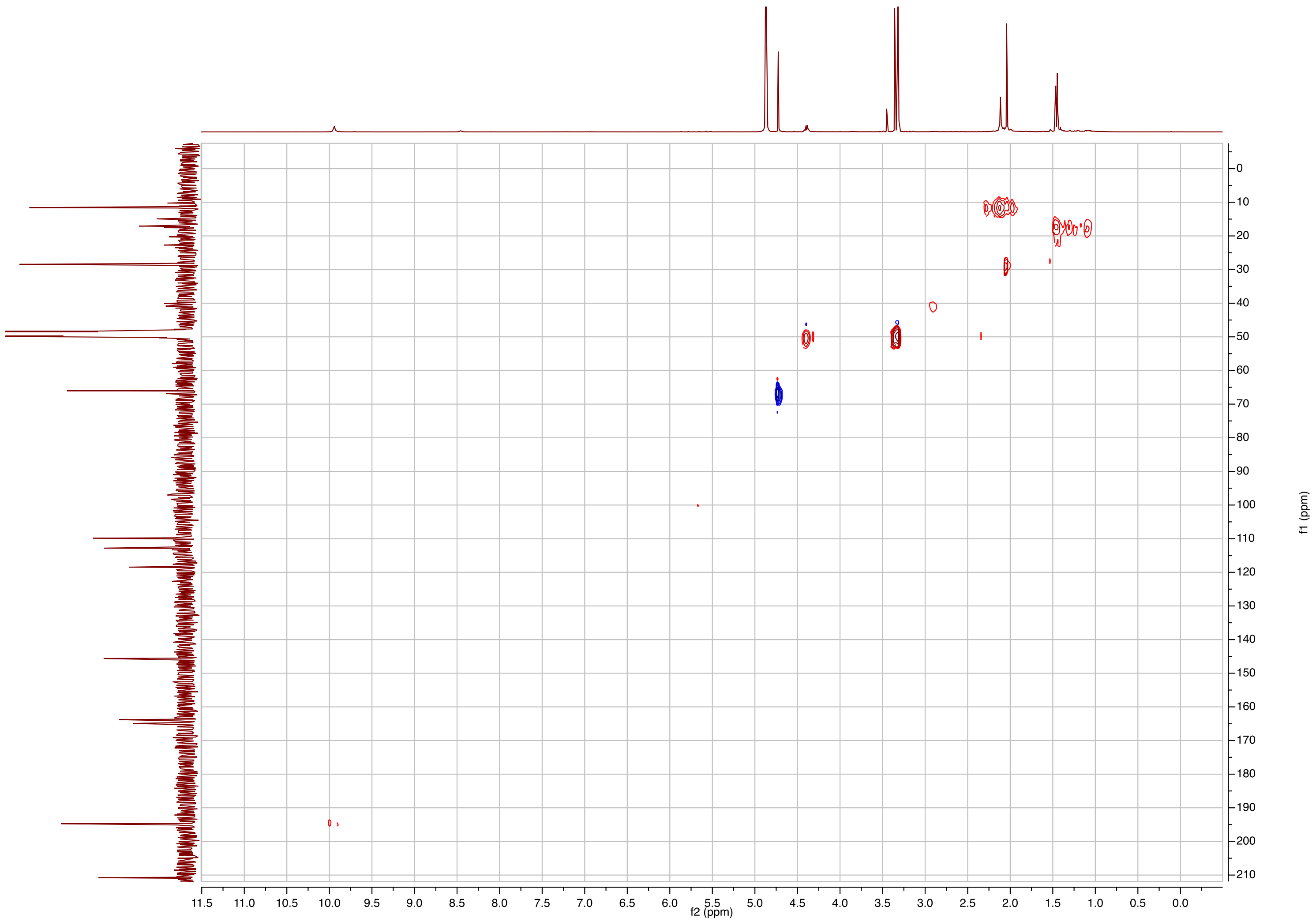

Compound 4 HSQC

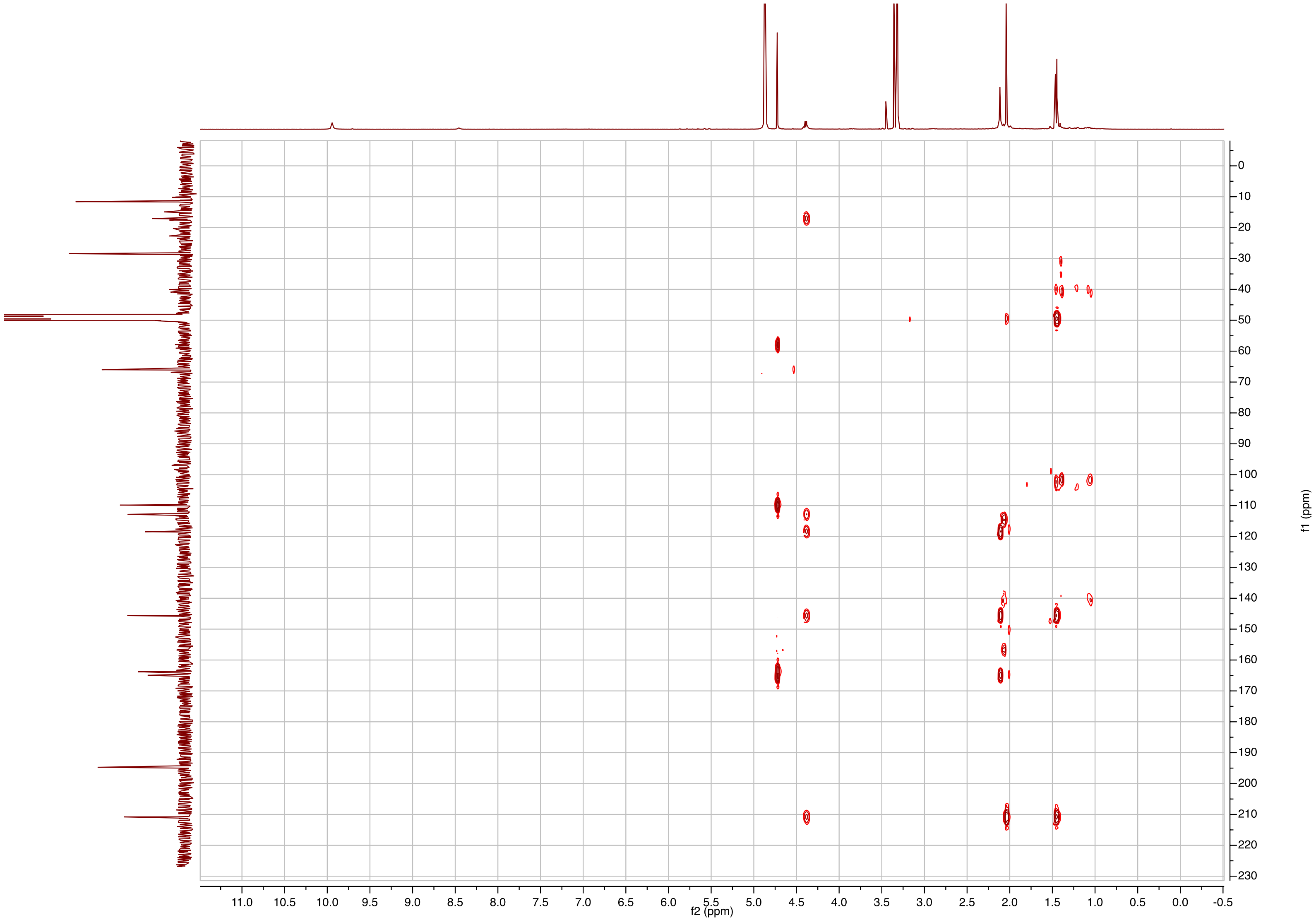

Compound 4 HMBC

7.5 NMR Spectra Compounds 5 + 6 (mix of diastereomers)

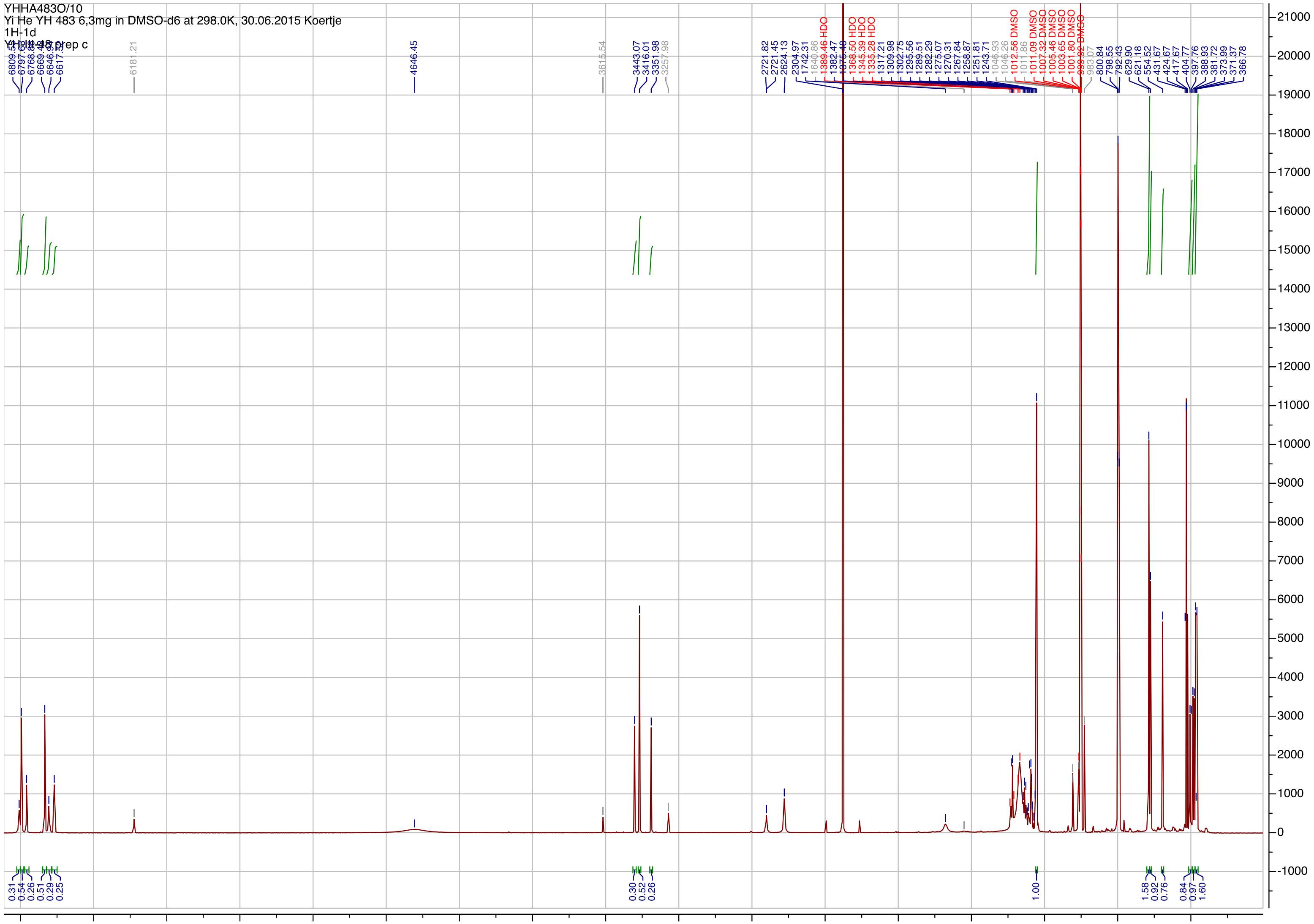

Compound 5+6 ^{1}H NMR, DMSO-d6 400 MHz

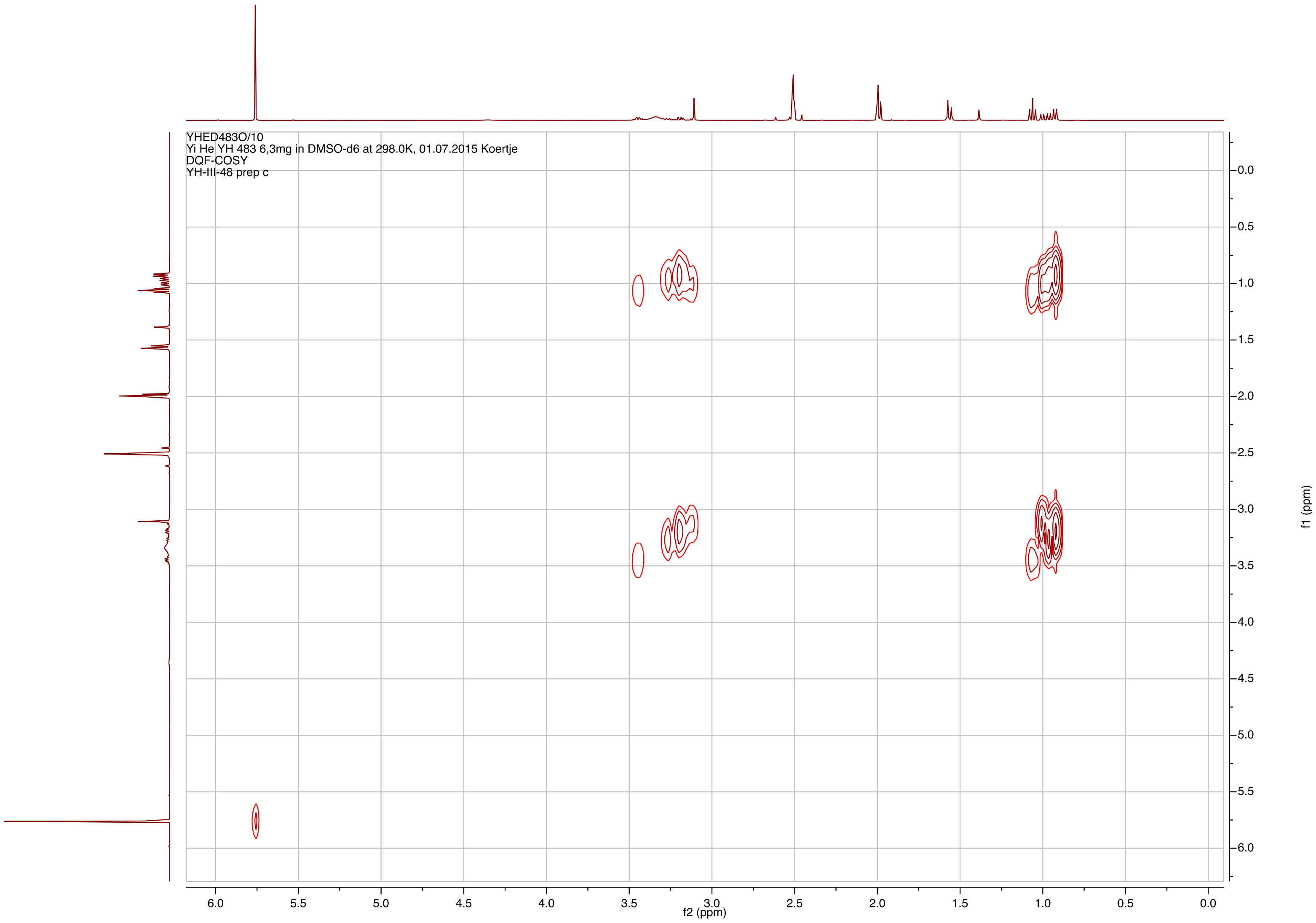

YHED483O/10
Yi He YH 483 6,3mg in DMSO-d6 at 298.0K, 01.07.2015 Koertje
DQF-COSY
YH-III-48 prep c
f2 (ppm)
f1 (ppm)

Compound 5+6

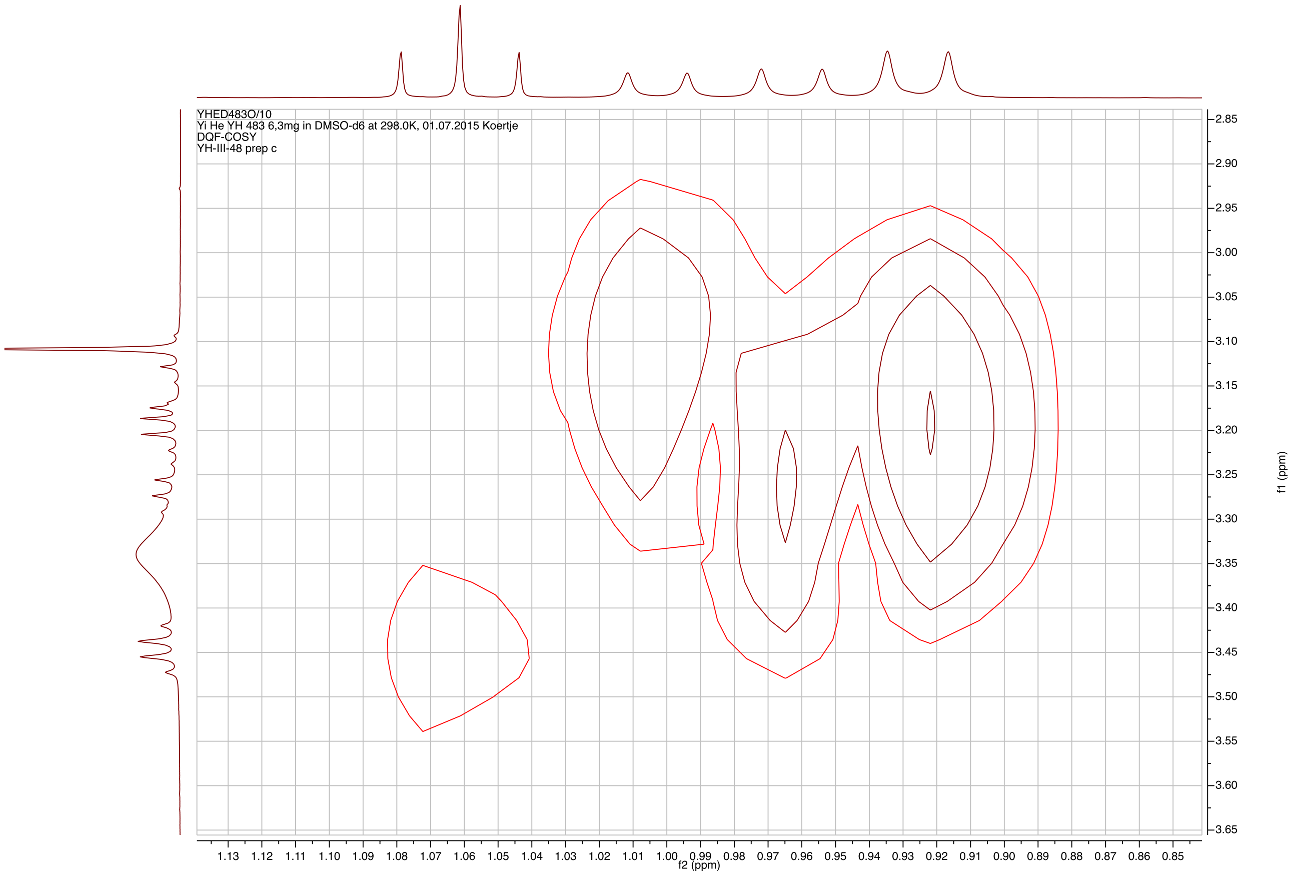

Mixture of 5 and diastereomers of 6.

Methyl-10 of **5**, d at 0.96 ppm; Methyl-10 of 6-major d at 0.92 ppm; Methyl-10 of 6-minor d at 1.00 ppm. Triplet at 1.06 ppm from contamination.

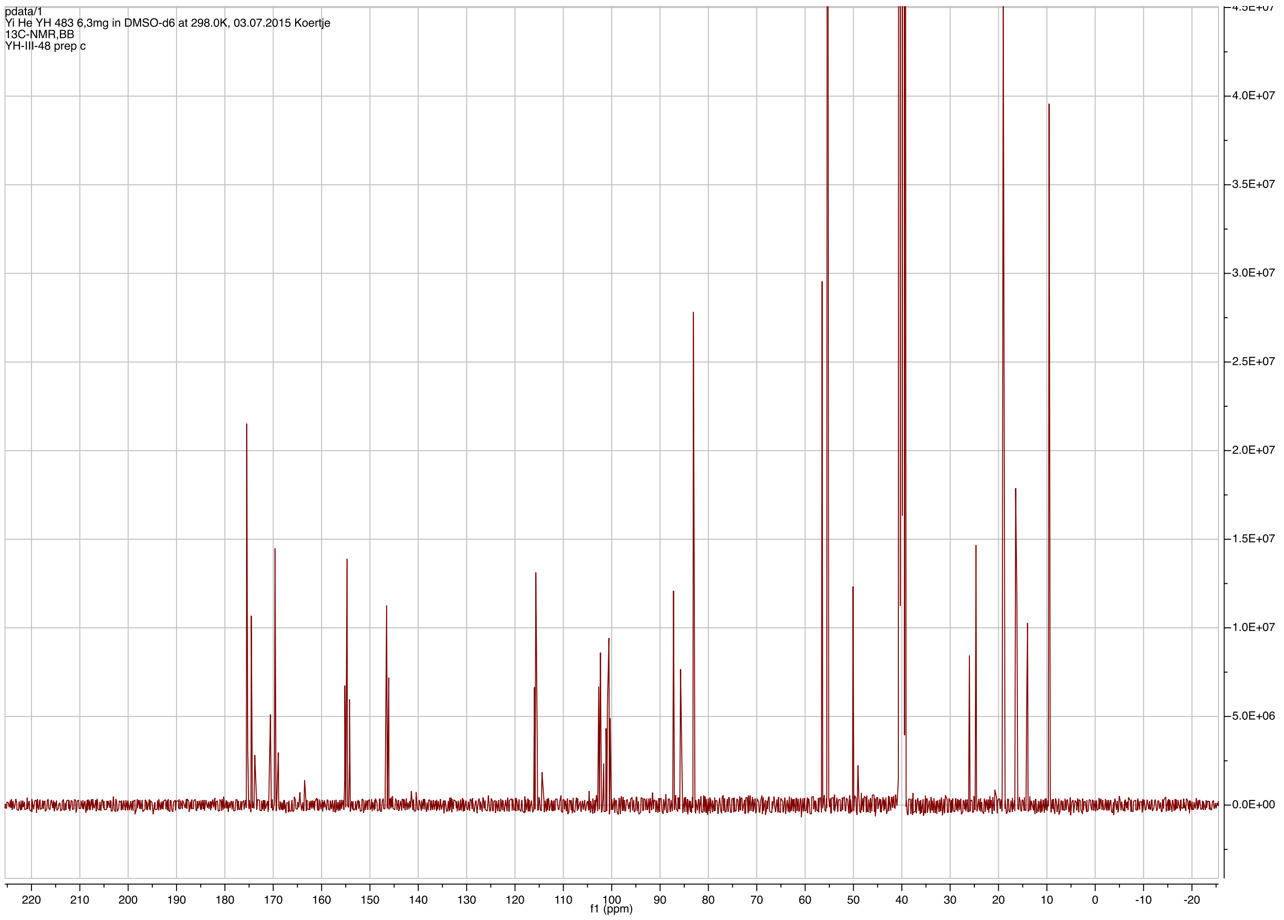

Compound 5+6 ^{13}C NMR DMSO-d6 100 MHz

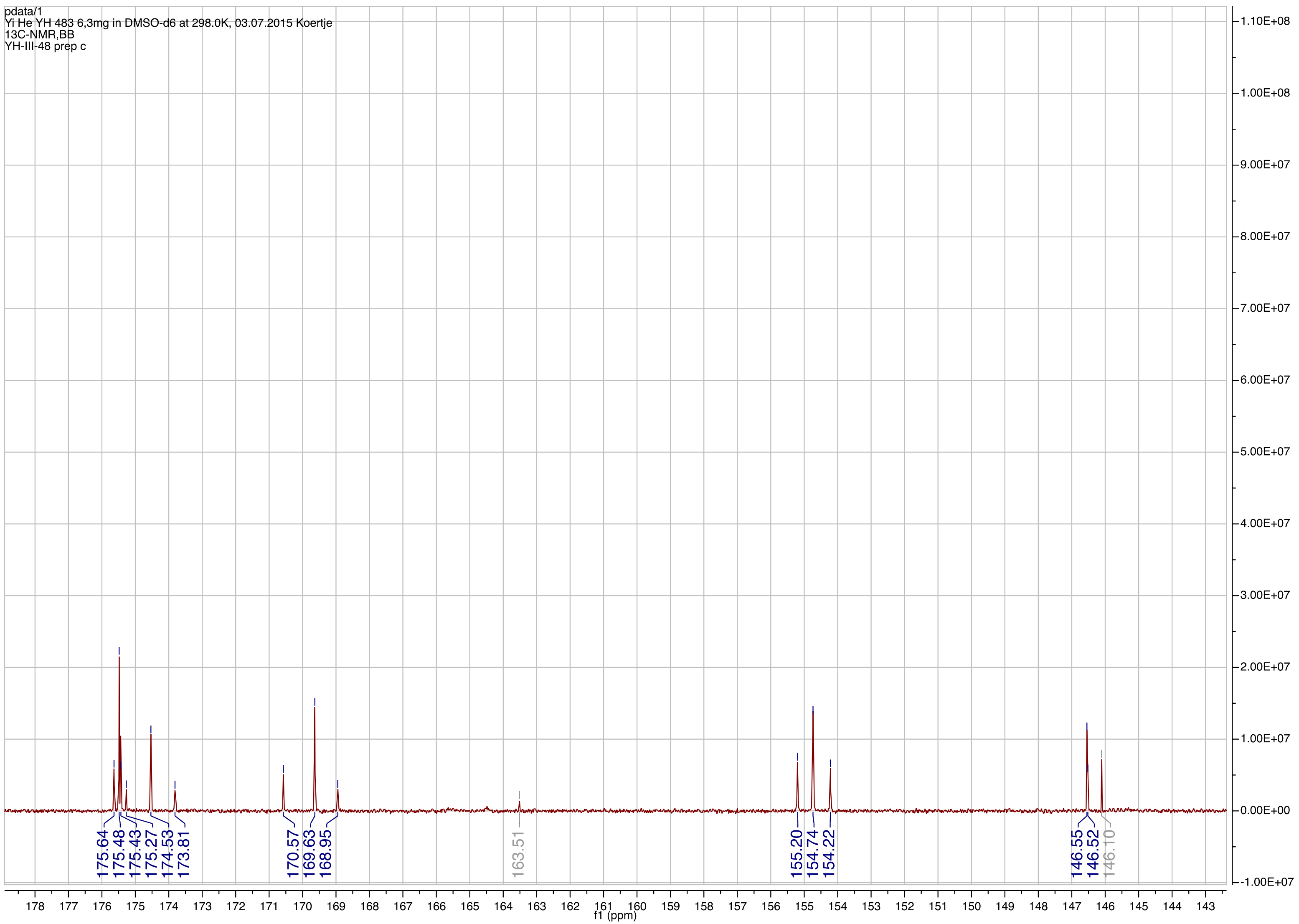

Compound 5+6 ^{13}C Expansion

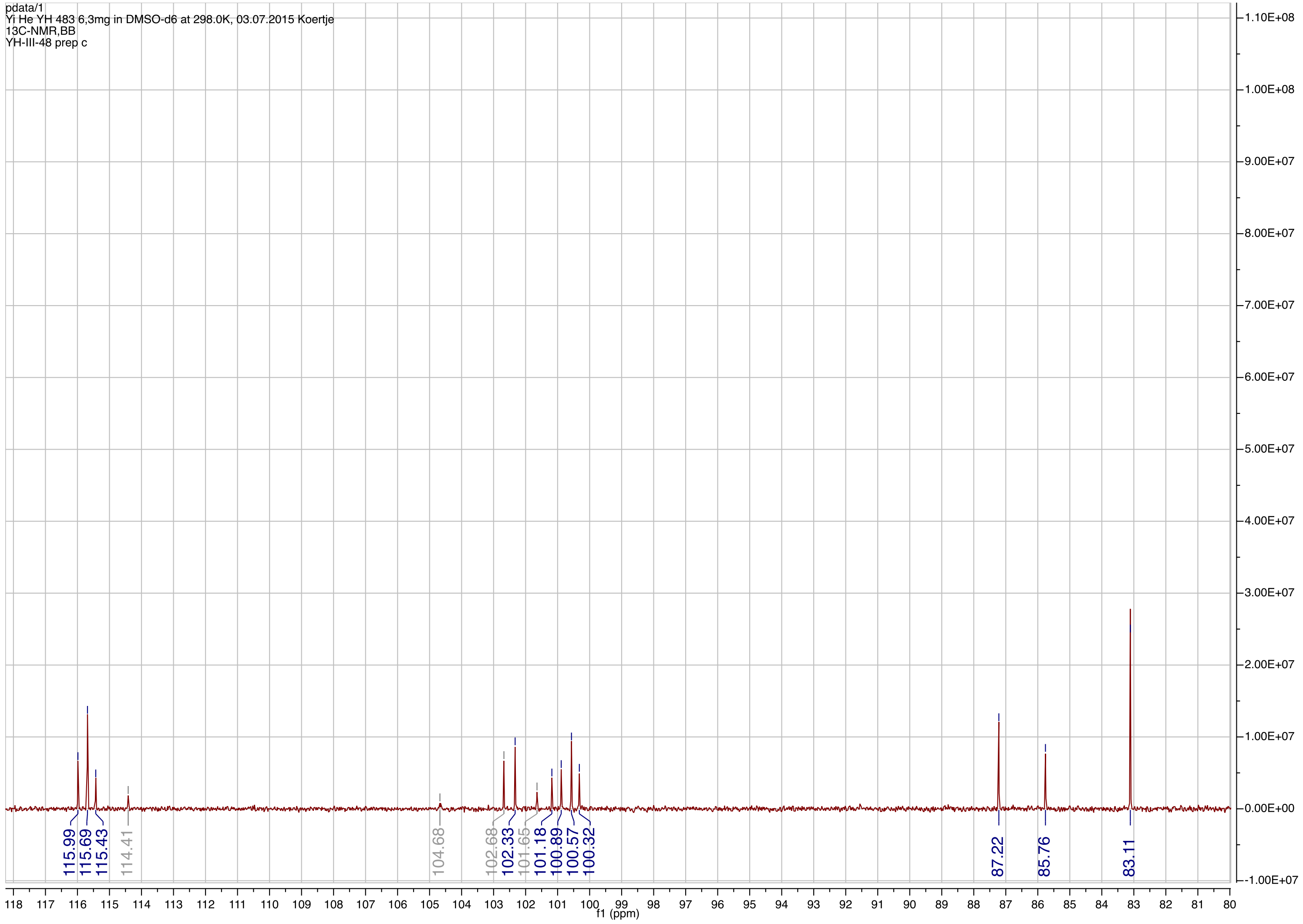

Compound 5+6 ^{13}C Expansion

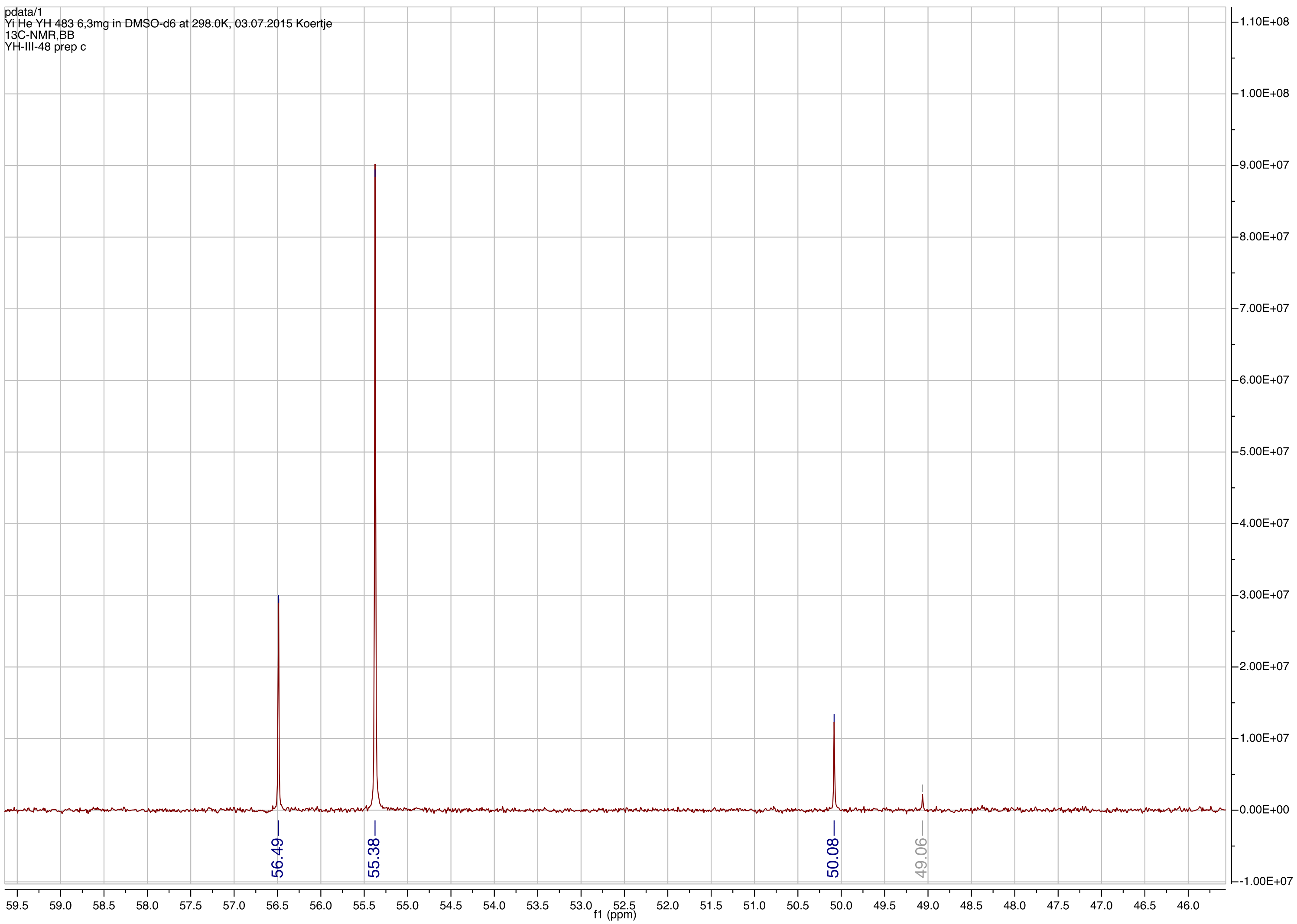

Compound 5+6 ^{13}C Expansion

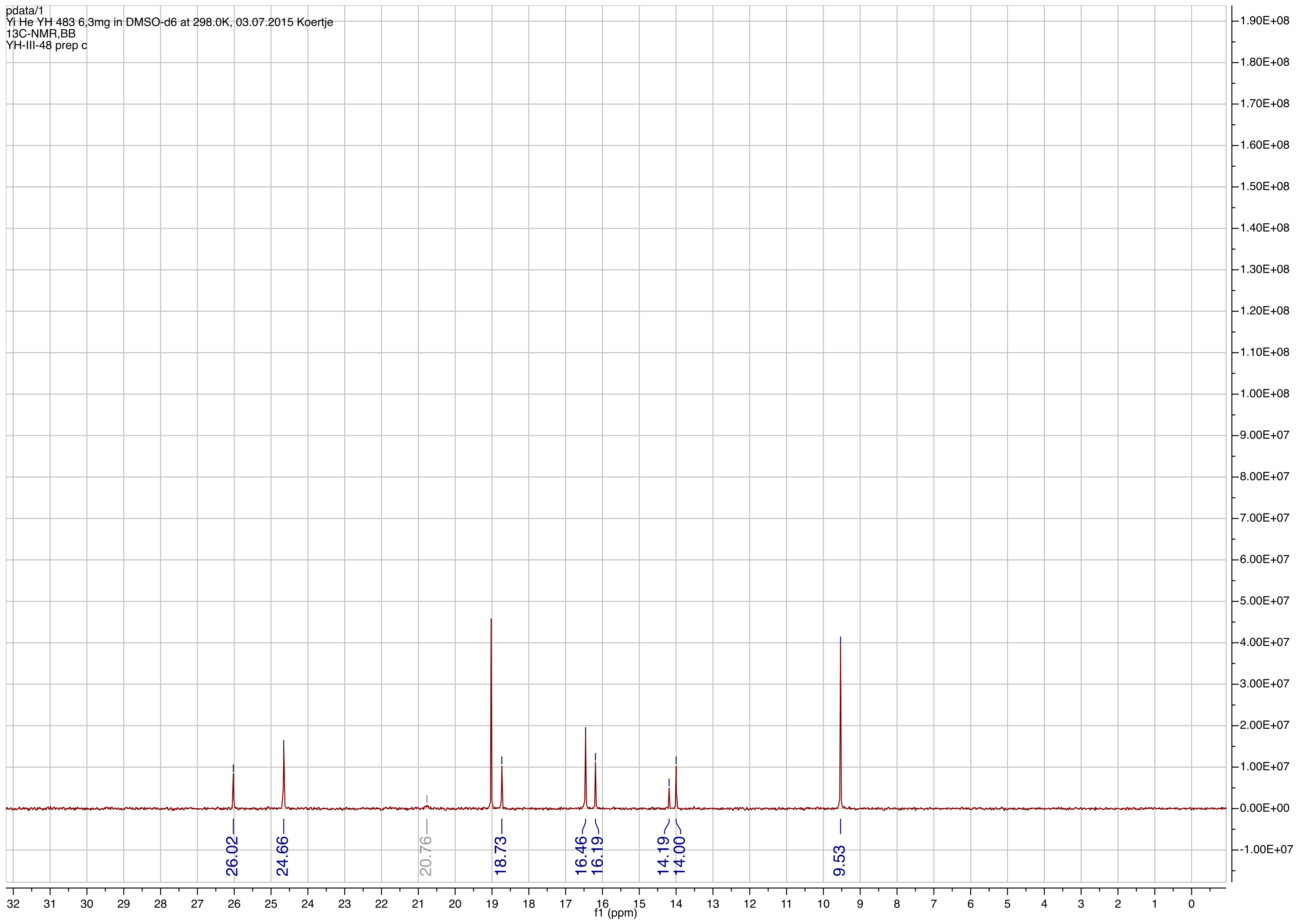

Compound 5+6 ^{13}C Expansion

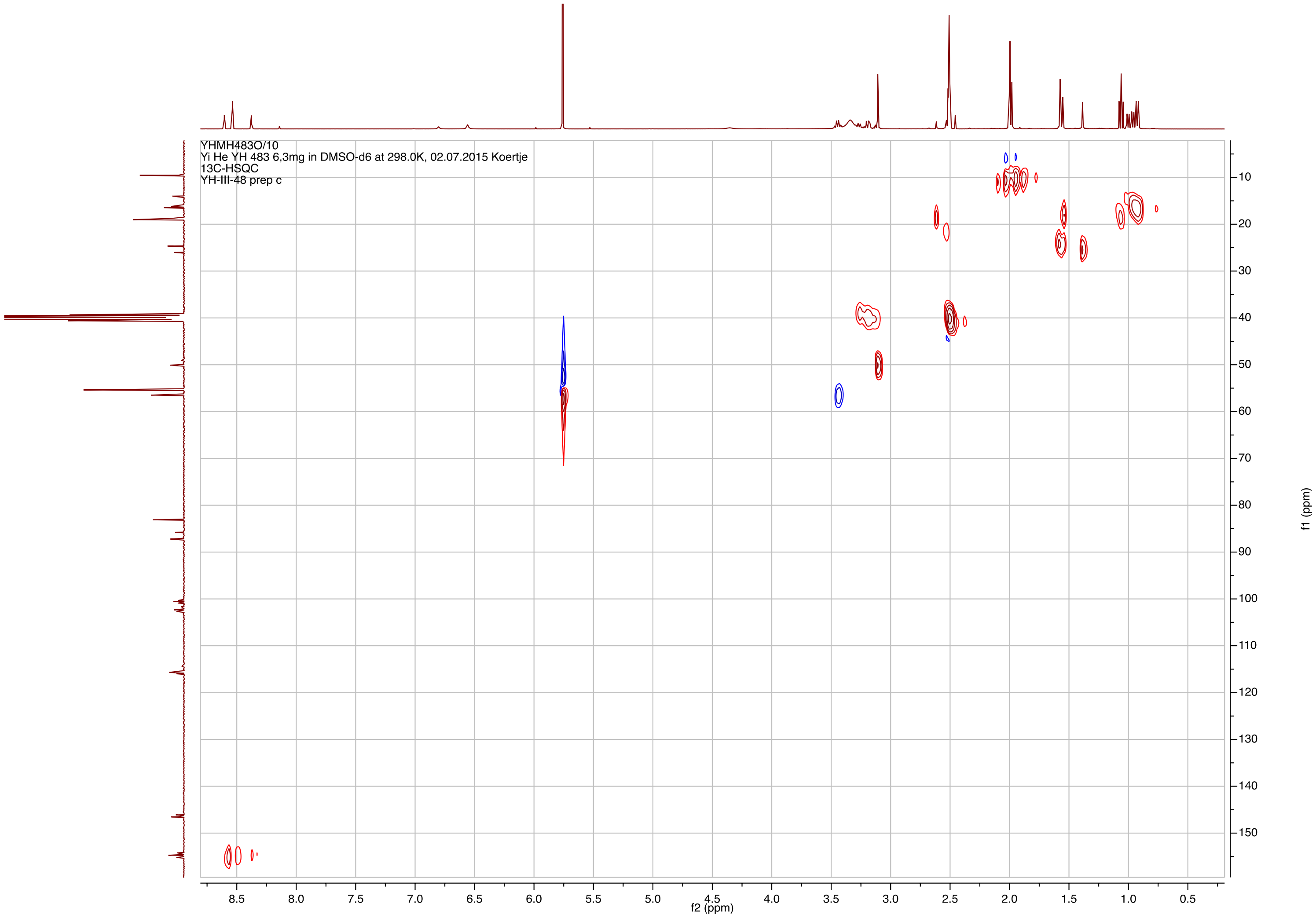

Compound 5+6 HSQC

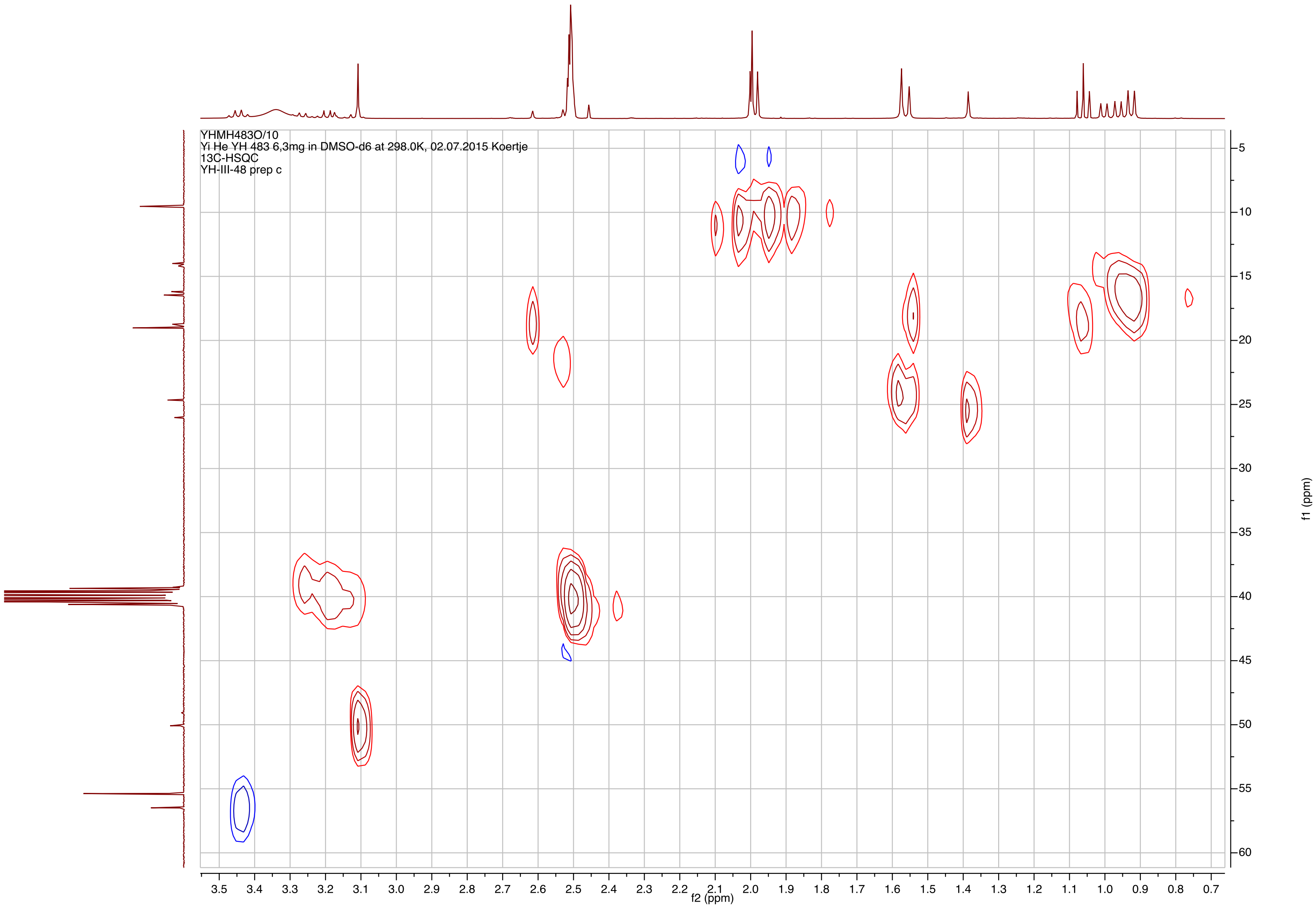

Compound 5+6 HSQC Expansion

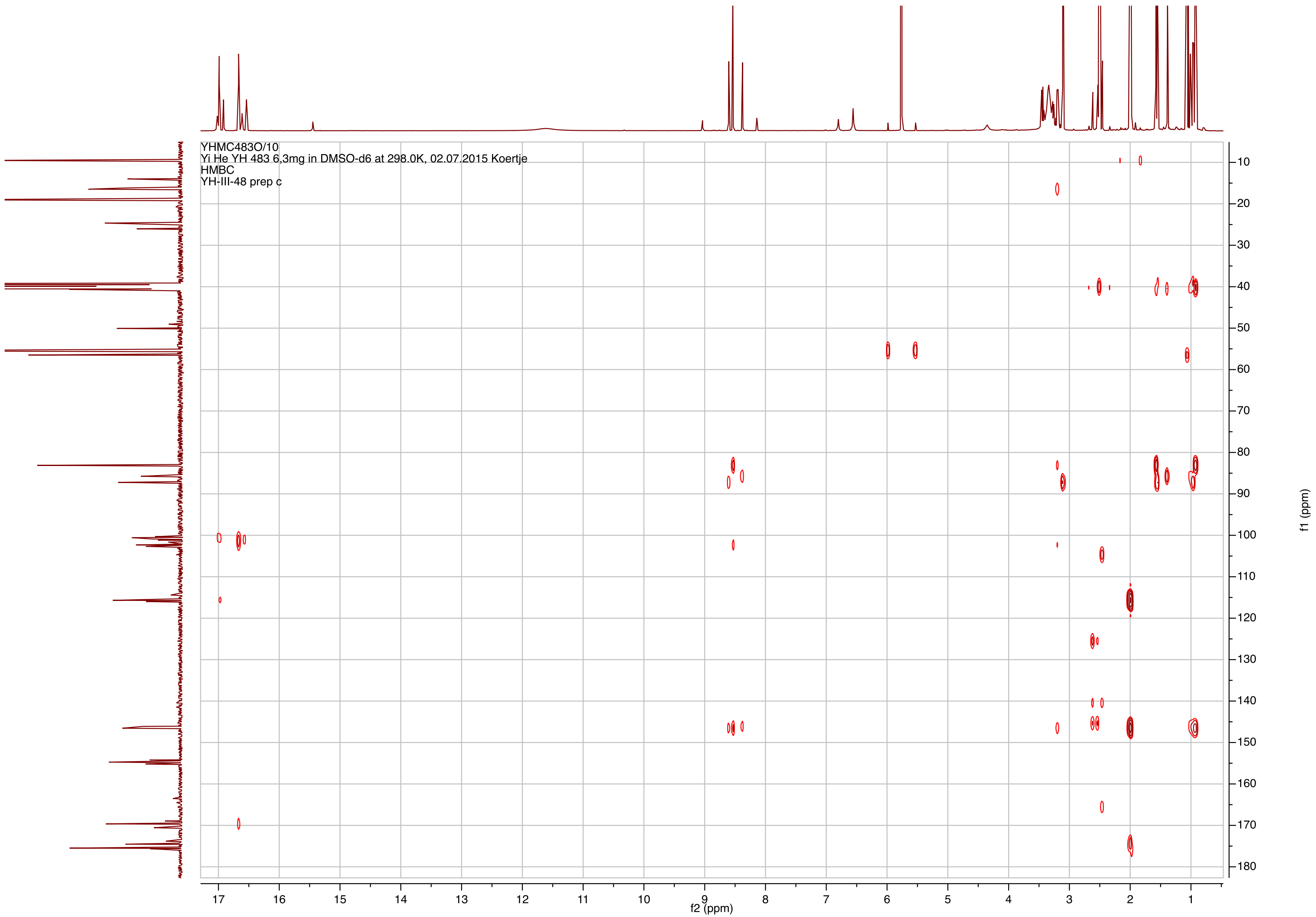

Compound 5+6 HMBC

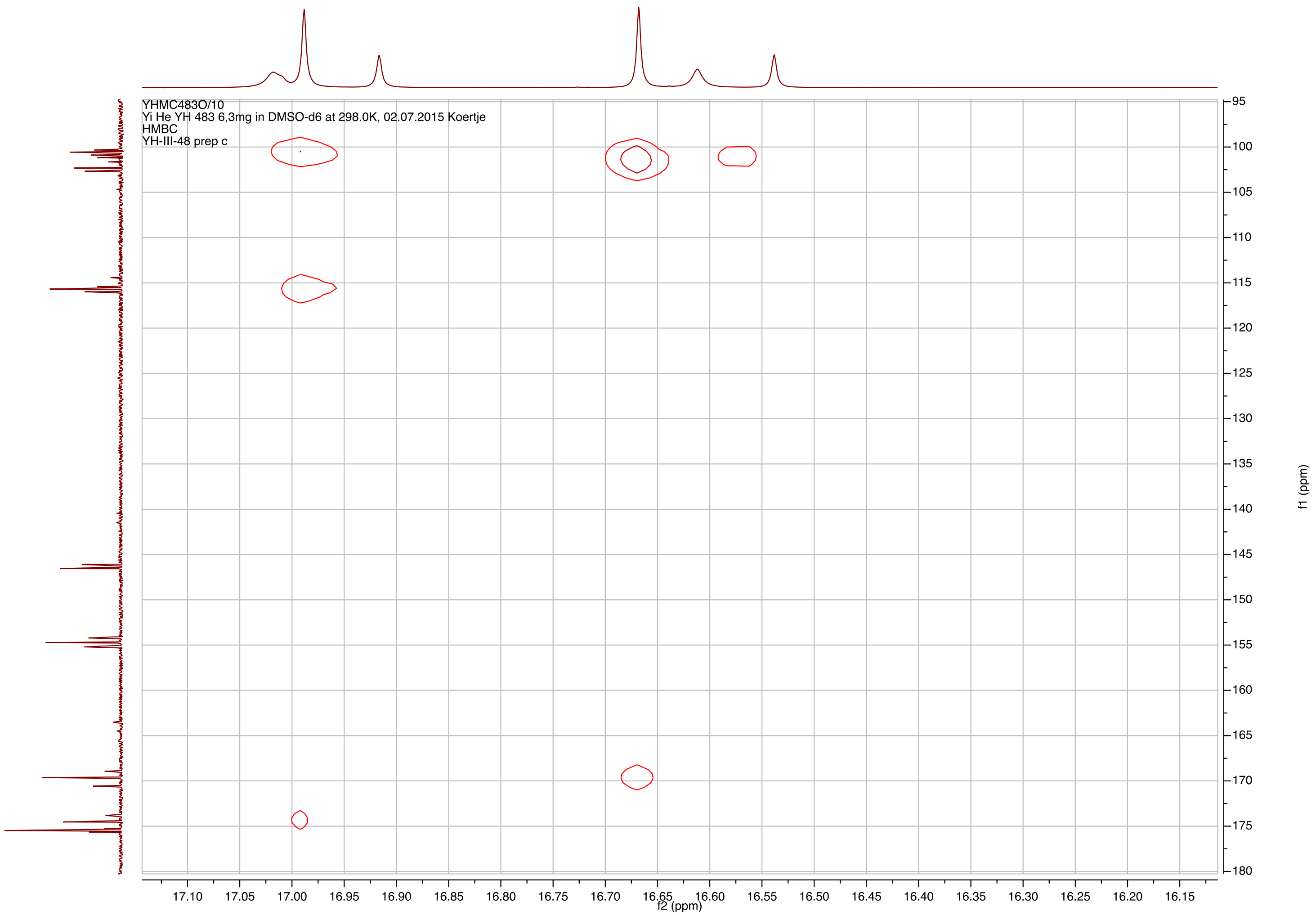

Compound 5+6 HMBC Expansion

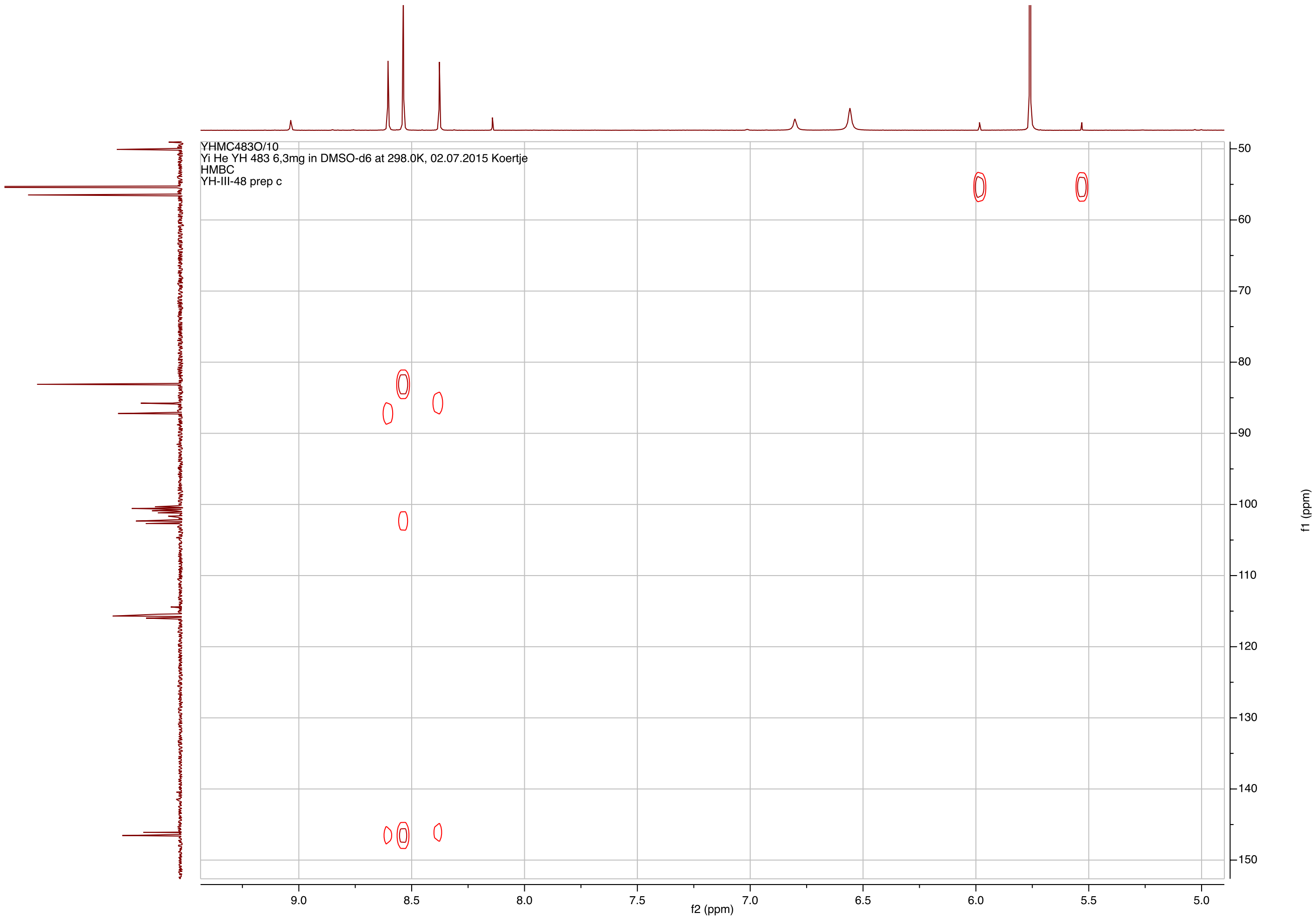

Compound 5+6 HMBC Expansion

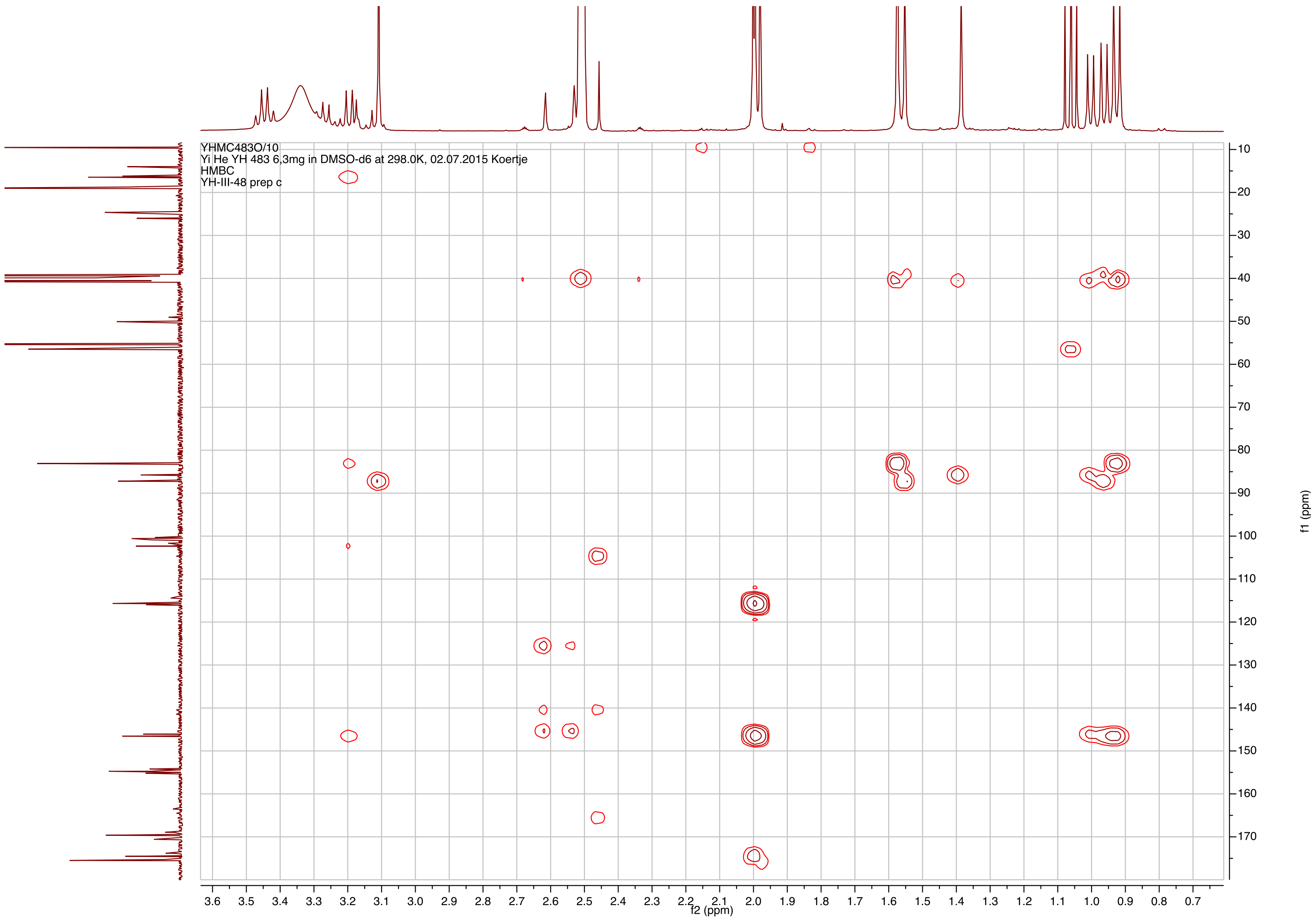

Compound 5+6 HMBC Expansion

7.7 NMR Spectra Compound 7 (mixture of diastereomers) + 6 and other degradation products.

7 was isolated by rapid mass-directed reverse-phase purification. Solvent was evaporated, NMR solvent (DMSO-d_6) was added and NMR spectra gained as quickly as possible. However spectra always contain traces of **6** and other degradation products. **7** was identified as the only component of the mixture consistent with the measured HRMS data indicating a molecular formula of $C_{15}H_{19}NO_6$. In particular HMBC correlations confirmed the skeleton as the same as **6**, and also the location of the CH_2CH_2OH group (HMBC from H-1 to C-13).

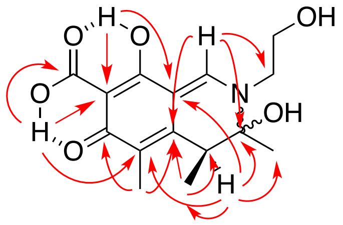

HMBC H➛C Correlations

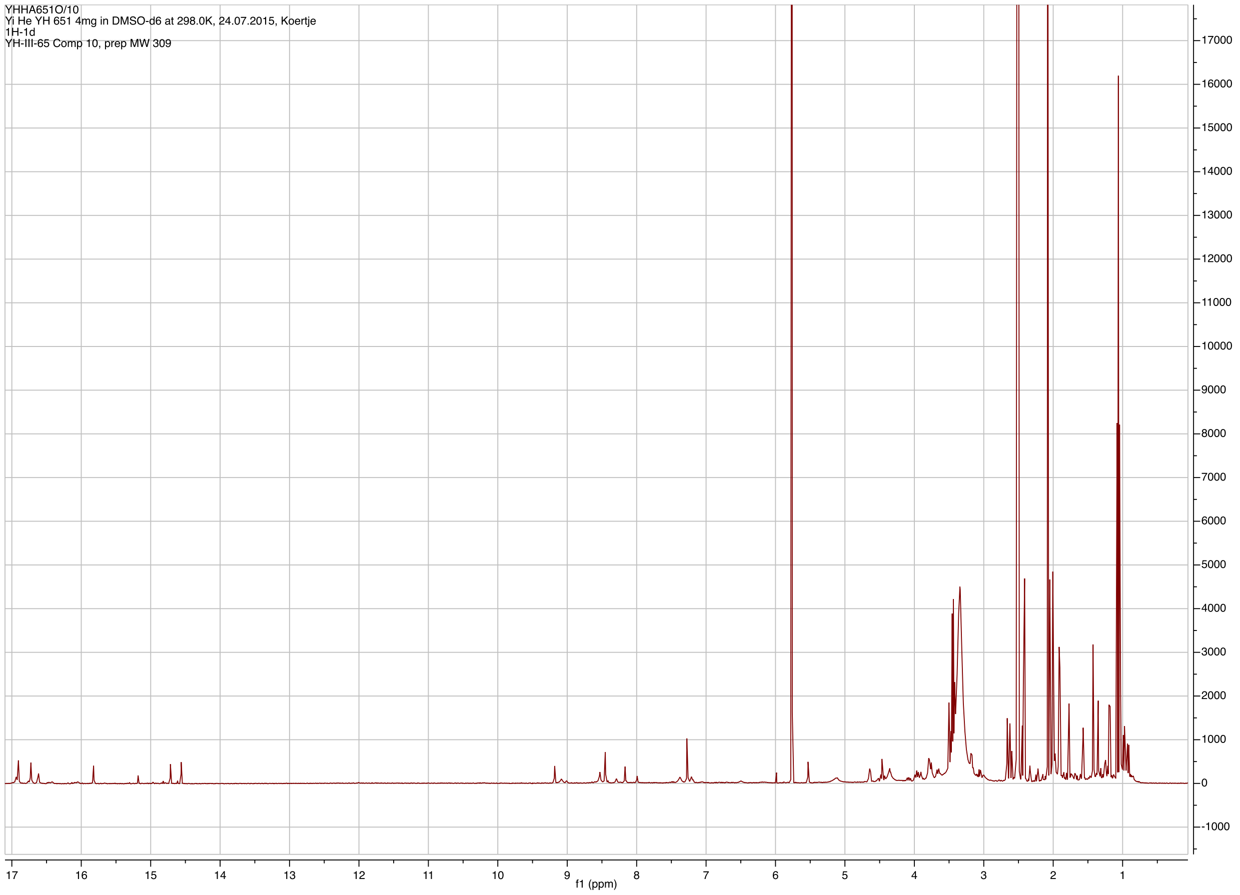

Compound 7 ^{1}H NMR DMSO-d_6 400 MHz

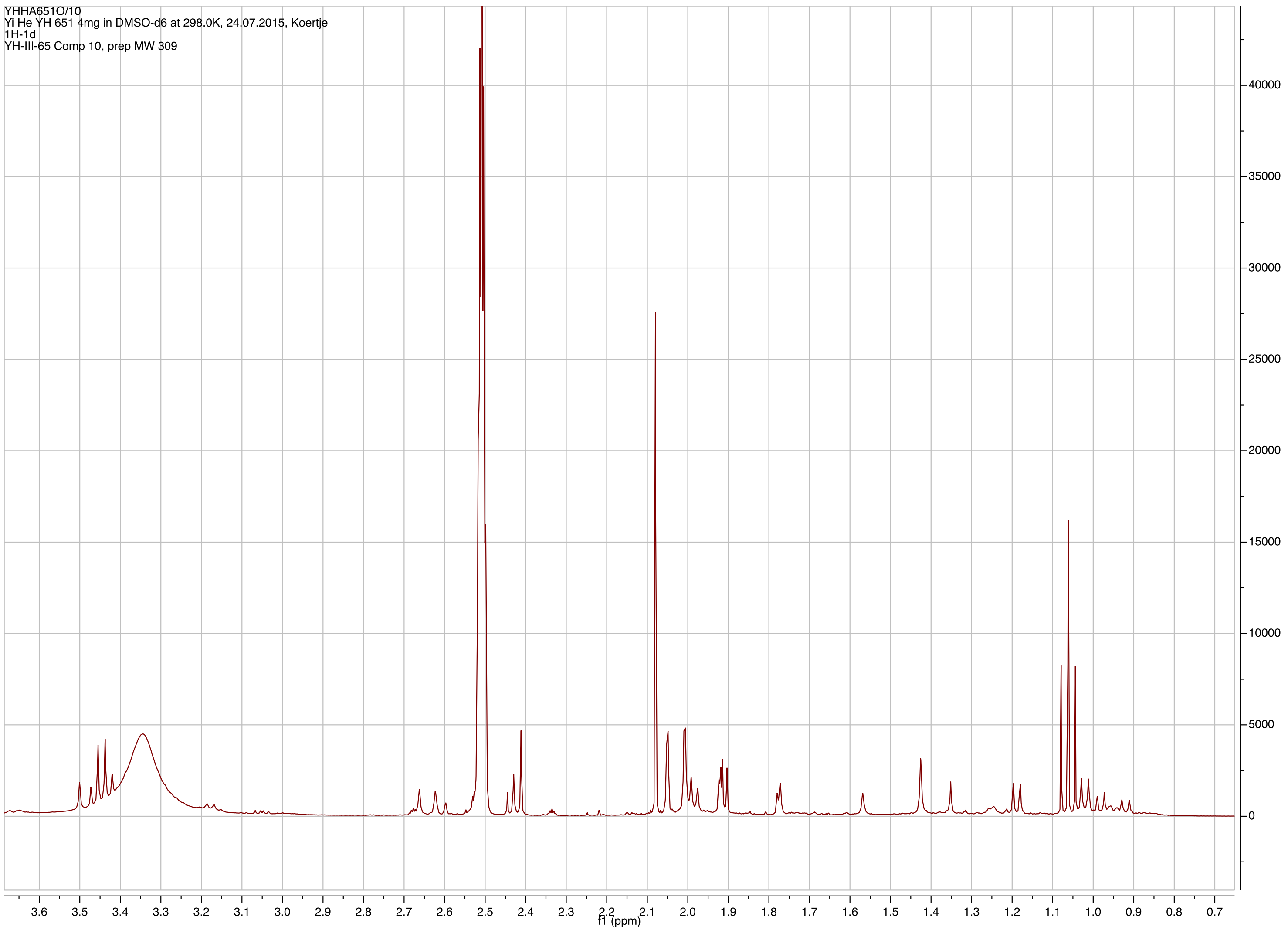

Compound 7 ^{1}H NMR

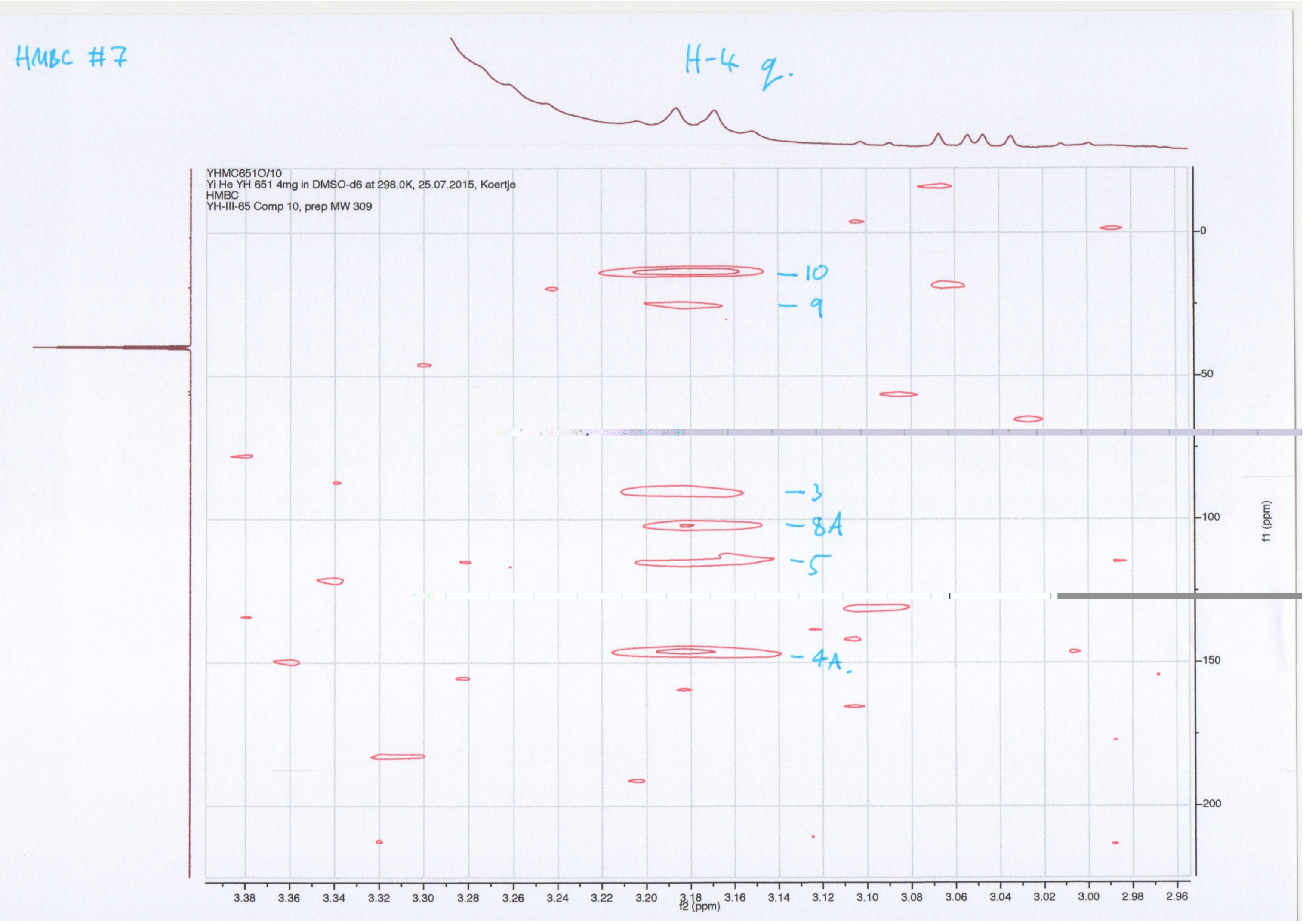

Compound 7 HMBC Expansion

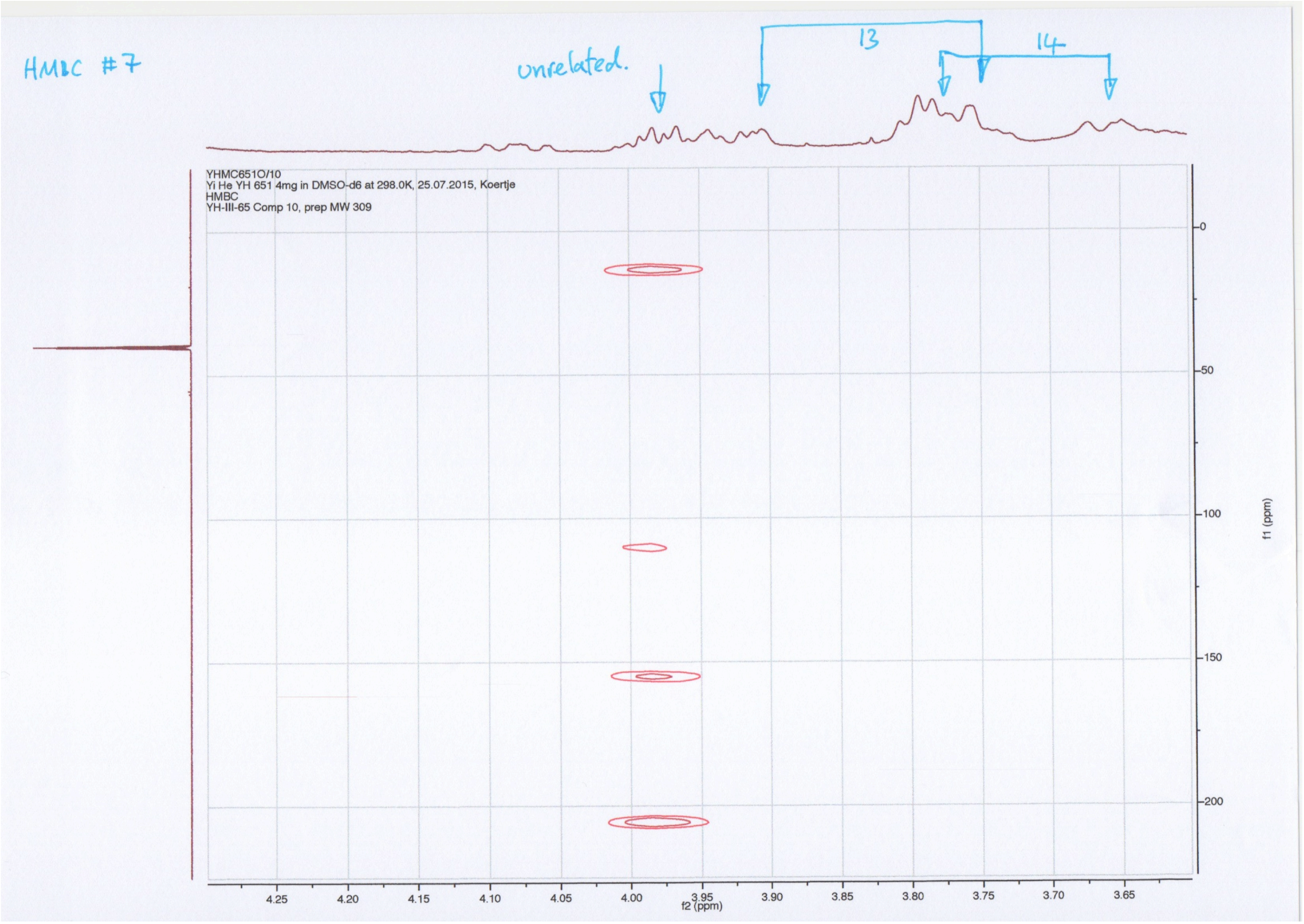

Compound 7 HMBC Expansion - sample too weak to show HMBC correlations from the heavily coupled 13- and 14-protons

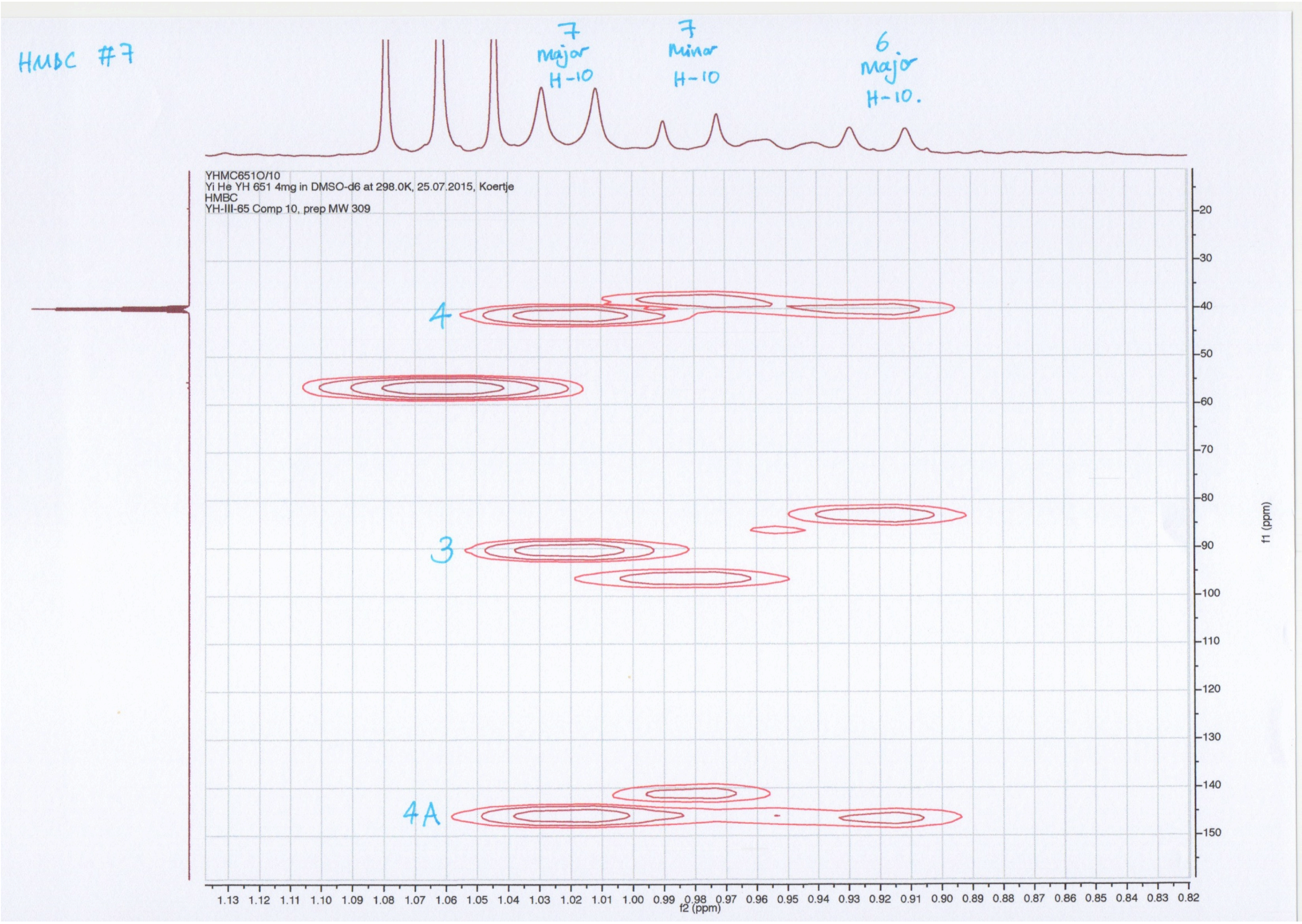

Compound 7 HMBC Expansion - showing presence of a secoind minor diastereomer and **6**.

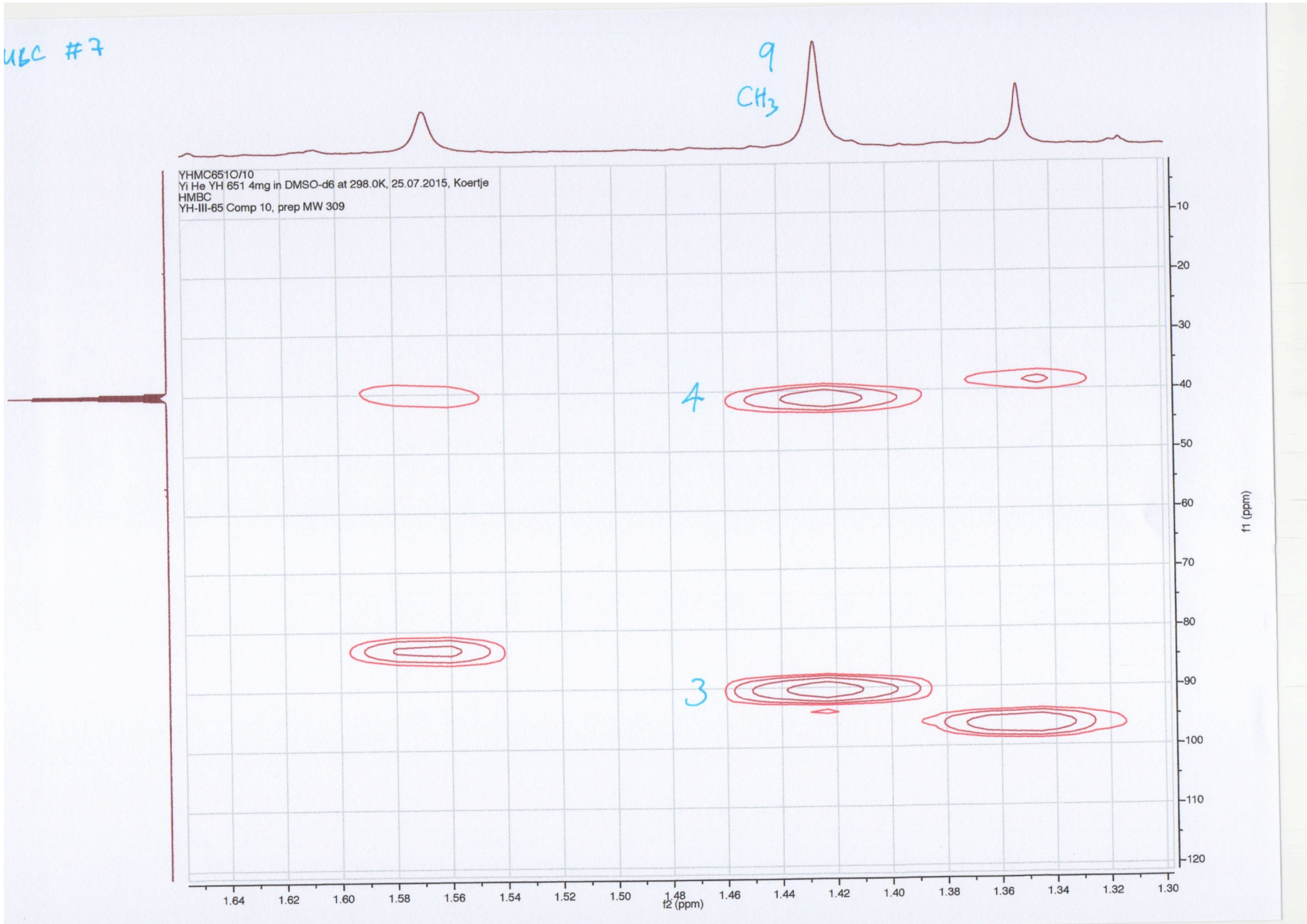

Compound 7 HMBC Expansion

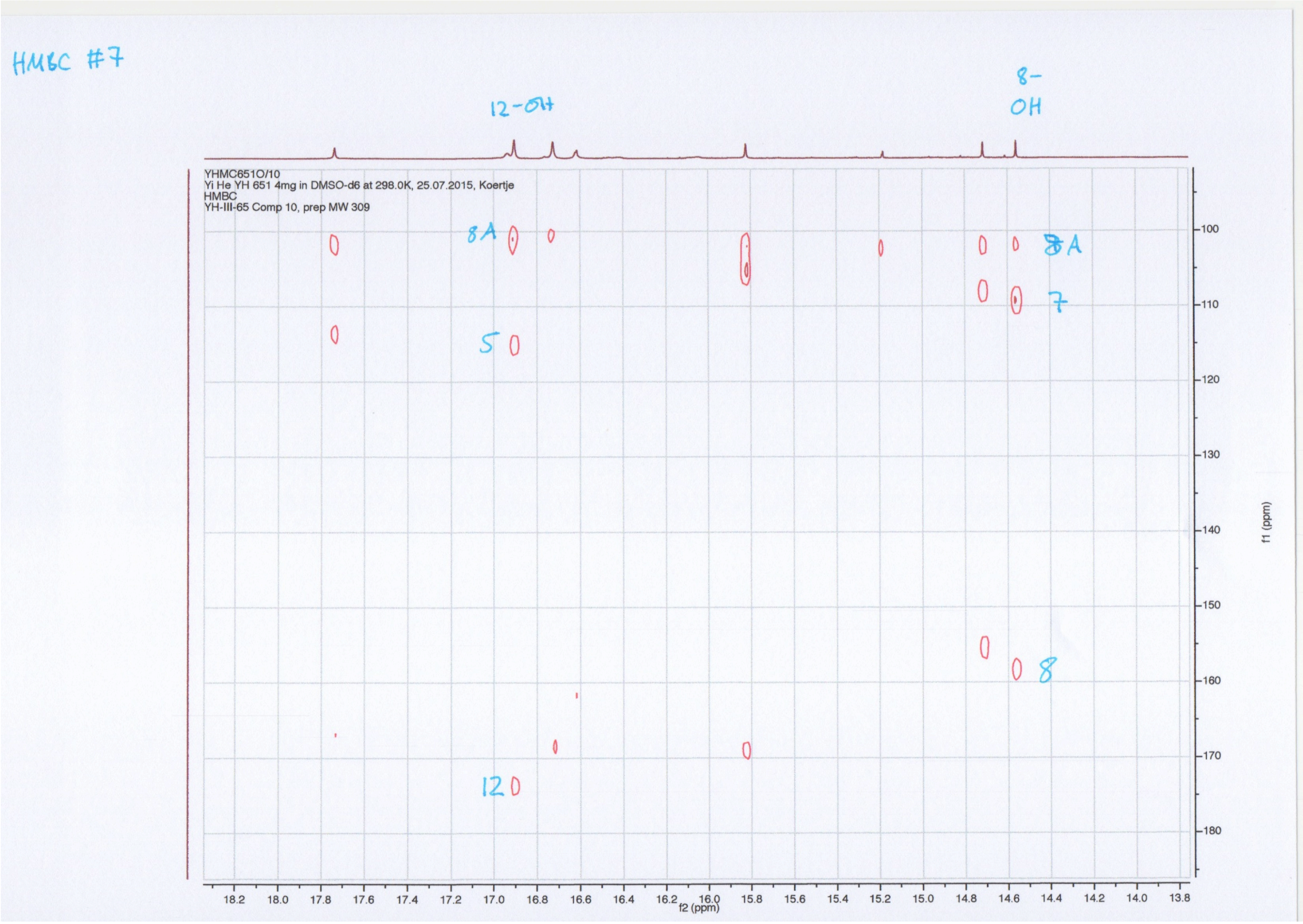

Compound 7 HMBC Expansion

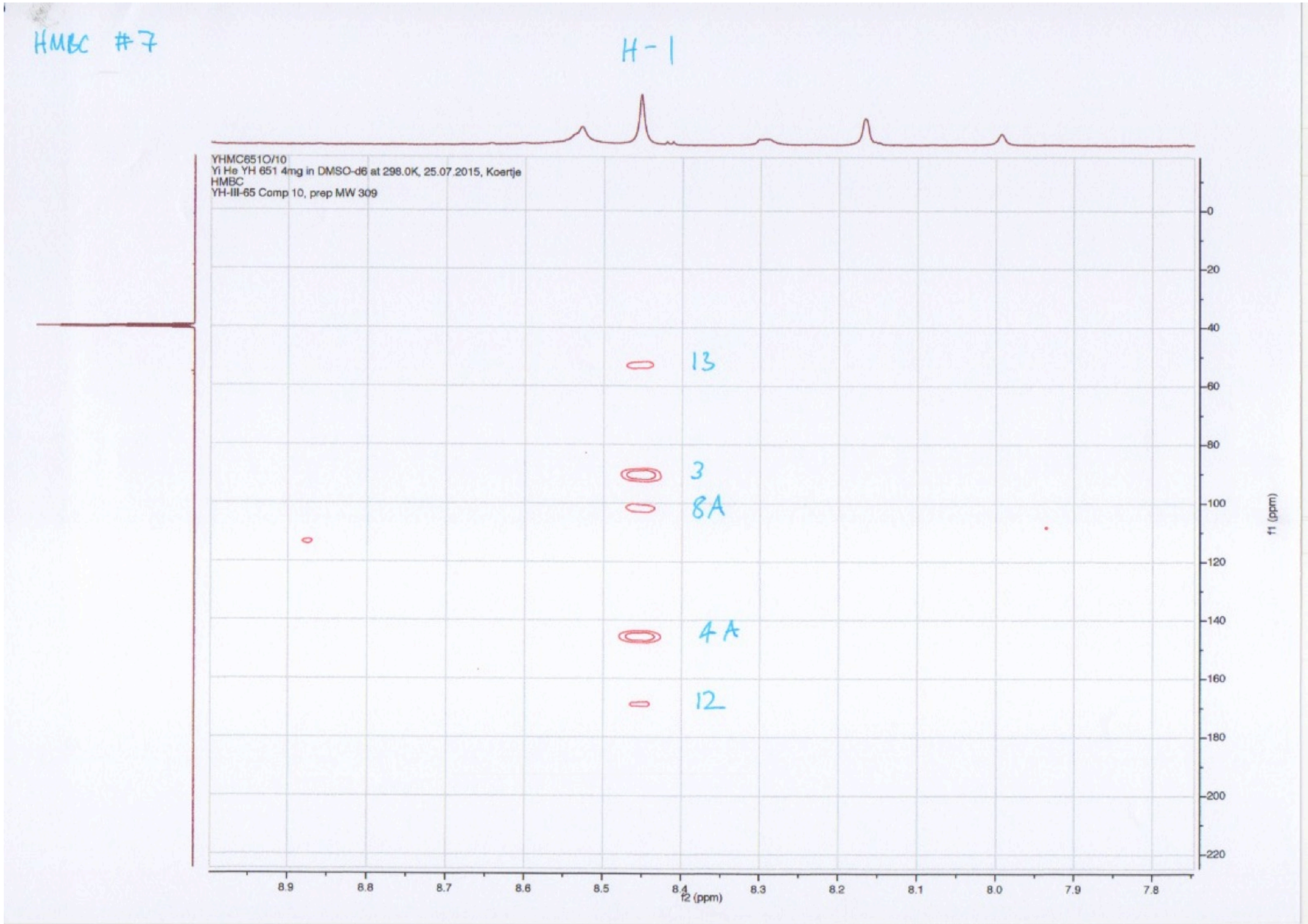

Compound 7 HMBC Expansion - key correlations from H-1

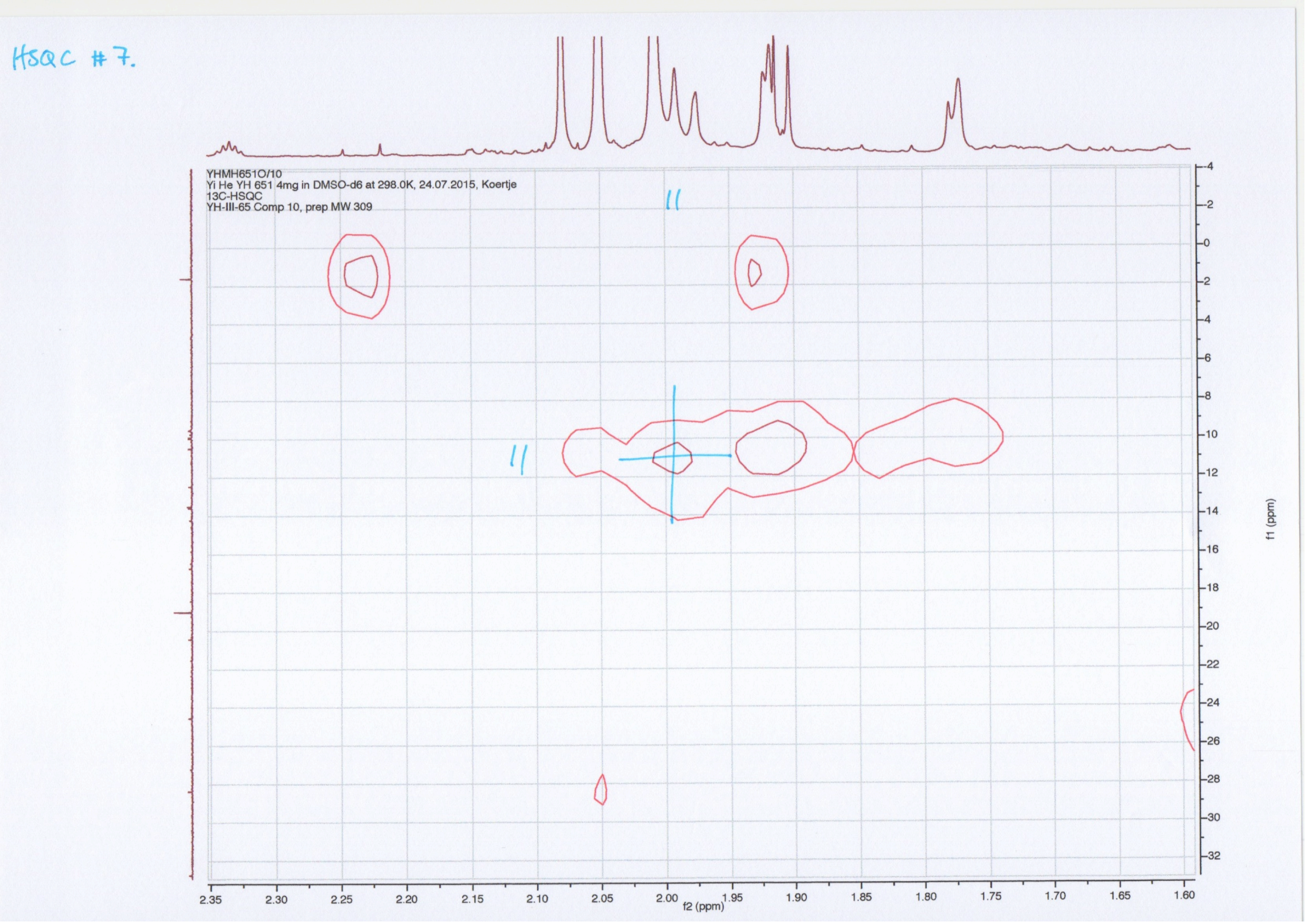

Compound 7 HSQC Expansion

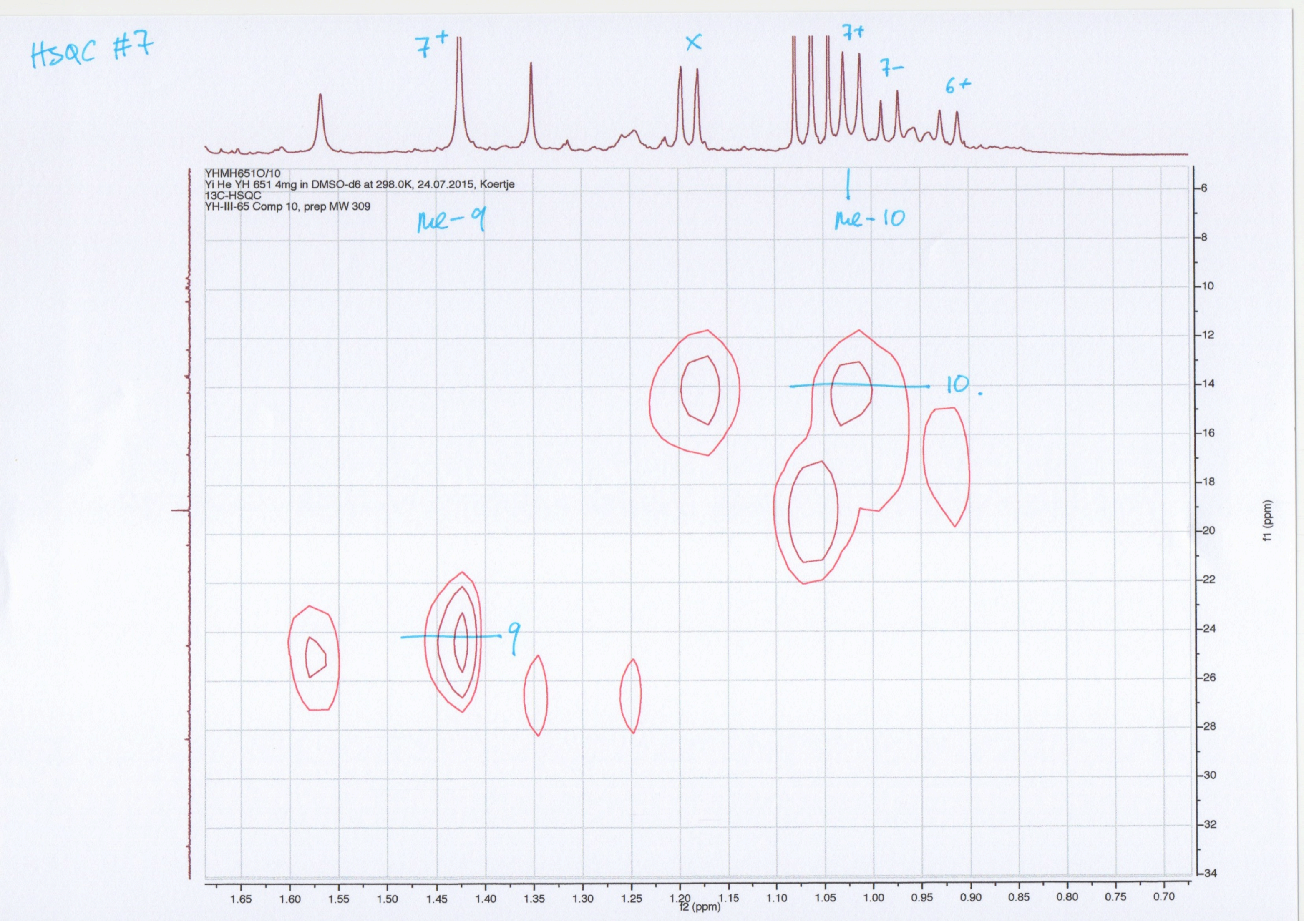

Compound 7 HSQC Expansion

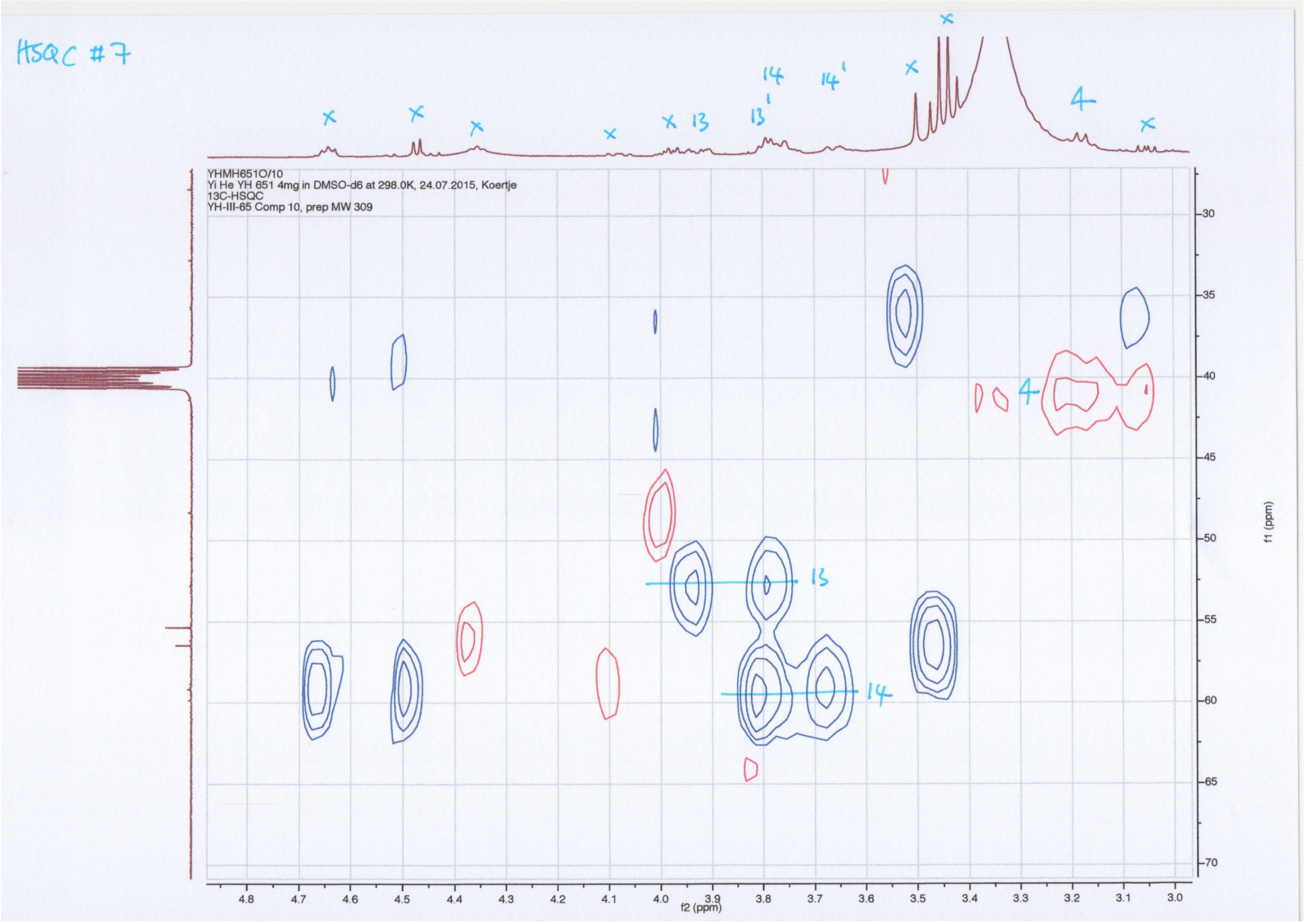

Compound 7 HSQC Expansion

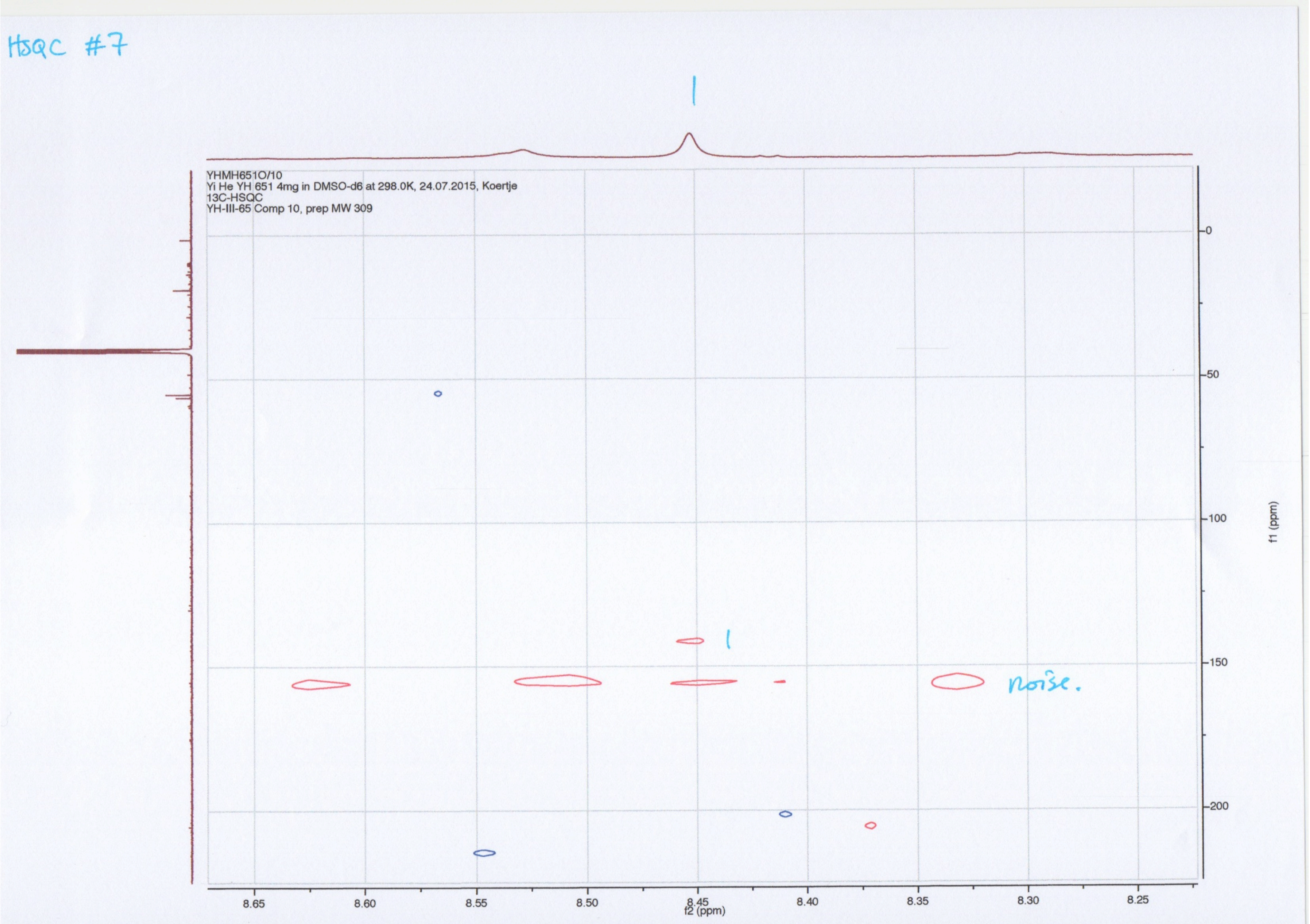

Compound 7 HSQC Expansion

7.8 NMR Spectra Compound 8

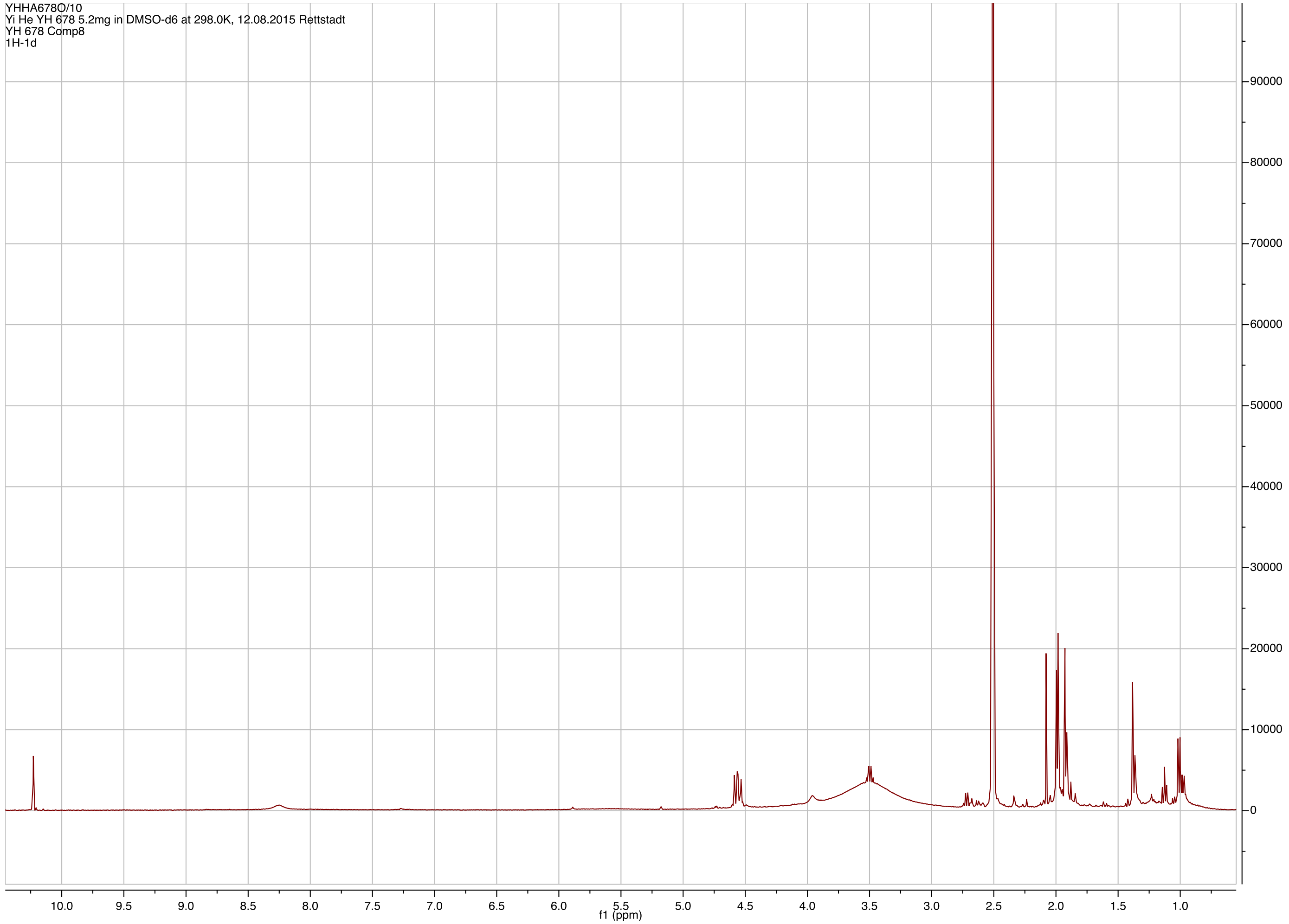

Compound 8 ^{1}H NMR DMSO-d_6 400 MHz

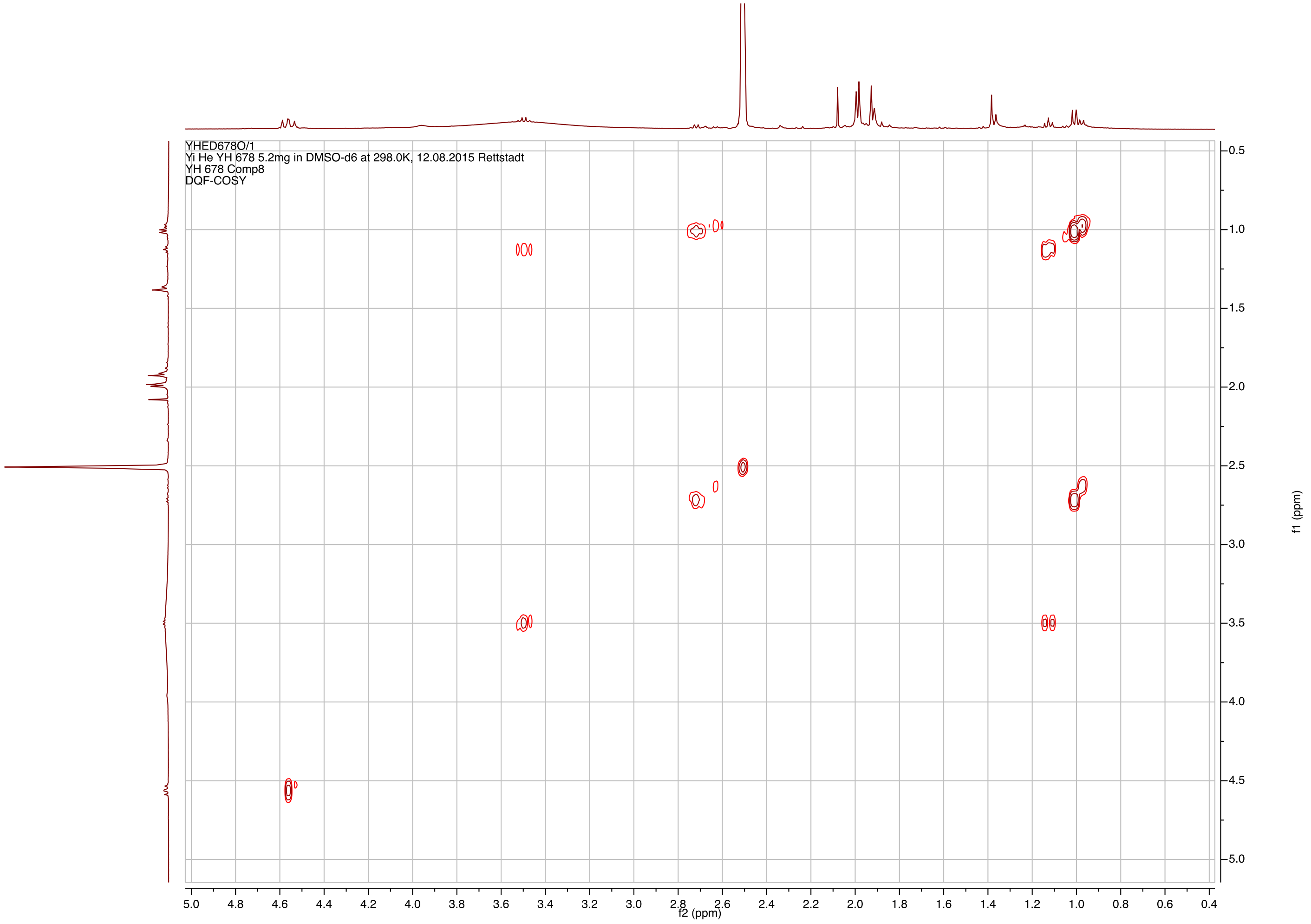

Compound 8 COSY

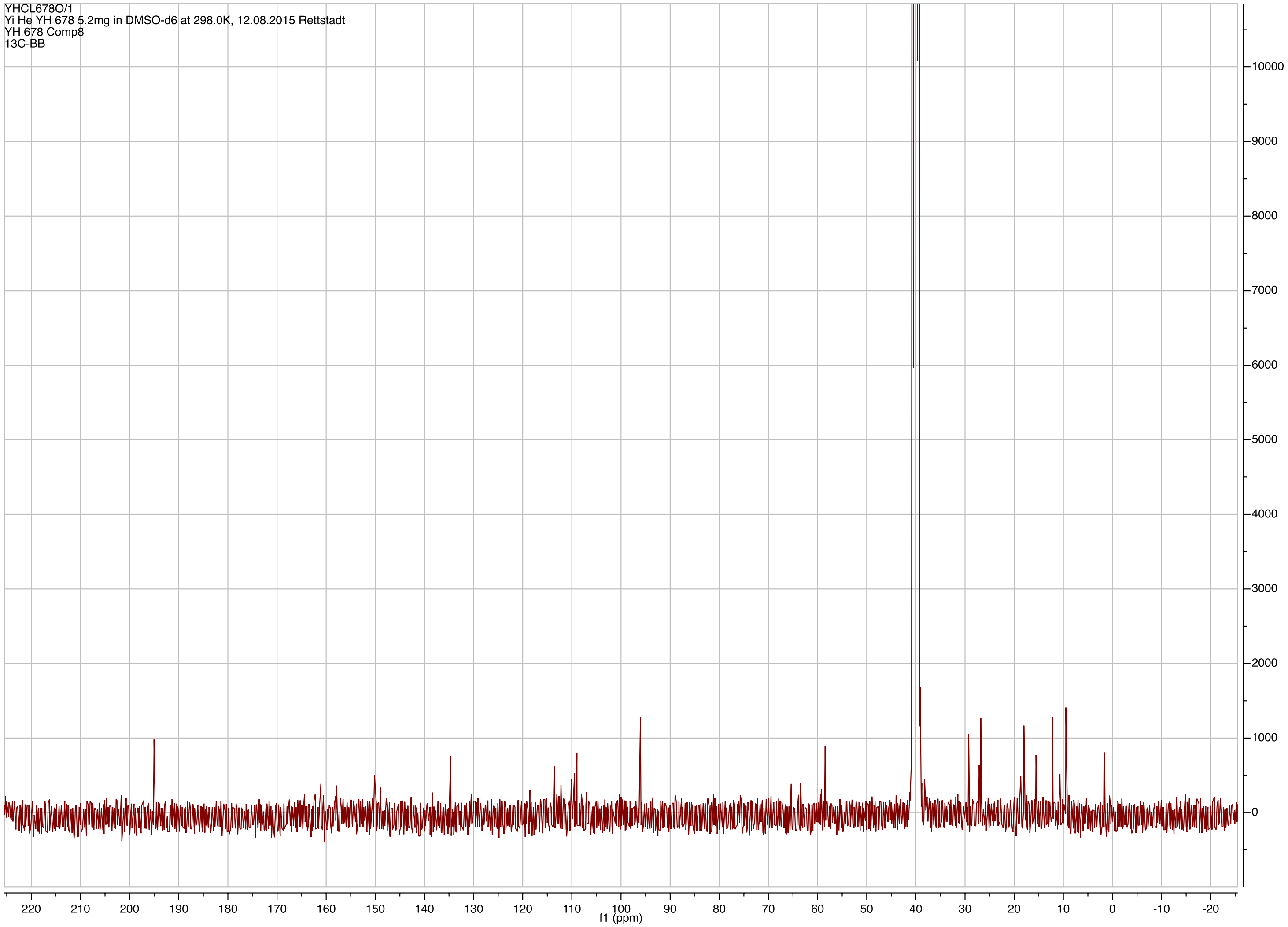

Compound 8 ^{13}C NMR DMSO-d6 100 MHz

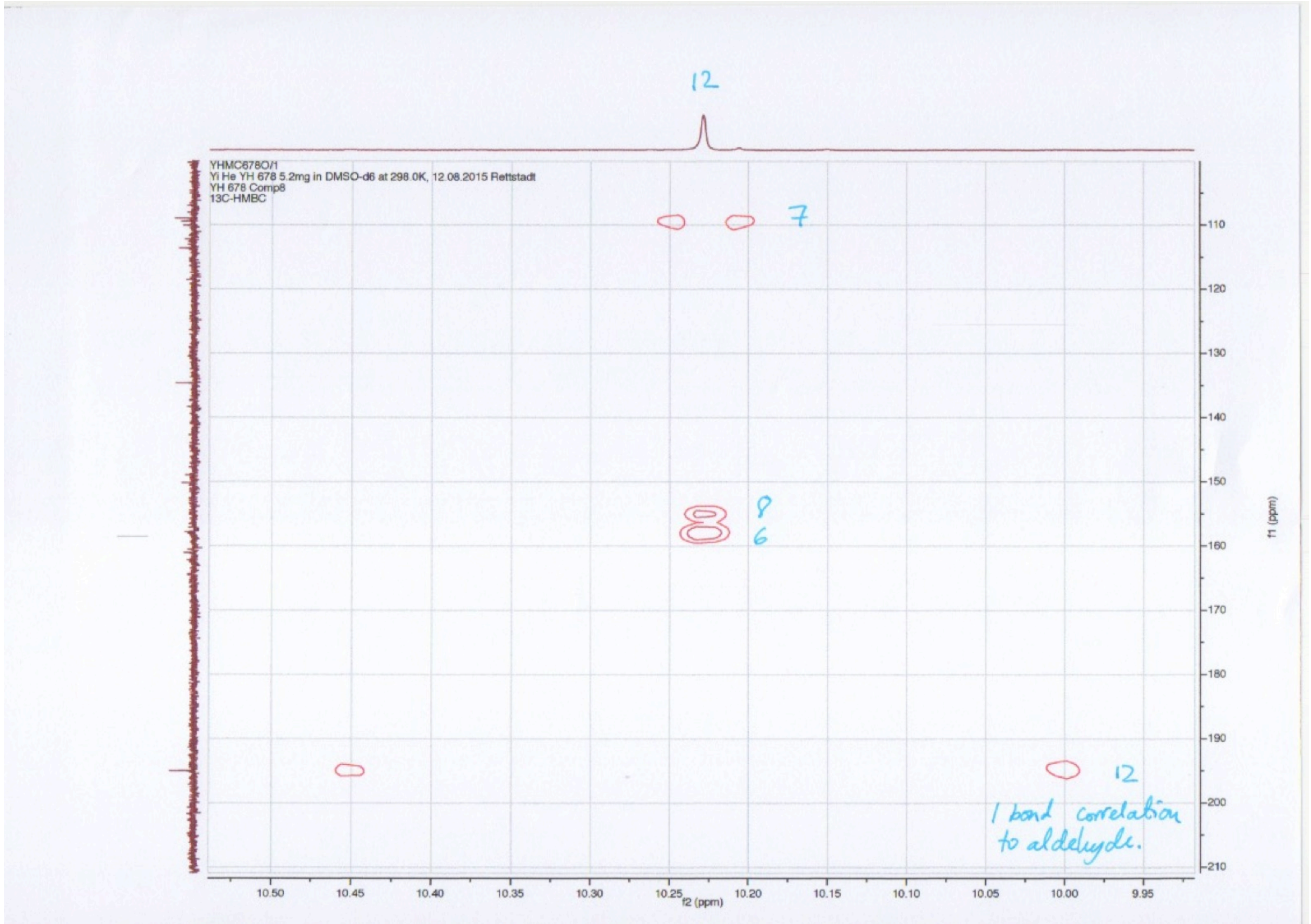

Compound 8 HMBC Expansion. Note that aldehyde C-H coupling of *ca* 8 Hz means that aldehyde shows in HMBC spectrum and not in HSQC.

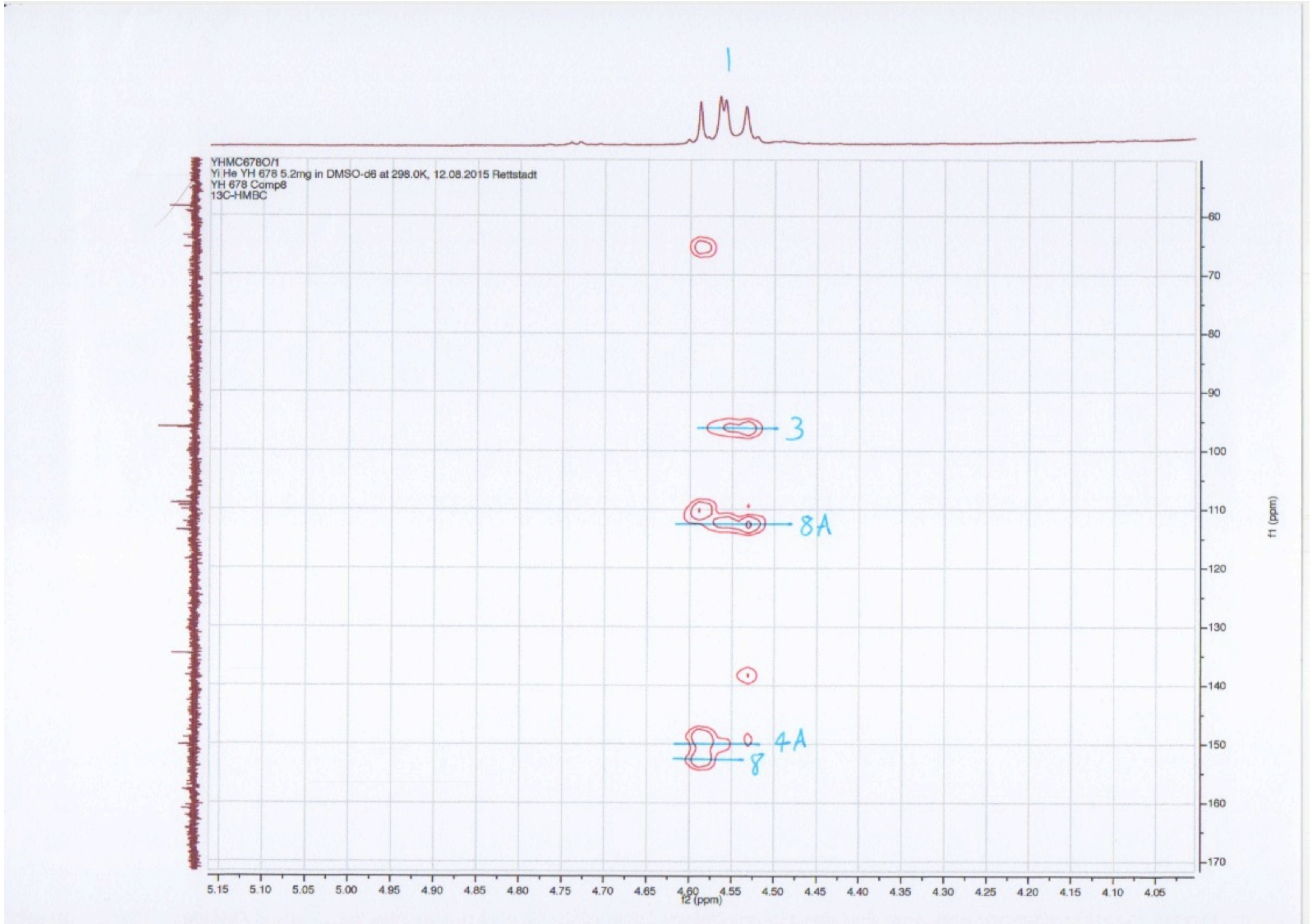

Compound 8 HMBC Expansion

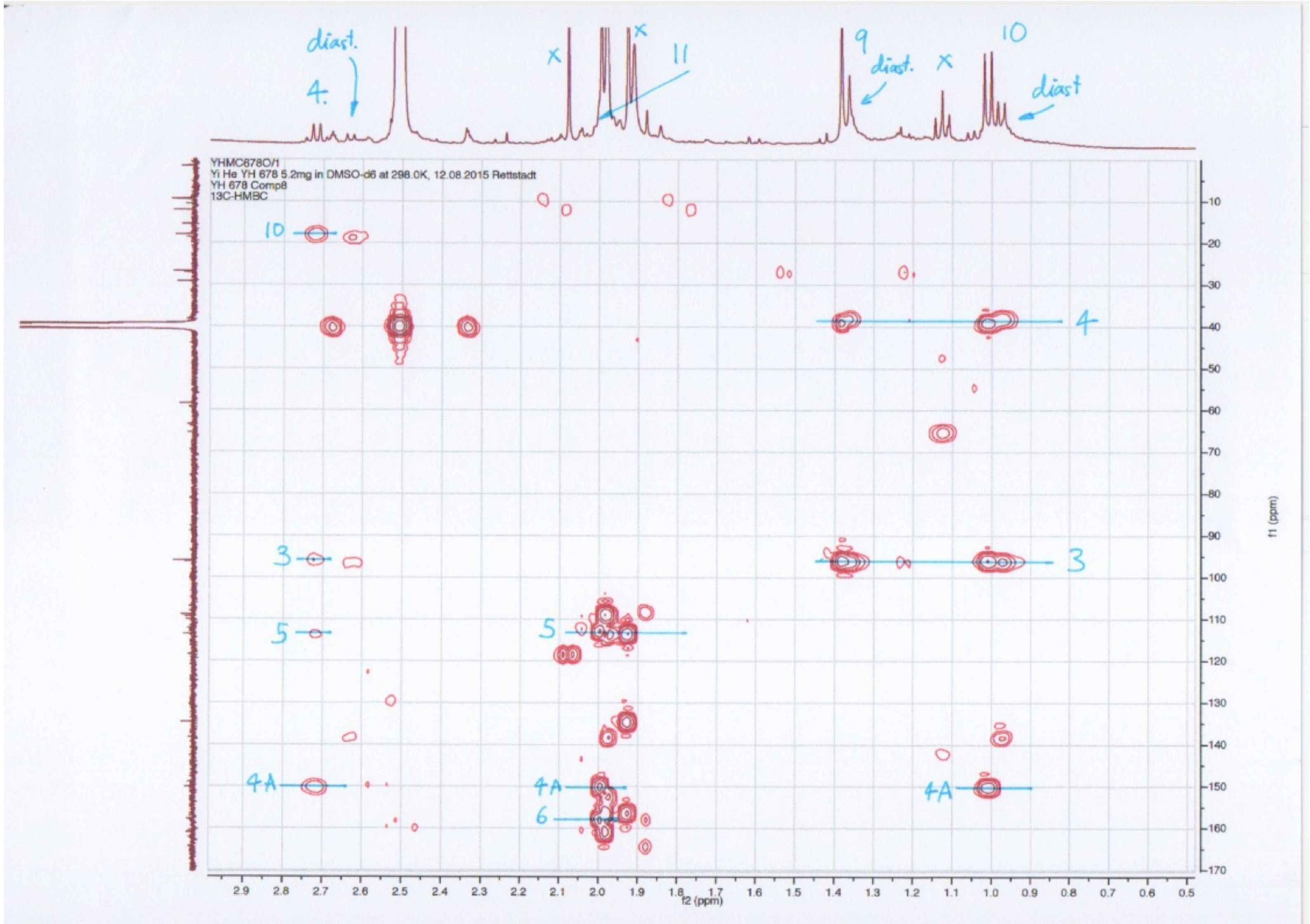

Compound 8 HMBC Expansion

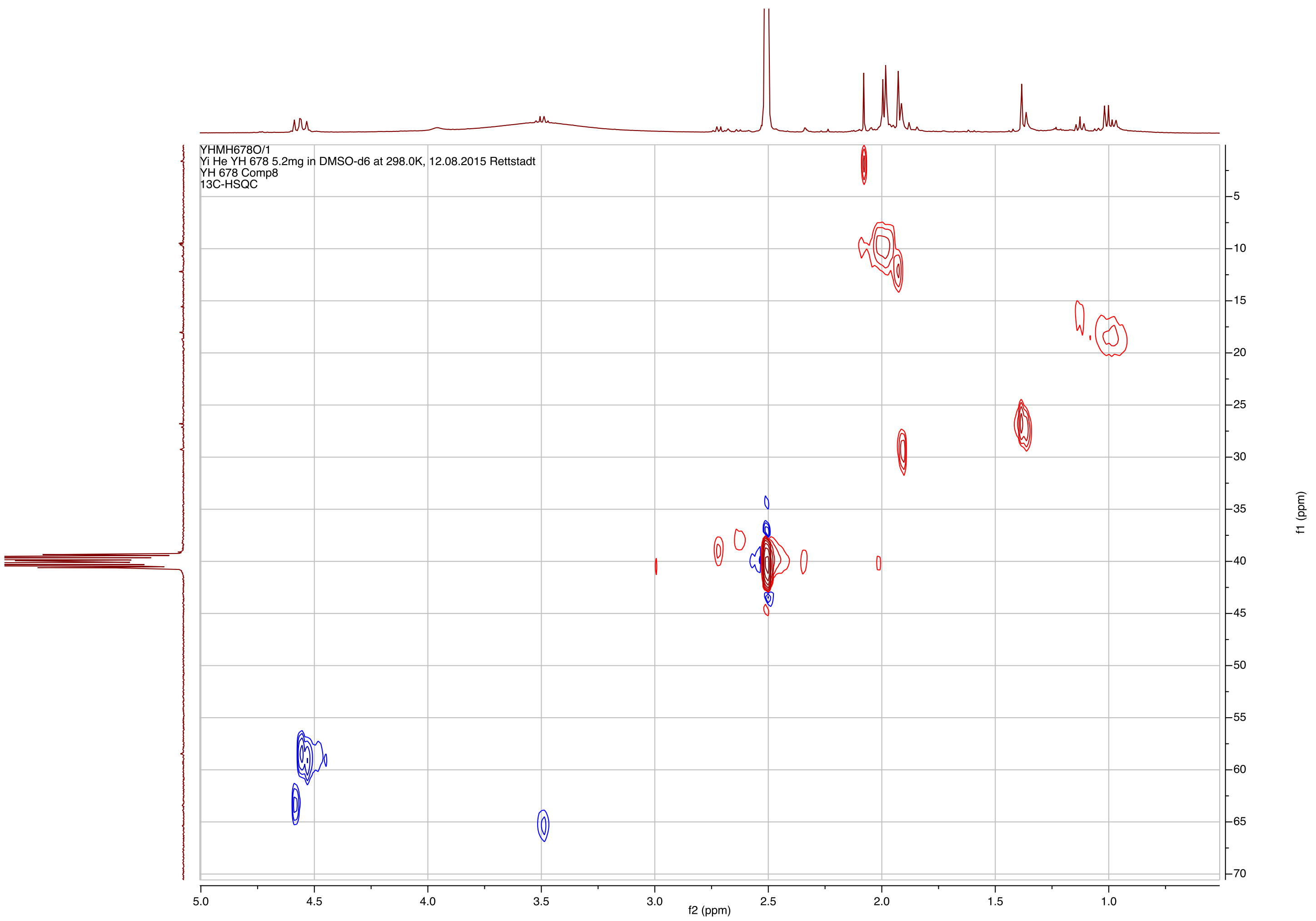

Compound 8 HSQC

7.9 NMR Spectra Compound 9

Insufficient **9** was purified to collect full 2D data - in particular correlations from H-12 to C-12, C-6, C-7 and C-8 are too weak to observe. However comparison of the extant NMR data with that of **8** and HRMS data suggest it is the methyl acetal **9**.

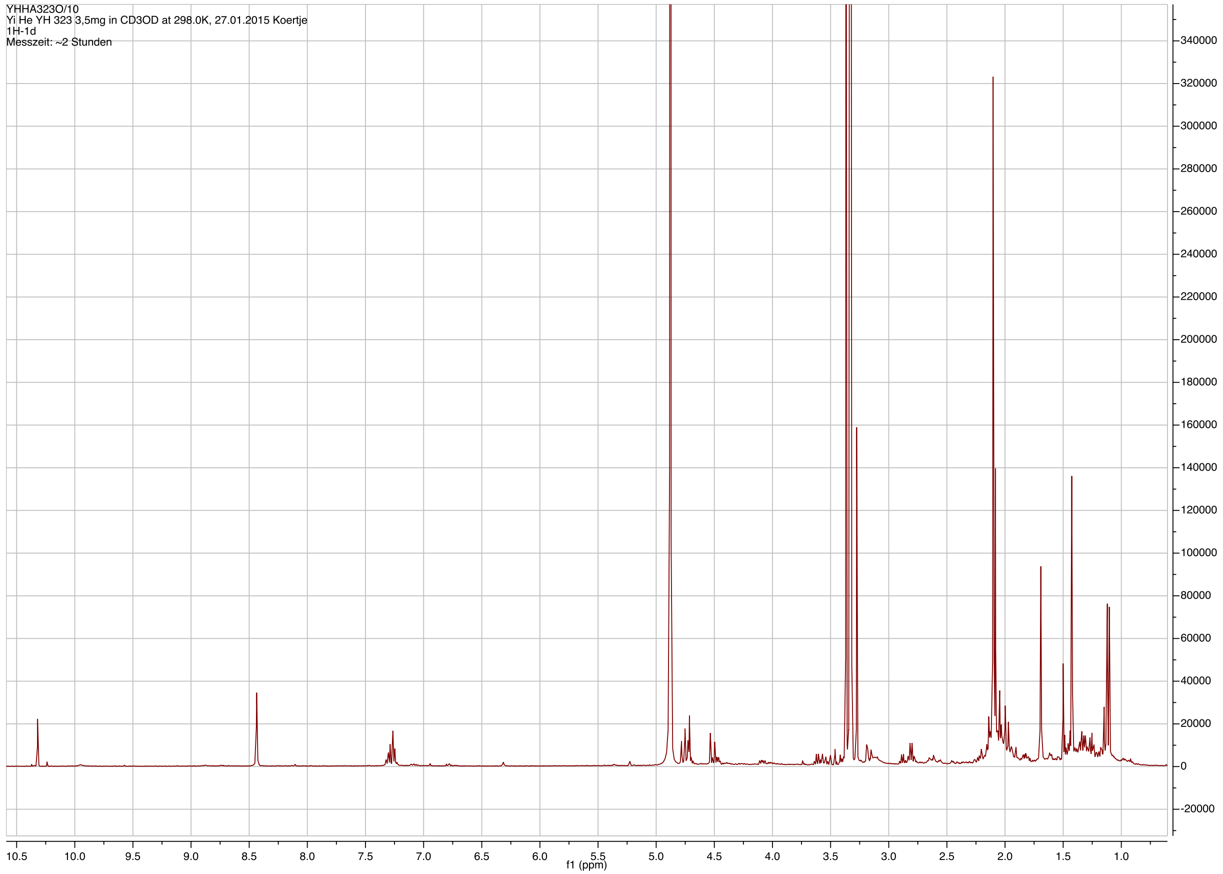

Compound 9 ^{1}H NMR CD_3OD 400 MHz

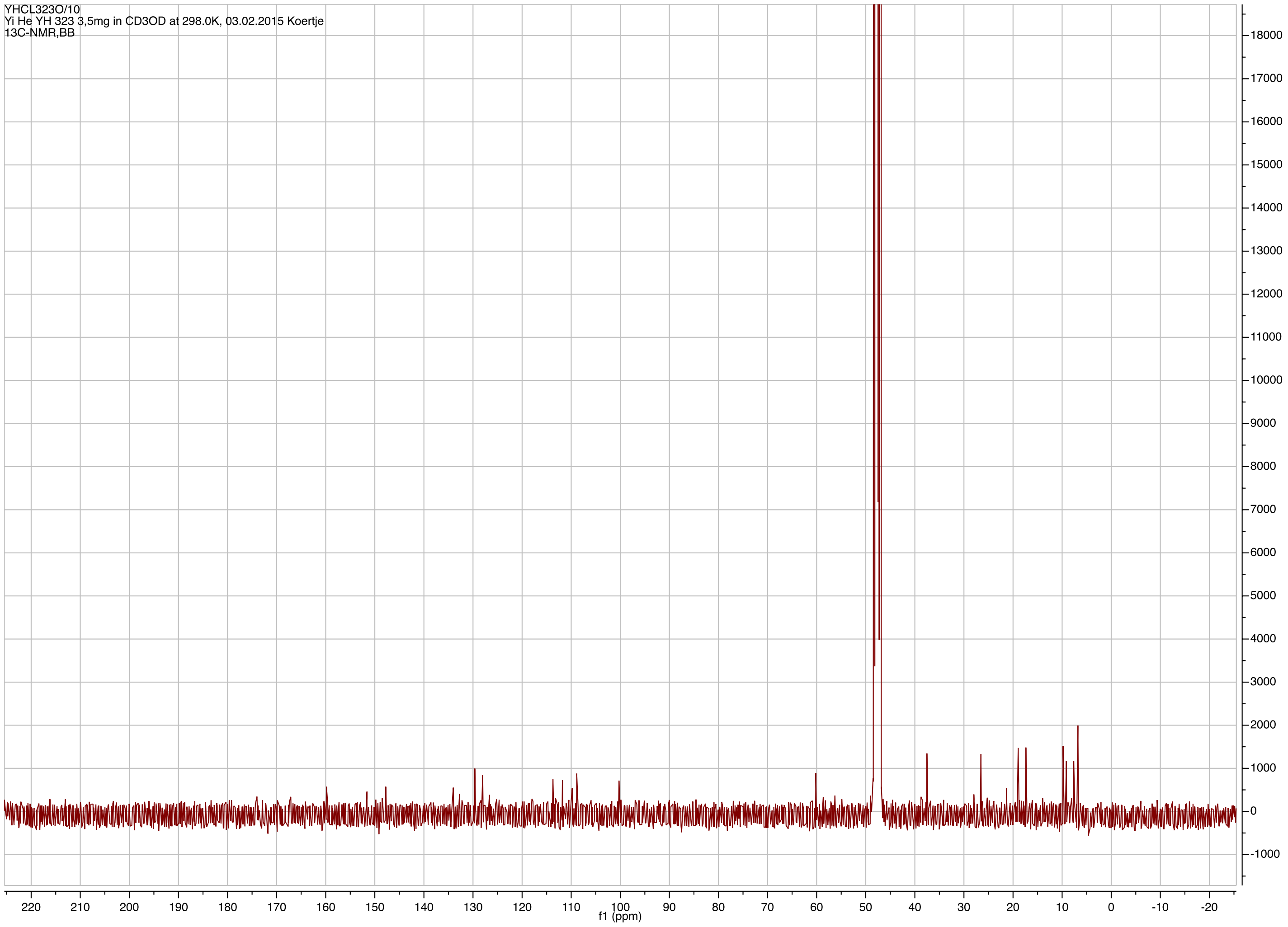

Compound 9 $CDCl_3$ ^{13}C NMR 100 MHz

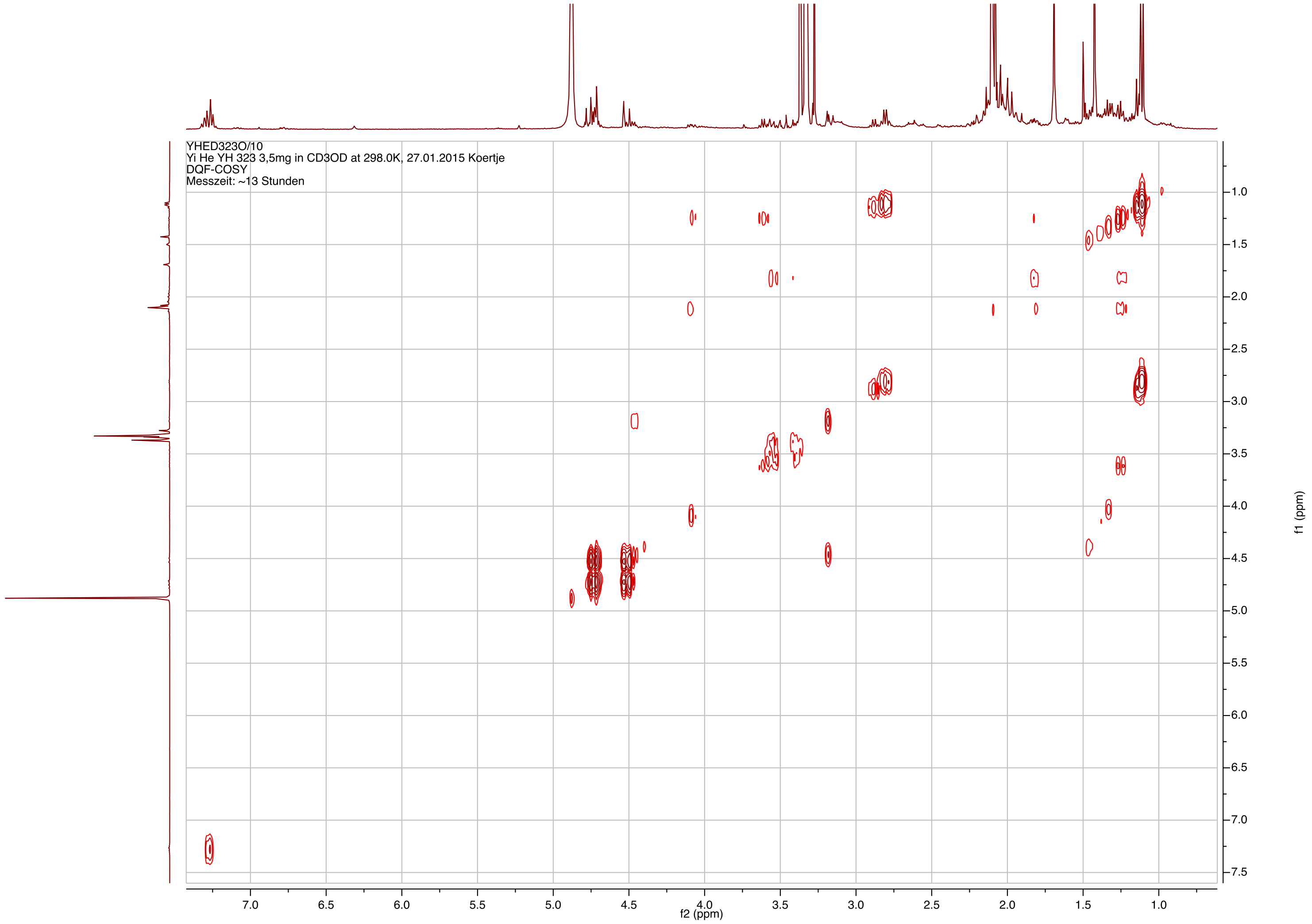

Compound 9 COSY

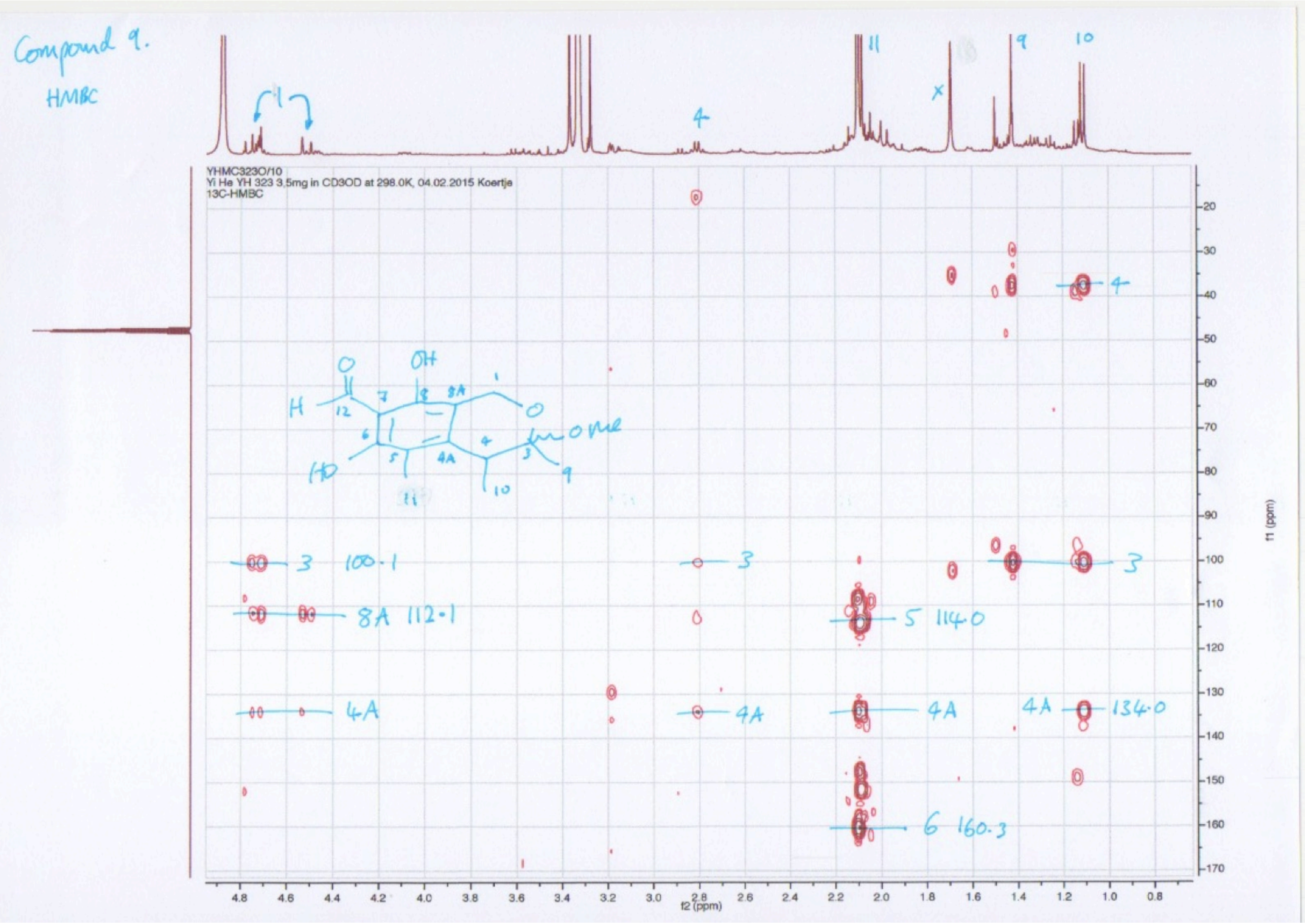

Compound 9 HMBC expansion

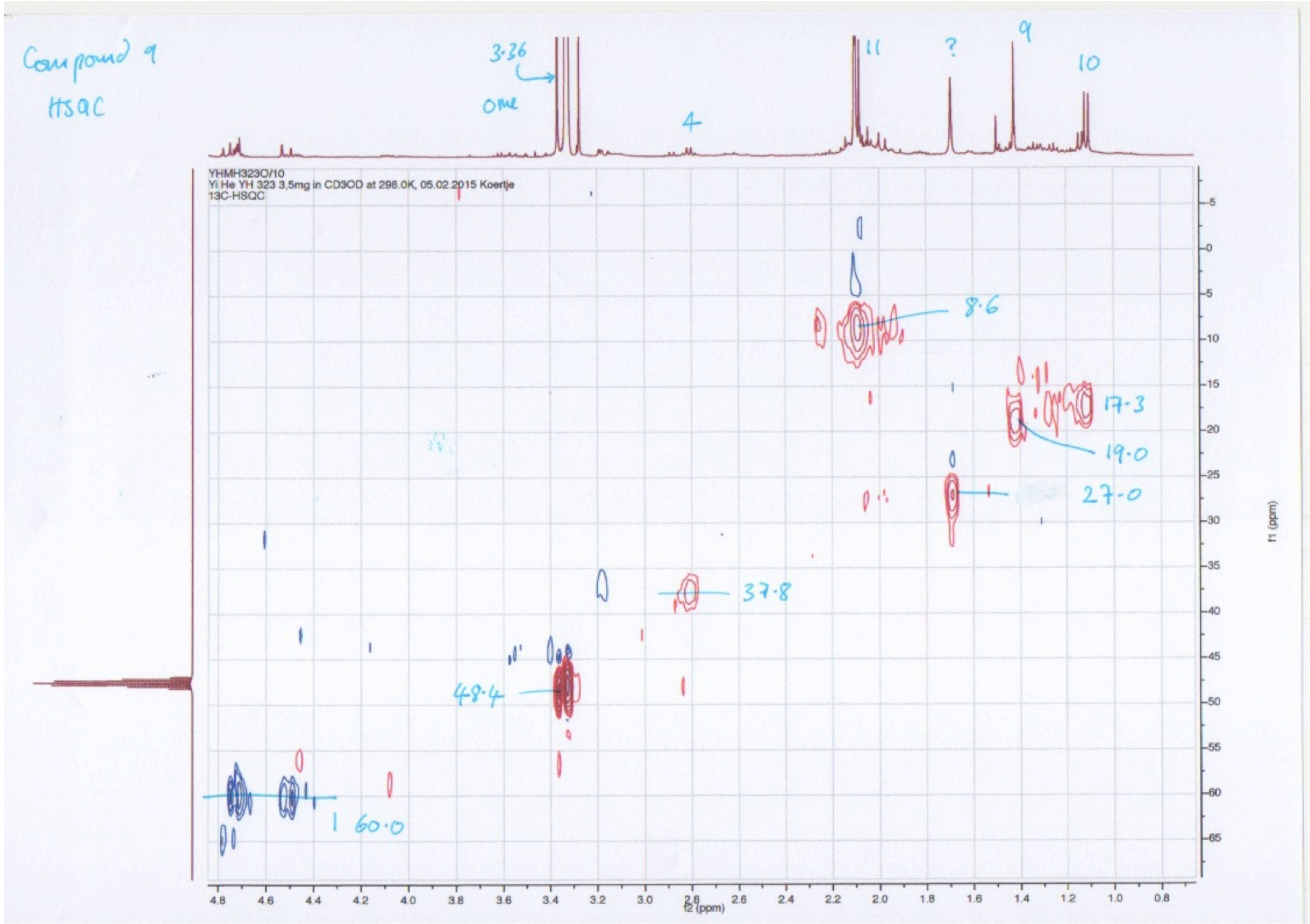

Compound 9 HSQC

8. Experimental Section

8.1 General

LC-MS data were obtained with using a Waters LCMS system comprising of a Waters 2767 autosampler, Waters 2545 pump system, a Phenomenex Kinetex column (2.6 μ, C_{18}, 100 Å, 4.6 × 100 mm) equipped with a Phenomenex Security Guard precolumn (Luna C_5 300 Å) eluted at 1 mL/min. Detection was by Waters 2998 Diode Array detector between 200 and 600 nm; Waters 2424 ELSD and Waters SQD-2 mass detector operating simultaneously in ES+ and ES- modes between 100 *m/z* and 650 *m/z*. Solvents were: A, HPLC grade H_2O containing 0.05% formic acid; B, HPLC grade MeOH containing 0.045% formic acid; and C, HPLC grade CH_3CN containing 0.045% formic acid. Gradients were as follows. *Method 1*. Kinetex/CH_3CN: 0 min, 10% C; 10 min, 90% C; 12 min, 90% C; 13 min, 10% C; 15 min, 10% C.

Semi-Preparative LCMS and compound purification.

Purification of compounds was generally achieved using a Waters mass-directed autopurification system comprising of a Waters 2767 autosampler, Waters 2545 pump system, a Phenomenex Kinetex Axia column (5μ, C_{18}, 100 Å, 21.2 × 250 mm) equipped with a Phenomenex Security Guard precolumn (Luna C_5 300 Å) eluted at 20 mL/min at ambient temperature. Solvent A, HPLC grade H_2O + 0.05% formic acid; Solvent B, HPLC grade CH_3CN + 0.045% formic acid. The post-column flow was split (100:1) and the minority flow was made up with HPLC grade MeOH + 0.045% formic acid to 1 mL·min-1 for simultaneous analysis by diode array (Waters 2998), evaporative light scattering (Waters 2424) and ESI mass spectrometry in positive and negative modes (Waters SQD-2). Detected peaks were collected into glass test tubes. Combined tubes were evaporated under a flow of dry N_2 gas, weighed, and residues dissolved directly in NMR solvent for NMR analysis.

8.2 General techniques for DNA manipulation

Polymerase chain reactions were performed with PrimeSTAR® HS DNA Polymerase (TaKaRa Bio Inc.). Restriction digests were carried out according to the manufacturer's protocols (NEB, Fermentas, Promega). The primers used to amplify each fragment were synthesized by Sigma, and are listed in Table S2.

8.3 Strains and culturing

Escherichia coli TOP10 (Invitrogen) was used as the host for plasmids that did not contain a Gateway destination cassette. Gateway destination vectors were propagated in *E.coli ccdB* Survival™ 2 T1R cells (Invitrogen). *Saccharomyces cerevisiae* strain YPH499 (Stratagene) was used as the host for plasmid assembly by homologous recombination. *Aspergillus oryzae* strainNSAR1, a quadruple auxotrophic (*niaD⁻ sC⁻ ΔargB adeA⁻*) host, was used for heterologous expression of citrinin gene cluster, and routinely maintained at 28 °C on DPY (2% (w/v) dextrin from potato starch, 1% (w/v) polypeptone, 0.5% (w/v) yeast extract, 0.5% (w/v) monopotassium phosphate, 0.05% (w/v) magnesium sulfate, 2.5% (w/v) agar) plate. *A. oryzae*

strainM-2-3, an arginine auxotroph, was obtained from Professor Teruo Fujii, the University of Tokyo and mycelium was routinely maintained at 28 °C on MEA (3.36% malt extract agar) plate.

8.4 Cloning procedures for heterologous expression of citrinin genes in A. oryzae NSAR1

8.4.1 exp. 1: The *citS* gene was amplified from *M. ruber* M7 genomic DNA as four fragments using primers pks-1-F/pks-1-R, pks-2-F/pks-2-R, pks-3-F/pks-3-R, pks-4-F/pks-4-R according to the strategy described in Fig. S8.1. These fragments with overlaps with each other were reassembled by homologous recombination in *S. cerevisiae* with *Not*I and *Asc*I cut pE-YA vector and shuttled back into *E. coli* to create pE-YA·*citS* (the only 56-bp intron in *citS* based on bioinformatics analysis was removed). The cloned *citS* gene with the only 56-bp intron removed was then transferred into pTYGS·arg by Gateway LR recombination (Invitrogen) to create pTYGS·arg·*citS*. Thus, the *citS* gene was placed under the control of P_{amyB} and T_{amyB}. Transformation of *A. oryzae* NSAR1 with this plasmid yielded 8 transformants.

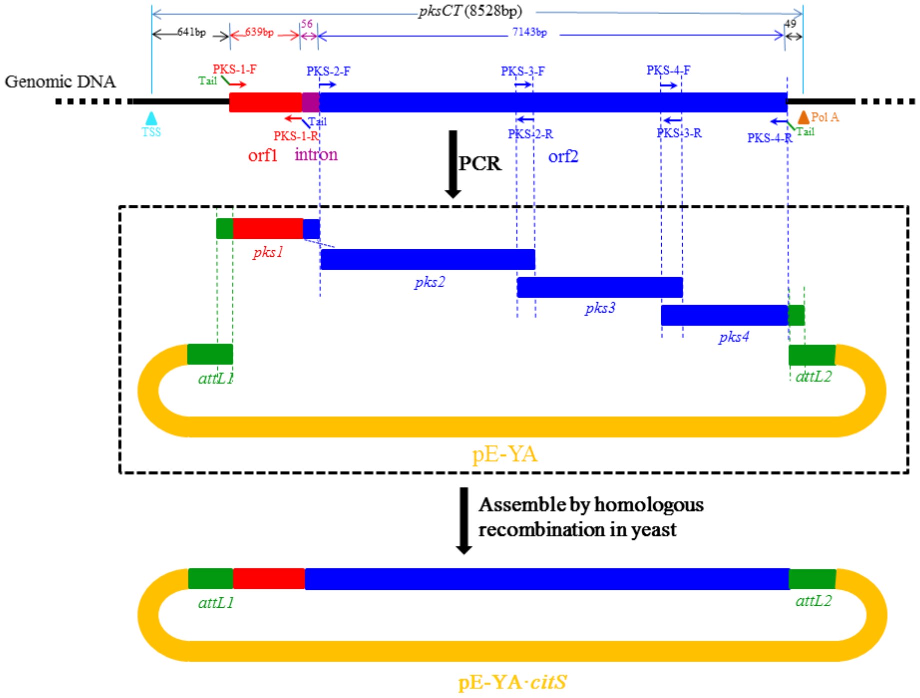

Figure S8.1 Cloning strategy of *citS* to yield pE-YA·*citS*

8.4.2 exp. 2: The long 942-bp *mrl1* gene was amplified from *M. ruber* M7 cDNA as a single fragment using primers mrl1-F-long and mrl1-R which flanked the PCR product with sequences overlapping the 3′ terminal of alcohol dehydrogenase promoters (P_{adh}) and the 5′ terminal of enolase terminator (T_{eno}). This fragment was cloned into *Asc*I-*cut* pTYGS·arg·*citS* under the control of P_{adh} and T_{eno} by homologous recombination in *S. cerevisiae* and shuttled back into *E. coli* to create pTYGS·arg·*citS*·*mrl1*·long. Transformation of *A. oryzae* NSAR1 with this plasmid yielded 13 transformants.

exp. 2′: The short *mrl1* gene with first 156 bp nucleotides removed (define as *mrl1* gene in this MS) was amplified from *M. ruber* M7 cDNA as a single fragment using primers mrl1-F and mrl1-R which flanked the PCR product with sequences overlapping the 3′ terminal of P_{adh} and the 5′ terminal of T_{eno}. The same method was used to create pTYGS·arg·*citS*·*mrl1*. Transformation of *A. oryzae* NSAR1 with this plasmid yielded 10 transformants.

8.4.3 exp. 3: The *mrl2* gene was amplified from *M. ruber* M7 cDNA as a single fragment using primers mrl2-F and mrl2-R-b which flanked the PCR product with sequences overlapping P_{adh} and T_{eno}. This fragment was cloned into *Asc*I-*cut* pTYGS·ade under the control of P_{adh} and T_{eno} by homologous recombination in *S. cerevisiae* and shuttled back into *E. coli* to create pTYGS·ade·*mrl2*. Transformation of *A. oryzae* NSAR1 harboring pTYGS·arg·*citS*·*mrl1* with this plasmid yielded 10 transformants.

8.4.4 exp. 4: The *mrl2* and *mrl4* genes were amplified from *M. ruber* M7 cDNA as single fragment using primers mrl2-F/mrl2-R and mrl4-F/mrl4-R-b respectively. The cloned *mrl2* gene was 5′ flanked by a 30-bp nucleotides overlap with P_{adh} and 3′ flanked by a 30-bp nucleotides overlap with the alcohol dehydrogenase terminator (T_{adh}). The cloned *mrl4* gene was 5′ flanked by a 30-bp nucleotides overlap with the glyceraldehyde-3-phosphate dehydrogenase promoter (P_{gpdA}) and 3′ flanked by a 30-bp nucleotides overlap with T_{eno}. These two fragments together with a patch fragment T_{adh}-P_{gpdA} (gel purified from *Asc*I digested pTYGS·ade vector) were cloned into *Asc*I-*cut* pTYGS·ade by homologous recombination in *S. cerevisiae* and shuttled back into *E. coli* to create pTYGS·ade·*mrl2*·*mrl4*. Transformation of *A. oryzae* NSAR1 harboring pTYGS·arg·*citS*·*mrl1* with this plasmid yielded 22 transformants.

8.4.5 exp. 5: The *mrl7* gene was amplified from *M. ruber* M7 cDNA as a single fragment using primers mrl7-F and mrl7-R which flanked the PCR product with sequences overlapping the upstream and downstream of *Not*I and *Asc*I cut pE-YA vector. This fragment was reassembled by homologous recombination in *S. cerevisiae* with *Not*I and *Asc*I cut pE-YA vector and shuttled back into *E. coli* to create pE-YA·*mrl7*. The cloned *mrl7* gene was then transferred into pTYGS·ade·*mrl2* by Gateway LR recombination to create pTYGS·ade·*mrl2*·*mrl7*. Thus, the *mrl7* gene was placed under the control of P_{amyB} and T_{amyB}. Transformation of *A. oryzae* NSAR1 harboring pTYGS·arg·*citS*·*mrl1* with this plasmid yielded 16 transformants.

8.4.6 exp. 6: The *mrl2* and *mrl6* genes were amplified from *M. ruber* M7 cDNA as single fragment using primers mrl2-F/mrl2-R and mrl6-F-b/mrl6-R respectively. The cloned *mrl2* gene was 5′ flanked by a 30-bp nucleotides overlap with P_{adh} and 3′ flanked by a 30-bp nucleotides overlap with the T_{adh}. The cloned *mrl6* gene was 5′ flanked by a 30-bp nucleotides overlap with the P_{gpdA} and 3′ flanked by a 30-bp nucleotides overlap with T_{eno}. These two fragments together with a patch fragment T_{adh}-P_{gpdA} (gel purified from *Asc*I digested pTYGS·ade vector) were cloned into *Asc*I-*cut* pTYGS·ade by homologous recombination in *S. cerevisiae* and shuttled back into *E. coli* to create pTYGS·ade·*mrl2·mrl6*. Transformation of *A. oryzae* NSAR1 harboring pTYGS·arg·*citS·mrl1* with this plasmid yielded 27 transformants.

8.4.7 exp. 7: The constructed vector pE-YA·*mrl7* in 8.4.5 was used to do Gateway LR recombination with pTYGS·ade·*mrl2·mrl4* which constructed in 8.4.4 to create pTYGS·ade·*mrl2·mrl4·mrl7*. Transformation of *A. oryzae* NSAR1 harboring pTYGS·arg·*citS·mrl1* with this plasmid yielded 24 transformants.

8.4.8 exp. 8: The constructed vector pE-YA·*mrl7* in 8.4.5 was used to do Gateway LR recombination with pTYGS·ade·*mrl2·mrl6* which constructed in 8.4.6 to create pTYGS·ade·*mrl2·mrl6·mrl7*. Transformation of *A. oryzae* NSAR1 harboring pTYGS·arg·*citS·mrl1* with this plasmid yielded 41 transformants.

8.4.9 exp. 9: The *mrl2*, *mrl4* and *mrl6* genes were amplified from *M. ruber* M7 cDNA as single fragment using primers mrl2-F/mrl2-R, mrl4-F/mrl4-R and mrl6-F/mrl6-R respectively. The cloned *mrl2* gene was 5′ flanked by a 30-bp nucleotides overlap with P_{adh} and 3′ flanked by a 30-bp nucleotides overlap with T_{adh}. The cloned *mrl4* gene was 5′ flanked by a 30-bp nucleotides overlap with P_{gpdA} and 3′ flanked by a 30-bp nucleotides overlap with the glyceraldehyde-3-phosphate dehydrogenase terminator (T_{gpdA}). The cloned *mrl6* gene was 5′ flanked by a 30-bp nucleotides overlap with the enolase promoter (P_{eno}) and 3′ flanked by a 30-bp nucleotides overlap with T_{eno}. Digestion of pTYGS·ade with *Asc*I generates three fragments: the pTYGS·ade vector plus P_{adh} in one terminal and T_{eno} in the other terminal (11749 bp), T_{adh}-P_{gpdA} (2488 bp), and T_{gpdA}-P_{eno} (808 bp). These three gel purified fragments together with the cloned *mrl2*, *mrl4* and *mrl6* genes were reassembled by homologous recombination in *S. cerevisiae* and shuttled back into *E. coli* to create pTYGS·ade·*mrl2·mrl4·mrl6*. Transformation of *A. oryzae* NSAR1 harboring pTYGS·arg·*citS·mrl1* with this plasmid yielded 33 transformants.

8.4.10 exp. 10: The constructed vector pE-YA·*mrl7* in 8.4.5 was used to do Gateway LR recombination with pTYGS·ade·*mrl2·mrl4·mrl6* which constructed in 8.4.9 to create pTYGS·ade·*mrl2·mrl4·mrl6·mrl7*. Transformation of *A. oryzae* NSAR1 harboring pTYGS·arg·*citS·mrl1* with this plasmid yielded 15 transformants.

8.5 Gene knock-out procedures, schemes in M. ruber M7

8.5.1 exp. 11: gene knock-out of *citS*. The *citS* gene knock-out strategy was designed to insert the 1.2 kb neomycin phosphotransferase resistance gene (*neo*) which was amplified by PCR from pKN1 using the primers G418F and G418R into the internal of *citS*. A 721 bp of 5′ fragment and a 954 bp of 3′ fragment of *citS* were amplified from *M. ruber* M7 genomic DNA using primers pksCT-5F/pksCT-5R and pksCT-3F/pksCT-3R respectively and served as homologous arms for recombination event (Fig. S8.2A). These three fragments were reassembled by homologous recombination in *S. cerevisiae* with *Not*I and *Asc*I cut pE-YA vector and shuttled back into *E. coli* to create pE-YA-citS-KO. Then both pE-YA-citS-KO and pCAMBIA3300 were digested with *Sal*I and *Hin*dIII and ligated by T4 DNA ligase to create pC3300-citS. This plasmid was transformed into *Agrobacterium tumefaciens* EHA105 afterwards. Subsequently, *A. tumefaciens* mediated transformation (ATMT) was performed with MrΔku80 strain (a *ku80* knock-out mutant of *M. ruber* M7). A *citS* knock-out mutant (Δ*citS*::*neo*) was confirmed by analytical PCR (Fig. S8.2B). A 1410 bp fragment was expected to amplify from the WT (MrΔku80) genomic DNA using primers pksCT-VF and pksCT-VR, while nothing was obtained from the *citS* knock-out mutant. A 1.2 kb fragment of the *neo* gene could be amplified using primers G418F and G418R from the *citS* knock-out mutant, while no PCR product got from the WT sample.

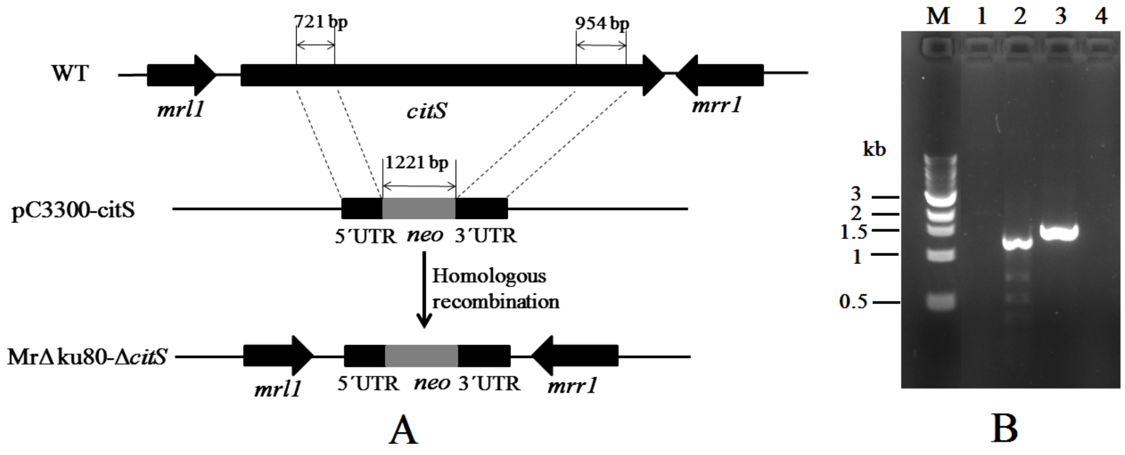

Figure S8.2 Scheme to knock-out *citS* (A) and PCR verification of Δ*citS*::*neo* mutant (B)

Lanes 1/3 and 2/4 were PCR results to amplify partial *citS* gene and *neo* with primers pksCT-VF/pksCT-VR and G418F/G418R respectively. Lane 1 and 2 were using gDNA of Δ*citS*::*neo* mutant as template, lane 3 and 4 were using gDNA of MrΔku80 strain as template. M: NEB 1 kb DNA ladder.

8.5.2 exp. 12: gene knock-out of *mrl1*. The similar strategy was used to inactivate *mrl1*. A 611 bp of 5′ fragment and a 594 bp of 3′ fragment of *mrl1* were amplified from *M. ruber* M7 genomic DNA using primers mrl1-5F/mrl1-5R and mrl1-3F/mrl1-3R respectively and served as homologous arms for recombination event (Fig. S8.3A). These two fragments together with the *neo* gene were reassembled as a knock-out cassette by homologous recombination in *S. cerevisiae* with *Not*I and *Asc*I cut pE-YA vector and shuttled back into *E. coli* to create pE-YA-mrl1-KO. *Kpn*I and *Xba*I digestion and T4 DNA ligation of pE-YA-mrl1-KO and pCAMBIA3300 yielded pC3300-mrl1. The same ATMT method was used to obtain *mrl1* knock-out mutant (Δ*mrl1*::*neo*). Analytical PCR verified the homologous recombination at the right position in the *mrl1* knock-out mutant (Fig. S8.3B). A 633 bp fragment was expected to amplify from the WT (MrΔku80) genomic DNA using primers mrl1-VF and mrl1-VR, while nothing was obtained from the *mrl1* knock-out mutant. A 1.2 kb fragment of the *neo* gene could be amplified using primers G418F and G418R from the *mrl1* knock-out mutant, while no PCR product got from the WT sample.

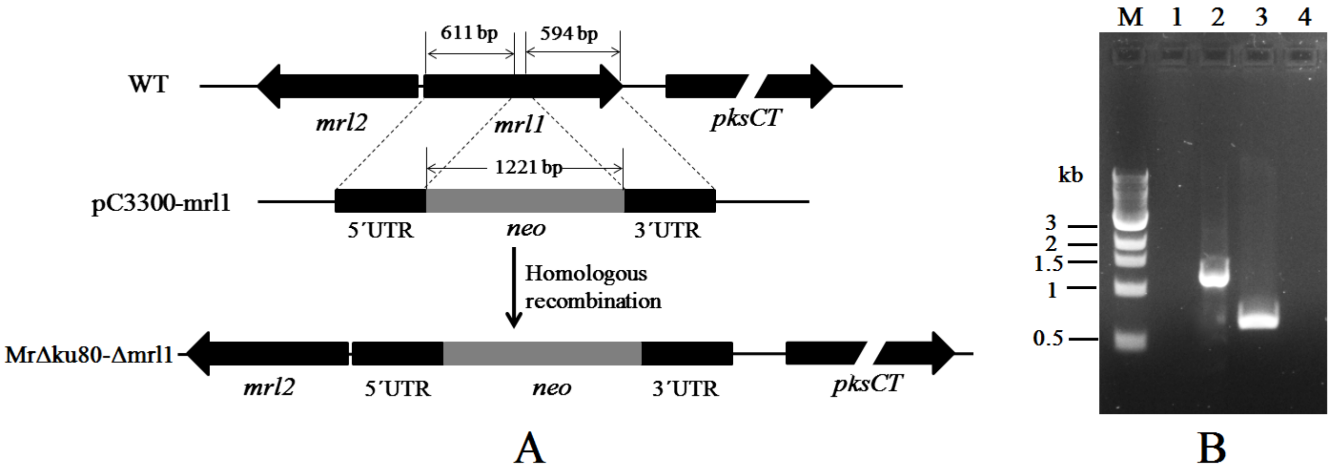

Figure S8.3 Scheme to knock-out *mrl1* (A) and PCR verification of Δ*mrl1*::*neo* mutant (B)

Lanes 1/3 and 2/4 were PCR results to amplify partial *mrl1* gene and *neo* with primers mrl1-VF/mrl1-VR and G418F/G418R respectively. Lane 1 and 2 were using gDNA of Δ*mrl1*::*neo* mutant as template, lane 3 and 4 were using gDNA of MrΔku80 strain as template. M: NEB 1 kb DNA ladder.

8.5.3 exp. 13: gene knock-out of *mrl2*. The similar strategy was used to inactivate *mrl2*. A 520 bp of 5′ fragment and a 489 bp of 3′ fragment of *mrl2* were amplified from *M. ruber* M7 genomic DNA using primers mrl2-5F/mrl2-5R and mrl2-3F/mrl2-3R respectively and served as homologous arms for recombination event (Fig. S8.4A). These two fragments together with the *neo* gene were reassembled as a knock-out cassette by homologous

recombination in *S. cerevisiae* with *Not*I and *Asc*I cut pE-YA vector and shuttled back into *E. coli* to create pE-YA-mrl2-KO. *Kpn*I and *Xba*I digestion and T4 DNA ligation of pE-YA-mrl2-KO and pCAMBIA3300 yielded pC3300-mrl2. The same ATMT method was used to obtain *mrl2* knock-out mutant (Δ*mrl2*::*neo*). Analytical PCR verified the homologous recombination at the right position in the *mrl2* knock-out mutant (Fig. S8.4B). Briefly, part of *mrl2* (537 bp) could only be amplified from the WT and *neo* could only be amplified from the *mrl2* knock-out mutant using primers mrl2-VF/mrl2-VR and G418F/G418R respectively, while the other two situations yielded no PCR products.

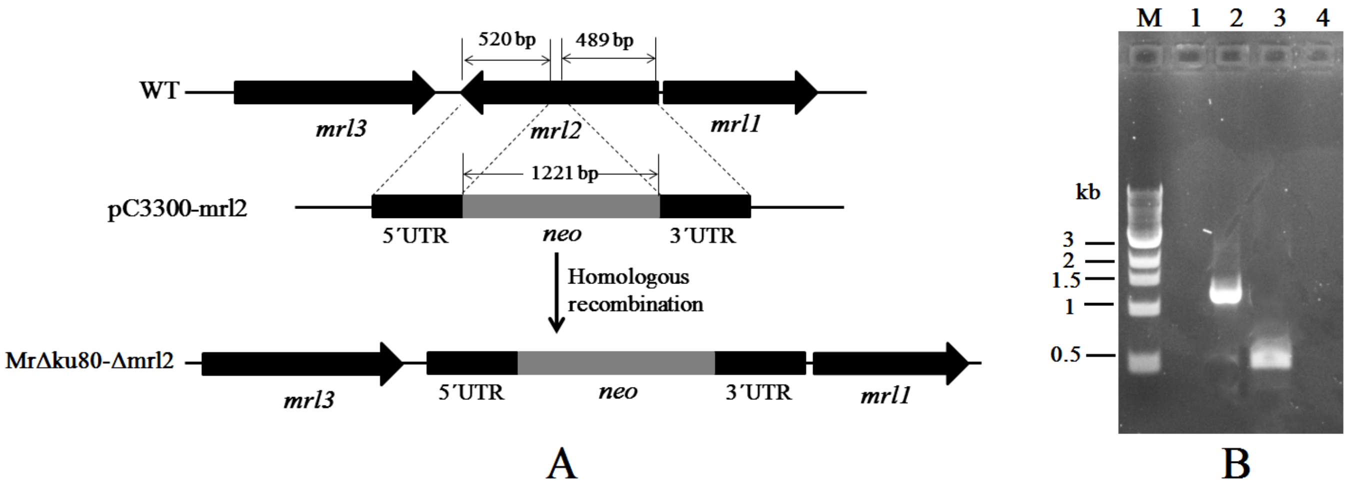

Figure S8.4 Scheme to knock-out *mrl2* (A) and PCR verification of Δ*mrl2*::*neo* mutant (B)

Lanes 1/3 and 2/4 were PCR results to amplify partial *mrl2* gene and *neo* with primers mrl2-VF/mrl2-VR and G418F/G418R respectively. Lane 1 and 2 were using gDNA of Δ*mrl2*::*neo* mutant as template, lane 3 and 4 were using gDNA of MrΔku80 strain as template. M: NEB 1 kb DNA ladder.

8.5.4 exp. 14: gene knock-out of *mrl4*. The similar strategy was used to inactivate *mrl4*. A 839 bp of 5′ fragment and a 520 bp of 3′ fragment of *mrl4* were amplified from *M. ruber* M7 genomic DNA using primers mrl4-5F/mrl4-5R and mrl4-3F/mrl4-3R respectively and served as homologous arms for recombination event (Fig. S8.5A). These two fragments together with the *neo* gene were reassembled as a knock-out cassette by homologous recombination in *S. cerevisiae* with *Not*I and *Asc*I cut pE-YA vector and shuttled back into *E. coli* to create pE-YA-mrl4-KO. *Xba*I and *Hin*dIII digestion and T4 DNA ligation of pE-YA-mrl4-KO and pCAMBIA3300 yielded pC3300-mrl4. The same ATMT method was used to obtain *mrl4* knock-out mutant (Δ*mrl4*::*neo*). Analytical PCR verified the homologous recombination at the right position in the *mrl4* knock-out mutant (Fig. S8.5B). Briefly, part of *mrl4* (740 bp) could only be amplified from the WT and *neo* could only be amplified from the *mrl4* knock-out mutant using primers

mrl4-VF/mrl4-VR and G418F/G418R respectively, while the other two situations yielded no PCR products.

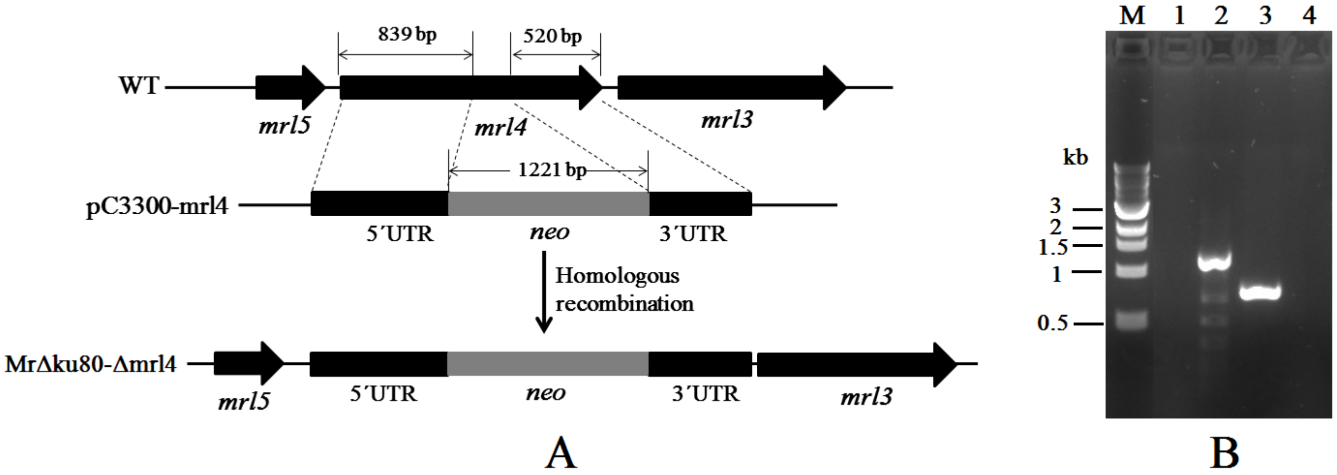

Figure S8.5 Scheme to knock-out *mrl4* (A) and PCR verification of Δ*mrl4*::*neo* mutant (B)

Lanes 1/3 and 2/4 were PCR results to amplify partial *mrl4* gene and *neo* with primers mrl4-VF/mrl4-VR and G418F/G418R respectively. Lane 1 and 2 were using gDNA of Δ*mrl4*::*neo* mutant as template, lane 3 and 4 were using gDNA of MrΔku80 strain as template. M: NEB 1 kb DNA ladder.

8.5.5 exp. 15: gene knock-out of *mrl6*. The similar strategy was used to inactivate *mrl6*. A 542 bp of 5′ fragment and a 576 bp of 3′ fragment of *mrl6* were amplified from *M. ruber* M7 genomic DNA using primers mrl6-5F/mrl6-5R and mrl6-3F/mrl6-3R respectively and served as homologous arms for recombination event (Fig. S8.6A). These two fragments together with the *neo* gene were reassembled as a knock-out cassette by homologous recombination in *S. cerevisiae* with *Not*I and *Asc*I cut pE-YA vector and shuttled back into *E. coli* to create pE-YA-mrl6-KO. *Kpn*I and *Xba*I digestion and T4 DNA ligation of pE-YA-mrl6-KO and pCAMBIA3300 yielded pC3300-mrl6. The same ATMT method was used to obtain *mrl6* knock-out mutant (Δ*mrl6*::*neo*). One mutant was confirmed by analytical PCR (Fig. S8.6B). Part of *mrl6* (829 bp) could only be amplified from the WT and *neo* could only be amplified from the *mrl6* knock-out mutant using primers mrl6-VF/mrl6-VR and G418F/G418R respectively, while the other two situations yielded no PCR products.

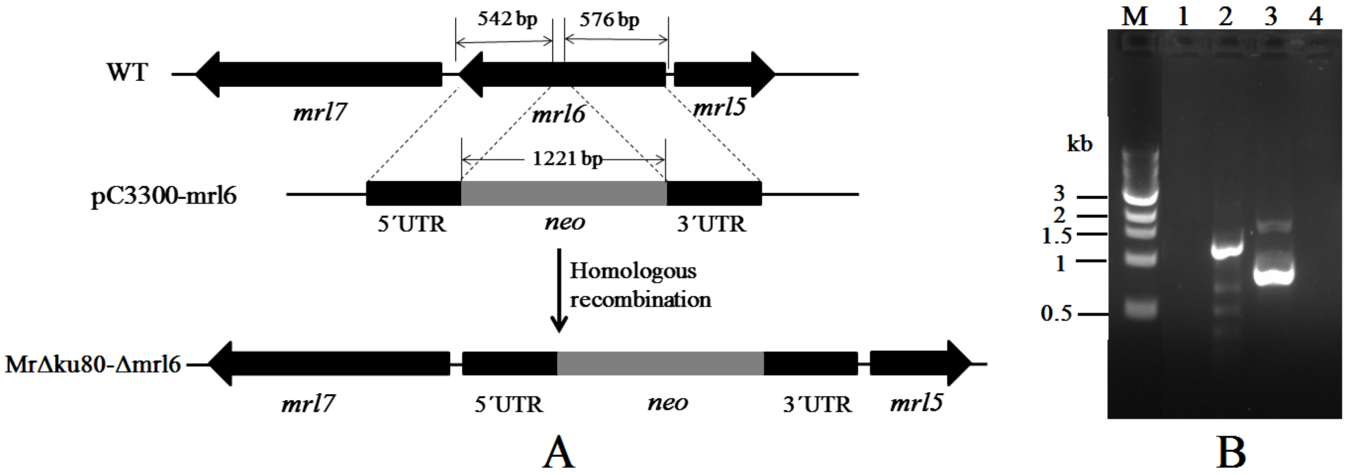

Figure S8.6 Scheme to knock-out *mrl6* (A) and PCR verification of Δ*mrl6*::*neo* mutant (B)

Lanes 1/3 and 2/4 were PCR results to amplify partial *mrl6* gene and *neo* with primers mrl6-VF/mrl6-VR and G418F/G418R respectively. Lane 1 and 2 were using gDNA of Δ*mrl6*::*neo* mutant as template, lane 3 and 4 were using gDNA of MrΔku80 strain as template. M: NEB 1 kb DNA ladder.

8.5.6 exp. 16: gene knock-out of *mrl7*. The similar strategy was used to inactivate *mrl7*. A 878 bp of 5′ fragment and a 536 bp of 3′ fragment of *mrl7* were amplified from *M. ruber* M7 genomic DNA using primers mrl7-5F/mrl7-5R and mrl7-3F/mrl7-3R respectively and served as homologous arms for recombination event (Fig. S8.7A). These two fragments together with the *neo* gene were reassembled as a knock-out cassette by homologous recombination in *S. cerevisiae* with *Not*I and *Asc*I cut pE-YA vector and shuttled back into *E. coli* to create pE-YA-mrl7-KO. *Kpn*I and *Xba*I digestion and T4 DNA ligation of pE-YA-mrl7-KO and pCAMBIA3300 yielded pC3300-mrl7. The same ATMT method was used to obtain *mrl7* knock-out mutant (Δ*mrl7*::*neo*). One mutant was confirmed by analytical PCR (Fig. S8.7B). Part of *mrl7* (491 bp) could only be amplified from the WT and *neo* could only be amplified from the *mrl7* knock-out mutant using primers mrl7-VF/mrl7-VR and G418F/G418R respectively, while the other two situations yielded no PCR products.

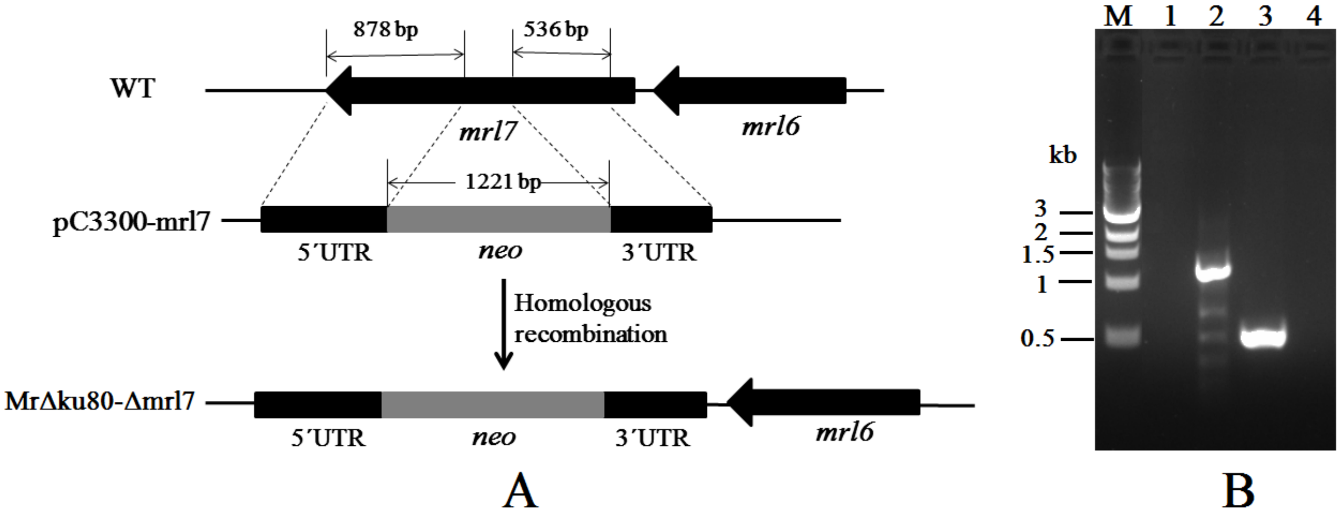

Figure S8.7 Scheme to knock-out *mrl7* (A) and PCR verification of Δ*mrl7*::*neo* mutant (B)

Lanes 1/3 and 2/4 were PCR results to amplify partial *mrl7* gene and *neo* with primers mrl7-VF/mrl7-VR and G418F/G418R respectively. Lane 1 and 2 were using gDNA of Δ*mrl7*::*neo* mutant as template, lane 3 and 4 were using gDNA of MrΔku80 strain as template. M: NEB 1 kb DNA ladder.

8.6 Cloning procedures for heterologous expression of different intron removed citS gene

8.6.1 exp. 17: construction of pTYGS·arg·*citS*·Δ62bp (*citS* removed both 56 bp intron 1 and 62 bp intron 2). The primers and the cloning procedures were the same with 8.4.1, the only difference was *M. ruber* M7 cDNA was used as PCR template. The constructed pTYGS·arg·*citS*·Δ62bp was sequenced to make sure both the 56 bp intron 1 and 62 bp intron 2 were correctly removed. Transformation of *A. oryzae* M-2-3 with this plasmid yielded 20 transformants.

8.6.2 exp. 18: construction of pTYGS·arg·*citS*·Δ62bp·*mrl1* (*citS* removed both 56 bp intron 1 and 62 bp intron 2, *mrl1*). The cloned *mrl1* gene was inserted into *Asc*I-*cut* pTYGS·arg·*citS*·Δ62bp under the control of P_{adh} and T_{eno} according to the same procedure in 8.4.2 to yield pTYGS·arg·*citS*·Δ62bp·*mrl1*. Transformation of *A. oryzae* M-2-3 with this plasmid yielded 12 transformants.

8.6.3 exp. 19: construction of pTYGS·arg·*citS*·Δ60bp (*citS* removed 56 bp intron 1 and 60 bp intron 2). The *citS* gene removed 56 bp intron 1 and

60 bp intron 2 was amplified from *M. ruber* M7 genomic DNA as four fragments using primers pks-1-F/pks-1-R, pks-2-F/pks-2-R, pks-3-F/pks-3-R-7189, pks-4-F-7250/pks-4-R and cloned into pTYGS·arg vector according to the same strategy described in 8.4.1 to yield pTYGS·arg·*citS*·Δ60bp. Transformation of *A. oryzae* M-2-3 with this plasmid yielded 17 transformants.

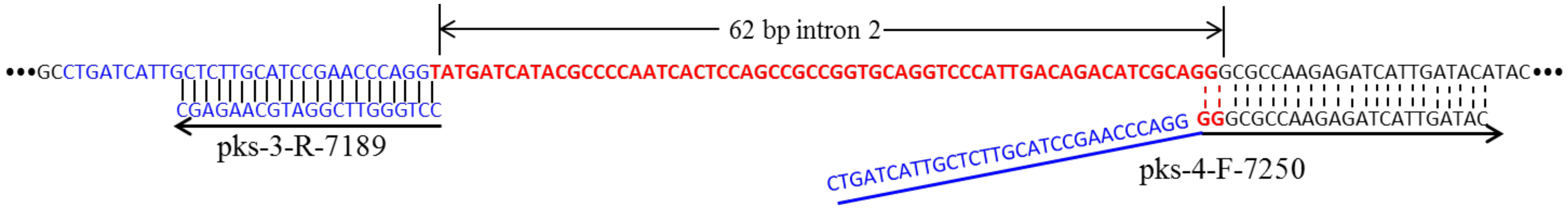

Figure S8.8 Cloning strategy to remove 60 bp intron 2

8.6.4 exp. 20: construction of pTYGS·arg·*citS*·Δ60bp·*mrl1* (*citS* removed 56 bp intron 1 and 60 bp intron 2, *mrl1*). The cloned *mrl1* gene was inserted into *Asc*I-*cut* pTYGS·arg·*citS*·Δ60bp under the control of P_{adh} and T_{eno} according to the same procedure in 8.4.2 to yield pTYGS·arg·*citS*·Δ60bp·*mrl1*. Transformation of *A. oryzae* M-2-3 with this plasmid yielded 24 transformants.

8.7 Cloning procedures to mutate cysteine to alanine at position 2551 in CitS protein

8.7.1 exp. 21: construction of pTYGS·arg·*citS*·C_{2551}A (*citS* with mutation of C_{2551} to A). A 349-bp DNA fragment containing mutation of TGT to GCC (C_{2551} to A) was synthesized. Then the *Xba*I cut pE-YA·*citS* was used to do yeast recombination in *S. cerevisiae* with the synthetic DNA fragment and shuttled back into *E. coli* to create pE-YA·*citS*·C_{2551}A. Gateway LR recombination between pE-YA·*citS*·C_{2551}A and pTYGS·arg transfered the mutant *citS* into pTYGS·arg to create pTYGS·arg·*citS*·C_{2551}A. Transformation of *A. oryzae* M-2-3 with this plasmid yielded 20 transformants.

8.7.2 exp. 22: construction of pTYGS·arg·*citS*·C_{2551}A·*mrl1* (*citS* with mutation of C_{2551} to A, *mrl1*). The cloned *mrl1* gene was inserted into *Asc*I-*cut* pTYGS·arg·*citS*·C_{2551}A under the control of P_{adh} and T_{eno} according to the same procedure in 8.4.2 to yield pTYGS·arg·*citS*·C_{2551}A·*mrl1*. Transformation of *A. oryzae* M-2-3 with this plasmid yielded 25 transformants.

8.8 Cloning procedures to mutate catalytic triad in mrl1 protein

8.8.1 exp. 23: construction of pTYGS·arg·*citS*·*mrl1*·S_{122}A (*citS*, *mrl1* with mutation of S_{122} to A). The *mrl1* gene was amplified from *M. ruber*

cDNA as two fragments using primers mrl1-F/mrl1-R-519 and mrl1-F-490/mrl1-R. The cloned two fragments were used to do yeast recombination in *S. cerevisiae* with *Asc*I-*cut* pTYGS·arg·*citS* and shuttled back into *E. coli* to create pTYGS·arg·*citS*·*mrl1*·S_{122}A. Transformation of *A. oryzae* M-2-3 with this plasmid yielded 11 transformants.

8.8.2 exp. 24: construction of pTYGS·arg·*citS*·*mrl1*·D_{207}A (*citS*, *mrl1* with mutation of D_{207} to A). The *mrl1* gene was amplified from *M. ruber* cDNA as two fragments using primers mrl1-F/mrl1-R-774 and mrl1-F-745/mrl1-R. The cloned two fragments were used to do yeast recombination in *S. cerevisiae* with *Asc*I-*cut* pTYGS·arg·*citS* and shuttled back into *E. coli* to create pTYGS·arg·*citS*·*mrl1*·D_{207}A. Transformation of *A. oryzae* M-2-3 with this plasmid yielded 23 transformants.

8.8.3 exp. 25: construction of pTYGS·arg·*citS*·*mrl1*·H_{235}A (*citS*, *mrl1* with mutation of H_{235} to A). The *mrl1* gene was amplified from *M. ruber* cDNA as two fragments using primers mrl1-F/mrl1-R-858 and mrl1-F-829/mrl1-R. The cloned two fragments were used to do yeast recombination in *S. cerevisiae* with *Asc*I-*cut* pTYGS·arg·*citS* and shuttled back into *E. coli* to create pTYGS·arg·*citS*·*mrl1*·H_{235}A. Transformation of *A. oryzae* M-2-3 with this plasmid yielded 19 transformants.

8.9 Fermentation conditions and extraction methods

8.9.1 *A. oryzae* NSAR1 and transformants

The *A. oryzae* NSAR1 transformants harboring pTYGS·arg·*citS* or pTYGS·arg·*citS*·*mrl1* were selected on MAM (0.2% (w/v) ammonium chloride, 0.1% (w/v) ammonium sulfate, 0.05% (w/v) potassium chloride, 0.05% (w/v) sodium chloride, 0.1% (w/v) monopotassium phosphate, 0.05% (w/v) magnesium sulfate, 0.002% (w/v) iron-(II)-sulfate heptahydrate, 2% (w/v) D(+)-glucose monohydrate, 0.15% (w/v) L-methionine, 0.01% (w/v) adenine, 2% (w/v) agar) plates. The *A. oryzae* NSAR1 transformants harboring pTYGS·arg·*citS*·*mrl1* and pTYGS·ade series vectors (pTYGS·ade·*mrl2*, pTYGS·ade·*mrl2*·*mrl4*, pTYGS·ade·*mrl2*·*mrl7*, pTYGS·ade·*mrl2*·*mrl6*, pTYGS·ade·*mrl2*·*mrl4*·*mrl7*, pTYGS·ade·*mrl2*·*mrl6*·*mrl7*, pTYGS·ade·*mrl2*·*mrl4*·*mrl6*, or pTYGS·ade·*mrl2*·*mrl4*·*mrl6*·*mrl7*) were selected on MAM plates without adenine. For extraction, the spores were collected from 5 days old growing *A. oryzae* NSAR1 or transformants strains and inoculated in each 100 mL MPM (0.2% (w/v) ammonium chloride, 0.1% (w/v) ammonium sulfate, 0.05% (w/v) potassium chloride, 0.05% (w/v) sodium chloride, 0.1% (w/v) monopotassium phosphate, 0.05% (w/v) magnesium sulfate, 0.002% (w/v) iron-(II)-sulfate heptahydrate, 2% (w/v) D(+)-maltose monohydrate, 1% (w/v) polypeptone, 0.01% (w/v) adenine, pH 5.5) liquid media contained in 500 mL Erlenmeyer flask. The spores were allowed to grow in the liquid culture for 5 days on shakers at 160 rpm at 28 °C.

The MPM fermentation broth was filtered to remove the mycelium and acidified to pH 4.0 using 37% HCl and then transferred into a separating funnel. An equal volume of ethyl acetate was added into the separating funnel and shaken vigorously. The mixture was allowed to stand to separate the

layers. After taking out the organic layer, the rest water layer was extracted with equal volume ethyl acetate again. The organic phase from two extractions was dried ($MgSO_4$), filtered and evaporated to dryness. The crude extract was dissolved in 2 mL HPLC grade MeOH and analysed by LC-MS.

8.9.2 *M. ruber* and mutants

The wild-type *M. ruber* M7 or MrΔku80 strains were grown on PDA (2.4%(w/v) potato dextrose broth, 1.5% (w/v) agar) plates for 7-10 days at 28 °C for spores production. The mutants obtained through ATMT method were selected on PDA plates with 15 μg/mL G418. For extraction, the spores were collected from 10 days old growing MrΔku80 or mutants strains and inoculated in each 100 mL PDB (2.4%(w/v) potato dextrose broth) liquid media contained in 500 mL Erlenmeyer flask. The spores were allowed to grow in the liquid culture for 10 days on shakers at 160 rpm at 28 °C. The extraction method of PDB fermentation broth was the same with MPM fermentation broth.

8.9.3 *A. oryzae* M-2-3 and transformants

The *A. oryzae* M-2-3 transformants were selected on Czapek-Dox (3.5% (w/v) Czapek Dox broth, 4.68% (w/v) sodium chloride, 0.1% (w/v) ammonium sulfate, 0.05% (w/v) adenine, 0.15% (w/v) L-methionine, 1.5% (w/v) agar) plates. For extraction, the spores were collected from 5 days old growing *A. oryzae* M-2-3 or transformants strains and inoculated in each 100 mL CMP (3.5% (w/v) Czapek Dox broth, 2% (w/v) D(+)-maltose monohydrate, 1% (w/v) polypeptone) liquid media contained in 500 mL Erlenmeyer flask. The spores were allowed to grow in the liquid culture for 5 days on shakers at 160 rpm at 28 °C. The extraction method of CMP fermentation broth was the same with MPM fermentation broth.

8.10 Fungal transformation methods

8.10.1 Transformation of *A. oryzae* NSAR1

Plasmid DNA for fungal transformation was prepared using Fermentas Miniprep kits. *A. oryzae* NSAR1 or *A. oryzae* NSAR1 harboring pTYGS·arg·*citS*·*mrl1* strains were grown on DPY plates for 10 days. Spores washed by 4 mL sterile water were inoculated into 100 mL DPY liquid medium and cultivated for 2 day at 28 °C. Collect the mycelia on a sterile filter paper (autoclaved with a filter funnel) and wash with sterile water, then 0.8 M NaCl. Put the mycelia in a sterile falcon centrifuge tube. Add 10 mL of filter-sterilized TF buffer 1 (10 mg/mL Yatalase (Takara), 0.6 M $(NH_4)_2SO_4$, 50 mM maleic acid, pH 5.5) and incubate at 30 °C, 100rpm for 2 hours. Filter the protoplasting solution through a syringe with glasswool inside. Centrifuge the filtrate at 3000 rpm for 10 min. Wash the pelleted protoplasts with 15 mL TF buffer 2 (1.2 M sorbitol, 50 mM $CaCl_2$, 35 mM NaCl, 10 mM Tris HCl pH 7.5). Resuspend the protoplasts in TF buffer 2 to final concentration of 2.5×10^8/mL. Put 0.2 mL portions into Falcon tubes. Add 20 μL plasmid DNA and place on ice for 30 min. Add 250 μL, 250 μL and 850 μL TF buffer 3 (PEG 4000 (60% w/v), 50 mM $CaCl_2$, 10 mM Tris HCl pH 7.5), mix well gently and place at room temperature for 20 min. 10 mL soft agar (0.8% agar containing 5% NaCl) was added to the

transformation mixtures, and then poured onto MPM selection plates supplemented with sorbitol (1 M) and incubated at 28˚C for 5-7 days.

8.10.2 Transformation of *A. oryzae* M-2-3

Plasmid DNA for fungal transformation was prepared using Fermentas Miniprep kits. *A. oryzae* M-2-3 was grown on MEA plates for 10 days. Spores washed by 4 mL sterile water were inoculated into 100 mL GNB liquid medium (2% glucose, 1% nutrient broth number 2 (from Thermo Scientific)) and cultivated for 2 day at 28 °C. Collect the mycelia on a sterile filter paper (autoclaved with a filter funnel) under vacuum and wash with sterile water, then 0.8 M NaCl. Put the mycelia in a sterile falcon centrifuge tube. Add 10 mL of filter-sterilized protoplasting solution (20 mg/mL lysing enzyme (Sigma L1412), 10 mg/mL driselase (Sigma D9515), 0.8 M NaCl, 10 mM Na phosphate buffer pH 6) and incubate at 30 °C, 100rpm for no longer than 3 hours. Filter the protoplasting solution through a syringe with glasswool inside. Centrifuge the filtrate at 3000 rpm for 10 min. Wash the pelleted protoplasts once with 0.8 M NaCl (ca. 15 mL) and then once with Solution 1 (0.8 M NaCl, 10 mM $CaCl_2$, 50 mM Tris HCl pH 7.5). Resuspend the protoplasts in Solution 1 to final concentration of 2.5 $\times 10^8$/mL and add 1/5 volume of Solution 2 (PEG 4000 (60% w/v) in Solution 1 but 50 mM $CaCl_2$). Put 0.2 mL portions into Falcon tubes. Add 20 µL plasmid DNA and place on ice for 30 min. Add 1 mL of Solution 2, mix well gently and place at room temperature for 20 min. 10 ml soft agar (0.8% agar containing 5% NaCl) was added to the transformation mixtures, and then poured onto Czapek-Dox plates supplemented with sorbitol (1 M) and incubated at 28˚C for 5-7 days.

8.11 Transformation of S. cerevisiae for yeast recombination

A single colony of *S. cerevisiae* YPH499 was inoculated into a 10 mL YPAD (1% (w/v) yeast extract, 2% (w/v) bactotryptone, 2% (w/v) glucose, 0.04% (w/v) adenine sulphate) starter culture and grown overnight at 28 °C with shaking at 200 rpm. The starter culture was then added to 40 mL of YPAD in a 250 mL flask and incubated at 28 °C with shaking at 200 rpm for 5 hours, after which the culture was centrifuged at 3000 g for 5 min and the supernatant discarded. The cells were washed with 25 mL sterile H_2O and the centrifugation repeated, the pellet was then resuspended in 1 mL 0.1 M LiOAc and transferred to a 1.5 mL microfuge tube. The cells were then pelleted at 14500 rpm for 15 sec and the supernatant discarded, after which the cells were resuspended in 400 µL 0.1 M LiOAc. For each transformation to be performed 50 µL of the suspension was transferred to a new 1.5 mL microfuge tube and pelleted again at 14500 rpm for 15 sec and the supernatant discarded. 240 µL of PEG solution (50% (w/v) polyethylene glycol 3350), 36 µL 1 M LiOAc, 20 µl SS-DNA (Salmon Sperm DNA, 5 mg/ml in TE buffer, Rockland MB-103-0025) and up to 34 µL of DNA were added to the pelleted cells in order. Approximately 0.5 - 1 µg of each DNA fragment was added, with linear DNA fragments to be joined containing at least 30 bp overlap. Cells were resuspended in the transformation mixture by vortexing, and incubated at 30 °C for 30 min and then 42 °C for 30 min. The cells were pelleted at 6000 rpm for 15 sec then gently resuspended in 1 mL of sterile water. 200 µL aliquots were spread on SM-URA plates (0.17% (w/v) yeast nitrogen base, 0.5% (w/v) ammonium sulphate, 2% (w/v) glucose, 0.077% (w/v) complete supplement mixture minus uracil (Q-biogene), 1.5% (w/v) agar) and incubated at 28 °C for 3-4 days until colonies appeared.

Table S2 List of primers used in this study

Name	Sequence (5´-3´)
pks-1-F	GCCAACTTTGTACAAAAAAGCAGGCTCCGCATGATTGACTCAACTTCGCACTC
pks-1-R	TCTCTGGTCAACGATGACAGACACATATGCATCAGGGAATGTCTCG
pks-2-F	GCATATGTGTCTGTCATCG
pks-2-R	CGTGATGGTGGAGTTC
pks-3-F	CTGTTGCCATTTGGTTAGAGG
pks-3-R	ATCCATACCGCATTGATAGGAG
pks-4-F	TGCCAAATCTCCTATCAATGCGGTATG
pks-4-R	TGCCAACTTTGTACAAGAAAGCTGGGTCGGTTAATCTAGAAATCCCATG
mrl1-F-long	TTTCTTTCAACACAAGATCCCAAAGTCAAAATGAAAGGGCAGACAGGGCTTC
mrl1-F	TTTCTTTCAACACAAGATCCCAAAGTCAAAATGGTCCAGACGAATTTAGAGG
mrl1-R	AGGTTGGCTGGTAGACGTCATATAATCATACCTAGGGAGCACCCGTCTGCGTTG
mrl2-F	TTTCTTTCAACACAAGATCCCAAAGTCAAAATGCCCATCTCAACCAAGTC
mrl2-R	TTCATTCTATGCGTTATGAACATGTTCCCTTTACTTTACTTTGAGATTG
mrl2-R-b	AGGTTGGCTGGTAGACGTCATATAATCATATTACTTTACTTTGAGATTG
mrl4-F	AACAGCTACCCCGCTTGAGCAGACATCACCATGGCCGAAGCAGCAGC
mrl4-R	ACGACAATGTCCATATCATCAATCATGACCCTACAACTTGCATACATC
mrl4-R-b	AGGTTGGCTGGTAGACGTCATATAATCATACTACAACTTGCATACATC
mrl6-F	TCGACTGACCAATTCCGCAGCTCGTCAAAGATGGCCTTTCCACCG
mrl6-F-b	AACAGCTACCCCGCTTGAGCAGACATCACCATGGCCTTTCCACCG
mrl6-R	AGGTTGGCTGGTAGACGTCATATAATCATACTACAGAACCAACTTG
mrl7-F	GCCAACTTTGTACAAAAAAGCAGGCTCCGCATGGCCACAGTCAAGGTCATCG
mrl7-R	TGCCAACTTTGTACAAGAAAGCTGGGTCGGTTATATATGAGCACGGAGTCG
G418F	CCAACTCAACCCCATCGAACCGTAACC
G418R	ATCATCATGCAACATGCATG
pksCT-5F	GCCAACTTTGTACAAAAAAGCAGGCTCCGCGTCGACCACAGACCTACCCGATGAGC
pksCT-5R	TGGGGTTACGGTTCGATGGGGTTGAGTTGGAAACACGGCACCAACACC

pksCT-3F	TCAGACAGTACATGCATGTTGCATGATGATCGGAACCTGGAAATCTCAAC
pksCT-3R	TGCCAACTTTGTACAAGAAAGCTGGGTCGGAAGCTTGACGGAATCTGCGGTCATAG
pksCT-VF	CTGTTGCCATTTGGTTAGAGG
pksCT-VR	CGCTTACCGCAGTAGACGA
mrl1-5F	GCCAACTTTGTACAAAAAAGCAGGCTCCGCGGTACCATGCCACGCCACTTTCTG
mrl1-5R	TGGGGTTACGGTTCGATGGGGTTGAGTTGGCCCATTGACTGTACACTG
mrl1-3F	TCAGACAGTACATGCATGTTGCATGATGATTCCGAGCGATTGATGACTG
mrl1-3R	TGCCAACTTTGTACAAGAAAGCTGGGTCGGTCTAGACTCTAACGCCCGTGACACC
mrl1-VF	ATGCCACGCCACTTTCTG
mrl1-VR	CGAAGCCAACGTCTGAAC
mrl2-5F	GCCAACTTTGTACAAAAAAGCAGGCTCCGCGGTACCTCATTTTCCGCTGAAAG
mrl2-5R	TGGGGTTACGGTTCGATGGGGTTGAGTTGGGCCCACTTCGACTGCTCC
mrl2-3F	TCAGACAGTACATGCATGTTGCATGATGATCCAACACTACCCAGCACAGCTTC
mrl2-3R	TGCCAACTTTGTACAAGAAAGCTGGGTCGGTCTAGAATAGAATGCCCATCTCAACCAAG
mrl2-VF	TCATTTTCCGCTGAAAG
mrl2-VR	GGAAAACGATCCTGCAT
mrl4-5F	GCCAACTTTGTACAAAAAAGCAGGCTCCGCTCTAGATATCAGGCAAGATTACCAGAACCA
mrl4-5R	TGGGGTTACGGTTCGATGGGGTTGAGTTGGCAGGTCGGCGTCCTCAAAGA
mrl4-3F	TCAGACAGTACATGCATGTTGCATGATGATCTGTCGTACATCGAGCAAGGC
mrl4-3R	TGCCAACTTTGTACAAGAAAGCTGGGTCGGAAGCTTGGGTATTCCCGCTGTCCATCA
mrl4-VF	GCCAGGAGCGGTCACTCTATCT
mrl4-VR	CGCCCGTTCGCAGTTTCTT
mrl6-5F	GCCAACTTTGTACAAAAAAGCAGGCTCCGCGGTACCTTCTTATACGAAACGACGTACAAACG
mrl6-5R	TGGGGTTACGGTTCGATGGGGTTGAGTTGGATGACGAGGGCCATGCTG
mrl6-3F	TCAGACAGTACATGCATGTTGCATGATGATTCTCCGCAAGCGGGACCCAT
mrl6-3R	TGCCAACTTTGTACAAGAAAGCTGGGTCGGTCTAGACCAGCAGGAACCACGACCTA
mrl6-VF	GGTACGTGCCCTTGAGGTTG
mrl6-VR	GGTCTTGCGTCGTGTTTCTTTA

mrl7-5F	GCCAACTTTGTACAAAAAAGCAGGCTCCGCGGTACCCCATCGTTGCGACTACATCAC
mrl7-5R	TGGGGTTACGGTTCGATGGGGTTGAGTTGGACTCCATACTCGTCACTATCCACC
mrl7-3F	TCAGACAGTACATGCATGTTGCATGATGATACTCCTTCCGCGACGGGTAT
mrl7-3R	TGCCAACTTTGTACAAGAAAGCTGGGTCGGTCTAGAGCCAATGCGAAAGCGTATCA
mrl7-VF	TGTTGCTGACGACGATGGAG
mrl7-VR	TGGGATTGCTTGTAGAGTGGC
pks-3-R-7189	CCTGGGTTCGGATGCAAGAGC
pks-4-F-7250	CTGATCATTGCTCTTGCATCCGAACCCAGGGGGCGCCAAGAGATCATTGATAC
mrl1-R-519	AAATCCAAGCAATCCAACCCAC
mrl1-F-490	ACAGGAGCGTGGGTTGGATTGCTTGGATTTGCCCAAGGCGCGAAGATGTGCGC
mrl1-R-774	TCGCATCCCGTGTACATGCACG
mrl1-F-745	ATTCCCACCGTGCATGTACACGGGATGCGAGCCCCCACGTGGACCTTCACCG
mrl1-R-858	GTCACCATCCCACTCTACTAGTC
mrl1-F-829	AGCAGGAGACTAGTAGAGTGGGATGGTGACGCCCGGGTTCCGCTGAAGTACAATG

9. Previous Questionable Biosynthetic Proposals for Citrinin from the Literature.

Figure S9.1 The biosynthesis of citrinin proposed by Hajjaj and coworkers.[1]

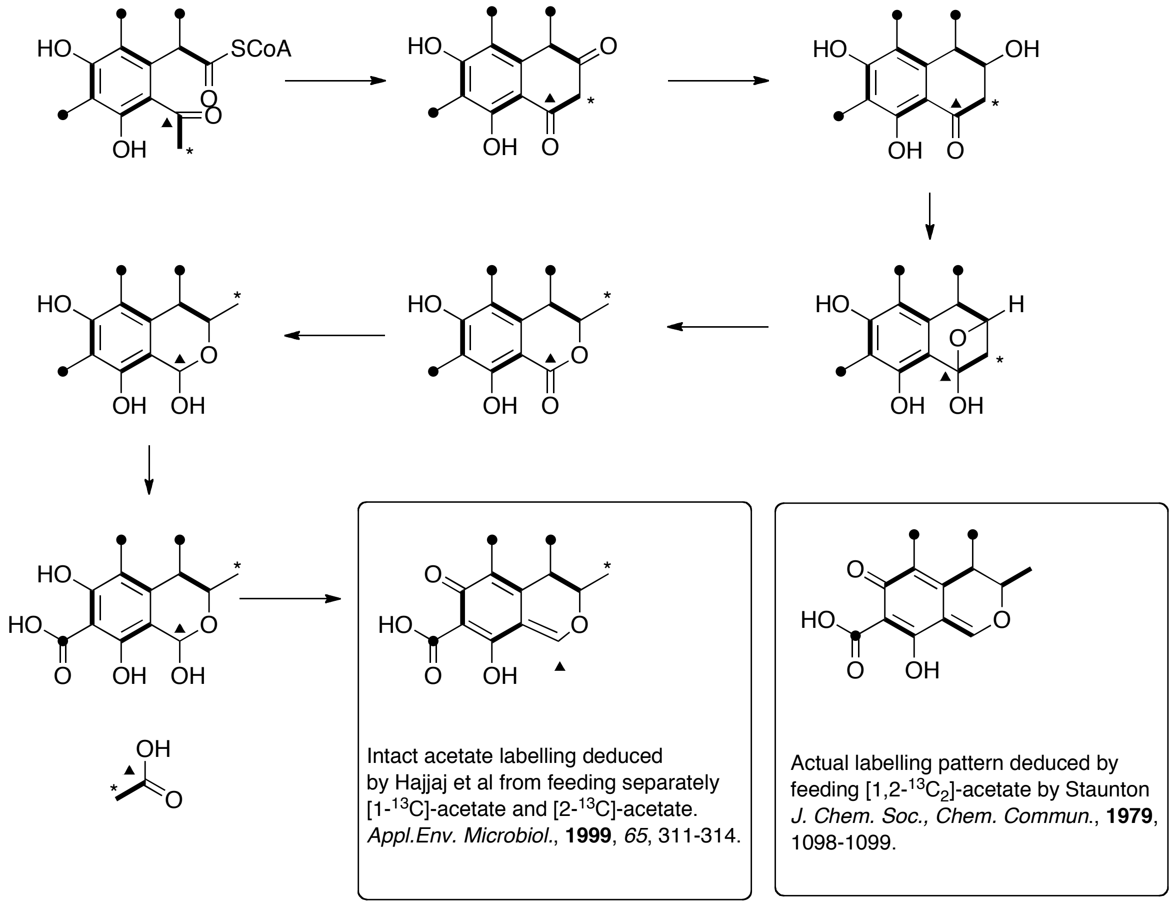

Figure S9.2 Biosynthetic route to Citrinin proposed by Li and coworkers.[2]

N.b. *ctnB* is also incorrectly referred to as encoding an oxidoreductase in this paper.

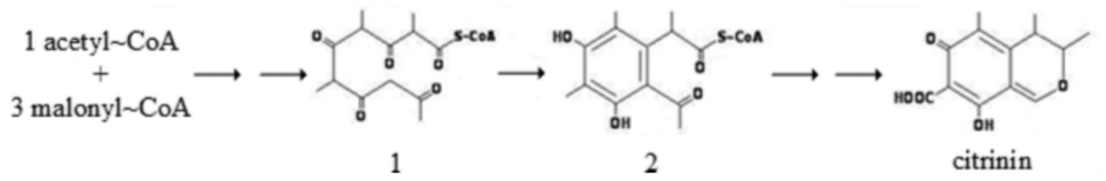

Figure 2. Proposed pathway participated by the CtnB protein.

Figure S9.3. Biosynthetic route to citrinin and other azaphilones proposed by Woo and coworkers.[3]

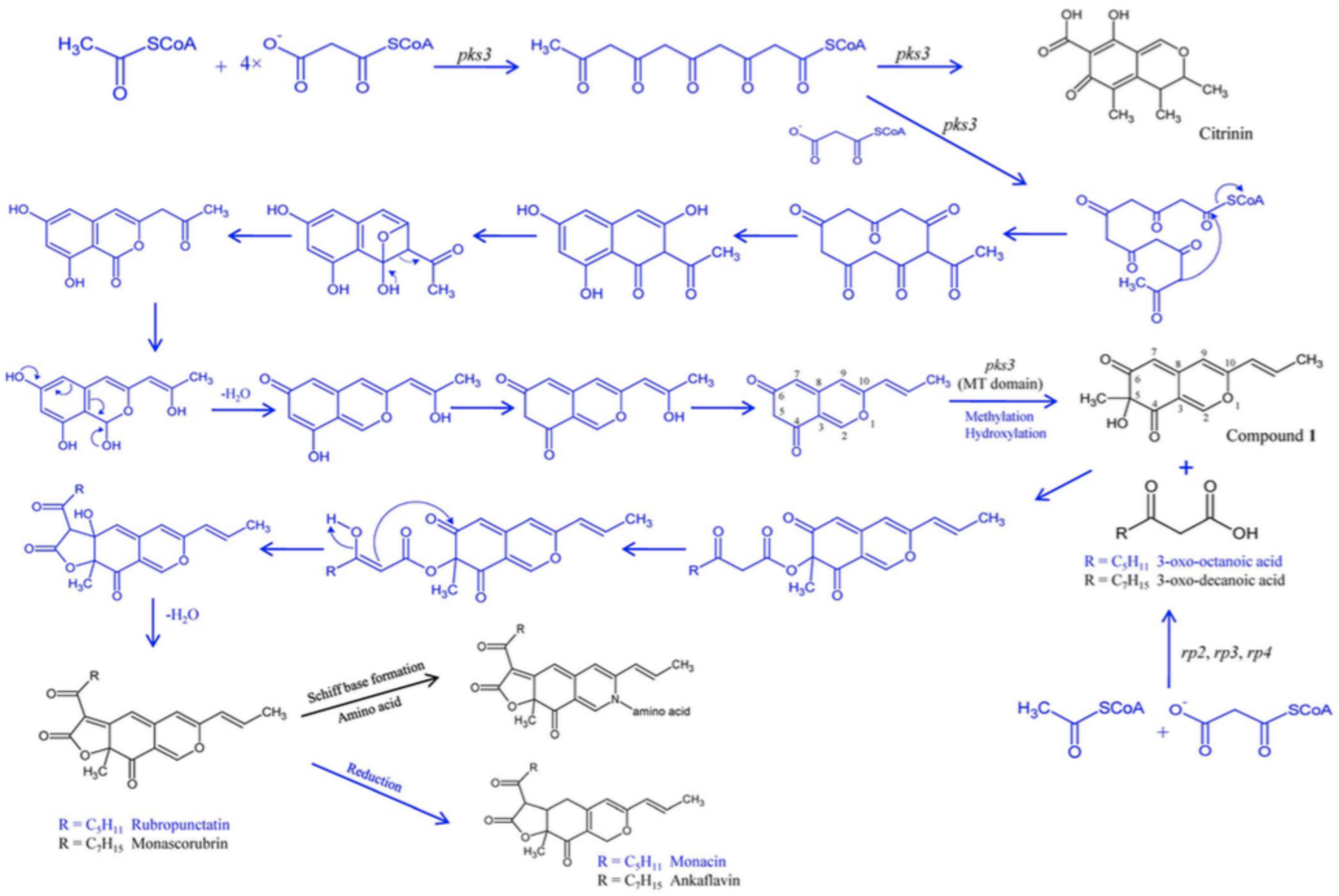

Figure 5 | Hypothetical pathway for monascorubrin, ankaflavin and citrinin biosynthesis in *P. marneffei*. Compounds and the five genes of the red pigment biosynthesis cluster identified in this study are in black. Hypothetical compounds, intermediates and pathways are depicted in blue.

10. 3D Model of CitS Reductive Release Domain

10.1 Multiple Alignment

Peptide sequences for *M. ruber* CitS, *M. purpureus CitS*, *A. strictum pks1* (MOS) and the myxalamid R-domain (4U7W) were aligned using ClustalW (standard parameters). Red boxes show conserved catalytic Y and K residues and NADPH binding region. C2551 is conserved in the PKS R-domains but not the myxalamid R-domain.

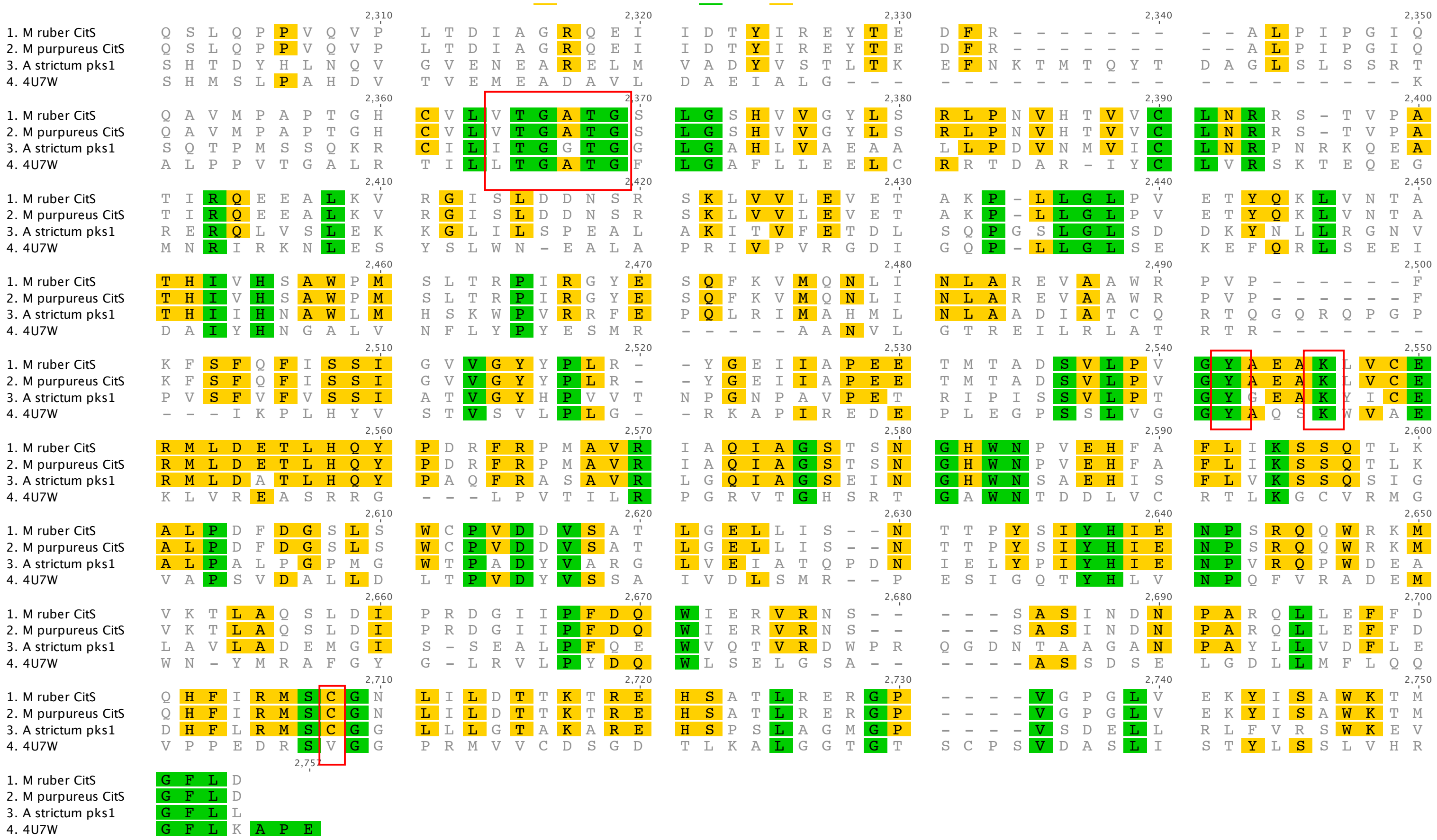

10.2 3D model

CitS residies 2305 - 2593were submitted to SwissModel[4] and a model structure was built using the myxalamid structure 4U7W using standard parameters. Data was visualised using the software iMol.

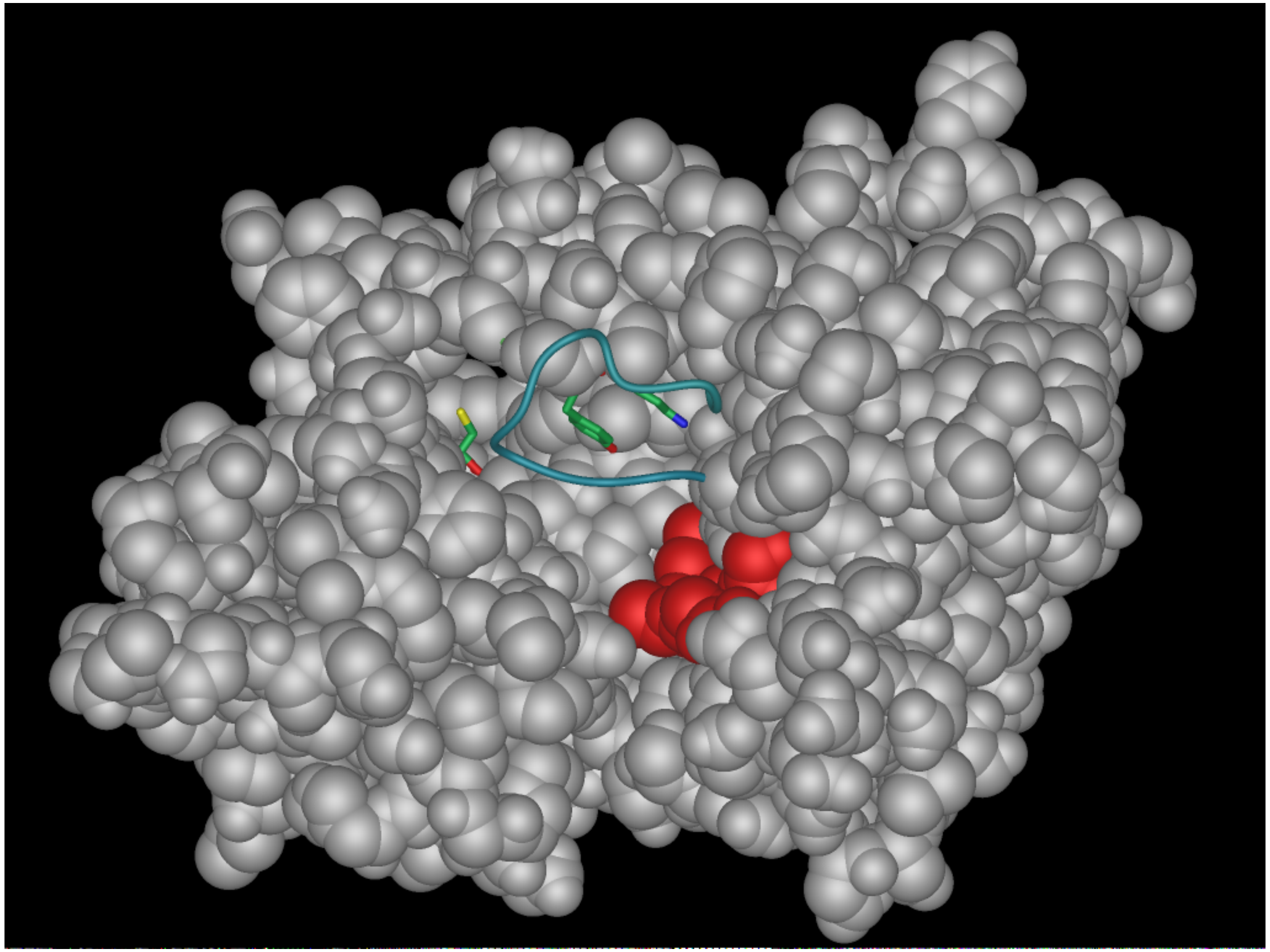

View of R-domain active site showing the conserved residues: C2551 (sticks, rear); Y2392 (sticks, centre); and K2396 (sticks, front). Residues on loop W2316 - Q2330 removed for clarity. All other residues shown as spheres. Red spheres, NADPH binding residues.

11. Presumed Mechanism of CitE / mrl6

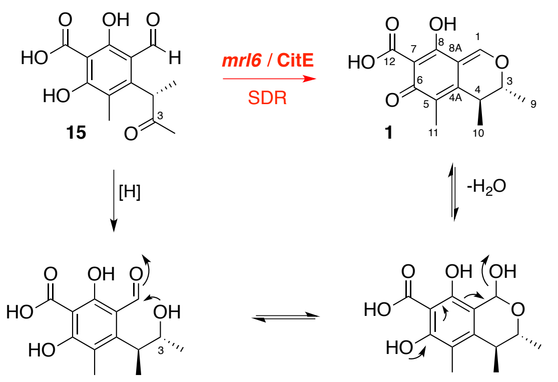

12. References

1. H. Hajjaj, A. Klaebe, M. Loret, G. Goma, P. Blanc and J. Francois, *Appl. Environ. Microb.*, 1999, **65**, 311–314.
2. Y.-P. Li, Y.-F. Pan, L.-H. Zou, Y. Xu, Z.-B. Huang and Q.-H. He, *J. Agr. Food Chem.*, 2013, **61**, 7397–7402.
3. P. C. Y. Woo, C.-W. Lam, E. W. T. Tam, K.-C. Lee, K. K. Y. Yung, C. K. F. Leung, K.-H. Sze, S. K. P. Lau and K.-Y. Yuen, *Sci. Rep*. 2014, **4**, 6728–8.
4. M. Biasini, S. Bienert, A. Waterhouse, K. Arnold, G. Studer, T. Schmidt, F. Kiefer, T. G. Cassarino, M. Bertoni, L. Bordoli, T. Schwede, *Nucleic Acids Research*, 2014, 42 (W1): W252-W258; K. Arnold, L. Bordol, J. Kopp, and T. Schwede T., *Bioinformatics*, 2006, 22, 195-201; F. Kiefer, K. Arnold, M. Künzli, L. Bordoli and T. Schwede, *Nucleic Acids Research*. 2009, 37, D387-D392; N. Guex, M. C. Peitsch and T. Schwede, *Electrophoresis*, 2009, 30(S1), S162-S173.
